# Few Ramachandran Angle Changes Provide Interaction Strength Increase in Aβ42 versus Aβ40 Amyloid Fibrils

**DOI:** 10.1038/srep36499

**Published:** 2016-11-03

**Authors:** Oscar H. Bastidas, Benjamin Green, Mary Sprague, Michael H. Peters

**Affiliations:** 1 Department of Chemical and Life Science Engineering, Virginia Commonwealth University, Richmond, Virginia, United States of America

## Abstract

The pathology of Alzheimer’s disease can ultimately be traced to the increased aggregation stability of Aβ42 peptides which possess two extra residues (Ile 41 & Ala 42) that the non-pathological strain (Aβ40) lacks. We have found Aβ42 fibrils to exhibit stronger energies in inter-chain interactions and we have also identified the cause for this increase to be the result of different Ramachandran angle values in certain residues of the Aβ42 strain compared to Aβ40. These unique angle configurations result in the peptide planes in the fibril structures to be more vertical along the fibril axis for Aβ42 which thus reduces the inter-atomic distance between interacting atoms on vicinal peptide chains thereby increasing the electrostatic interaction energies. We lastly postulate that these different Ramachandran angle values could possibly be traced to the unique conformational folding avenues sampled by the Aβ42 peptide owing to the presence of its two extra residues.

The neurodegeneration that marks the onset of Alzheimer’s disease (AD) is believed to be caused by neurotoxic soluble aggregate oligomers of Aβ42 peptides that result from the cleavage of the Alzheimer precursor protein^1^. In this disorder, Aβ42 peptides co-exist with the more benign Aβ40 peptide but at a greater Aβ42/Aβ40 ratio, where the Aβ42 is much more pathogenic^2^,^3^,^4^. This has recently been demonstrated to be clinically significant as measurements of Aβ42/Aβ40 ratios in cerebrospinal fluid can function as an important clinical diagnostic marker of AD^5^. Aβ42’s pathogenicity is consequently owed to its greater neuronal toxicity, greater aggregation propensity, and its increased kinetics or rate of aggregate formation compared to Aβ40^6^,^7^,^8^,^9^,^10^. Small, prefibrillar/oligomeric aggregate species of Aβ42 are now recognized as the primary neurotoxic species responsible for neuronal death in Alzheimer’s disease^11^,^12^,^13^,^14^,^15^ although there is also a recognized appreciable toxicity of Aβ42 mature fibrils as has been seen in cell culture experiments^16^,^17^,^18^,^19^. In this regard, mature fibril aggregates are also known to act as a source of these toxic pre-fibrillar oligomers and aggregates in aggregation pathway schemes^20^,^21^,^22^. Neurotoxicity is thought to proceed by Aβ42 targeting the synapse of neurons^23^ with the likely mechanism of cell death being apoptosis^24^.

Despite the obvious clinical importance of both oligomers and fibrils, their structural details at the atomic level have been unfortunately difficult to characterize^12^,^25^,^26^,^27^,^28^. It is known, however, that in these aggregate species, the constituent peptide chains, or monomers, of both Aβ40 and Aβ42 are held together by hydrogen bonds that stabilize the aggregate formation^29^,^30^,^31^. In spite of the enigmatic nature of Aβ40 and Aβ42 oligomeric structure, there is a recognized considerable difference in morphology between Aβ40 and Aβ42 aggregates as evidenced by aggregation seeds of one strain failing to initiate fibril formation in the other^32^,^33^,^34^. These differences in morphology have been observed in experimentally determined structures of Aβ40 and Aβ42 mature fibrils, which demonstrate distinct differences in both their conformations and size, or number of constituent monomer Aβ chains. The addition of only two residues, as seen in the Aβ42 strain, significantly alters the folded state conformations of the monomer chains in the mature fibril structure. Such distinctions have even been proposed as clinically significant in their exploitation as potential novel biomarkers for diagnosing late phase Alzheimer’s disease specifically exploiting the more β-sheet rich Aβ42 isoform^35^. A comparison of the structural and energetic properties of mature Aβ40 and Aβ42 fibrils may therefore provide insight into key differences between the two isoforms and thus help to establish the structural and energetic constraints on their fibril formation pathways. Such a comparative analysis may in turn help to characterize the long-term stability and associated neuronal toxicity of Aβ42. Additionally, identifying the underlying key features of the differences of the two strains may provide insight into new therapeutic approaches to inhibit Aβ42 aggregation formation. We therefore attempt to identify and characterize the underlying behavior of inter-peptide chain non-covalent interactions by carrying out detailed atomic level energy mappings for both Aβ40 and Aβ42 mature fibril structures. In doing so, we aim to answer the following two questions: (1) What are the key differences in the inter-chain interaction energetic profiles of Aβ40 fibrils and Aβ42 fibrils? and (2) What are the underlying conformational changes that are responsible for these differences?

## Results

For our study, we initially analyzed and compared the fibril structures of Aβ40 (PDB ID: 2M4J by Lu *et al.*^36^) and Aβ42 (PDB ID: 2MXU by Xiao *et al.*^32^) according to the Coulombic (charge and partial atomic charge) and Lennard-Jones (Born and van der Waals forces) atom-atom interaction forces as laid out in the open-source energy mapping algorithm developed by Krall, Brunn, Kankanala and Peters^37^. This mapping algorithm efficiently parses the strongest non-covalent atom-atom interactions and their inter-atomic distances from structure file data according to empirically established criteria based on the AMBER 03 force field model to ensure that all dominant interactions are accounted for^37^,^38^,^39^. Those parsing criteria were taken as the upper limit of −0.1 *kT* units for Lennard-Jones criteria and −0.3 *kT* units for Coulombic interactions^37^. The Aβ40 PDB structure file was composed of three Aβ40 peptide stacks, each stack containing three Aβ40 peptide chains, arranged in a triangular three-fold symmetry^36^ whereas the Aβ42 structure was comprised of only one stack possessing twelve Aβ42 peptide chains^32^. These two published structures were selected for our analysis due to the fact that they are believed to represent the *in vivo* forms of mature fibrils^32^,^36^; very recently, two additional structures of mature Aβ42 fibrils have also appeared (PDB ID: 2NAO by Walti *et al.*^40^ and PDB ID: 5KK3 by Colvin *et al.*^41^), which are analyzed and compared following the present analysis of 2MXU. Given the ensemble nature of the structure data for both isoforms, the mapping results for each ensemble member were averaged to obtain the data reported here in the form of 95% confidence intervals for each isoform (questions on how we specifically processed the data files can be directed to the corresponding author). For the Aβ40 structure, we found the results of our energy mappings for each stack to be virtually identical, deviating by only a few percent. Thus, we present the data of the A-D-G stack as representative for what we observed for the entire Aβ40 isoform. Our energy mappings thus involved the investigation of any two inter-chain interaction configurations within one Aβ peptide chain stack: 1) mapping the atom-atom interactions between consecutive/vicinal chains (1:2 interactions) and 2) mapping the atom-atom interactions between non-vicinal chains (1:3 interactions, 1:4, etc…). We also note that, within each isoform, the mapping results between any 1:2, 1:3, etc… chain interactions in the fibril structures were virtually identical to the results of other 1:2, 1:3, etc… interaction systems in the respective fibril structure; so, we report the results for the mapping of the first two chains of each isoform as representative data for their respective strains (mapping chains A-D and A-G for 1:2 and 1:3 interactions respectively for Aβ40 and mapping chains A-B and A-C for 1:2 and 1:3 interactions respectively for Aβ42). The results of the energy mappings found that the Aβ42 isoform has appreciably stronger inter-chain atom-atom interaction binding energies and smaller inter-atomic distances than Aβ40 for both 1:2 and 1:3 interactions thus implying its superior aggregate stability. Interestingly, we observed that for the 1:2 interactions, despite Aβ42 having stronger inter-chain atom-atom interactions, the quantity of those interactions for that isoform were fewer in number than the quantity of atom-atom interactions observed in the Aβ40 isoform. These results are summarized in Table 1 for Aβ40 compared against Aβ42.

As can be seen, the magnitude of both Coulombic and Lennard-Jones interactions are statistically distinct between the two isoforms according to a 95% confidence interval analysis (i.e. the intervals do not overlap) thus showing that the two strains are energetically different from each other in their inter-chain interaction profiles. Coulombic type interactions were further found to dominate as the primary force for either fibril which stabilizes both strains’ infrastructure (Coulombic force interactions being up to 3 to 4 times greater in magnitude than Lennard-Jones interactions). As is widely recognized in the literature, hydrogen bonds were observed to be the greatest constituent contributor to the Coulombic interactions^29^,^30^ holding the 1:2 chain configuration together for both Aβ40 and Aβ42 and they were observed to be primarily from backbone carbonyl oxygens and amino hydrogens from the same residues in both strains as discussed in more detail below.

Looking at the longer-range 1:3 interactions, we found that the Aβ42 isoform likewise exhibited stronger overall interactions and smaller atom-atom separation distances than Aβ40. Unlike the 1:2 interactions, however, all of these 1:3 interactions were exclusively composed of Coulombic atom-atom interactions. No Lennard-Jones interactions were observed for the 1:3 configuration in either fibril. This data is summarized in Table 2.

Like the 1:2 interactions, the observed average energies were statistically different for each isoform. Unlike the 1:2 interactions, however, the stronger bound Aβ42 has a superior number of strong atom-atom interactions in addition to a superior average energy per interaction pair for those interactions. An additional noteworthy distinction is that the 1:3 non-vicinal interactions for both strains are not stabilized by hydrogen bonds, but rather carbonyl carbon atoms interacting with carbonyl oxygen atoms serve as the main long-range inter-chain-stabilizing interacting atoms. This is in contrast to the hydrogen bond-rich scenario that marks the 1:2 interactions. No dominant interactions beyond 1:3 interactions (1:4 and up) were observed for either strain. A complete listing of the 1:2 configuration atom-atom pair interaction data is provided as Supplementary Table 1a,b (Aβ40 Coulombic and Lennard-Jones interactions respectively) and Supplementary Table 2a,b (Aβ42 Coulombic and Lennard-Jones interactions respectively). 1:3 interaction data are in Supplementary Tables 3 and 4.

**Figure 1:**
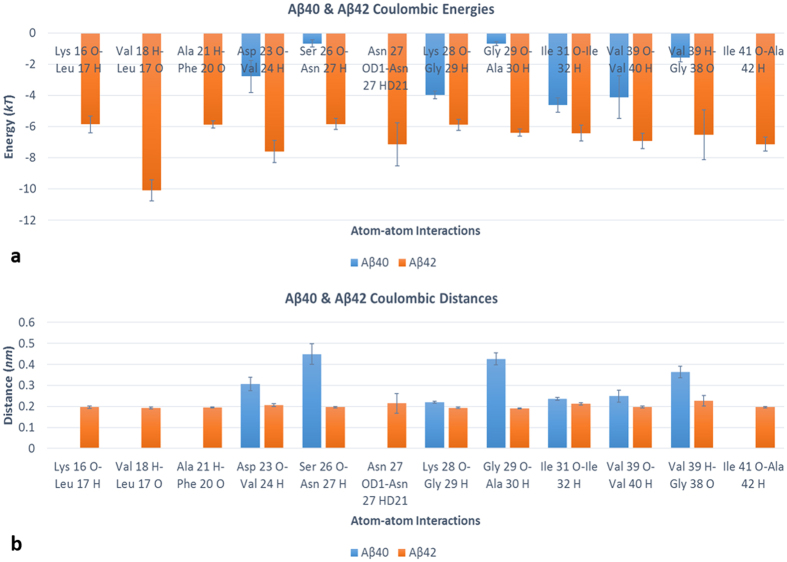
(**a,b**) Atom-atom interactions imparting exceptionally strong hydrogen bonding energies in the 1:2 configuration of Aβ42 compared to Aβ40 (**a**) and their respective atom-atom interaction distances (**b**) 95% confidence interval error bars included for analysis across all ensemble members. The first three, sixth, and last interactions were not observed in Aβ40. Interaction partners are presented as the residue, residue number in the sequence and the residue’s atom of one chain (chain A for both strains) interacting with its partner atom in the 1:2 configuration (on the D-chain in Aβ40 or on the B-chain in Aβ42).

Due to the fact that the dominant atom-atom interactions involved nearly identical residues in both Aβ40 and Aβ42 and that the overall average atom-atom distances were smaller in Aβ42 (Tables 1 and 2), we postulated that the differences observed for the stronger Aβ42 were due to a reduced distance between a smaller set of key interacting atoms in that isoform. Statistical data in the form of 95% confidence intervals for the average distance of the mapping results for both isoforms indeed revealed that the distances were statistically distinct between the two strains for both 1:2 and 1:3 interactions (see Tables 1 and 2). This motivated us to identify those atom-atom interactions, and their corresponding residues, that were primarily responsible for the changes in interacting energies that marked Aβ42’s superior aggregation interaction stabilities. We note that the strongest 1:2 interaction inter-atomic hydrogen bonds of Aβ40 had energy potential values within −4 to −5 *kT* whereas Aβ42’s hydrogen bond energy values exhibited a range between −6 to −10 *kT* for these vicinal chain energy mappings (mapping results for each isoform in Supplementary Table 1a,b and Supplementary Tables 2a,b). From these results of the mapping analysis, we identified 12 atom-atom interaction pairs spanning residues between Lys 16 to Ala 42 that imparted the aforementioned exceptionally strong hydrogen bonds observed throughout the 1:2 chain configuration for Aβ42. These residues were identified as those engaging in the exceptionally strong atom-atom interaction pairs that were found in the −6 to −10 *kT* range for Aβ42 which marked that isoform’s unique interaction energy profile. Seven of those 12 interactions involved the same atom-atom pairs in the Aβ40 strain, but their magnitudes in Aβ40 were noticeably weaker. These residues’ interactions, their average Coulombic energies and average inter-atomic distances are shown in Fig. 1a,b.

Those interaction energies which were observed in both isoforms were seen to be statistically distinct and considerably weaker in the Aβ40 strain (Fig. 1a) owing to larger inter-atomic distances (Fig. 1b). Interestingly, Aβ42 inter-atomic distances for these exceptionally strong interactions were appreciably uniform unlike those of Aβ40 which showed greater variability. Lastly, we note that for 1:3 interactions, although inter-atomic distances were statistically smaller in Aβ42, atom-atom interaction energy values resided within the same range for both strains (Aβ40 minimum and maximum of −0.3 *kT* and −0.4 *kT* respectively vs. Aβ42 minimum and maximum of −0.3 *kT* and −0.5 *kT* respectively) indicating that Aβ42’s superior 1:3 interaction strength is owed to a greater number of uniform interactions instead of any single interactions of exceptional energy as was seen in the 1:2 configuration (1:3 interaction mapping results for Aβ40 are in Supplementary Table 3 and 1:3 interaction mapping data for Aβ42 are in Supplementary Table 4).

In light of these findings, we next sought to identify the detailed atomic configurational reasons for the reduced inter-atomic distances of the homologous residues and associated atom-atom interactions seen in the 1:2 configuration’s exceptionally strong interactions; it was natural, therefore, to investigate the Ramachandran angle (φ and ψ) differences. The seven exceptionally strong atom-atom interaction pairs that were in both Aβ40 and Aβ42 were found to have φ and ψ angles that oriented the peptide planes more vertically in the Aβ42 isoform which consequently placed the backbone carbonyl oxygen and amino hydrogen atoms closer to each other. Overall, two particular observations are of note for the observed Ramachandran angles: 1) the Aβ42 strain shows more well-defined secondary structure primarily favoring β-sheet or left-handed α-helix regions on the Ramachandran plot and 2) the spread of φ and ψ angle values tends to be significantly reduced in Aβ42. A summary of these more well-defined secondary structure motifs acquired in Aβ42’s seven exceptionally strong interactions are shown in Table 3.

Additionally, the φ and ψ values proved to be statistically unique between the two isoforms thus illustrating distinctiveness between the two structures (complete φ and ψ angle data is in Supplementary Table 5). Ramachandran angle changes for three representative interactions are depicted in the figures for the following discussion on these results. The remaining interaction illustrations and Ramachandran angle data are found in Supplementary Fig. 1a,b through Supplementary Fig. 8a–d.

**Figure 2:**
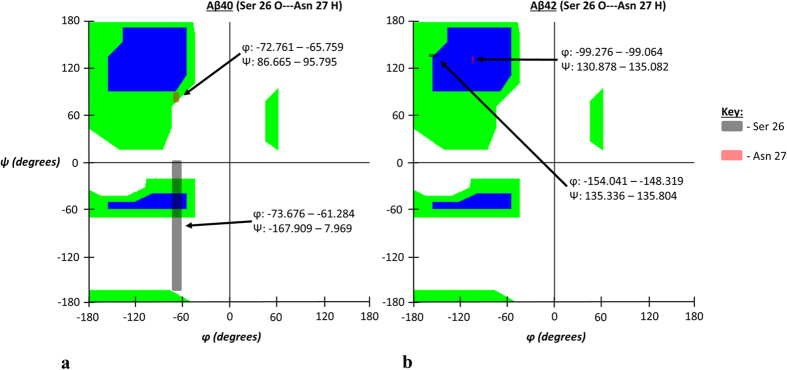
(**a,b**) Ramachandran angle profiles for an exceptionally strong atom-atom interaction (Ser 26 O interacting with Asn 27 H) for Aβ40 (**a**) and Aβ42 (**b**). Ranges for φ and ψ correspond to data spread according to 95% confidence interval analysis for all ensemble members as previously described. As stated before, the first atom is from the A chain of both isoforms and the second corresponds to the partner atom on the appropriate 1:2 interaction chain configuration. Note noticeable increase in β-sheet Ramachandran angle values for both residues of Aβ42.

**Figure 3:**
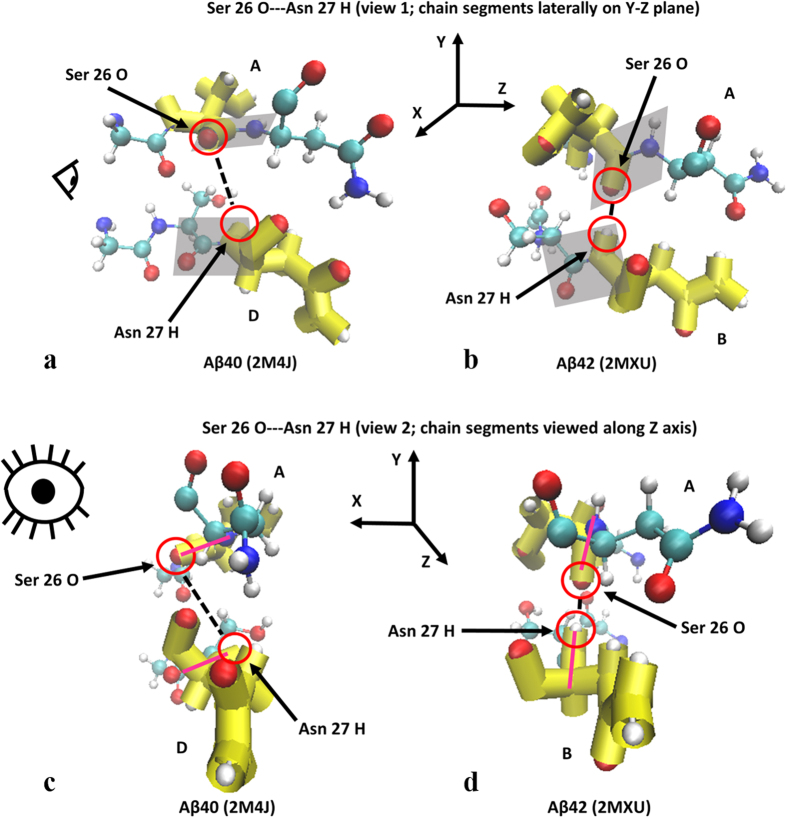
(**a–d**) Molecule representations of peptide plane alignment for Aβ40 (**a,c**) and Aβ42 (**b,d**). Shaded parallelograms in (**a**,**b**) are the peptide planes for the residues whose atoms are participating in the hydrogen bonding. (**c**,**d**) correspond to a view down the peptide bonds showing the peptide plane profile orientation in magenta. Eye icons indicate view perspective.

This particular interaction shows the Ramachandran angle values for the Aβ42 isoform to clearly reside in β-sheet territory compared to Aβ40 thus showing the increased secondary structure characteristics of Aβ42 that favor the β-sheet motif for both interacting residues. The resulting changes in peptide plane orientations for the interaction shown in Fig. 2a,b are further depicted in Fig. 3a–d for different viewing perspectives.

As can be seen in Fig. 3, the carbonyl oxygen and amino hydrogen that are involved in the hydrogen bond are considerably closer to each other in the Aβ42 isoform thus illustrating that the cause of reduced inter-atomic distances is indeed a vertically-oriented peptide plane. Two additional interactions are presented below that further illustrate this behavior in Ramachandran angles and their effects on peptide plane orientations.

**Figure 4:**
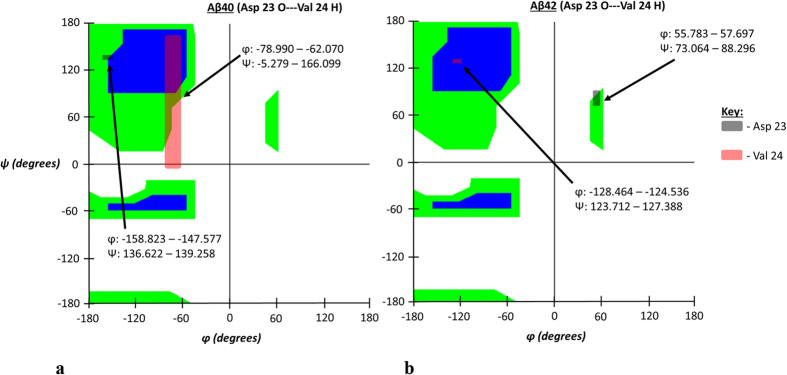
(**a,b**) Ramachandran angle profiles for an exceptionally strong atom-atom interaction (Asp 23 O interacting with Val 24 H) for Aβ40 (**a**) and Aβ42 (**b**). Ranges for φ and ψ correspond to data spread according to 95% confidence interval analysis for all ensemble members as previously described. As stated before, the first atom is from the A chain of both isoforms and the second corresponds to the partner atom on the appropriate 1:2 interaction chain configuration. Note noticeable increase in left-handed α-helix and β-sheet Ramachandran angle values for Asp 23 and Val 24 respectively in the Aβ42 isoform.

**Figure 5:**
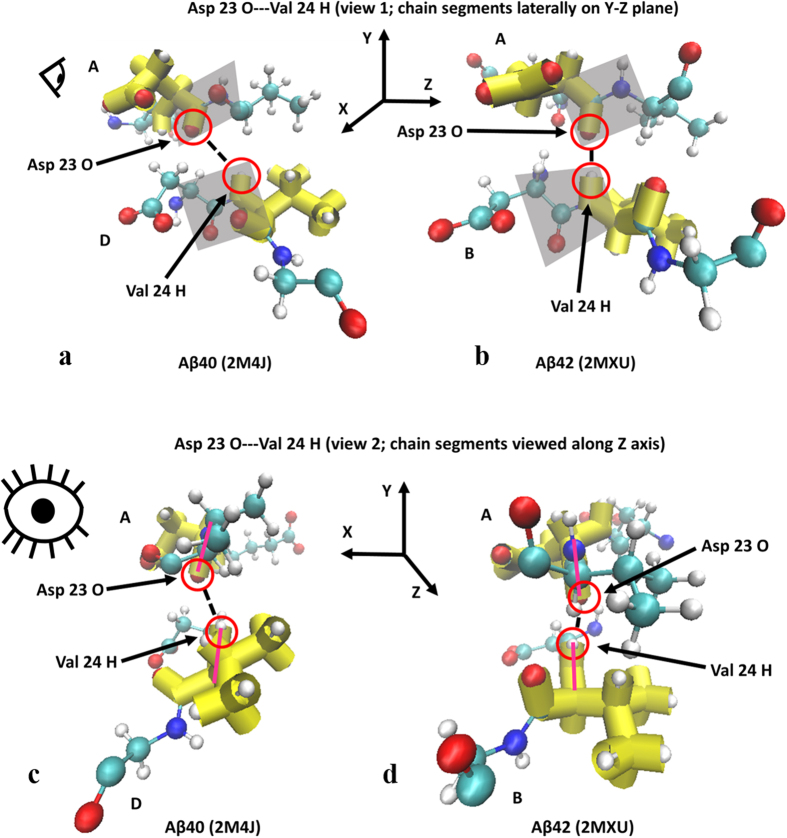
(**a–d**) Molecule representations of peptide plane alignment for Aβ40 (**a,c**) and Aβ42 (**b,d**). Shaded parallelograms in (**a**,**b**) are the peptide planes for the residues whose atoms are participating in the hydrogen bonding. (**c**,**d**) correspond to a view down the peptide bonds showing the peptide plane profile orientation in magenta. Eye icons indicate view perspective.

Like the first atom-atom interaction case shown in Fig. 2a,b, the secondary structure of the interaction pair illustrated in Fig. 4a,b shows much more defined secondary structure in the Aβ42 strain compared to Aβ40. Unlike the preceding case, however, each residue takes on a different secondary structure motif, either left-handed α-helix (Asp 23) or β-sheet (Val 24) as summarized in Table 3. Such differing structure motifs are interesting given the proximal nature of these two residues relative to the primary sequence. Despite the differences in secondary structure characteristics assumed by the interacting residues of Aβ42 for this interaction, the peptide planes of the participant atoms were likewise more vertical in the Aβ42 isoform as was also seen in the preceding case. This orientation likewise contributed to decreased atom-atom interaction distances and therefore results in the stronger hydrogen bonding observed in that isoform. Images for these peptide plane configurations are shown in Fig. 5a–d.

Although the atoms involved in this example did not show as exaggerated vertical orientation of the peptide planes in the Aβ42 strain, inter-atomic distance is nonetheless reduced in that isoform which corresponded to the observed superior interaction energy for this atom-atom pair interaction. For hydrogen bond Coulombic interactions, given the mathematical inverse relationship between the inter-atomic interacting energies and their inter-atomic distances, any appreciable increases in the interaction energy due to reduced distances, however small, are understandable and mathematically expected.

**Figure 6:**
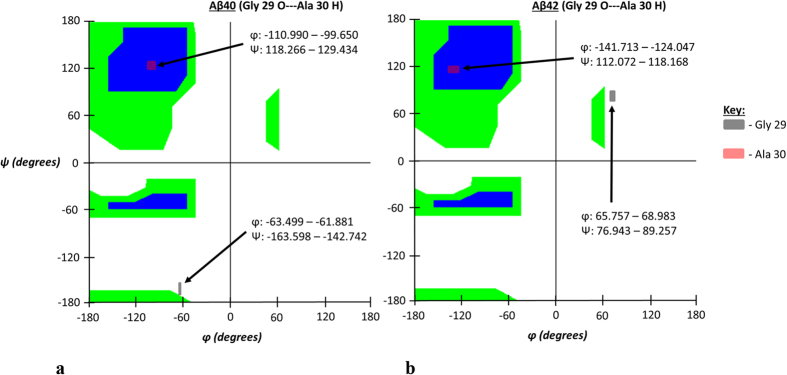
(**a,b**) Ramachandran angle profiles for an exceptionally strong atom-atom interaction (Gly 29 O interacting with Ala 30 H) for Aβ40 (**a**) and Aβ42 (**b**). Ranges for φ and ψ correspond to data spread according to 95% confidence interval analysis for all ensemble members as previously described. As stated before, the first atom is from the A chain of both isoforms and the second corresponds to the partner atom on the appropriate 1:2 interaction chain configuration. Note left-handed α-helix Ramachandran angle values for Gly 29 and the retention of β-sheet Ramachandran angle values for Ala 30 in Aβ42 compared to Aβ40.

We lastly present a final case where secondary structure was more well-defined in the Aβ42 isoform but for only one residue as opposed to both as has been seen in the previous two cases. Ramachandran plots for the respective angles of both Aβ40 and Aβ42 are shown in Fig. 6a,b.

**Figure 7:**
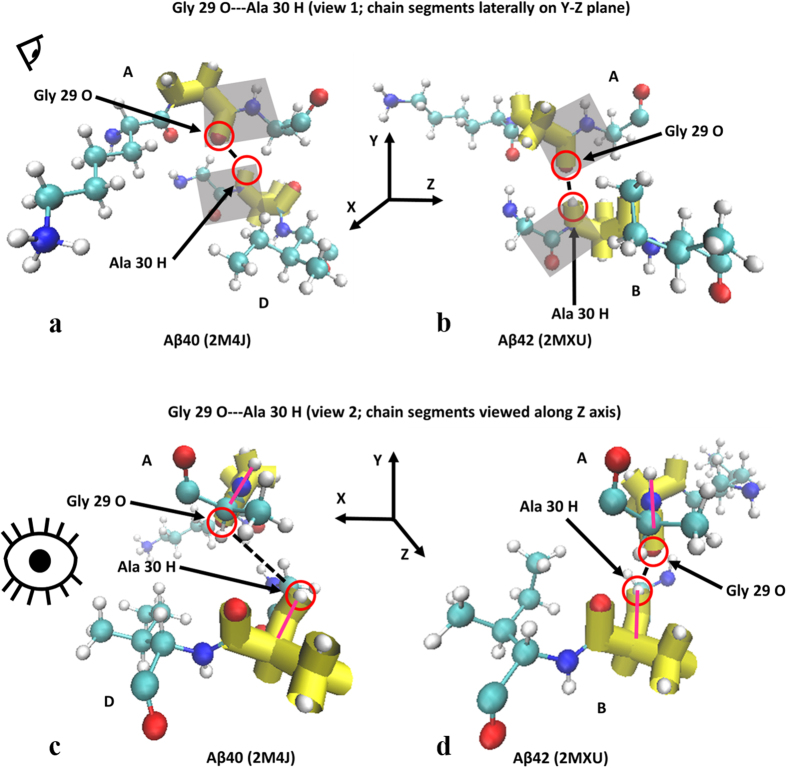
(**a–d**) Molecule representations of peptide plane alignment for Aβ40 (**a,c**) and Aβ42 (**b,d**). Shaded parallelograms in (**a**,**b**) are the peptide planes for the residues whose atoms are participating in the hydrogen bonding. (**c**,**d**) correspond to a view down the peptide bonds showing the peptide plane profile orientation in magenta. Eye icons indicate view perspective.

In spite of the variation for the types of secondary characteristics acquired in the Aβ42 isoform observed from case to case, the main motif of Aβ42 possessing more vertical peptide planes (and hence reduced inter-atomic distances) remained true to this case as well as can be seen in Fig. 7a–d.

Although specific attributes regarding acquired secondary characteristics in the Aβ42 strain are observed to vary from different interaction cases, the increased inter-chain interaction strength and stability of the Aβ42 fibrils, compared to Aβ40 fibrils, can confidently be attributed to Ramachandran angular changes that favor atom orientations that reduce key atom-atom interaction distances. These reduced distances thus favor strong attractive atom-atom interactions (particularly hydrogen bonding) between neighboring chains which appear to result in the superior aggregation stabilities and propensities observed in the Aβ42 fibril isoform.

Recently, two additional structures of Aβ42 aggregates have been published (PDB ID: 2NAO by Walti *et al.*^40^ and 5KK3 by Colvin *et al.*^41^) that allow more comprehensive inter-chain interaction comparisons between Aβ42 and Aβ40 across independently published structure files. As described below, nearly identical conformational attributes noted above for the Aβ42 structure by Xiao *et al.*^32^ (PDB ID: 2MXU) also occur for these two newly available structure files. Both 2NAO and 5KK3 were comprised of two stacks, but each structure’s energy mappings were virtually identical for both stacks so representative data for one stack (the A-C and A-I stacks for 2NAO and 5KK3 respectively) is provided below.

In the case of 2NAO (for the A-B chain interactions) by Walti *et al.*^40^, we found that the average energy per atom-atom interaction pair and the average distance, likewise per atom-atom interaction pair, were −0.828 ± 0.010 *kT* and 0.558 ± 0.002 *nm* respectively. This compares as appreciably similar to the results we obtained for 2MXU’s average inter-atomic energy and distance of −0.819 ± 0.008 *kT* and 0.568 ± 0.001 *nm* respectively. This comparative analysis between these two Aβ42 structures also led to our observing similar results for 2NAO concerning that structure’s β-sheet characteristics likewise reported by Walti *et al.*^40^. Those residues we identified as contributing to the strongest inter-chain hydrogen bond energies for 2NAO (which also possessed more well-defined secondary structure characteristics through our Ramachandran plot analysis), were also identified as key β-sheet residues by Walti *et al.*’s^40^ experimental chemical shift data. A complete comparison between 2MXU and 2NAO following our above analysis for the 2MXU structure can be found in the Supplemental Information.

In the case of 5KK3 (for the A-B chain interactions) by Colvin *et al.*^41^, concurrently, we found that the results were likewise similar to the 2MXU structure. Average energy and distance data per atom-atom interaction pair for 5KK3 were −0.889 ± 0.009 *kT* and 0.544 ± 0.002 *nm* respectively compared to 2MXU’s -average energy and distance data of 0.819 ± 0.008 *kT* and 0.568 ± 0.001 *nm* respectively. As with the study by Walti *et al.*^40^ for 2NAO, Colvin *et al.*^41^ also determined β-sheet structure regions using chemical shift data for their Aβ42 fibril structure. Our energetic analysis likewise yielded the identification of the same key residues involved in β-sheet structure as Colvin *et al.*’s^41^ chemical shift data. The full analysis data for our comparison of 5KK3 and 2MXU is found in the Supplemental Information.

## Discussion

Aβ42 is known to engage in persistent aggregation structures more readily than Aβ40, but until now, the details of why this is the case have not been clear. Our studies have indicated, however, that the underlying reason behind increased aggregation attractive interactions and their corresponding superior attractive energies in mature Aβ42 fibrils is due to a more vertical orientation of peptide planes within each constituent chain that places backbone carbonyl oxygens and amino hydrogens in closer proximity to each other as evidenced by Ramachandran angle data. This consequently allows distance-dependent non-covalent attractive interaction energies in the form of hydrogen bonds to flourish in Aβ42 which results in that isoform’s stronger inter-chain interactions compared to the weaker Aβ40 strain. Additionally, Ramachandran angles of Aβ42 peptide chains in the fibril structure indicate that Aβ42 has more well-defined secondary structure characteristics (specifically, β-sheet and left-handed α-helix) compared to Aβ40. Indeed, the importance of Ramachandran angle changes have even been observed in the study of aggregation transition from pre-fibrillar aggregate structures to mature fibrils^42^,^43^.

A natural follow-up inquiry to these observations would then seek to probe the reason(s) why the Aβ42 strain adopts more vertical peptide plane configurations. We postulate that, given Aβ42’s additional two C-terminal residues (Ile 41 and Ala 42), individual Aβ42 peptide chains are perhaps able to sample fold-like configurations in the early aggregation process that allow Ramachandran angles which permit the more vertical peptide planes that favor stronger hydrogen bonding attractive interaction energies. Given Aβ40’s lack of these two C-terminal residues, it, by contrast, may perhaps not favorably sample those same conformations that favor reduced inter-atomic distances. Indeed, Urbanc *et al.*^44^ discerned differences in the conformations assumed by monomer peptides of Aβ40 and Aβ42 during their computational simulation of the actual aggregation process for both isoforms. Such differences between isoforms observed during aggregation growth may yet be observed in the range of individual monomer motion preceding the actual aggregation process when considering the potential effects on individual monomer movement imparted by the last two C-terminal residues in Aβ42. Ultimately, therefore, the phenomenon behind Aβ42’s superior attractive interactions and superior stability, may be traced to concepts in the protein folding problem as they pertain to the three-dimensional configurations both isoforms’ monomers distinctively sample prior to the early aggregation/oligomer stages leading up to mature fibril formation.

## Additional Information

**How to cite this article**: Bastidas, O. H. *et al.* Few Ramachandran Angle Changes Provide Interaction Strength Increase in Aβ42 versus Aβ40 Amyloid Fibrils. *Sci. Rep.* **6**, 36499; doi: 10.1038/srep36499 (2016).

**Publisher’s note:** Springer Nature remains neutral with regard to jurisdictional claims in published maps and institutional affiliations.

## Supplementary Material

Supplementary Information

## Figures and Tables

**Table 1:** Comparison of the quantity of the number of dominant atom-atom interaction pairs, average energy (per interaction pair) and average inter-atomic distance (per interaction pair) of 1:2 atom-atom interactions between two chains for Aβ40 (A-D chain mapping results) and Aβ42 (A-B chain mapping results) with 95% confidence intervals for analysis across all ensemble members.

	Aβ40 (A-D)	Aβ42 (A-B)
Coulombic Interactions:	811	779
Coulombic Average E (*kT*):	−0.729 ± 0.006	−0.819 ± 0.008
Coulombic Average D (nm):	0.575 ± 0.001	0.568 ± 0.001
Lennard-Jones Interactions:	135	118
Lennard-Jones Average E (*kT*):	−0.177 ± 0.001	−0.188 ± 0.002
Lennard-Jones Average D (nm)	0.397 ± 0.001	0.393 ± 0.001

Energy units are in *kT* and distance units are in nanometers.

**Table 2:** Comparison of the quantity of the number of dominant atom-atom interaction pairs, average energy (per interaction pair) and average inter-atomic distance (per interaction pair) of 1:3 atom-atom interactions between two chains for Aβ40 (A-G chain mapping results) and Aβ42 (A-C chain mapping results) with 95% confidence intervals for analysis across all ensemble members.

	Aβ40 (A-G)	Aβ42 (A-C)
Coulombic Interactions:	19	36
Coulombic Average E (*kT*):	−0.352 ± 0.004	−0.398 ± 0.003
Coulombic Average D (nm):	0.892 ± 0.006	0.877 ± 0.004
Lennard-Jones Interactions:	0	0
Lennard-Jones Average E (*kT*):	0	0
Lennard-Jones Average D (nm)	0	0

Energy units are in *kT* and distance units are in nanometers.

**Table 3:** Secondary structure motifs that are more well-defined in Aβ42 compared to Aβ40.

Atom-atom Interaction Pairs		Secondary Structure Acquired in Aβ42 Interaction Pairs	
A Chain	Partner Chain: D (Aβ40) or B (Aβ42)	A Chain Residue (Aβ42)	B Chain Residue (Aβ42)
Ser 26 O	Asn 27 H	β-sheet increase	β-sheet increase
Asp 23 O	Val 24 H	Left-hand α-helix increase	β-sheet increase
Gly 29 O	Ala 30 H	Left-hand α-helix increase	No change
Lys 28 O	Gly 29 H	No change	Left-hand α-helix increase
Ile 31 O	Ile 32 H	β-sheet decrease	β-sheet increase
Val 39 O	Val 40 H	No change	β-sheet increase
Val 39 H	Gly 38 O	No change	β-sheet increase
