# Supplementary material for: Few Ramachandran Angle Changes Provide Interaction Strength Increase in Aβ42 versus Aβ40 Amyloid Fibrils
Source: Sci Rep. 2016 Nov 3;6:36499. doi: 10.1038/srep36499 (PMC5093553; doi:10.1038/srep36499)
Supplement: Supplementary Information [file srep36499-s1.pdf]

Title: Few Ramachandran Angle Changes Provide Interaction Strength Increase in Aβ42 versus Aβ40 Amyloid Fibrils

Authors: Oscar H. Bastidas, Benjamin Green, Mary Sprague, Michael H. Peters

Supplementary Table 1a: Mapping results for Aβ40's (PDB ID: 2M4J by Lu et al.) short range (1:2) dominant atom-atom Coulombic interactions across ensemble structures. Columns for each chain correspond to: residue abbreviation, residue number in peptide sequence, atom identity (IUPAC naming convention) and atom number in PDB file. Energy in *kT*, distance in *nm*. Mapping analysis began on the 11th residue for both isoforms because original structure data for Aβ42 begins with the 11th residue.

Chain A				Chain D				Average Distance	Average Coulombic Values	Lower 95% Confidence Interval Bound	Upper 95% Confidence Interval Bound	Margin of Error
GLU	11	N	152	GLU	11	C	1951	5.75E-01	-4.21E-01	-4.26E-01	-4.16E-01	5.02E-03
GLU	11	N	152	GLU	11	CD	1955	6.25E-01	-5.73E-01	-5.80E-01	-5.66E-01	6.71E-03
GLU	11	N	152	VAL	12	H	1971	5.03E-01	-5.36E-01	-5.45E-01	-5.27E-01	8.92E-03
GLU	11	C	154	GLU	11	N	1949	5.60E-01	-4.46E-01	-4.50E-01	-4.42E-01	4.16E-03
GLU	11	C	154	GLU	11	O	1952	6.27E-01	-4.90E-01	-4.93E-01	-4.87E-01	2.76E-03
GLU	11	C	154	GLU	11	OE1	1956	7.22E-01	-5.28E-01	-5.88E-01	-4.68E-01	5.97E-02
GLU	11	C	154	GLU	11	OE2	1957	7.04E-01	-5.52E-01	-6.05E-01	-4.99E-01	5.30E-02
GLU	11	C	154	VAL	12	N	1964	4.47E-01	-8.07E-01	-8.14E-01	-8.00E-01	6.98E-03
GLU	11	C	154	VAL	12	O	1967	4.55E-01	-6.99E-01	-7.05E-01	-6.92E-01	6.19E-03
GLU	11	C	154	HIS	13	N	1980	6.66E-01	-3.87E-01	-3.88E-01	-3.85E-01	1.71E-03
GLU	11	C	154	HIS	13	ND1	1986	6.26E-01	-3.51E-01	-3.54E-01	-3.49E-01	2.79E-03
GLU	11	O	155	GLU	11	C	1951	3.99E-01	-1.43E+00	-1.44E+00	-1.42E+00	1.46E-02
GLU	11	O	155	GLU	11	CD	1955	6.26E-01	-8.01E-01	-8.04E-01	-7.97E-01	3.47E-03
GLU	11	O	155	GLU	11	H	1958	5.55E-01	-4.17E-01	-4.21E-01	-4.12E-01	4.11E-03
GLU	11	O	155	GLU	11	HA	1959	2.73E-01	-5.68E-01	-5.81E-01	-5.56E-01	1.25E-02
GLU	11	O	155	VAL	12	C	1966	4.69E-01	-9.00E-01	-9.08E-01	-8.92E-01	8.08E-03
GLU	11	O	155	VAL	12	CB	1968	4.57E-01	-8.48E-01	-8.62E-01	-8.33E-01	1.41E-02
GLU	11	O	155	VAL	12	H	1971	2.72E-01	-3.86E+00	-3.92E+00	-3.79E+00	6.26E-02
GLU	11	O	155	HIS	13	C	1982	8.06E-01	-4.27E-01	-4.29E-01	-4.25E-01	1.79E-03
GLU	11	CD	158	GLU	11	N	1949	7.24E-01	-4.28E-01	-4.30E-01	-4.25E-01	2.95E-03
GLU	11	CD	158	GLU	11	O	1952	8.84E-01	-4.22E-01	-4.24E-01	-4.21E-01	1.46E-03
GLU	11	CD	158	GLU	11	OE1	1956	5.55E-01	-1.61E+00	-2.00E+00	-1.22E+00	3.89E-01
GLU	11	CD	158	GLU	11	OE2	1957	5.15E-01	-1.88E+00	-2.26E+00	-1.50E+00	3.78E-01
GLU	11	CD	158	VAL	12	N	1964	7.59E-01	-4.17E-01	-4.19E-01	-4.15E-01	1.83E-03
GLU	11	CD	158	VAL	12	O	1967	8.02E-01	-3.40E-01	-3.42E-01	-3.39E-01	1.40E-03
GLU	11	CD	158	HIS	13	N	1980	9.55E-01	-3.33E-01	-3.34E-01	-3.32E-01	8.98E-04
GLU	11	CD	158	HIS	13	ND1	1986	7.15E-01	-4.38E-01	-4.42E-01	-4.34E-01	3.97E-03
GLU	11	OE1	159	GLU	11	C	1951	7.82E-01	-4.51E-01	-4.88E-01	-4.15E-01	3.66E-02
GLU	11	OE1	159	GLU	11	CD	1955	4.89E-01	-2.18E+00	-2.67E+00	-1.68E+00	4.93E-01
GLU	11	OE1	159	VAL	12	C	1966	8.81E-01	-3.46E-01	-3.58E-01	-3.34E-01	1.23E-02
GLU	11	OE1	159	VAL	12	H	1971	6.93E-01	-5.26E-01	-5.62E-01	-4.89E-01	3.66E-02
GLU	11	OE1	159	GLY	38	C	2358	8.60E-01	-4.71E-01	-4.98E-01	-4.43E-01	2.75E-02

GLU	11	OE1	159	VAL	39	CB	2367	7.09E-01	-4.58E-01	-5.06E-01	-4.10E-01	4.78E-02
GLU	11	OE1	159	VAL	39	H	2370	8.52E-01	-3.68E-01	-4.08E-01	-3.29E-01	3.96E-02
GLU	11	OE2	160	GLU	11	C	1951	8.19E-01	-4.16E-01	-4.48E-01	-3.83E-01	3.25E-02
GLU	11	OE2	160	GLU	11	CD	1955	5.33E-01	-1.80E+00	-2.28E+00	-1.32E+00	4.80E-01
GLU	11	OE2	160	VAL	12	H	1971	7.24E-01	-4.83E-01	-5.12E-01	-4.53E-01	2.93E-02
GLU	11	OE2	160	GLY	38	C	2358	8.64E-01	-4.69E-01	-5.04E-01	-4.34E-01	3.50E-02
GLU	11	OE2	160	VAL	39	CB	2367	7.02E-01	-4.80E-01	-5.57E-01	-4.04E-01	7.65E-02
GLU	11	H	161	GLU	11	N	1949	4.36E-01	-5.31E-01	-5.36E-01	-5.25E-01	5.66E-03
GLU	11	H	161	GLU	11	O	1952	6.29E-01	-3.19E-01	-3.21E-01	-3.16E-01	2.23E-03
GLU	11	H	161	GLU	11	OE1	1956	6.11E-01	-4.78E-01	-5.22E-01	-4.33E-01	4.42E-02
GLU	11	H	161	GLU	11	OE2	1957	5.93E-01	-5.06E-01	-5.42E-01	-4.71E-01	3.56E-02
GLU	11	H	161	VAL	12	N	1964	5.39E-01	-3.39E-01	-3.42E-01	-3.35E-01	3.28E-03
VAL	12	N	167	GLU	11	C	1951	5.93E-01	-4.19E-01	-4.21E-01	-4.16E-01	2.41E-03
VAL	12	N	167	GLU	11	CD	1955	7.55E-01	-4.21E-01	-4.24E-01	-4.18E-01	2.94E-03
VAL	12	N	167	VAL	12	C	1966	5.40E-01	-4.90E-01	-4.92E-01	-4.87E-01	2.59E-03
VAL	12	N	167	VAL	12	CB	1968	5.89E-01	-3.57E-01	-3.59E-01	-3.55E-01	2.22E-03
VAL	12	N	167	VAL	12	H	1971	4.21E-01	-8.83E-01	-8.92E-01	-8.75E-01	8.46E-03
VAL	12	N	167	HIS	13	C	1982	8.12E-01	-3.20E-01	-3.21E-01	-3.19E-01	9.45E-04
VAL	12	C	169	VAL	12	N	1964	5.81E-01	-4.16E-01	-4.18E-01	-4.13E-01	2.45E-03
VAL	12	C	169	VAL	12	O	1967	3.82E-01	-1.03E+00	-1.04E+00	-1.03E+00	8.37E-03
VAL	12	C	169	HIS	13	N	1980	5.91E-01	-4.70E-01	-4.72E-01	-4.69E-01	1.58E-03
VAL	12	C	169	HIS	13	ND1	1986	5.36E-01	-4.69E-01	-4.73E-01	-4.65E-01	3.97E-03
VAL	12	C	169	HIS	14	N	1997	6.74E-01	-3.58E-01	-3.59E-01	-3.56E-01	1.70E-03
VAL	12	O	170	VAL	12	C	1966	6.29E-01	-3.17E-01	-3.18E-01	-3.15E-01	1.28E-03
VAL	12	O	170	VAL	12	H	1971	6.14E-01	-3.28E-01	-3.30E-01	-3.26E-01	2.00E-03
VAL	12	O	170	HIS	13	C	1982	7.83E-01	-3.07E-01	-3.07E-01	-3.06E-01	7.76E-04
VAL	12	CB	171	VAL	12	N	1964	5.40E-01	-4.33E-01	-4.36E-01	-4.30E-01	3.09E-03
VAL	12	CB	171	VAL	12	O	1967	4.25E-01	-6.94E-01	-6.98E-01	-6.89E-01	4.21E-03
VAL	12	CB	171	HIS	13	N	1980	6.40E-01	-3.52E-01	-3.53E-01	-3.50E-01	1.06E-03
VAL	12	H	174	GLU	11	OE1	1956	8.49E-01	-3.71E-01	-4.12E-01	-3.30E-01	4.08E-02
VAL	12	H	174	GLU	11	OE2	1957	8.25E-01	-3.87E-01	-4.22E-01	-3.52E-01	3.50E-02
VAL	12	H	174	VAL	12	N	1964	5.97E-01	-3.86E-01	-3.88E-01	-3.84E-01	1.85E-03
VAL	12	H	174	VAL	12	O	1967	5.11E-01	-4.94E-01	-4.97E-01	-4.92E-01	2.57E-03
VAL	12	H	174	HIS	13	ND1	1986	6.33E-01	-3.21E-01	-3.24E-01	-3.19E-01	2.56E-03
HIS	13	N	183	GLU	11	C	1951	7.00E-01	-3.50E-01	-3.51E-01	-3.48E-01	1.67E-03
HIS	13	N	183	GLU	11	CD	1955	9.00E-01	-3.66E-01	-3.67E-01	-3.64E-01	1.31E-03
HIS	13	N	183	VAL	12	C	1966	4.46E-01	-9.07E-01	-9.13E-01	-9.00E-01	6.52E-03
HIS	13	N	183	VAL	12	CB	1968	5.97E-01	-4.08E-01	-4.09E-01	-4.06E-01	1.94E-03
HIS	13	N	183	VAL	12	H	1971	5.04E-01	-6.65E-01	-6.70E-01	-6.59E-01	5.40E-03
HIS	13	N	183	HIS	13	C	1982	5.59E-01	-7.86E-01	-7.90E-01	-7.83E-01	3.39E-03
HIS	13	N	183	HIS	13	CG	1985	4.95E-01	-4.37E-01	-4.42E-01	-4.32E-01	5.19E-03
HIS	13	N	183	HIS	14	C	1999	6.97E-01	-4.97E-01	-5.00E-01	-4.93E-01	3.43E-03

HIS	13	N	183	HIS	14	H	2007	4.78E-01	-4.84E-01	-4.89E-01	-4.80E-01	4.70E-03
HIS	13	C	185	GLU	11	O	1952	9.66E-01	-3.18E-01	-3.18E-01	-3.17E-01	7.87E-04
HIS	13	C	185	VAL	12	N	1964	7.53E-01	-3.66E-01	-3.68E-01	-3.65E-01	1.37E-03
HIS	13	C	185	VAL	12	O	1967	4.73E-01	-8.94E-01	-9.01E-01	-8.86E-01	7.25E-03
HIS	13	C	185	HIS	13	N	1980	5.67E-01	-7.63E-01	-7.66E-01	-7.59E-01	3.86E-03
HIS	13	C	185	HIS	13	O	1983	6.25E-01	-6.20E-01	-6.22E-01	-6.18E-01	1.70E-03
HIS	13	C	185	HIS	13	ND1	1986	5.23E-01	-7.33E-01	-7.41E-01	-7.25E-01	7.88E-03
HIS	13	C	185	HIS	14	N	1997	4.34E-01	-1.44E+00	-1.45E+00	-1.43E+00	9.89E-03
HIS	13	C	185	HIS	14	O	2000	4.57E-01	-1.27E+00	-1.28E+00	-1.26E+00	1.27E-02
HIS	13	C	185	GLN	15	N	2014	5.81E-01	-5.30E-01	-5.38E-01	-5.22E-01	7.73E-03
HIS	13	C	185	GLN	15	O	2017	8.20E-01	-3.95E-01	-3.98E-01	-3.92E-01	3.27E-03
HIS	13	C	185	GLN	15	OE1	2021	8.07E-01	-4.86E-01	-5.74E-01	-3.99E-01	8.73E-02
HIS	13	C	185	GLN	15	NE2	2022	7.53E-01	-7.48E-01	-8.47E-01	-6.49E-01	9.89E-02
HIS	13	O	186	VAL	12	C	1966	4.78E-01	-7.65E-01	-7.71E-01	-7.59E-01	6.09E-03
HIS	13	O	186	VAL	12	CB	1968	6.56E-01	-3.35E-01	-3.37E-01	-3.34E-01	1.59E-03
HIS	13	O	186	VAL	12	H	1971	6.51E-01	-3.79E-01	-3.81E-01	-3.77E-01	2.11E-03
HIS	13	O	186	HIS	13	C	1982	3.84E-01	-1.97E+00	-1.99E+00	-1.96E+00	1.51E-02
HIS	13	O	186	HIS	13	CG	1985	5.01E-01	-4.27E-01	-4.33E-01	-4.22E-01	5.67E-03
HIS	13	O	186	HIS	13	H	1990	5.52E-01	-3.46E-01	-3.48E-01	-3.44E-01	1.83E-03
HIS	13	O	186	HIS	13	HA	1991	2.66E-01	-7.07E-01	-7.19E-01	-6.94E-01	1.29E-02
HIS	13	O	186	HIS	14	C	1999	4.19E-01	-1.58E+00	-1.61E+00	-1.55E+00	2.69E-02
HIS	13	O	186	HIS	14	H	2007	2.34E-01	-3.43E+00	-3.53E+00	-3.33E+00	9.58E-02
HIS	13	O	186	GLN	15	CD	2020	7.19E-01	-4.95E-01	-5.72E-01	-4.19E-01	7.67E-02
HIS	13	O	186	GLN	15	H	2023	5.59E-01	-3.59E-01	-3.64E-01	-3.53E-01	5.74E-03
HIS	13	CG	188	VAL	12	O	1967	5.13E-01	-3.08E-01	-3.13E-01	-3.04E-01	4.43E-03
HIS	13	CG	188	HIS	13	ND1	1986	3.96E-01	-6.12E-01	-6.18E-01	-6.06E-01	6.37E-03
HIS	13	CD2	190	VAL	12	H	1971	5.32E-01	-3.31E-01	-3.36E-01	-3.27E-01	4.38E-03
HIS	13	CD2	190	HIS	13	HE1	1995	2.91E-01	-4.62E-01	-4.66E-01	-4.58E-01	4.07E-03
HIS	13	H	193	VAL	12	N	1964	4.95E-01	-3.78E-01	-3.81E-01	-3.75E-01	2.93E-03
HIS	13	H	193	VAL	12	O	1967	2.35E-01	-2.61E+00	-2.66E+00	-2.57E+00	4.43E-02
HIS	13	H	193	HIS	13	N	1980	4.09E-01	-7.14E-01	-7.19E-01	-7.10E-01	4.88E-03
HIS	13	H	193	HIS	13	O	1983	5.86E-01	-3.03E-01	-3.05E-01	-3.02E-01	1.25E-03
HIS	13	H	193	HIS	13	ND1	1986	3.99E-01	-6.10E-01	-6.20E-01	-6.00E-01	1.02E-02
HIS	13	H	193	HIS	14	N	1997	4.82E-01	-4.73E-01	-4.77E-01	-4.69E-01	4.05E-03
HIS	13	HD2	197	VAL	12	O	1967	3.74E-01	-3.93E-01	-4.03E-01	-3.83E-01	1.02E-02
HIS	13	HD2	197	HIS	13	ND1	1986	3.11E-01	-6.84E-01	-6.93E-01	-6.74E-01	9.49E-03
HIS	14	N	200	VAL	12	C	1966	6.93E-01	-3.39E-01	-3.41E-01	-3.38E-01	1.57E-03
HIS	14	N	200	HIS	13	C	1982	5.96E-01	-6.85E-01	-6.87E-01	-6.82E-01	2.47E-03
HIS	14	N	200	HIS	14	C	1999	5.09E-01	-9.79E-01	-9.89E-01	-9.69E-01	9.95E-03
HIS	14	N	200	HIS	14	H	2007	4.14E-01	-6.92E-01	-6.97E-01	-6.87E-01	4.83E-03
HIS	14	N	200	GLN	15	CD	2020	7.29E-01	-4.93E-01	-5.87E-01	-3.99E-01	9.42E-02
HIS	14	C	202	HIS	13	N	1980	8.84E-01	-3.25E-01	-3.26E-01	-3.24E-01	1.00E-03

HIS	14	C	202	HIS	13	O	1983	8.38E-01	-3.56E-01	-3.57E-01	-3.55E-01	9.89E-04
HIS	14	C	202	HIS	14	N	1997	6.13E-01	-6.45E-01	-6.48E-01	-6.42E-01	3.11E-03
HIS	14	C	202	HIS	14	O	2000	4.13E-01	-1.64E+00	-1.65E+00	-1.63E+00	1.12E-02
HIS	14	C	202	GLN	15	N	2014	5.48E-01	-6.03E-01	-6.10E-01	-5.96E-01	6.62E-03
HIS	14	C	202	GLN	15	O	2017	6.98E-01	-5.30E-01	-5.37E-01	-5.24E-01	6.75E-03
HIS	14	C	202	GLN	15	OE1	2021	7.53E-01	-5.78E-01	-7.20E-01	-4.36E-01	1.42E-01
HIS	14	C	202	GLN	15	NE2	2022	7.32E-01	-7.70E-01	-8.33E-01	-7.07E-01	6.29E-02
HIS	14	C	202	LYS	16	N	2031	6.29E-01	-5.05E-01	-5.14E-01	-4.95E-01	9.57E-03
HIS	14	C	202	LYS	16	O	2034	7.73E-01	-4.37E-01	-4.45E-01	-4.29E-01	8.28E-03
HIS	14	O	203	HIS	13	C	1982	8.32E-01	-3.60E-01	-3.61E-01	-3.59E-01	1.09E-03
HIS	14	O	203	HIS	14	C	1999	6.10E-01	-6.53E-01	-6.56E-01	-6.49E-01	3.71E-03
HIS	14	O	203	GLN	15	CD	2020	8.14E-01	-3.92E-01	-4.47E-01	-3.38E-01	5.42E-02
HIS	14	O	203	LYS	16	C	2033	8.68E-01	-3.68E-01	-3.74E-01	-3.62E-01	5.77E-03
HIS	14	CB	204	HIS	14	C	1999	4.44E-01	-3.93E-01	-4.03E-01	-3.82E-01	1.00E-02
HIS	14	CG	205	HIS	14	N	1997	5.19E-01	-3.92E-01	-3.95E-01	-3.88E-01	3.88E-03
HIS	14	CG	205	HIS	14	O	2000	2.88E-01	-1.85E+00	-1.89E+00	-1.80E+00	4.49E-02
HIS	14	ND1	206	HIS	13	C	1982	6.07E-01	-5.33E-01	-5.71E-01	-4.96E-01	3.76E-02
HIS	14	ND1	206	HIS	14	C	1999	3.75E-01	-1.69E+00	-1.74E+00	-1.65E+00	4.35E-02
HIS	14	ND1	206	HIS	14	CG	2002	4.16E-01	-5.69E-01	-6.51E-01	-4.88E-01	8.18E-02
HIS	14	ND1	206	HIS	14	H	2007	4.77E-01	-3.92E-01	-4.13E-01	-3.71E-01	2.07E-02
HIS	14	ND1	206	GLN	15	C	2016	5.48E-01	-4.33E-01	-4.99E-01	-3.67E-01	6.59E-02
HIS	14	CD2	207	HIS	14	C	1999	4.61E-01	-6.98E-01	-7.15E-01	-6.82E-01	1.67E-02
HIS	14	H	210	HIS	14	O	2000	5.28E-01	-3.83E-01	-3.86E-01	-3.81E-01	2.32E-03
HIS	14	HA	211	HIS	14	O	2000	2.45E-01	-9.01E-01	-9.09E-01	-8.94E-01	7.22E-03
HIS	14	HD2	214	HIS	14	O	2000	4.13E-01	-3.96E-01	-4.05E-01	-3.87E-01	9.06E-03
HIS	14	HE1	215	HIS	14	O	2000	3.70E-01	-4.21E-01	-4.34E-01	-4.09E-01	1.23E-02
GLN	15	N	217	HIS	13	C	1982	7.57E-01	-3.12E-01	-3.14E-01	-3.10E-01	2.07E-03
GLN	15	N	217	HIS	14	C	1999	5.00E-01	-7.46E-01	-7.53E-01	-7.40E-01	6.51E-03
GLN	15	N	217	GLN	15	C	2016	5.40E-01	-3.95E-01	-4.01E-01	-3.89E-01	5.77E-03
GLN	15	N	217	GLN	15	CD	2020	5.98E-01	-5.54E-01	-6.87E-01	-4.21E-01	1.33E-01
GLN	15	N	217	GLN	15	HA	2024	3.41E-01	-4.59E-01	-4.69E-01	-4.50E-01	9.68E-03
GLN	15	N	217	LYS	16	C	2033	7.01E-01	-3.95E-01	-4.02E-01	-3.87E-01	7.68E-03
GLN	15	C	219	HIS	14	O	2000	5.40E-01	-5.40E-01	-5.50E-01	-5.29E-01	1.05E-02
GLN	15	C	219	GLN	15	N	2014	5.83E-01	-3.33E-01	-3.36E-01	-3.29E-01	3.24E-03
GLN	15	C	219	GLN	15	O	2017	6.20E-01	-4.27E-01	-4.29E-01	-4.24E-01	2.58E-03
GLN	15	C	219	GLN	15	NE2	2022	6.84E-01	-5.49E-01	-5.77E-01	-5.21E-01	2.82E-02
GLN	15	C	219	LYS	16	N	2031	4.54E-01	-6.72E-01	-6.83E-01	-6.62E-01	1.06E-02
GLN	15	C	219	LYS	16	O	2034	4.82E-01	-7.53E-01	-7.80E-01	-7.25E-01	2.73E-02
GLN	15	O	220	HIS	13	C	1982	8.58E-01	-3.66E-01	-3.69E-01	-3.63E-01	2.64E-03
GLN	15	O	220	HIS	14	C	1999	5.15E-01	-1.01E+00	-1.03E+00	-9.97E-01	1.77E-02
GLN	15	O	220	GLN	15	C	2016	4.04E-01	-1.18E+00	-1.20E+00	-1.15E+00	2.47E-02
GLN	15	O	220	GLN	15	CD	2020	6.15E-01	-7.09E-01	-7.90E-01	-6.28E-01	8.09E-02

GLN	15	O	220	GLN	15	H	2023	5.77E-01	-3.58E-01	-3.65E-01	-3.51E-01	7.01E-03
GLN	15	O	220	GLN	15	HA	2024	2.80E-01	-1.17E+00	-1.23E+00	-1.11E+00	5.89E-02
GLN	15	O	220	LYS	16	C	2033	4.79E-01	-1.33E+00	-1.37E+00	-1.28E+00	4.65E-02
GLN	15	O	220	LYS	16	H	2040	3.01E-01	-1.59E+00	-1.73E+00	-1.45E+00	1.36E-01
GLN	15	O	220	LEU	17	C	2055	8.04E-01	-3.54E-01	-3.58E-01	-3.50E-01	3.98E-03
GLN	15	CD	223	HIS	14	N	1997	8.80E-01	-3.32E-01	-3.45E-01	-3.20E-01	1.25E-02
GLN	15	CD	223	HIS	14	O	2000	7.59E-01	-4.32E-01	-4.56E-01	-4.07E-01	2.45E-02
GLN	15	CD	223	GLN	15	O	2017	8.40E-01	-3.92E-01	-4.29E-01	-3.54E-01	3.77E-02
GLN	15	CD	223	GLN	15	OE1	2021	5.12E-01	-1.31E+00	-1.64E+00	-9.75E-01	3.34E-01
GLN	15	CD	223	GLN	15	NE2	2022	5.00E-01	-1.94E+00	-2.43E+00	-1.46E+00	4.82E-01
GLN	15	CD	223	LYS	16	N	2031	6.87E-01	-4.49E-01	-5.14E-01	-3.85E-01	6.44E-02
GLN	15	CD	223	LYS	16	O	2034	7.42E-01	-4.97E-01	-5.62E-01	-4.32E-01	6.49E-02
GLN	15	OE1	224	HIS	14	C	1999	8.36E-01	-4.37E-01	-4.84E-01	-3.90E-01	4.72E-02
GLN	15	OE1	224	GLN	15	CD	2020	5.25E-01	-1.21E+00	-1.48E+00	-9.34E-01	2.73E-01
GLN	15	OE1	224	GLN	15	HE22	2030	5.10E-01	-1.17E+00	-1.75E+00	-5.84E-01	5.82E-01
GLN	15	OE1	224	LYS	16	C	2033	7.77E-01	-5.79E-01	-6.89E-01	-4.69E-01	1.10E-01
GLN	15	NE2	225	HIS	14	C	1999	8.28E-01	-6.08E-01	-6.24E-01	-5.92E-01	1.59E-02
GLN	15	NE2	225	GLN	15	C	2016	7.86E-01	-4.26E-01	-4.58E-01	-3.93E-01	3.26E-02
GLN	15	NE2	225	GLN	15	CD	2020	5.39E-01	-1.60E+00	-1.97E+00	-1.23E+00	3.72E-01
GLN	15	NE2	225	GLN	15	HE21	2029	5.20E-01	-1.02E+00	-1.20E+00	-8.35E-01	1.84E-01
GLN	15	NE2	225	GLN	15	HE22	2030	5.00E-01	-1.05E+00	-1.07E+00	-1.03E+00	2.32E-02
GLN	15	NE2	225	LYS	16	C	2033	8.23E-01	-6.98E-01	-7.89E-01	-6.07E-01	9.11E-02
GLN	15	NE2	225	MET	35	C	2318	8.58E-01	-5.30E-01	-5.81E-01	-4.79E-01	5.13E-02
GLN	15	NE2	225	VAL	36	C	2335	7.19E-01	-5.56E-01	-6.44E-01	-4.68E-01	8.83E-02
GLN	15	NE2	225	VAL	36	CB	2337	6.49E-01	-6.51E-01	-8.25E-01	-4.77E-01	1.74E-01
GLN	15	NE2	225	VAL	36	H	2340	8.95E-01	-3.63E-01	-4.01E-01	-3.26E-01	3.76E-02
GLN	15	NE2	225	GLY	37	C	2351	8.17E-01	-5.54E-01	-6.02E-01	-5.05E-01	4.85E-02
GLN	15	NE2	225	GLY	37	H	2353	5.41E-01	-5.92E-01	-7.21E-01	-4.62E-01	1.30E-01
GLN	15	NE2	225	GLY	38	C	2358	9.48E-01	-4.30E-01	-4.51E-01	-4.09E-01	2.08E-02
GLN	15	H	226	HIS	14	N	1997	5.68E-01	-3.46E-01	-3.51E-01	-3.41E-01	5.13E-03
GLN	15	H	226	HIS	14	O	2000	3.82E-01	-9.14E-01	-9.44E-01	-8.84E-01	2.98E-02
GLN	15	H	226	GLN	15	N	2014	4.33E-01	-4.84E-01	-4.85E-01	-4.82E-01	1.70E-03
GLN	15	H	226	GLN	15	O	2017	5.85E-01	-3.47E-01	-3.51E-01	-3.44E-01	3.64E-03
GLN	15	H	226	GLN	15	NE2	2022	5.41E-01	-6.64E-01	-7.40E-01	-5.89E-01	7.57E-02
GLN	15	H	226	LYS	16	N	2031	4.95E-01	-3.92E-01	-4.00E-01	-3.84E-01	7.83E-03
GLN	15	HE21	232	GLN	15	NE2	2022	5.10E-01	-1.09E+00	-1.31E+00	-8.62E-01	2.25E-01
GLN	15	HE22	233	GLN	15	OE1	2021	5.75E-01	-7.49E-01	-1.05E+00	-4.48E-01	3.00E-01
GLN	15	HE22	233	GLN	15	NE2	2022	5.38E-01	-8.85E-01	-8.95E-01	-8.75E-01	1.01E-02
LYS	16	N	234	HIS	14	C	1999	7.30E-01	-3.76E-01	-3.79E-01	-3.72E-01	3.64E-03
LYS	16	N	234	GLN	15	C	2016	5.86E-01	-3.72E-01	-3.73E-01	-3.70E-01	1.41E-03
LYS	16	N	234	GLN	15	CD	2020	7.06E-01	-4.13E-01	-4.56E-01	-3.69E-01	4.34E-02
LYS	16	N	234	LYS	16	C	2033	5.30E-01	-8.05E-01	-8.22E-01	-7.87E-01	1.75E-02

LYS	16	N	234	LYS	16	H	2040	4.24E-01	-4.79E-01	-4.86E-01	-4.73E-01	6.57E-03
LYS	16	C	236	HIS	14	O	2000	8.72E-01	-3.65E-01	-3.68E-01	-3.61E-01	3.55E-03
LYS	16	C	236	GLN	15	O	2017	8.09E-01	-4.43E-01	-4.46E-01	-4.39E-01	3.03E-03
LYS	16	C	236	GLN	15	OE1	2021	8.36E-01	-4.86E-01	-5.55E-01	-4.17E-01	6.89E-02
LYS	16	C	236	GLN	15	NE2	2022	8.63E-01	-6.24E-01	-6.62E-01	-5.87E-01	3.73E-02
LYS	16	C	236	LYS	16	N	2031	5.91E-01	-6.31E-01	-6.38E-01	-6.23E-01	7.67E-03
LYS	16	C	236	LYS	16	O	2034	3.98E-01	-2.10E+00	-2.12E+00	-2.09E+00	1.18E-02
LYS	16	C	236	LEU	17	N	2053	5.77E-01	-5.40E-01	-5.43E-01	-5.37E-01	2.77E-03
LYS	16	C	236	LEU	17	O	2056	7.69E-01	-4.78E-01	-4.84E-01	-4.73E-01	5.53E-03
LYS	16	C	236	VAL	18	N	2072	6.74E-01	-4.96E-01	-5.02E-01	-4.89E-01	6.37E-03
LYS	16	O	237	LYS	16	C	2033	6.21E-01	-7.34E-01	-7.36E-01	-7.32E-01	2.08E-03
LYS	16	O	237	LEU	17	C	2055	7.64E-01	-3.86E-01	-3.89E-01	-3.84E-01	2.36E-03
LYS	16	O	237	VAL	18	H	2079	6.84E-01	-3.66E-01	-3.74E-01	-3.58E-01	8.32E-03
LYS	16	H	243	LYS	16	O	2034	5.25E-01	-3.68E-01	-3.78E-01	-3.58E-01	9.97E-03
LYS	16	HA	244	LYS	16	N	2031	3.81E-01	-3.25E-01	-3.33E-01	-3.17E-01	7.73E-03
LYS	16	HA	244	LYS	16	O	2034	2.47E-01	-1.42E+00	-1.47E+00	-1.38E+00	4.69E-02
LEU	17	N	256	LYS	16	C	2033	4.69E-01	-8.73E-01	-8.81E-01	-8.64E-01	8.48E-03
LEU	17	N	256	LEU	17	C	2055	5.71E-01	-4.37E-01	-4.40E-01	-4.34E-01	3.04E-03
LEU	17	N	256	LEU	17	HA	2062	3.62E-01	-3.23E-01	-3.27E-01	-3.19E-01	4.39E-03
LEU	17	N	256	VAL	18	H	2079	4.99E-01	-4.60E-01	-4.81E-01	-4.38E-01	2.13E-02
LEU	17	C	258	GLN	15	NE2	2022	9.61E-01	-4.20E-01	-4.54E-01	-3.85E-01	3.43E-02
LEU	17	C	258	LYS	16	N	2031	7.48E-01	-3.11E-01	-3.17E-01	-3.05E-01	5.76E-03
LEU	17	C	258	LYS	16	O	2034	4.83E-01	-1.02E+00	-1.07E+00	-9.77E-01	4.70E-02
LEU	17	C	258	LEU	17	N	2053	5.58E-01	-4.61E-01	-4.66E-01	-4.56E-01	5.19E-03
LEU	17	C	258	LEU	17	O	2056	6.25E-01	-5.67E-01	-5.71E-01	-5.63E-01	3.93E-03
LEU	17	C	258	VAL	18	N	2072	4.46E-01	-9.90E-01	-1.01E+00	-9.68E-01	2.27E-02
LEU	17	C	258	VAL	18	O	2075	5.16E-01	-6.29E-01	-6.53E-01	-6.06E-01	2.34E-02
LEU	17	O	259	GLN	15	CD	2020	8.83E-01	-3.49E-01	-3.60E-01	-3.37E-01	1.14E-02
LEU	17	O	259	LYS	16	C	2033	4.70E-01	-1.37E+00	-1.44E+00	-1.31E+00	6.51E-02
LEU	17	O	259	LEU	17	C	2055	3.86E-01	-1.79E+00	-1.84E+00	-1.74E+00	5.23E-02
LEU	17	O	259	LEU	17	CG	2058	3.83E-01	-6.83E-01	-8.36E-01	-5.29E-01	1.53E-01
LEU	17	O	259	LEU	17	H	2061	5.39E-01	-3.57E-01	-3.60E-01	-3.55E-01	2.67E-03
LEU	17	O	259	LEU	17	HA	2062	2.45E-01	-1.53E+00	-1.55E+00	-1.51E+00	1.89E-02
LEU	17	O	259	VAL	18	C	2074	5.00E-01	-7.32E-01	-7.73E-01	-6.91E-01	4.11E-02
LEU	17	O	259	VAL	18	CB	2076	4.59E-01	-7.91E-01	-8.16E-01	-7.66E-01	2.49E-02
LEU	17	O	259	VAL	18	H	2079	2.51E-01	-4.89E+00	-5.73E+00	-4.04E+00	8.46E-01
LEU	17	O	259	PHE	19	C	2090	8.23E-01	-3.21E-01	-3.28E-01	-3.15E-01	6.51E-03
LEU	17	H	264	LYS	16	N	2031	5.09E-01	-3.20E-01	-3.28E-01	-3.12E-01	8.32E-03
LEU	17	H	264	LYS	16	O	2034	3.10E-01	-1.50E+00	-1.52E+00	-1.48E+00	2.10E-02
LEU	17	H	264	LEU	17	N	2053	4.24E-01	-4.07E-01	-4.10E-01	-4.04E-01	2.91E-03
VAL	18	N	275	LYS	16	C	2033	6.88E-01	-4.75E-01	-4.85E-01	-4.66E-01	9.70E-03
VAL	18	N	275	LEU	17	C	2055	5.83E-01	-5.30E-01	-5.37E-01	-5.24E-01	6.48E-03

VAL	18	N	275	VAL	18	C	2074	5.88E-01	-4.06E-01	-4.10E-01	-4.02E-01	4.19E-03
VAL	18	N	275	VAL	18	CB	2076	5.53E-01	-4.11E-01	-4.24E-01	-3.99E-01	1.24E-02
VAL	18	N	275	VAL	18	H	2079	4.05E-01	-9.74E-01	-9.91E-01	-9.57E-01	1.73E-02
VAL	18	C	277	LEU	17	O	2056	7.43E-01	-3.15E-01	-3.17E-01	-3.12E-01	2.71E-03
VAL	18	C	277	VAL	18	N	2072	5.21E-01	-5.32E-01	-5.37E-01	-5.27E-01	4.86E-03
VAL	18	C	277	VAL	18	O	2075	3.84E-01	-1.03E+00	-1.04E+00	-1.01E+00	1.36E-02
VAL	18	C	277	PHE	19	N	2088	6.08E-01	-3.11E-01	-3.13E-01	-3.09E-01	2.14E-03
VAL	18	O	278	VAL	18	C	2074	6.14E-01	-3.33E-01	-3.34E-01	-3.32E-01	9.60E-04
VAL	18	O	278	VAL	18	H	2079	5.55E-01	-4.08E-01	-4.14E-01	-4.02E-01	6.18E-03
VAL	18	CB	279	VAL	18	N	2072	5.61E-01	-3.98E-01	-4.08E-01	-3.88E-01	9.74E-03
VAL	18	CB	279	VAL	18	O	2075	5.17E-01	-4.35E-01	-4.62E-01	-4.07E-01	2.73E-02
VAL	18	H	282	LYS	16	O	2034	6.77E-01	-3.74E-01	-3.80E-01	-3.67E-01	6.47E-03
VAL	18	H	282	VAL	18	N	2072	5.95E-01	-3.90E-01	-3.93E-01	-3.86E-01	3.37E-03
VAL	18	H	282	VAL	18	O	2075	5.82E-01	-3.68E-01	-3.75E-01	-3.60E-01	7.54E-03
PHE	19	N	291	LEU	17	C	2055	6.01E-01	-4.09E-01	-4.16E-01	-4.02E-01	6.81E-03
PHE	19	N	291	VAL	18	C	2074	4.01E-01	-8.35E-01	-8.39E-01	-8.31E-01	4.16E-03
PHE	19	N	291	VAL	18	CB	2076	4.73E-01	-4.92E-01	-5.21E-01	-4.62E-01	2.98E-02
PHE	19	N	291	VAL	18	H	2079	4.14E-01	-7.58E-01	-7.65E-01	-7.51E-01	7.04E-03
PHE	19	N	291	PHE	19	C	2090	5.66E-01	-4.60E-01	-5.22E-01	-3.99E-01	6.14E-02
PHE	19	C	293	VAL	18	N	2072	6.62E-01	-3.90E-01	-4.07E-01	-3.73E-01	1.70E-02
PHE	19	C	293	VAL	18	O	2075	3.94E-01	-1.20E+00	-1.33E+00	-1.08E+00	1.25E-01
PHE	19	C	293	PHE	19	N	2088	5.44E-01	-4.99E-01	-5.53E-01	-4.46E-01	5.35E-02
PHE	19	C	293	PHE	19	O	2091	5.98E-01	-5.48E-01	-5.93E-01	-5.02E-01	4.54E-02
PHE	19	C	293	PHE	20	N	2108	4.37E-01	-8.27E-01	-8.57E-01	-7.97E-01	3.00E-02
PHE	19	C	293	PHE	20	O	2111	6.78E-01	-4.33E-01	-4.95E-01	-3.71E-01	6.24E-02
PHE	19	C	293	ALA	21	N	2128	5.17E-01	-7.03E-01	-9.31E-01	-4.75E-01	2.28E-01
PHE	19	C	293	GLU	22	O	2141	8.65E-01	-3.14E-01	-3.15E-01	-3.12E-01	1.61E-03
PHE	19	C	293	ASP	23	OD1	2159	8.89E-01	-3.77E-01	-4.15E-01	-3.39E-01	3.78E-02
PHE	19	C	293	ASP	23	OD2	2160	8.45E-01	-4.19E-01	-4.74E-01	-3.64E-01	5.47E-02
PHE	19	O	294	LEU	17	C	2055	7.41E-01	-3.71E-01	-3.95E-01	-3.48E-01	2.34E-02
PHE	19	O	294	VAL	18	C	2074	4.38E-01	-9.83E-01	-1.16E+00	-8.09E-01	1.74E-01
PHE	19	O	294	VAL	18	CB	2076	5.40E-01	-4.95E-01	-5.36E-01	-4.55E-01	4.06E-02
PHE	19	O	294	VAL	18	H	2079	5.91E-01	-4.45E-01	-4.63E-01	-4.27E-01	1.79E-02
PHE	19	O	294	PHE	19	C	2090	4.13E-01	-1.38E+00	-1.62E+00	-1.15E+00	2.33E-01
PHE	19	O	294	PHE	20	C	2110	4.89E-01	-8.54E-01	-8.67E-01	-8.41E-01	1.33E-02
PHE	19	O	294	PHE	20	H	2119	3.06E-01	-2.09E+00	-2.82E+00	-1.36E+00	7.31E-01
PHE	19	O	294	ALA	21	C	2130	6.88E-01	-4.22E-01	-4.34E-01	-4.09E-01	1.27E-02
PHE	19	O	294	ALA	21	H	2133	3.79E-01	-8.88E-01	-9.70E-01	-8.07E-01	8.15E-02
PHE	19	H	302	VAL	18	N	2072	3.95E-01	-5.53E-01	-5.56E-01	-5.49E-01	3.20E-03
PHE	19	H	302	VAL	18	O	2075	1.91E-01	-4.03E+00	-4.16E+00	-3.90E+00	1.29E-01
PHE	19	H	302	PHE	19	N	2088	4.08E-01	-4.20E-01	-4.25E-01	-4.15E-01	4.77E-03
PHE	19	HB2	304	VAL	18	O	2075	2.52E-01	-4.53E-01	-4.56E-01	-4.51E-01	2.55E-03

PHE	20	N	311	PHE	19	C	2090	5.89E-01	-4.08E-01	-4.10E-01	-4.06E-01	2.30E-03
PHE	20	N	311	PHE	20	C	2110	5.91E-01	-4.53E-01	-5.71E-01	-3.35E-01	1.18E-01
PHE	20	C	313	PHE	19	O	2091	6.93E-01	-4.13E-01	-4.58E-01	-3.67E-01	4.54E-02
PHE	20	C	313	PHE	20	N	2108	5.05E-01	-6.53E-01	-7.90E-01	-5.17E-01	1.37E-01
PHE	20	C	313	PHE	20	O	2111	5.99E-01	-5.44E-01	-5.82E-01	-5.06E-01	3.80E-02
PHE	20	C	313	ALA	21	N	2128	4.18E-01	-1.01E+00	-1.05E+00	-9.74E-01	3.62E-02
PHE	20	C	313	ALA	21	O	2131	6.65E-01	-4.86E-01	-5.40E-01	-4.32E-01	5.41E-02
PHE	20	C	313	ALA	21	CB	2132	4.56E-01	-5.14E-01	-6.54E-01	-3.74E-01	1.40E-01
PHE	20	C	313	GLU	22	N	2138	5.43E-01	-5.58E-01	-5.69E-01	-5.47E-01	1.09E-02
PHE	20	C	313	GLU	22	O	2141	5.94E-01	-6.49E-01	-6.97E-01	-6.01E-01	4.79E-02
PHE	20	C	313	GLU	22	OE1	2145	9.40E-01	-3.82E-01	-3.97E-01	-3.67E-01	1.53E-02
PHE	20	C	313	GLU	22	OE2	2146	9.95E-01	-3.51E-01	-3.62E-01	-3.39E-01	1.16E-02
PHE	20	C	313	ASP	23	N	2153	7.29E-01	-4.12E-01	-4.57E-01	-3.66E-01	4.54E-02
PHE	20	C	313	ASP	23	OD1	2159	7.11E-01	-5.76E-01	-6.75E-01	-4.76E-01	9.92E-02
PHE	20	C	313	ASP	23	OD2	2160	6.84E-01	-6.76E-01	-8.35E-01	-5.17E-01	1.59E-01
PHE	20	O	314	PHE	19	C	2090	5.58E-01	-7.75E-01	-9.68E-01	-5.82E-01	1.93E-01
PHE	20	O	314	PHE	20	C	2110	4.04E-01	-1.47E+00	-1.71E+00	-1.22E+00	2.43E-01
PHE	20	O	314	ALA	21	C	2130	4.69E-01	-9.86E-01	-1.05E+00	-9.25E-01	6.09E-02
PHE	20	O	314	ALA	21	H	2133	2.71E-01	-3.53E+00	-4.74E+00	-2.32E+00	1.21E+00
PHE	20	O	314	GLU	22	C	2140	5.69E-01	-5.25E-01	-5.70E-01	-4.81E-01	4.46E-02
PHE	20	O	314	GLU	22	CD	2144	8.63E-01	-3.75E-01	-3.83E-01	-3.68E-01	7.61E-03
PHE	20	O	314	GLU	22	H	2147	3.90E-01	-8.46E-01	-8.57E-01	-8.34E-01	1.14E-02
PHE	20	O	314	ASP	23	CG	2158	6.19E-01	-7.68E-01	-9.21E-01	-6.14E-01	1.53E-01
ALA	21	N	331	PHE	20	C	2110	5.95E-01	-4.38E-01	-4.49E-01	-4.26E-01	1.18E-02
ALA	21	N	331	ALA	21	C	2130	6.06E-01	-4.39E-01	-4.63E-01	-4.14E-01	2.46E-02
ALA	21	N	331	ALA	21	H	2133	4.22E-01	-5.46E-01	-6.12E-01	-4.80E-01	6.60E-02
ALA	21	C	333	PHE	20	O	2111	6.87E-01	-4.23E-01	-4.27E-01	-4.18E-01	4.88E-03
ALA	21	C	333	ALA	21	N	2128	4.96E-01	-6.97E-01	-7.60E-01	-6.34E-01	6.32E-02
ALA	21	C	333	ALA	21	O	2131	5.98E-01	-6.19E-01	-6.56E-01	-5.81E-01	3.78E-02
ALA	21	C	333	ALA	21	CB	2132	4.10E-01	-6.30E-01	-6.75E-01	-5.86E-01	4.48E-02
ALA	21	C	333	GLU	22	N	2138	4.41E-01	-1.03E+00	-1.22E+00	-8.50E-01	1.84E-01
ALA	21	C	333	GLU	22	O	2141	4.31E-01	-1.50E+00	-1.74E+00	-1.26E+00	2.40E-01
ALA	21	C	333	GLU	22	OE1	2145	7.40E-01	-6.84E-01	-8.25E-01	-5.42E-01	1.42E-01
ALA	21	C	333	GLU	22	OE2	2146	7.85E-01	-5.71E-01	-6.62E-01	-4.81E-01	9.05E-02
ALA	21	C	333	ASP	23	N	2153	6.25E-01	-5.88E-01	-6.65E-01	-5.12E-01	7.62E-02
ALA	21	C	333	ASP	23	OD1	2159	6.99E-01	-6.24E-01	-7.27E-01	-5.21E-01	1.03E-01
ALA	21	C	333	ASP	23	OD2	2160	6.85E-01	-6.39E-01	-7.29E-01	-5.50E-01	8.92E-02
ALA	21	O	334	PHE	20	C	2110	5.55E-01	-7.35E-01	-8.41E-01	-6.29E-01	1.06E-01
ALA	21	O	334	ALA	21	C	2130	4.08E-01	-1.59E+00	-1.81E+00	-1.37E+00	2.19E-01
ALA	21	O	334	ALA	21	H	2133	4.08E-01	-9.72E-01	-1.23E+00	-7.15E-01	2.57E-01
ALA	21	O	334	ALA	21	HB2	2136	2.81E-01	-6.46E-01	-7.87E-01	-5.05E-01	1.41E-01
ALA	21	O	334	GLU	22	C	2140	4.21E-01	-1.44E+00	-1.82E+00	-1.06E+00	3.78E-01

ALA	21	O	334	GLU	22	CD	2144	6.77E-01	-7.64E-01	-9.37E-01	-5.90E-01	1.73E-01
ALA	21	O	334	GLU	22	H	2147	2.87E-01	-5.49E+00	-7.65E+00	-3.34E+00	2.16E+00
ALA	21	O	334	ASP	23	CG	2158	5.90E-01	-8.29E-01	-8.54E-01	-8.04E-01	2.50E-02
GLU	22	N	341	PHE	20	C	2110	6.98E-01	-3.31E-01	-3.47E-01	-3.14E-01	1.65E-02
GLU	22	N	341	ALA	21	C	2130	5.75E-01	-5.28E-01	-5.99E-01	-4.56E-01	7.12E-02
GLU	22	N	341	GLU	22	C	2140	5.31E-01	-5.08E-01	-5.43E-01	-4.74E-01	3.47E-02
GLU	22	N	341	GLU	22	CD	2144	6.96E-01	-4.90E-01	-5.60E-01	-4.19E-01	7.06E-02
GLU	22	N	341	GLU	22	H	2147	4.24E-01	-5.79E-01	-6.32E-01	-5.26E-01	5.30E-02
GLU	22	N	341	ASP	23	CG	2158	7.28E-01	-4.13E-01	-4.23E-01	-4.03E-01	1.02E-02
GLU	22	C	343	GLU	22	N	2138	5.77E-01	-4.19E-01	-4.43E-01	-3.95E-01	2.38E-02
GLU	22	C	343	GLU	22	O	2141	3.81E-01	-1.61E+00	-1.62E+00	-1.59E+00	1.31E-02
GLU	22	C	343	GLU	22	OE1	2145	6.79E-01	-6.74E-01	-8.28E-01	-5.20E-01	1.54E-01
GLU	22	C	343	GLU	22	OE2	2146	7.46E-01	-5.03E-01	-5.83E-01	-4.24E-01	7.95E-02
GLU	22	C	343	ASP	23	N	2153	5.95E-01	-5.16E-01	-5.19E-01	-5.12E-01	3.50E-03
GLU	22	C	343	ASP	23	OD1	2159	6.96E-01	-5.94E-01	-7.96E-01	-3.92E-01	2.02E-01
GLU	22	C	343	ASP	23	OD2	2160	7.18E-01	-4.68E-01	-5.07E-01	-4.29E-01	3.93E-02
GLU	22	O	344	ALA	21	C	2130	7.84E-01	-4.00E-01	-4.55E-01	-3.46E-01	5.43E-02
GLU	22	O	344	GLU	22	C	2140	6.24E-01	-4.95E-01	-4.97E-01	-4.93E-01	2.31E-03
GLU	22	O	344	GLU	22	CD	2144	7.82E-01	-5.41E-01	-6.05E-01	-4.77E-01	6.42E-02
GLU	22	O	344	ASP	23	CG	2158	7.74E-01	-5.27E-01	-5.79E-01	-4.75E-01	5.22E-02
GLU	22	CD	347	ALA	21	O	2131	8.97E-01	-4.09E-01	-4.78E-01	-3.40E-01	6.89E-02
GLU	22	CD	347	GLU	22	N	2138	6.96E-01	-5.14E-01	-6.44E-01	-3.85E-01	1.29E-01
GLU	22	CD	347	GLU	22	O	2141	5.72E-01	-1.06E+00	-1.29E+00	-8.18E-01	2.38E-01
GLU	22	CD	347	GLU	22	OE1	2145	4.60E-01	-2.69E+00	-3.42E+00	-1.97E+00	7.23E-01
GLU	22	CD	347	GLU	22	OE2	2146	5.23E-01	-1.83E+00	-2.32E+00	-1.35E+00	4.86E-01
GLU	22	CD	347	ASP	23	N	2153	7.52E-01	-5.81E-01	-7.17E-01	-4.45E-01	1.36E-01
GLU	22	OE1	348	ALA	21	C	2130	8.79E-01	-5.13E-01	-6.58E-01	-3.68E-01	1.45E-01
GLU	22	OE1	348	GLU	22	C	2140	7.13E-01	-6.29E-01	-8.36E-01	-4.22E-01	2.07E-01
GLU	22	OE1	348	GLU	22	CD	2144	5.47E-01	-1.71E+00	-2.21E+00	-1.22E+00	4.95E-01
GLU	22	OE2	349	ALA	21	C	2130	8.56E-01	-4.74E-01	-5.24E-01	-4.23E-01	5.01E-02
GLU	22	OE2	349	GLU	22	C	2140	6.93E-01	-5.83E-01	-6.75E-01	-4.91E-01	9.23E-02
GLU	22	OE2	349	GLU	22	CD	2144	4.94E-01	-2.05E+00	-2.41E+00	-1.68E+00	3.69E-01
GLU	22	H	350	GLU	22	O	2141	5.23E-01	-4.78E-01	-4.94E-01	-4.62E-01	1.63E-02
GLU	22	HA	351	GLU	22	O	2141	2.58E-01	-6.63E-01	-6.79E-01	-6.47E-01	1.59E-02
ASP	23	N	356	PHE	20	C	2110	7.89E-01	-3.46E-01	-3.56E-01	-3.36E-01	1.01E-02
ASP	23	N	356	ALA	21	C	2130	6.48E-01	-5.40E-01	-6.11E-01	-4.69E-01	7.10E-02
ASP	23	N	356	GLU	22	C	2140	4.33E-01	-1.09E+00	-1.09E+00	-1.08E+00	8.39E-03
ASP	23	N	356	GLU	22	CD	2144	6.73E-01	-6.66E-01	-7.31E-01	-6.02E-01	6.44E-02
ASP	23	N	356	GLU	22	H	2147	5.23E-01	-4.52E-01	-4.74E-01	-4.30E-01	2.19E-02
ASP	23	N	356	ASP	23	C	2155	6.02E-01	-4.75E-01	-4.95E-01	-4.54E-01	2.05E-02
ASP	23	N	356	ASP	23	CG	2158	5.66E-01	-9.37E-01	-1.06E+00	-8.15E-01	1.21E-01
ASP	23	N	356	ASP	23	H	2161	5.99E-01	-3.46E-01	-3.48E-01	-3.44E-01	1.85E-03

ASP	23	N	356	VAL	24	H	2172	5.56E-01	-5.75E-01	-6.47E-01	-5.04E-01	7.14E-02
ASP	23	C	358	GLU	22	O	2141	4.20E-01	-1.19E+00	-1.28E+00	-1.10E+00	8.78E-02
ASP	23	C	358	GLU	22	OE1	2145	7.92E-01	-4.27E-01	-4.81E-01	-3.73E-01	5.40E-02
ASP	23	C	358	GLU	22	OE2	2146	8.32E-01	-3.82E-01	-4.15E-01	-3.48E-01	3.37E-02
ASP	23	C	358	ASP	23	N	2153	5.17E-01	-6.69E-01	-7.00E-01	-6.38E-01	3.09E-02
ASP	23	C	358	ASP	23	O	2156	6.20E-01	-4.00E-01	-4.02E-01	-3.97E-01	2.21E-03
ASP	23	C	358	ASP	23	OD1	2159	5.66E-01	-7.53E-01	-8.97E-01	-6.09E-01	1.44E-01
ASP	23	C	358	ASP	23	OD2	2160	6.23E-01	-5.80E-01	-6.07E-01	-5.54E-01	2.66E-02
ASP	23	C	358	VAL	24	N	2165	4.75E-01	-6.65E-01	-7.24E-01	-6.06E-01	5.86E-02
ASP	23	O	359	ALA	21	C	2130	7.07E-01	-3.95E-01	-3.99E-01	-3.90E-01	4.40E-03
ASP	23	O	359	GLU	22	C	2140	4.27E-01	-1.02E+00	-1.08E+00	-9.62E-01	5.81E-02
ASP	23	O	359	GLU	22	CD	2144	7.26E-01	-5.04E-01	-5.16E-01	-4.93E-01	1.16E-02
ASP	23	O	359	ASP	23	C	2155	3.94E-01	-1.18E+00	-1.23E+00	-1.13E+00	4.79E-02
ASP	23	O	359	ASP	23	CG	2158	4.39E-01	-1.52E+00	-1.64E+00	-1.39E+00	1.26E-01
ASP	23	O	359	ASP	23	H	2161	5.04E-01	-4.60E-01	-4.76E-01	-4.43E-01	1.64E-02
ASP	23	O	359	ASP	23	HA	2162	2.44E-01	-8.30E-01	-8.33E-01	-8.28E-01	2.55E-03
ASP	23	O	359	VAL	24	C	2167	5.67E-01	-4.97E-01	-5.42E-01	-4.51E-01	4.54E-02
ASP	23	O	359	VAL	24	CB	2169	5.28E-01	-5.64E-01	-7.11E-01	-4.17E-01	1.47E-01
ASP	23	O	359	VAL	24	H	2172	3.07E-01	-2.78E+00	-3.83E+00	-1.74E+00	1.04E+00
ASP	23	CG	361	PHE	20	O	2111	8.47E-01	-3.81E-01	-4.04E-01	-3.58E-01	2.28E-02
ASP	23	CG	361	ALA	21	N	2128	7.28E-01	-4.05E-01	-4.44E-01	-3.65E-01	3.96E-02
ASP	23	CG	361	ALA	21	O	2131	7.75E-01	-5.05E-01	-5.83E-01	-4.27E-01	7.82E-02
ASP	23	CG	361	GLU	22	N	2138	6.76E-01	-4.76E-01	-4.87E-01	-4.65E-01	1.07E-02
ASP	23	CG	361	GLU	22	O	2141	4.79E-01	-1.43E+00	-1.46E+00	-1.39E+00	3.44E-02
ASP	23	CG	361	GLU	22	OE2	2146	9.95E-01	-4.78E-01	-5.04E-01	-4.52E-01	2.61E-02
ASP	23	CG	361	ASP	23	N	2153	6.00E-01	-8.12E-01	-8.69E-01	-7.55E-01	5.67E-02
ASP	23	CG	361	ASP	23	O	2156	7.48E-01	-4.70E-01	-5.05E-01	-4.35E-01	3.48E-02
ASP	23	CG	361	ASP	23	OD1	2159	5.17E-01	-1.70E+00	-2.23E+00	-1.17E+00	5.28E-01
ASP	23	CG	361	ASP	23	OD2	2160	5.58E-01	-1.24E+00	-1.31E+00	-1.17E+00	6.61E-02
ASP	23	CG	361	VAL	24	N	2165	6.77E-01	-5.06E-01	-5.24E-01	-4.87E-01	1.84E-02
ASP	23	OD1	362	PHE	20	C	2110	7.91E-01	-4.86E-01	-5.67E-01	-4.05E-01	8.11E-02
ASP	23	OD1	362	ALA	21	C	2130	7.57E-01	-5.10E-01	-5.43E-01	-4.77E-01	3.34E-02
ASP	23	OD1	362	GLU	22	C	2140	6.09E-01	-6.63E-01	-7.44E-01	-5.83E-01	8.05E-02
ASP	23	OD1	362	ASP	23	C	2155	6.59E-01	-5.55E-01	-6.48E-01	-4.61E-01	9.32E-02
ASP	23	OD1	362	ASP	23	CG	2158	5.07E-01	-1.66E+00	-1.95E+00	-1.36E+00	2.94E-01
ASP	23	OD1	362	VAL	24	H	2172	6.19E-01	-6.08E-01	-6.93E-01	-5.23E-01	8.54E-02
ASP	23	OD1	362	LYS	28	HZ3	2234	6.02E-01	-4.32E-01	-5.10E-01	-3.53E-01	7.88E-02
ASP	23	OD2	363	PHE	20	C	2110	7.12E-01	-5.82E-01	-6.70E-01	-4.93E-01	8.86E-02
ASP	23	OD2	363	ALA	21	C	2130	6.67E-01	-6.62E-01	-7.41E-01	-5.84E-01	7.83E-02
ASP	23	OD2	363	GLU	22	C	2140	5.52E-01	-8.05E-01	-8.60E-01	-7.50E-01	5.50E-02
ASP	23	OD2	363	GLU	22	CD	2144	9.16E-01	-4.97E-01	-5.30E-01	-4.64E-01	3.30E-02
ASP	23	OD2	363	ASP	23	C	2155	6.74E-01	-4.98E-01	-5.40E-01	-4.56E-01	4.20E-02

ASP	23	OD2	363	ASP	23	CG	2158	4.84E-01	-1.74E+00	-1.89E+00	-1.59E+00	1.50E-01
ASP	23	OD2	363	VAL	24	H	2172	6.64E-01	-5.09E-01	-5.51E-01	-4.66E-01	4.22E-02
ASP	23	H	364	GLU	22	N	2138	4.72E-01	-4.53E-01	-4.63E-01	-4.42E-01	1.04E-02
ASP	23	H	364	GLU	22	O	2141	2.20E-01	-5.26E+00	-5.49E+00	-5.04E+00	2.27E-01
ASP	23	H	364	GLU	22	OE1	2145	6.26E-01	-5.12E-01	-6.09E-01	-4.14E-01	9.74E-02
ASP	23	H	364	GLU	22	OE2	2146	6.75E-01	-4.08E-01	-4.57E-01	-3.59E-01	4.92E-02
ASP	23	H	364	ASP	23	N	2153	4.07E-01	-8.67E-01	-8.74E-01	-8.61E-01	6.56E-03
ASP	23	H	364	ASP	23	OD1	2159	5.23E-01	-8.35E-01	-1.22E+00	-4.47E-01	3.88E-01
ASP	23	H	364	ASP	23	OD2	2160	5.49E-01	-5.58E-01	-6.10E-01	-5.07E-01	5.13E-02
VAL	24	N	368	GLU	22	C	2140	6.21E-01	-3.83E-01	-4.05E-01	-3.60E-01	2.23E-02
VAL	24	N	368	GLU	22	CD	2144	8.47E-01	-3.45E-01	-3.60E-01	-3.30E-01	1.49E-02
VAL	24	N	368	ASP	23	C	2155	5.63E-01	-4.43E-01	-4.62E-01	-4.24E-01	1.87E-02
VAL	24	N	368	ASP	23	CG	2158	6.44E-01	-5.60E-01	-5.76E-01	-5.44E-01	1.59E-02
VAL	24	N	368	VAL	24	CB	2169	5.69E-01	-3.94E-01	-4.40E-01	-3.48E-01	4.57E-02
VAL	24	N	368	VAL	24	H	2172	4.12E-01	-9.31E-01	-9.46E-01	-9.15E-01	1.54E-02
VAL	24	C	370	GLU	22	O	2141	6.47E-01	-4.45E-01	-4.85E-01	-4.06E-01	3.96E-02
VAL	24	C	370	ASP	23	N	2153	6.47E-01	-4.22E-01	-4.64E-01	-3.80E-01	4.17E-02
VAL	24	C	370	ASP	23	O	2156	6.11E-01	-4.25E-01	-4.64E-01	-3.86E-01	3.90E-02
VAL	24	C	370	ASP	23	OD1	2159	6.19E-01	-6.11E-01	-6.88E-01	-5.34E-01	7.75E-02
VAL	24	C	370	ASP	23	OD2	2160	6.95E-01	-5.01E-01	-5.92E-01	-4.10E-01	9.12E-02
VAL	24	C	370	VAL	24	N	2165	4.23E-01	-8.93E-01	-9.63E-01	-8.23E-01	7.02E-02
VAL	24	C	370	VAL	24	O	2168	5.01E-01	-5.55E-01	-6.39E-01	-4.70E-01	8.46E-02
VAL	24	C	370	VAL	24	HB	2174	3.48E-01	-4.07E-01	-4.88E-01	-3.25E-01	8.17E-02
VAL	24	C	370	SER	26	N	2188	6.03E-01	-5.29E-01	-6.82E-01	-3.75E-01	1.54E-01
VAL	24	C	370	SER	26	OG	2193	7.09E-01	-4.35E-01	-5.39E-01	-3.31E-01	1.04E-01
VAL	24	O	371	ASP	23	C	2155	5.23E-01	-4.75E-01	-5.11E-01	-4.38E-01	3.62E-02
VAL	24	O	371	ASP	23	CG	2158	5.74E-01	-6.51E-01	-7.03E-01	-6.00E-01	5.13E-02
VAL	24	O	371	VAL	24	C	2167	5.34E-01	-5.13E-01	-6.59E-01	-3.67E-01	1.46E-01
VAL	24	O	371	VAL	24	CB	2169	5.10E-01	-5.44E-01	-7.45E-01	-3.43E-01	2.01E-01
VAL	24	O	371	VAL	24	H	2172	3.88E-01	-1.16E+00	-1.56E+00	-7.56E-01	4.00E-01
VAL	24	CB	372	VAL	24	N	2165	5.54E-01	-4.16E-01	-4.52E-01	-3.80E-01	3.59E-02
VAL	24	H	375	GLU	22	O	2141	6.01E-01	-5.07E-01	-5.37E-01	-4.76E-01	3.01E-02
VAL	24	H	375	ASP	23	N	2153	7.02E-01	-3.46E-01	-3.62E-01	-3.30E-01	1.60E-02
VAL	24	H	375	ASP	23	OD1	2159	7.42E-01	-4.17E-01	-4.66E-01	-3.67E-01	4.95E-02
VAL	24	H	375	ASP	23	OD2	2160	8.08E-01	-3.49E-01	-3.59E-01	-3.38E-01	1.04E-02
VAL	24	H	375	VAL	24	N	2165	5.98E-01	-3.86E-01	-3.88E-01	-3.84E-01	2.01E-03
GLY	25	N	384	VAL	24	C	2167	4.56E-01	-6.76E-01	-8.10E-01	-5.42E-01	1.34E-01
GLY	25	N	384	VAL	24	CB	2169	3.98E-01	-8.25E-01	-9.77E-01	-6.74E-01	1.51E-01
GLY	25	N	384	VAL	24	H	2172	3.62E-01	-1.39E+00	-1.79E+00	-1.00E+00	3.92E-01
GLY	25	N	384	GLY	25	C	2183	5.78E-01	-4.77E-01	-5.56E-01	-3.98E-01	7.93E-02
GLY	25	N	384	SER	26	H	2194	4.61E-01	-4.71E-01	-5.24E-01	-4.18E-01	5.28E-02
GLY	25	C	386	ASP	23	OD1	2159	7.38E-01	-5.91E-01	-7.02E-01	-4.79E-01	1.12E-01

GLY	25	C	386	VAL	24	N	2165	5.95E-01	-5.57E-01	-6.53E-01	-4.62E-01	9.54E-02
GLY	25	C	386	GLY	25	N	2181	5.45E-01	-5.31E-01	-5.84E-01	-4.77E-01	5.36E-02
GLY	25	C	386	GLY	25	O	2184	5.71E-01	-6.84E-01	-8.28E-01	-5.41E-01	1.44E-01
GLY	25	C	386	SER	26	N	2188	4.27E-01	-1.39E+00	-1.55E+00	-1.23E+00	1.60E-01
GLY	25	C	386	SER	26	O	2191	5.66E-01	-7.70E-01	-8.68E-01	-6.72E-01	9.77E-02
GLY	25	C	386	SER	26	OG	2193	5.19E-01	-1.03E+00	-1.15E+00	-9.03E-01	1.25E-01
GLY	25	C	386	ASN	27	N	2199	5.54E-01	-5.75E-01	-5.88E-01	-5.61E-01	1.36E-02
GLY	25	C	386	ASN	27	O	2202	6.60E-01	-4.93E-01	-5.40E-01	-4.46E-01	4.73E-02
GLY	25	C	386	ASN	27	ND2	2206	9.59E-01	-3.73E-01	-3.85E-01	-3.60E-01	1.26E-02
GLY	25	O	387	GLY	25	C	2183	4.57E-01	-1.16E+00	-1.36E+00	-9.51E-01	2.06E-01
GLY	25	O	387	SER	26	C	2190	4.40E-01	-1.18E+00	-1.47E+00	-8.85E-01	2.95E-01
GLY	25	O	387	SER	26	HA	2195	4.19E-01	-3.28E-01	-3.51E-01	-3.05E-01	2.31E-02
GLY	25	O	387	ASN	27	C	2201	5.80E-01	-7.58E-01	-9.25E-01	-5.91E-01	1.67E-01
GLY	25	O	387	ASN	27	CG	2204	7.87E-01	-3.39E-01	-3.53E-01	-3.24E-01	1.41E-02
GLY	25	O	387	ASN	27	H	2207	4.62E-01	-4.59E-01	-4.77E-01	-4.41E-01	1.80E-02
SER	26	N	391	ASP	23	CG	2158	8.02E-01	-4.45E-01	-4.64E-01	-4.25E-01	1.91E-02
SER	26	N	391	VAL	24	C	2167	6.20E-01	-4.53E-01	-5.08E-01	-3.97E-01	5.52E-02
SER	26	N	391	VAL	24	H	2172	5.95E-01	-4.72E-01	-5.05E-01	-4.39E-01	3.27E-02
SER	26	N	391	GLY	25	C	2183	6.01E-01	-6.16E-01	-6.79E-01	-5.53E-01	6.31E-02
SER	26	N	391	SER	26	C	2190	5.64E-01	-5.80E-01	-6.12E-01	-5.48E-01	3.19E-02
SER	26	N	391	SER	26	CB	2192	4.10E-01	-3.80E-01	-3.89E-01	-3.72E-01	8.69E-03
SER	26	N	391	SER	26	H	2194	4.70E-01	-6.63E-01	-7.59E-01	-5.68E-01	9.56E-02
SER	26	N	391	SER	26	HG	2198	5.49E-01	-5.75E-01	-6.30E-01	-5.20E-01	5.54E-02
SER	26	N	391	ASN	27	C	2201	6.72E-01	-5.15E-01	-5.46E-01	-4.84E-01	3.10E-02
SER	26	C	393	GLY	25	O	2184	7.05E-01	-3.54E-01	-4.04E-01	-3.05E-01	4.96E-02
SER	26	C	393	SER	26	N	2188	5.63E-01	-5.79E-01	-5.88E-01	-5.70E-01	8.62E-03
SER	26	C	393	SER	26	O	2191	5.79E-01	-5.86E-01	-6.07E-01	-5.66E-01	2.07E-02
SER	26	C	393	SER	26	OG	2193	5.02E-01	-9.15E-01	-1.01E+00	-8.17E-01	9.80E-02
SER	26	C	393	ASN	27	N	2199	4.90E-01	-6.38E-01	-6.74E-01	-6.02E-01	3.55E-02
SER	26	C	393	ASN	27	O	2202	4.71E-01	-8.78E-01	-9.81E-01	-7.75E-01	1.03E-01
SER	26	C	393	ASN	27	ND2	2206	8.07E-01	-4.13E-01	-4.39E-01	-3.88E-01	2.54E-02
SER	26	C	393	LYS	28	N	2213	5.71E-01	-4.84E-01	-5.61E-01	-4.07E-01	7.70E-02
SER	26	O	394	ASP	23	CG	2158	8.69E-01	-4.15E-01	-4.28E-01	-4.02E-01	1.30E-02
SER	26	O	394	GLY	25	C	2183	6.18E-01	-6.17E-01	-6.56E-01	-5.78E-01	3.90E-02
SER	26	O	394	SER	26	C	2190	4.60E-01	-1.04E+00	-1.19E+00	-8.83E-01	1.53E-01
SER	26	O	394	SER	26	CB	2192	3.98E-01	-4.71E-01	-5.72E-01	-3.69E-01	1.02E-01
SER	26	O	394	SER	26	H	2194	5.13E-01	-5.57E-01	-6.01E-01	-5.13E-01	4.39E-02
SER	26	O	394	SER	26	HG	2198	5.40E-01	-6.43E-01	-7.01E-01	-5.84E-01	5.85E-02
SER	26	O	394	ASN	27	C	2201	4.50E-01	-1.37E+00	-1.48E+00	-1.26E+00	1.11E-01
SER	26	O	394	ASN	27	CG	2204	6.85E-01	-5.01E-01	-5.20E-01	-4.82E-01	1.92E-02
SER	26	O	394	ASN	27	H	2207	4.49E-01	-6.71E-01	-9.08E-01	-4.34E-01	2.37E-01
SER	26	O	394	LYS	28	C	2215	6.10E-01	-8.62E-01	-1.02E+00	-7.05E-01	1.58E-01

SER	26	OG	396	GLY	25	C	2183	8.11E-01	-4.12E-01	-4.62E-01	-3.63E-01	4.95E-02
SER	26	OG	396	SER	26	C	2190	6.83E-01	-4.72E-01	-5.31E-01	-4.12E-01	6.00E-02
SER	26	OG	396	SER	26	H	2194	6.64E-01	-3.47E-01	-3.65E-01	-3.29E-01	1.79E-02
SER	26	OG	396	SER	26	HG	2198	5.36E-01	-7.37E-01	-8.52E-01	-6.23E-01	1.14E-01
SER	26	OG	396	ASN	27	C	2201	7.39E-01	-5.04E-01	-5.19E-01	-4.89E-01	1.50E-02
SER	26	OG	396	ASN	27	CG	2204	9.12E-01	-3.31E-01	-3.35E-01	-3.27E-01	4.15E-03
SER	26	OG	396	LYS	28	C	2215	9.49E-01	-3.86E-01	-3.91E-01	-3.81E-01	4.71E-03
SER	26	H	397	SER	26	N	2188	5.50E-01	-4.65E-01	-5.59E-01	-3.72E-01	9.34E-02
SER	26	H	397	SER	26	OG	2193	5.58E-01	-5.21E-01	-6.04E-01	-4.37E-01	8.37E-02
SER	26	HG	401	SER	26	OG	2193	4.95E-01	-8.95E-01	-1.03E+00	-7.57E-01	1.38E-01
ASN	27	N	402	SER	26	C	2190	5.62E-01	-4.63E-01	-4.65E-01	-4.60E-01	2.65E-03
ASN	27	N	402	ASN	27	C	2201	5.17E-01	-7.46E-01	-8.47E-01	-6.45E-01	1.01E-01
ASN	27	N	402	ASN	27	CG	2204	6.66E-01	-3.91E-01	-3.97E-01	-3.85E-01	5.89E-03
ASN	27	N	402	ASN	27	H	2207	4.67E-01	-3.86E-01	-4.32E-01	-3.40E-01	4.59E-02
ASN	27	C	404	SER	26	N	2188	7.95E-01	-3.74E-01	-3.90E-01	-3.58E-01	1.60E-02
ASN	27	C	404	SER	26	O	2191	7.09E-01	-4.97E-01	-5.28E-01	-4.65E-01	3.13E-02
ASN	27	C	404	SER	26	OG	2193	7.13E-01	-5.38E-01	-5.54E-01	-5.23E-01	1.58E-02
ASN	27	C	404	ASN	27	N	2199	6.07E-01	-5.25E-01	-6.15E-01	-4.36E-01	8.98E-02
ASN	27	C	404	ASN	27	O	2202	4.14E-01	-1.51E+00	-1.57E+00	-1.44E+00	6.99E-02
ASN	27	C	404	ASN	27	OD1	2205	6.45E-01	-5.46E-01	-5.82E-01	-5.10E-01	3.61E-02
ASN	27	C	404	ASN	27	ND2	2206	7.50E-01	-6.49E-01	-7.88E-01	-5.09E-01	1.39E-01
ASN	27	C	404	LYS	28	N	2213	5.77E-01	-5.71E-01	-6.05E-01	-5.37E-01	3.41E-02
ASN	27	C	404	LYS	28	O	2216	7.16E-01	-4.86E-01	-5.42E-01	-4.30E-01	5.60E-02
ASN	27	C	404	GLY	29	N	2235	5.67E-01	-5.48E-01	-6.47E-01	-4.49E-01	9.90E-02
ASN	27	O	405	ASN	27	C	2201	6.19E-01	-5.84E-01	-5.92E-01	-5.76E-01	8.05E-03
ASN	27	O	405	ASN	27	CG	2204	7.43E-01	-3.88E-01	-4.11E-01	-3.65E-01	2.32E-02
ASN	27	O	405	LYS	28	C	2215	6.95E-01	-5.61E-01	-6.25E-01	-4.97E-01	6.38E-02
ASN	27	CG	407	SER	26	O	2191	7.72E-01	-3.98E-01	-4.02E-01	-3.95E-01	3.50E-03
ASN	27	CG	407	SER	26	OG	2193	7.83E-01	-4.39E-01	-4.80E-01	-3.97E-01	4.17E-02
ASN	27	CG	407	ASN	27	N	2199	6.08E-01	-4.73E-01	-4.96E-01	-4.50E-01	2.32E-02
ASN	27	CG	407	ASN	27	O	2202	4.08E-01	-1.62E+00	-1.92E+00	-1.31E+00	3.02E-01
ASN	27	CG	407	ASN	27	OD1	2205	4.87E-01	-1.07E+00	-1.29E+00	-8.48E-01	2.19E-01
ASN	27	CG	407	ASN	27	ND2	2206	5.78E-01	-1.09E+00	-1.40E+00	-7.77E-01	3.12E-01
ASN	27	CG	407	LYS	28	N	2213	6.22E-01	-4.68E-01	-5.18E-01	-4.18E-01	5.01E-02
ASN	27	OD1	408	ASN	27	C	2201	5.52E-01	-7.87E-01	-9.12E-01	-6.61E-01	1.26E-01
ASN	27	OD1	408	ASN	27	CG	2204	5.44E-01	-9.38E-01	-1.31E+00	-5.65E-01	3.73E-01
ASN	27	OD1	408	LYS	28	C	2215	6.49E-01	-6.34E-01	-6.79E-01	-5.89E-01	4.52E-02
ASN	27	ND2	409	GLY	25	C	2183	9.92E-01	-3.54E-01	-3.64E-01	-3.45E-01	9.23E-03
ASN	27	ND2	409	SER	26	C	2190	7.24E-01	-5.11E-01	-5.60E-01	-4.62E-01	4.87E-02
ASN	27	ND2	409	ASN	27	C	2201	5.11E-01	-1.59E+00	-1.97E+00	-1.21E+00	3.78E-01
ASN	27	ND2	409	ASN	27	CG	2204	4.55E-01	-1.89E+00	-2.29E+00	-1.49E+00	4.02E-01
ASN	27	ND2	409	ASN	27	H	2207	5.88E-01	-4.10E-01	-4.45E-01	-3.74E-01	3.52E-02

ASN	27	ND2	409	ASN	27	HD21	2211	5.36E-01	-8.02E-01	-1.05E+00	-5.48E-01	2.53E-01
ASN	27	ND2	409	ASN	27	HD22	2212	5.43E-01	-6.69E-01	-6.77E-01	-6.61E-01	7.83E-03
ASN	27	ND2	409	LYS	28	C	2215	6.48E-01	-1.10E+00	-1.35E+00	-8.52E-01	2.47E-01
ASN	27	HD21	414	ASN	27	ND2	2206	4.91E-01	-9.08E-01	-1.06E+00	-7.54E-01	1.54E-01
ASN	27	HD22	415	ASN	27	O	2202	4.41E-01	-9.30E-01	-1.18E+00	-6.79E-01	2.51E-01
ASN	27	HD22	415	ASN	27	ND2	2206	4.97E-01	-8.30E-01	-8.92E-01	-7.68E-01	6.20E-02
LYS	28	N	416	ASN	27	C	2201	4.76E-01	-9.36E-01	-1.12E+00	-7.55E-01	1.81E-01
LYS	28	N	416	ASN	27	CG	2204	6.74E-01	-4.12E-01	-4.89E-01	-3.35E-01	7.70E-02
LYS	28	N	416	LYS	28	C	2215	5.28E-01	-8.41E-01	-9.48E-01	-7.34E-01	1.07E-01
LYS	28	N	416	GLY	29	H	2239	4.03E-01	-6.32E-01	-7.80E-01	-4.83E-01	1.48E-01
LYS	28	C	418	SER	26	O	2191	8.08E-01	-4.77E-01	-5.48E-01	-4.05E-01	7.11E-02
LYS	28	C	418	SER	26	OG	2193	9.08E-01	-4.37E-01	-5.09E-01	-3.65E-01	7.20E-02
LYS	28	C	418	ASN	27	N	2199	7.72E-01	-3.98E-01	-4.84E-01	-3.13E-01	8.54E-02
LYS	28	C	418	ASN	27	O	2202	4.95E-01	-1.59E+00	-2.36E+00	-8.27E-01	7.65E-01
LYS	28	C	418	ASN	27	OD1	2205	7.15E-01	-5.23E-01	-5.63E-01	-4.84E-01	3.94E-02
LYS	28	C	418	ASN	27	ND2	2206	8.36E-01	-6.86E-01	-8.99E-01	-4.73E-01	2.13E-01
LYS	28	C	418	LYS	28	N	2213	5.99E-01	-6.44E-01	-7.54E-01	-5.34E-01	1.10E-01
LYS	28	C	418	LYS	28	O	2216	6.26E-01	-7.20E-01	-7.30E-01	-7.11E-01	9.61E-03
LYS	28	C	418	GLY	29	N	2235	4.16E-01	-1.25E+00	-1.29E+00	-1.20E+00	4.58E-02
LYS	28	C	418	GLY	29	O	2238	6.53E-01	-6.04E-01	-6.45E-01	-5.62E-01	4.16E-02
LYS	28	C	418	ALA	30	N	2242	6.54E-01	-4.80E-01	-5.22E-01	-4.39E-01	4.14E-02
LYS	28	C	418	ALA	30	O	2245	7.64E-01	-4.90E-01	-5.40E-01	-4.41E-01	4.94E-02
LYS	28	O	419	ASN	27	C	2201	5.08E-01	-1.23E+00	-1.72E+00	-7.46E-01	4.89E-01
LYS	28	O	419	ASN	27	CG	2204	6.70E-01	-5.73E-01	-7.31E-01	-4.15E-01	1.58E-01
LYS	28	O	419	LYS	28	C	2215	4.01E-01	-2.07E+00	-2.19E+00	-1.94E+00	1.24E-01
LYS	28	O	419	LYS	28	HA	2223	3.41E-01	-6.36E-01	-8.13E-01	-4.58E-01	1.78E-01
LYS	28	O	419	GLY	29	C	2237	4.85E-01	-1.06E+00	-1.19E+00	-9.29E-01	1.30E-01
LYS	28	O	419	GLY	29	H	2239	2.20E-01	-3.97E+00	-4.21E+00	-3.72E+00	2.47E-01
LYS	28	O	419	ALA	30	C	2244	7.04E-01	-4.57E-01	-5.02E-01	-4.12E-01	4.47E-02
LYS	28	O	419	ALA	30	H	2247	5.33E-01	-4.35E-01	-5.03E-01	-3.66E-01	6.85E-02
LYS	28	NZ	424	ASP	23	CG	2158	5.94E-01	-3.69E-01	-3.87E-01	-3.50E-01	1.82E-02
LYS	28	H	425	ASN	27	O	2202	3.35E-01	-1.55E+00	-2.34E+00	-7.51E-01	7.95E-01
LYS	28	H	425	LYS	28	N	2213	4.33E-01	-4.54E-01	-4.65E-01	-4.42E-01	1.18E-02
LYS	28	HZ1	435	ASP	23	OD1	2159	6.26E-01	-3.84E-01	-4.11E-01	-3.57E-01	2.73E-02
LYS	28	HZ2	436	ASP	23	OD1	2159	5.84E-01	-4.67E-01	-5.51E-01	-3.83E-01	8.40E-02
LYS	28	HZ3	437	ASP	23	OD1	2159	5.55E-01	-5.21E-01	-6.04E-01	-4.38E-01	8.31E-02
GLY	29	N	438	LYS	28	C	2215	6.20E-01	-4.90E-01	-5.13E-01	-4.67E-01	2.29E-02
GLY	29	N	438	GLY	29	C	2237	6.56E-01	-3.50E-01	-3.65E-01	-3.36E-01	1.44E-02
GLY	29	N	438	GLY	29	H	2239	4.26E-01	-4.13E-01	-4.36E-01	-3.90E-01	2.32E-02
GLY	29	C	440	ASN	27	O	2202	7.05E-01	-4.72E-01	-5.90E-01	-3.54E-01	1.18E-01
GLY	29	C	440	LYS	28	O	2216	6.81E-01	-4.95E-01	-5.41E-01	-4.50E-01	4.53E-02
GLY	29	C	440	GLY	29	N	2235	4.61E-01	-8.19E-01	-9.81E-01	-6.58E-01	1.62E-01

GLY	29	C	440	GLY	29	O	2238	6.03E-01	-5.66E-01	-5.79E-01	-5.53E-01	1.30E-02
GLY	29	C	440	ALA	30	N	2242	5.10E-01	-6.56E-01	-6.87E-01	-6.25E-01	3.12E-02
GLY	29	C	440	ALA	30	O	2245	5.42E-01	-7.98E-01	-8.90E-01	-7.06E-01	9.22E-02
GLY	29	C	440	ILE	31	N	2252	6.79E-01	-3.95E-01	-4.15E-01	-3.74E-01	2.08E-02
GLY	29	O	441	ASN	27	C	2201	7.13E-01	-4.76E-01	-5.93E-01	-3.58E-01	1.18E-01
GLY	29	O	441	LYS	28	C	2215	5.01E-01	-1.19E+00	-1.49E+00	-8.82E-01	3.03E-01
GLY	29	O	441	GLY	29	C	2237	4.40E-01	-1.19E+00	-1.28E+00	-1.10E+00	9.30E-02
GLY	29	O	441	GLY	29	H	2239	3.13E-01	-2.03E+00	-3.19E+00	-8.60E-01	1.17E+00
GLY	29	O	441	ALA	30	C	2244	5.39E-01	-7.25E-01	-8.04E-01	-6.46E-01	7.88E-02
GLY	29	O	441	ALA	30	H	2247	4.26E-01	-6.82E-01	-8.05E-01	-5.60E-01	1.22E-01
ALA	30	N	445	LYS	28	C	2215	6.79E-01	-4.49E-01	-4.97E-01	-4.02E-01	4.77E-02
ALA	30	N	445	GLY	29	C	2237	5.45E-01	-5.63E-01	-5.91E-01	-5.35E-01	2.82E-02
ALA	30	N	445	ALA	30	C	2244	5.65E-01	-5.10E-01	-5.28E-01	-4.93E-01	1.75E-02
ALA	30	N	445	ALA	30	H	2247	4.44E-01	-4.68E-01	-4.98E-01	-4.38E-01	3.02E-02
ALA	30	C	447	GLY	29	O	2238	7.07E-01	-4.03E-01	-4.20E-01	-3.87E-01	1.64E-02
ALA	30	C	447	ALA	30	N	2242	5.64E-01	-5.16E-01	-5.56E-01	-4.76E-01	4.01E-02
ALA	30	C	447	ALA	30	O	2245	4.09E-01	-1.53E+00	-1.63E+00	-1.44E+00	9.53E-02
ALA	30	C	447	ILE	31	N	2252	5.74E-01	-5.49E-01	-5.70E-01	-5.28E-01	2.11E-02
ALA	30	C	447	ILE	31	O	2255	7.58E-01	-4.30E-01	-4.47E-01	-4.13E-01	1.70E-02
ALA	30	C	447	ILE	32	N	2271	6.39E-01	-4.36E-01	-4.57E-01	-4.16E-01	2.05E-02
ALA	30	C	447	ILE	32	O	2274	7.35E-01	-4.55E-01	-4.66E-01	-4.43E-01	1.17E-02
ALA	30	O	448	LYS	28	C	2215	9.13E-01	-3.60E-01	-3.86E-01	-3.34E-01	2.60E-02
ALA	30	O	448	GLY	29	C	2237	7.38E-01	-4.13E-01	-4.32E-01	-3.93E-01	1.95E-02
ALA	30	O	448	ALA	30	C	2244	6.15E-01	-5.81E-01	-5.97E-01	-5.65E-01	1.60E-02
ALA	30	O	448	ILE	31	C	2254	7.39E-01	-4.05E-01	-4.24E-01	-3.85E-01	1.97E-02
ALA	30	CB	449	ALA	30	C	2244	4.41E-01	-5.33E-01	-5.93E-01	-4.73E-01	5.98E-02
ALA	30	H	450	ALA	30	O	2245	5.56E-01	-3.73E-01	-3.86E-01	-3.60E-01	1.31E-02
ALA	30	HA	451	ALA	30	O	2245	2.96E-01	-8.17E-01	-9.73E-01	-6.62E-01	1.56E-01
ILE	31	N	455	LYS	28	C	2215	8.01E-01	-3.66E-01	-4.01E-01	-3.32E-01	3.47E-02
ILE	31	N	455	GLY	29	C	2237	6.28E-01	-4.63E-01	-4.92E-01	-4.34E-01	2.91E-02
ILE	31	N	455	ALA	30	C	2244	4.73E-01	-8.65E-01	-9.38E-01	-7.92E-01	7.32E-02
ILE	31	N	455	ILE	31	C	2254	5.66E-01	-5.64E-01	-5.81E-01	-5.46E-01	1.74E-02
ILE	31	N	455	ILE	31	HA	2261	3.61E-01	-5.25E-01	-5.51E-01	-4.99E-01	2.60E-02
ILE	31	N	455	ILE	32	C	2273	7.16E-01	-3.47E-01	-3.51E-01	-3.42E-01	4.79E-03
ILE	31	N	455	ILE	32	H	2279	4.79E-01	-4.81E-01	-4.99E-01	-4.63E-01	1.79E-02
ILE	31	C	457	ALA	30	O	2245	4.72E-01	-1.09E+00	-1.23E+00	-9.50E-01	1.40E-01
ILE	31	C	457	ILE	31	N	2252	5.56E-01	-5.86E-01	-6.04E-01	-5.67E-01	1.83E-02
ILE	31	C	457	ILE	31	O	2255	6.27E-01	-6.21E-01	-6.35E-01	-6.07E-01	1.36E-02
ILE	31	C	457	ILE	32	N	2271	4.44E-01	-1.00E+00	-1.06E+00	-9.50E-01	5.49E-02
ILE	31	C	457	ILE	32	O	2274	4.45E-01	-1.37E+00	-1.42E+00	-1.32E+00	4.95E-02
ILE	31	C	457	GLY	33	N	2290	6.55E-01	-3.43E-01	-3.50E-01	-3.36E-01	6.55E-03
ILE	31	C	457	GLY	33	O	2293	7.43E-01	-3.67E-01	-3.88E-01	-3.47E-01	2.04E-02

ILE	31	O	458	LYS	28	C	2215	9.51E-01	-3.74E-01	-3.89E-01	-3.59E-01	1.48E-02
ILE	31	O	458	GLY	29	C	2237	7.39E-01	-4.60E-01	-4.75E-01	-4.44E-01	1.51E-02
ILE	31	O	458	ALA	30	C	2244	4.68E-01	-1.23E+00	-1.34E+00	-1.11E+00	1.16E-01
ILE	31	O	458	ILE	31	C	2254	3.85E-01	-1.99E+00	-2.06E+00	-1.92E+00	6.84E-02
ILE	31	O	458	ILE	31	H	2260	5.42E-01	-4.94E-01	-5.05E-01	-4.83E-01	1.12E-02
ILE	31	O	458	ILE	31	HA	2261	2.51E-01	-2.04E+00	-2.19E+00	-1.89E+00	1.49E-01
ILE	31	O	458	ILE	32	C	2273	4.47E-01	-1.36E+00	-1.40E+00	-1.31E+00	4.73E-02
ILE	31	O	458	ILE	32	H	2279	2.36E-01	-4.63E+00	-5.08E+00	-4.17E+00	4.55E-01
ILE	31	O	458	GLY	33	C	2292	6.55E-01	-5.80E-01	-5.93E-01	-5.67E-01	1.28E-02
ILE	31	H	463	ALA	30	N	2242	5.02E-01	-3.95E-01	-4.44E-01	-3.46E-01	4.86E-02
ILE	31	H	463	ALA	30	O	2245	3.32E-01	-1.90E+00	-2.63E+00	-1.16E+00	7.31E-01
ILE	31	H	463	ILE	31	N	2252	4.29E-01	-6.32E-01	-6.61E-01	-6.02E-01	2.93E-02
ILE	31	H	463	ILE	31	O	2255	6.17E-01	-3.72E-01	-3.80E-01	-3.64E-01	7.66E-03
ILE	31	H	463	ILE	32	N	2271	5.19E-01	-3.97E-01	-4.13E-01	-3.81E-01	1.60E-02
ILE	31	H	463	ILE	32	O	2274	6.45E-01	-3.40E-01	-3.51E-01	-3.28E-01	1.15E-02
ILE	32	N	474	ALA	30	C	2244	6.70E-01	-3.97E-01	-4.15E-01	-3.79E-01	1.78E-02
ILE	32	N	474	ILE	31	C	2254	5.94E-01	-5.06E-01	-5.13E-01	-5.00E-01	6.53E-03
ILE	32	N	474	ILE	32	C	2273	5.40E-01	-6.27E-01	-6.40E-01	-6.13E-01	1.35E-02
ILE	32	N	474	ILE	32	H	2279	4.12E-01	-6.99E-01	-7.18E-01	-6.80E-01	1.93E-02
ILE	32	N	474	GLY	33	C	2292	6.79E-01	-3.93E-01	-4.06E-01	-3.80E-01	1.30E-02
ILE	32	C	476	ALA	30	O	2245	7.75E-01	-3.70E-01	-3.85E-01	-3.55E-01	1.51E-02
ILE	32	C	476	ILE	31	O	2255	8.18E-01	-3.75E-01	-3.82E-01	-3.67E-01	7.51E-03
ILE	32	C	476	ILE	32	N	2271	5.94E-01	-5.08E-01	-5.28E-01	-4.88E-01	1.98E-02
ILE	32	C	476	ILE	32	O	2274	4.08E-01	-1.71E+00	-1.80E+00	-1.62E+00	8.79E-02
ILE	32	C	476	GLY	33	N	2290	5.79E-01	-4.44E-01	-4.55E-01	-4.34E-01	1.05E-02
ILE	32	C	476	GLY	33	O	2293	5.39E-01	-7.12E-01	-7.43E-01	-6.81E-01	3.11E-02
ILE	32	O	477	ILE	31	C	2254	8.01E-01	-3.88E-01	-3.92E-01	-3.84E-01	3.77E-03
ILE	32	O	477	ILE	32	C	2273	6.27E-01	-6.21E-01	-6.31E-01	-6.11E-01	9.95E-03
ILE	32	O	477	ILE	32	H	2279	6.05E-01	-3.88E-01	-3.95E-01	-3.80E-01	7.91E-03
ILE	32	O	477	GLY	33	C	2292	6.49E-01	-5.93E-01	-6.17E-01	-5.70E-01	2.34E-02
ILE	32	H	482	ILE	32	O	2274	5.29E-01	-5.23E-01	-5.36E-01	-5.09E-01	1.34E-02
ILE	32	HA	483	ILE	32	N	2271	3.92E-01	-4.23E-01	-4.43E-01	-4.03E-01	2.01E-02
ILE	32	HA	483	ILE	32	O	2274	2.52E-01	-2.06E+00	-2.27E+00	-1.84E+00	2.18E-01
GLY	33	N	493	ILE	32	C	2273	4.79E-01	-6.97E-01	-7.70E-01	-6.24E-01	7.31E-02
GLY	33	N	493	GLY	33	C	2292	4.75E-01	-7.19E-01	-7.44E-01	-6.94E-01	2.52E-02
GLY	33	C	495	ILE	32	O	2274	5.39E-01	-9.09E-01	-1.04E+00	-7.79E-01	1.31E-01
GLY	33	C	495	GLY	33	N	2290	6.55E-01	-3.51E-01	-3.65E-01	-3.36E-01	1.46E-02
GLY	33	C	495	GLY	33	O	2293	4.37E-01	-1.22E+00	-1.37E+00	-1.08E+00	1.44E-01
GLY	33	C	495	LEU	34	N	2297	6.05E-01	-3.92E-01	-3.98E-01	-3.86E-01	5.99E-03
GLY	33	C	495	LEU	34	O	2300	8.14E-01	-3.47E-01	-3.51E-01	-3.43E-01	3.91E-03
GLY	33	C	495	MET	35	N	2316	7.02E-01	-3.22E-01	-3.28E-01	-3.16E-01	5.98E-03
GLY	33	C	495	MET	35	O	2319	7.62E-01	-3.97E-01	-4.09E-01	-3.85E-01	1.19E-02

GLY	33	O	496	ILE	32	C	2273	7.53E-01	-3.58E-01	-3.78E-01	-3.38E-01	2.00E-02
GLY	33	O	496	GLY	33	C	2292	6.10E-01	-5.51E-01	-5.60E-01	-5.42E-01	9.10E-03
GLY	33	O	496	LEU	34	C	2299	7.75E-01	-3.40E-01	-3.49E-01	-3.32E-01	8.63E-03
GLY	33	H	497	ILE	32	O	2274	3.22E-01	-1.64E+00	-2.19E+00	-1.09E+00	5.51E-01
GLY	33	H	497	GLY	33	N	2290	4.29E-01	-4.05E-01	-4.23E-01	-3.86E-01	1.84E-02
GLY	33	H	497	GLY	33	O	2293	4.07E-01	-6.34E-01	-6.89E-01	-5.79E-01	5.53E-02
LEU	34	N	500	ILE	32	C	2273	5.75E-01	-4.32E-01	-4.66E-01	-3.98E-01	3.43E-02
LEU	34	N	500	GLY	33	C	2292	4.54E-01	-7.72E-01	-8.50E-01	-6.94E-01	7.80E-02
LEU	34	N	500	LEU	34	C	2299	6.19E-01	-3.68E-01	-3.83E-01	-3.54E-01	1.45E-02
LEU	34	C	502	ILE	32	O	2274	5.99E-01	-6.90E-01	-7.09E-01	-6.71E-01	1.88E-02
LEU	34	C	502	GLY	33	O	2293	3.81E-01	-1.72E+00	-1.89E+00	-1.55E+00	1.69E-01
LEU	34	C	502	LEU	34	N	2297	5.12E-01	-5.60E-01	-5.74E-01	-5.45E-01	1.46E-02
LEU	34	C	502	LEU	34	O	2300	6.30E-01	-5.56E-01	-5.66E-01	-5.46E-01	1.00E-02
LEU	34	C	502	MET	35	N	2316	4.60E-01	-8.10E-01	-8.50E-01	-7.70E-01	4.03E-02
LEU	34	C	502	MET	35	O	2319	4.64E-01	-1.15E+00	-1.25E+00	-1.05E+00	9.73E-02
LEU	34	C	502	VAL	36	N	2333	6.82E-01	-3.84E-01	-4.02E-01	-3.66E-01	1.83E-02
LEU	34	O	503	ILE	32	C	2273	6.10E-01	-5.93E-01	-6.16E-01	-5.71E-01	2.22E-02
LEU	34	O	503	GLY	33	C	2292	3.72E-01	-2.01E+00	-2.14E+00	-1.88E+00	1.34E-01
LEU	34	O	503	LEU	34	C	2299	3.93E-01	-1.71E+00	-1.78E+00	-1.63E+00	7.30E-02
LEU	34	O	503	LEU	34	CG	2302	4.71E-01	-3.61E-01	-3.76E-01	-3.46E-01	1.52E-02
LEU	34	O	503	LEU	34	H	2305	4.96E-01	-4.36E-01	-4.51E-01	-4.20E-01	1.50E-02
LEU	34	O	503	LEU	34	HA	2306	2.48E-01	-1.48E+00	-1.53E+00	-1.42E+00	5.54E-02
LEU	34	O	503	MET	35	C	2318	4.83E-01	-1.08E+00	-1.18E+00	-9.75E-01	1.05E-01
LEU	34	O	503	MET	35	H	2324	2.73E-01	-2.42E+00	-2.86E+00	-1.97E+00	4.44E-01
LEU	34	O	503	VAL	36	H	2340	6.77E-01	-3.72E-01	-3.94E-01	-3.50E-01	2.16E-02
LEU	34	H	508	ILE	32	O	2274	3.81E-01	-9.66E-01	-1.11E+00	-8.22E-01	1.43E-01
LEU	34	H	508	GLY	33	O	2293	3.46E-01	-1.60E+00	-2.55E+00	-6.40E-01	9.55E-01
LEU	34	H	508	LEU	34	N	2297	4.56E-01	-3.44E-01	-3.75E-01	-3.12E-01	3.18E-02
MET	35	N	519	GLY	33	C	2292	5.91E-01	-4.59E-01	-4.72E-01	-4.46E-01	1.27E-02
MET	35	N	519	LEU	34	C	2299	5.88E-01	-4.56E-01	-4.63E-01	-4.49E-01	6.81E-03
MET	35	N	519	MET	35	C	2318	5.53E-01	-5.49E-01	-5.70E-01	-5.27E-01	2.16E-02
MET	35	N	519	MET	35	H	2324	4.21E-01	-4.95E-01	-5.07E-01	-4.83E-01	1.22E-02
MET	35	C	521	GLN	15	NE2	2022	8.06E-01	-5.92E-01	-6.54E-01	-5.29E-01	6.26E-02
MET	35	C	521	GLY	33	O	2293	6.71E-01	-4.69E-01	-4.85E-01	-4.54E-01	1.53E-02
MET	35	C	521	LEU	34	O	2300	8.07E-01	-3.64E-01	-3.71E-01	-3.56E-01	7.46E-03
MET	35	C	521	MET	35	N	2316	5.85E-01	-4.83E-01	-4.98E-01	-4.69E-01	1.49E-02
MET	35	C	521	MET	35	O	2319	4.01E-01	-1.71E+00	-1.74E+00	-1.69E+00	2.29E-02
MET	35	C	521	VAL	36	N	2333	5.90E-01	-5.41E-01	-5.56E-01	-5.26E-01	1.48E-02
MET	35	C	521	GLY	38	O	2359	7.83E-01	-3.52E-01	-3.73E-01	-3.31E-01	2.13E-02
MET	35	O	522	GLY	33	C	2292	8.47E-01	-3.29E-01	-3.34E-01	-3.23E-01	5.12E-03
MET	35	O	522	LEU	34	C	2299	7.97E-01	-3.60E-01	-3.67E-01	-3.54E-01	6.18E-03
MET	35	O	522	MET	35	C	2318	6.38E-01	-5.76E-01	-5.87E-01	-5.65E-01	1.07E-02

MET	35	CG	524	MET	35	C	2318	4.43E-01	-4.95E-01	-5.35E-01	-4.54E-01	4.03E-02
MET	35	CE	526	GLY	38	C	2358	4.45E-01	-6.72E-01	-7.82E-01	-5.61E-01	1.10E-01
MET	35	CE	526	VAL	40	H	2387	3.73E-01	-9.59E-01	-1.28E+00	-6.40E-01	3.20E-01
MET	35	H	527	MET	35	O	2319	5.23E-01	-4.17E-01	-4.30E-01	-4.04E-01	1.28E-02
MET	35	HA	528	MET	35	O	2319	2.48E-01	-1.35E+00	-1.43E+00	-1.27E+00	7.92E-02
MET	35	HE1	533	GLY	38	O	2359	3.65E-01	-4.45E-01	-5.24E-01	-3.65E-01	7.94E-02
MET	35	HE2	534	GLY	38	O	2359	3.13E-01	-6.59E-01	-7.38E-01	-5.79E-01	7.94E-02
MET	35	HE3	535	GLY	38	O	2359	3.66E-01	-4.37E-01	-5.06E-01	-3.67E-01	6.99E-02
VAL	36	N	536	GLN	15	CD	2020	7.47E-01	-3.93E-01	-4.53E-01	-3.32E-01	6.05E-02
VAL	36	N	536	LEU	34	C	2299	7.14E-01	-3.51E-01	-3.65E-01	-3.37E-01	1.40E-02
VAL	36	N	536	MET	35	C	2318	4.70E-01	-9.16E-01	-9.51E-01	-8.80E-01	3.58E-02
VAL	36	N	536	MET	35	H	2324	5.17E-01	-3.43E-01	-3.66E-01	-3.20E-01	2.32E-02
VAL	36	N	536	VAL	36	C	2335	5.78E-01	-4.23E-01	-4.43E-01	-4.04E-01	1.92E-02
VAL	36	N	536	VAL	36	CB	2337	4.89E-01	-5.54E-01	-6.00E-01	-5.08E-01	4.62E-02
VAL	36	N	536	VAL	36	H	2340	6.09E-01	-3.72E-01	-3.79E-01	-3.64E-01	7.56E-03
VAL	36	C	538	GLN	15	OE1	2021	5.79E-01	-8.10E-01	-1.15E+00	-4.69E-01	3.40E-01
VAL	36	C	538	GLN	15	NE2	2022	5.22E-01	-1.24E+00	-1.62E+00	-8.59E-01	3.78E-01
VAL	36	C	538	MET	35	O	2319	4.92E-01	-7.79E-01	-8.51E-01	-7.07E-01	7.21E-02
VAL	36	C	538	VAL	36	N	2333	5.55E-01	-4.62E-01	-4.78E-01	-4.47E-01	1.53E-02
VAL	36	C	538	VAL	36	O	2336	6.32E-01	-3.14E-01	-3.18E-01	-3.09E-01	4.47E-03
VAL	36	C	538	GLY	37	N	2349	4.63E-01	-5.91E-01	-6.25E-01	-5.58E-01	3.38E-02
VAL	36	C	538	GLY	38	O	2359	6.02E-01	-4.51E-01	-5.15E-01	-3.87E-01	6.39E-02
VAL	36	O	539	GLN	15	CD	2020	5.41E-01	-7.67E-01	-1.00E+00	-5.32E-01	2.36E-01
VAL	36	O	539	GLN	15	HE22	2030	3.85E-01	-1.52E+00	-2.33E+00	-7.11E-01	8.11E-01
VAL	36	O	539	MET	35	C	2318	4.74E-01	-8.12E-01	-8.72E-01	-7.51E-01	6.04E-02
VAL	36	O	539	VAL	36	C	2335	3.96E-01	-9.51E-01	-9.91E-01	-9.11E-01	3.99E-02
VAL	36	O	539	VAL	36	CB	2337	3.90E-01	-9.19E-01	-1.08E+00	-7.54E-01	1.65E-01
VAL	36	O	539	VAL	36	H	2340	5.25E-01	-4.64E-01	-4.74E-01	-4.53E-01	1.03E-02
VAL	36	O	539	GLY	37	C	2351	5.44E-01	-5.70E-01	-6.10E-01	-5.29E-01	4.07E-02
VAL	36	O	539	GLY	37	H	2353	2.75E-01	-1.60E+00	-1.95E+00	-1.25E+00	3.49E-01
VAL	36	O	539	GLY	38	C	2358	6.10E-01	-4.51E-01	-5.06E-01	-3.96E-01	5.50E-02
VAL	36	O	539	GLY	38	H	2360	4.41E-01	-4.18E-01	-4.66E-01	-3.70E-01	4.81E-02
VAL	36	CB	540	GLN	15	NE2	2022	6.17E-01	-6.89E-01	-8.23E-01	-5.54E-01	1.35E-01
VAL	36	CB	540	MET	35	O	2319	5.26E-01	-5.92E-01	-6.57E-01	-5.26E-01	6.56E-02
VAL	36	H	543	GLN	15	OE1	2021	6.77E-01	-4.41E-01	-5.16E-01	-3.67E-01	7.43E-02
VAL	36	H	543	GLN	15	NE2	2022	6.28E-01	-7.15E-01	-8.23E-01	-6.07E-01	1.08E-01
VAL	36	H	543	MET	35	N	2316	5.20E-01	-4.67E-01	-5.03E-01	-4.30E-01	3.65E-02
VAL	36	H	543	MET	35	O	2319	2.79E-01	-3.58E+00	-4.16E+00	-3.00E+00	5.80E-01
VAL	36	H	543	VAL	36	N	2333	4.19E-01	-8.91E-01	-9.02E-01	-8.80E-01	1.12E-02
VAL	36	H	543	VAL	36	O	2336	5.94E-01	-3.53E-01	-3.64E-01	-3.41E-01	1.14E-02
VAL	36	H	543	GLY	37	N	2349	5.07E-01	-4.71E-01	-5.14E-01	-4.28E-01	4.31E-02
VAL	36	H	543	GLY	38	O	2359	6.37E-01	-3.87E-01	-4.21E-01	-3.52E-01	3.46E-02

GLY	37	N	552	GLN	15	CD	2020	5.92E-01	-6.19E-01	-8.39E-01	-3.99E-01	2.20E-01
GLY	37	N	552	VAL	36	C	2335	5.86E-01	-3.40E-01	-3.45E-01	-3.34E-01	5.86E-03
GLY	37	N	552	GLY	37	C	2351	6.78E-01	-3.27E-01	-3.39E-01	-3.15E-01	1.21E-02
GLY	37	N	552	GLY	37	H	2353	4.18E-01	-4.31E-01	-4.40E-01	-4.21E-01	9.76E-03
GLY	37	C	554	HIS	13	ND1	1986	5.82E-01	-5.35E-01	-6.27E-01	-4.44E-01	9.12E-02
GLY	37	C	554	GLN	15	OE1	2021	6.14E-01	-7.96E-01	-1.01E+00	-5.79E-01	2.17E-01
GLY	37	C	554	GLN	15	NE2	2022	5.27E-01	-1.59E+00	-2.07E+00	-1.12E+00	4.79E-01
GLY	37	C	554	MET	35	O	2319	6.76E-01	-5.00E-01	-5.31E-01	-4.69E-01	3.11E-02
GLY	37	C	554	VAL	36	N	2333	6.59E-01	-4.18E-01	-4.37E-01	-3.98E-01	1.94E-02
GLY	37	C	554	VAL	36	O	2336	6.29E-01	-4.12E-01	-4.27E-01	-3.97E-01	1.50E-02
GLY	37	C	554	GLY	37	N	2349	4.32E-01	-9.21E-01	-1.00E+00	-8.41E-01	7.97E-02
GLY	37	C	554	GLY	37	O	2352	6.35E-01	-5.07E-01	-5.13E-01	-5.01E-01	5.86E-03
GLY	37	C	554	GLY	38	N	2356	4.44E-01	-8.51E-01	-9.04E-01	-7.98E-01	5.30E-02
GLY	37	C	554	GLY	38	O	2359	4.26E-01	-1.37E+00	-1.68E+00	-1.07E+00	3.06E-01
GLY	37	C	554	VAL	39	N	2363	6.33E-01	-4.65E-01	-5.23E-01	-4.06E-01	5.86E-02
GLY	37	O	555	HIS	13	C	1982	8.20E-01	-3.61E-01	-3.91E-01	-3.31E-01	2.96E-02
GLY	37	O	555	HIS	14	C	1999	8.40E-01	-3.44E-01	-3.62E-01	-3.26E-01	1.79E-02
GLY	37	O	555	GLN	15	CD	2020	5.48E-01	-8.86E-01	-1.09E+00	-6.84E-01	2.02E-01
GLY	37	O	555	GLN	15	HE22	2030	4.38E-01	-1.12E+00	-1.52E+00	-7.14E-01	4.06E-01
GLY	37	O	555	MET	35	C	2318	6.61E-01	-4.84E-01	-5.08E-01	-4.61E-01	2.36E-02
GLY	37	O	555	VAL	36	C	2335	4.36E-01	-9.44E-01	-1.04E+00	-8.44E-01	9.99E-02
GLY	37	O	555	VAL	36	CB	2337	5.68E-01	-4.51E-01	-5.08E-01	-3.94E-01	5.70E-02
GLY	37	O	555	VAL	36	H	2340	6.32E-01	-3.89E-01	-4.05E-01	-3.72E-01	1.66E-02
GLY	37	O	555	GLY	37	C	2351	3.91E-01	-1.60E+00	-1.63E+00	-1.56E+00	3.38E-02
GLY	37	O	555	GLY	37	H	2353	2.87E-01	-1.88E+00	-2.41E+00	-1.34E+00	5.35E-01
GLY	37	O	555	GLY	38	C	2358	4.25E-01	-1.41E+00	-1.78E+00	-1.04E+00	3.67E-01
GLY	37	O	555	GLY	38	H	2360	2.30E-01	-3.25E+00	-3.76E+00	-2.73E+00	5.17E-01
GLY	37	O	555	VAL	39	H	2370	6.15E-01	-4.24E-01	-4.84E-01	-3.65E-01	5.93E-02
GLY	38	N	559	GLY	37	C	2351	6.04E-01	-4.15E-01	-4.23E-01	-4.06E-01	8.87E-03
GLY	38	N	559	GLY	38	C	2358	5.27E-01	-5.69E-01	-6.26E-01	-5.12E-01	5.71E-02
GLY	38	N	559	GLY	38	H	2360	4.18E-01	-4.32E-01	-4.40E-01	-4.23E-01	8.82E-03
GLY	38	C	561	GLY	38	N	2356	6.11E-01	-4.06E-01	-4.26E-01	-3.86E-01	2.00E-02
GLY	38	C	561	GLY	38	O	2359	4.18E-01	-1.35E+00	-1.43E+00	-1.26E+00	8.49E-02
GLY	38	C	561	VAL	39	N	2363	5.69E-01	-5.68E-01	-5.96E-01	-5.40E-01	2.81E-02
GLY	38	C	561	VAL	40	N	2379	6.37E-01	-4.70E-01	-5.44E-01	-3.95E-01	7.48E-02
GLY	38	O	562	GLY	38	C	2358	6.24E-01	-5.25E-01	-5.34E-01	-5.16E-01	8.80E-03
VAL	39	N	566	GLY	38	C	2358	4.99E-01	-7.69E-01	-8.04E-01	-7.34E-01	3.54E-02
VAL	39	N	566	VAL	39	C	2365	5.56E-01	-4.77E-01	-5.45E-01	-4.10E-01	6.76E-02
VAL	39	N	566	VAL	39	CB	2367	4.62E-01	-6.39E-01	-7.10E-01	-5.69E-01	7.02E-02
VAL	39	N	566	VAL	39	H	2370	6.05E-01	-3.76E-01	-3.82E-01	-3.70E-01	5.68E-03
VAL	39	N	566	VAL	40	H	2387	4.65E-01	-8.19E-01	-1.07E+00	-5.64E-01	2.55E-01
VAL	39	C	568	GLY	38	O	2359	5.42E-01	-5.70E-01	-6.47E-01	-4.94E-01	7.63E-02

VAL	39	C	568	VAL	39	N	2363	5.88E-01	-4.12E-01	-4.47E-01	-3.77E-01	3.54E-02
VAL	39	C	568	VAL	40	N	2379	4.51E-01	-7.76E-01	-8.81E-01	-6.70E-01	1.06E-01
VAL	39	O	569	GLY	38	C	2358	5.21E-01	-6.57E-01	-7.66E-01	-5.49E-01	1.08E-01
VAL	39	O	569	VAL	39	C	2365	4.00E-01	-9.21E-01	-9.45E-01	-8.97E-01	2.38E-02
VAL	39	O	569	VAL	39	CB	2367	3.86E-01	-1.05E+00	-1.32E+00	-7.76E-01	2.73E-01
VAL	39	O	569	VAL	39	H	2370	5.63E-01	-4.05E-01	-4.50E-01	-3.60E-01	4.47E-02
VAL	39	O	569	VAL	40	C	2381	5.25E-01	-5.18E-01	-6.38E-01	-3.97E-01	1.21E-01
VAL	39	O	569	VAL	40	CB	2383	5.38E-01	-4.19E-01	-4.97E-01	-3.41E-01	7.80E-02
VAL	39	O	569	VAL	40	H	2387	2.49E-01	-4.13E+00	-5.49E+00	-2.76E+00	1.37E+00
VAL	39	H	573	GLY	38	O	2359	3.63E-01	-1.58E+00	-1.86E+00	-1.29E+00	2.87E-01
VAL	39	H	573	VAL	39	N	2363	4.38E-01	-8.01E-01	-8.45E-01	-7.56E-01	4.45E-02
VAL	39	H	573	VAL	40	N	2379	4.89E-01	-7.38E-01	-9.88E-01	-4.88E-01	2.50E-01
VAL	40	N	582	VAL	39	C	2365	6.06E-01	-3.81E-01	-3.91E-01	-3.70E-01	1.07E-02
VAL	40	N	582	VAL	40	H	2387	4.26E-01	-8.58E-01	-8.73E-01	-8.42E-01	1.54E-02
VAL	40	C	584	VAL	40	N	2379	4.51E-01	-7.82E-01	-8.84E-01	-6.79E-01	1.03E-01
VAL	40	C	584	VAL	40	OXT	2386	5.11E-01	-5.29E-01	-6.08E-01	-4.51E-01	7.86E-02
VAL	40	O	585	VAL	40	C	2381	4.52E-01	-7.50E-01	-9.02E-01	-5.99E-01	1.52E-01
VAL	40	O	585	VAL	40	H	2387	3.42E-01	-2.86E+00	-4.11E+00	-1.60E+00	1.25E+00
VAL	40	CB	586	VAL	40	N	2379	4.36E-01	-7.52E-01	-8.58E-01	-6.46E-01	1.06E-01
VAL	40	OXT	589	VAL	40	C	2381	5.38E-01	-5.05E-01	-6.49E-01	-3.61E-01	1.44E-01
VAL	40	OXT	589	VAL	40	H	2387	4.28E-01	-7.96E-01	-9.23E-01	-6.70E-01	1.27E-01
VAL	40	H	590	VAL	40	N	2379	6.13E-01	-3.66E-01	-3.77E-01	-3.55E-01	1.08E-02
VAL	40	HB	592	VAL	40	H	2387	2.71E-01	-9.34E-01	-1.24E+00	-6.32E-01	3.02E-01

Supplementary Table 1b: Mapping results for Aβ40's (PDB ID: 2M4J) short range (1:2) dominant atom-atom Lennard-Jones interactions across ensemble structures. Columns for each chain correspond to: residue abbreviation, residue number in peptide sequence, atom identity (IUPAC naming convention) and atom number in PDB file. Energy in *kT*, distance in *nm*. Mapping analysis began on the 11th residue for both isoforms because original structure data for Aβ42 begins with the 11th residue.

Chain A				Chain D				Average Distance	Average L-J Values	Lower 95% Confidence Interval Bound	Upper 95% Confidence Interval Bound	Margin of Error
GLU	11	C	154	VAL	12	N	1964	4.47E-01	-1.10E-01	-1.12E-01	-1.08E-01	1.72E-03
GLU	11	O	155	GLU	11	CA	1950	3.72E-01	-2.33E-01	-2.36E-01	-2.31E-01	2.82E-03
GLU	11	O	155	GLU	11	C	1951	3.99E-01	-1.67E-01	-1.70E-01	-1.65E-01	2.59E-03
GLU	11	O	155	VAL	12	N	1964	3.43E-01	-3.03E-01	-3.05E-01	-3.01E-01	1.75E-03
GLU	11	O	155	VAL	12	CA	1965	4.44E-01	-1.15E-01	-1.18E-01	-1.13E-01	2.65E-03
GLU	11	O	155	VAL	12	O	1967	4.04E-01	-1.79E-01	-1.83E-01	-1.76E-01	3.39E-03
GLU	11	O	155	VAL	12	CG1	1969	3.49E-01	-2.37E-01	-2.44E-01	-2.29E-01	7.04E-03
VAL	12	N	167	VAL	12	O	1967	4.34E-01	-1.43E-01	-1.45E-01	-1.41E-01	1.94E-03
VAL	12	CA	168	VAL	12	O	1967	3.41E-01	-2.22E-01	-2.25E-01	-2.19E-01	2.98E-03
VAL	12	CA	168	VAL	12	CG1	1969	3.95E-01	-1.71E-01	-1.72E-01	-1.70E-01	9.92E-04
VAL	12	C	169	VAL	12	O	1967	3.82E-01	-1.93E-01	-1.95E-01	-1.92E-01	1.48E-03
VAL	12	CB	171	VAL	12	O	1967	4.25E-01	-1.42E-01	-1.43E-01	-1.41E-01	1.47E-03
VAL	12	CB	171	VAL	12	CG1	1969	3.72E-01	-1.73E-01	-1.74E-01	-1.72E-01	8.52E-04
VAL	12	CG2	173	VAL	12	O	1967	4.32E-01	-1.32E-01	-1.34E-01	-1.31E-01	1.58E-03
VAL	12	CG2	173	VAL	12	CG1	1969	3.95E-01	-1.71E-01	-1.72E-01	-1.71E-01	7.36E-04
HIS	13	N	183	VAL	12	C	1966	4.46E-01	-1.11E-01	-1.13E-01	-1.10E-01	1.44E-03
HIS	13	N	183	VAL	12	O	1967	3.28E-01	-2.43E-01	-2.52E-01	-2.35E-01	8.49E-03
HIS	13	N	183	HIS	13	ND1	1986	4.49E-01	-1.36E-01	-1.39E-01	-1.33E-01	3.06E-03
HIS	13	CA	184	VAL	12	O	1967	4.44E-01	-1.15E-01	-1.17E-01	-1.13E-01	1.86E-03
HIS	13	C	185	HIS	14	N	1997	4.34E-01	-1.26E-01	-1.28E-01	-1.25E-01	1.41E-03
HIS	13	O	186	VAL	12	O	1967	4.08E-01	-1.71E-01	-1.74E-01	-1.68E-01	3.16E-03
HIS	13	O	186	HIS	13	CA	1981	3.65E-01	-2.42E-01	-2.44E-01	-2.41E-01	1.27E-03
HIS	13	O	186	HIS	13	C	1982	3.84E-01	-1.90E-01	-1.91E-01	-1.88E-01	1.61E-03
HIS	13	O	186	HIS	13	CB	1984	4.48E-01	-1.10E-01	-1.13E-01	-1.07E-01	2.76E-03
HIS	13	O	186	HIS	14	CA	1998	4.21E-01	-1.48E-01	-1.51E-01	-1.46E-01	2.57E-03
HIS	13	O	186	HIS	14	C	1999	4.19E-01	-1.35E-01	-1.39E-01	-1.31E-01	3.98E-03
HIS	13	O	186	HIS	14	O	2000	3.88E-01	-2.16E-01	-2.22E-01	-2.11E-01	5.69E-03
HIS	13	CB	187	HIS	13	ND1	1986	3.84E-01	-2.16E-01	-2.17E-01	-2.15E-01	1.34E-03
HIS	13	CG	188	HIS	13	ND1	1986	3.96E-01	-1.78E-01	-1.80E-01	-1.76E-01	1.84E-03
HIS	13	CG	188	HIS	13	CE1	1988	4.11E-01	-1.22E-01	-1.23E-01	-1.20E-01	1.55E-03
HIS	13	CD2	190	HIS	13	ND1	1986	3.67E-01	-1.94E-01	-1.95E-01	-1.92E-01	1.57E-03
HIS	13	CD2	190	HIS	13	NE2	1989	4.38E-01	-1.21E-01	-1.23E-01	-1.19E-01	2.19E-03
HIS	13	NE2	192	HIS	13	CE1	1988	4.20E-01	-1.46E-01	-1.48E-01	-1.43E-01	2.73E-03
HIS	14	N	200	HIS	14	O	2000	4.40E-01	-1.33E-01	-1.36E-01	-1.31E-01	2.26E-03
HIS	14	CA	201	HIS	14	C	1999	4.19E-01	-1.28E-01	-1.31E-01	-1.26E-01	2.37E-03
HIS	14	CA	201	HIS	14	O	2000	3.29E-01	-1.50E-01	-1.61E-01	-1.38E-01	1.14E-02

HIS	14	C	202	HIS	14	O	2000	4.13E-01	-1.44E-01	-1.46E-01	-1.43E-01	1.30E-03
HIS	14	CG	205	HIS	14	C	1999	4.06E-01	-1.26E-01	-1.30E-01	-1.22E-01	3.57E-03
HIS	14	ND1	206	HIS	14	C	1999	3.75E-01	-1.95E-01	-1.96E-01	-1.93E-01	1.64E-03
HIS	14	CD2	207	HIS	14	O	2000	3.42E-01	-1.94E-01	-2.03E-01	-1.84E-01	9.61E-03
HIS	14	CE1	208	HIS	14	C	1999	4.17E-01	-1.15E-01	-1.20E-01	-1.11E-01	4.22E-03
HIS	14	NE2	209	HIS	14	O	2000	3.61E-01	-2.93E-01	-3.00E-01	-2.86E-01	7.25E-03
GLN	15	N	217	HIS	14	O	2000	4.26E-01	-1.56E-01	-1.62E-01	-1.50E-01	5.59E-03
GLN	15	N	217	GLN	15	CA	2015	4.35E-01	-1.42E-01	-1.46E-01	-1.39E-01	3.44E-03
GLN	15	N	217	GLN	15	CB	2018	4.44E-01	-1.29E-01	-1.39E-01	-1.19E-01	1.00E-02
GLN	15	O	220	GLN	15	CA	2015	3.72E-01	-2.33E-01	-2.41E-01	-2.24E-01	8.39E-03
GLN	15	O	220	GLN	15	C	2016	4.04E-01	-1.59E-01	-1.64E-01	-1.54E-01	5.02E-03
GLN	15	O	220	GLN	15	CB	2018	4.15E-01	-1.60E-01	-1.78E-01	-1.42E-01	1.81E-02
GLN	15	O	220	LYS	16	N	2031	3.60E-01	-2.94E-01	-3.00E-01	-2.87E-01	6.59E-03
LYS	16	N	234	LYS	16	O	2034	4.49E-01	-1.20E-01	-1.28E-01	-1.12E-01	8.07E-03
LYS	16	CA	235	LYS	16	O	2034	3.42E-01	-2.20E-01	-2.30E-01	-2.09E-01	1.04E-02
LYS	16	C	236	LYS	16	O	2034	3.98E-01	-1.68E-01	-1.70E-01	-1.67E-01	1.44E-03
LYS	16	CB	238	LYS	16	O	2034	3.95E-01	-1.95E-01	-2.01E-01	-1.89E-01	5.97E-03
LYS	16	CG	239	LYS	16	O	2034	3.57E-01	-2.43E-01	-2.46E-01	-2.41E-01	2.26E-03
LEU	17	N	256	LYS	16	O	2034	3.74E-01	-2.70E-01	-2.74E-01	-2.67E-01	3.56E-03
LEU	17	N	256	LEU	17	CA	2054	4.54E-01	-1.16E-01	-1.20E-01	-1.11E-01	4.57E-03
LEU	17	N	256	LEU	17	CG	2058	4.03E-01	-1.75E-01	-2.08E-01	-1.42E-01	3.28E-02
LEU	17	O	259	LYS	16	O	2034	4.00E-01	-1.93E-01	-2.25E-01	-1.60E-01	3.27E-02
LEU	17	O	259	LEU	17	N	2053	4.52E-01	-1.16E-01	-1.18E-01	-1.14E-01	1.98E-03
LEU	17	O	259	LEU	17	C	2055	3.86E-01	-1.88E-01	-1.94E-01	-1.81E-01	6.48E-03
LEU	17	O	259	LEU	17	CB	2057	3.86E-01	-2.08E-01	-2.37E-01	-1.78E-01	2.94E-02
LEU	17	O	259	VAL	18	N	2072	3.36E-01	-2.51E-01	-2.73E-01	-2.29E-01	2.17E-02
VAL	18	CA	276	VAL	18	N	2072	4.58E-01	-1.11E-01	-1.13E-01	-1.09E-01	2.14E-03
VAL	18	CA	276	VAL	18	O	2075	4.08E-01	-1.71E-01	-1.82E-01	-1.60E-01	1.12E-02
VAL	18	C	277	VAL	18	O	2075	3.84E-01	-1.91E-01	-1.94E-01	-1.89E-01	2.72E-03
PHE	19	N	291	VAL	18	C	2074	4.01E-01	-1.72E-01	-1.73E-01	-1.71E-01	1.01E-03
PHE	19	CA	292	VAL	18	O	2075	3.52E-01	-2.40E-01	-2.42E-01	-2.38E-01	2.02E-03
PHE	19	C	293	VAL	18	O	2075	3.94E-01	-1.76E-01	-2.01E-01	-1.50E-01	2.53E-02
PHE	19	C	293	PHE	20	N	2108	4.37E-01	-1.24E-01	-1.32E-01	-1.16E-01	7.70E-03
PHE	19	O	294	VAL	18	O	2075	3.48E-01	-2.72E-01	-3.18E-01	-2.26E-01	4.61E-02
PHE	19	O	294	PHE	20	CA	2109	4.20E-01	-1.51E-01	-1.57E-01	-1.45E-01	6.17E-03
PHE	19	CB	295	VAL	18	O	2075	3.38E-01	-2.05E-01	-2.18E-01	-1.92E-01	1.32E-02
PHE	19	CB	295	PHE	19	CA	2089	4.34E-01	-1.26E-01	-1.45E-01	-1.07E-01	1.87E-02
PHE	20	C	313	ALA	21	N	2128	4.18E-01	-1.49E-01	-1.57E-01	-1.41E-01	7.59E-03
PHE	20	O	314	ALA	21	CA	2129	3.88E-01	-1.61E-01	-1.66E-01	-1.56E-01	4.74E-03
PHE	20	O	314	ALA	21	CB	2132	3.98E-01	-1.88E-01	-2.08E-01	-1.68E-01	2.00E-02
PHE	20	O	314	GLU	22	N	2138	4.56E-01	-1.11E-01	-1.14E-01	-1.07E-01	3.00E-03
PHE	20	CD2	318	PHE	20	CD1	2114	4.17E-01	-1.16E-01	-1.18E-01	-1.14E-01	2.13E-03

PHE	20	CE2	320	PHE	20	CE1	2116	4.18E-01	-1.15E-01	-1.17E-01	-1.12E-01	2.49E-03
ALA	21	CA	332	ALA	21	CB	2132	4.18E-01	-1.45E-01	-1.58E-01	-1.33E-01	1.24E-02
ALA	21	C	333	ALA	21	CB	2132	4.10E-01	-1.36E-01	-1.44E-01	-1.27E-01	8.45E-03
ALA	21	O	334	ALA	21	CA	2129	4.04E-01	-1.79E-01	-1.86E-01	-1.71E-01	7.21E-03
ALA	21	O	334	ALA	21	CB	2132	3.49E-01	-2.37E-01	-2.44E-01	-2.30E-01	7.21E-03
GLU	22	CA	342	GLU	22	O	2141	3.52E-01	-2.43E-01	-2.45E-01	-2.41E-01	2.03E-03
GLU	22	C	343	GLU	22	O	2141	3.81E-01	-1.95E-01	-1.97E-01	-1.93E-01	1.82E-03
ASP	23	N	356	GLU	22	C	2140	4.33E-01	-1.28E-01	-1.29E-01	-1.26E-01	1.69E-03
ASP	23	CA	357	GLU	22	O	2141	4.20E-01	-1.51E-01	-1.54E-01	-1.47E-01	3.39E-03
ASP	23	O	359	GLU	22	O	2141	3.57E-01	-2.95E-01	-3.25E-01	-2.64E-01	3.03E-02
ASP	23	O	359	ASP	23	N	2153	4.15E-01	-1.80E-01	-1.98E-01	-1.62E-01	1.81E-02
ASP	23	O	359	ASP	23	C	2155	3.94E-01	-1.75E-01	-1.84E-01	-1.66E-01	8.81E-03
ASP	23	O	359	VAL	24	N	2165	3.79E-01	-1.99E-01	-2.08E-01	-1.90E-01	8.96E-03
VAL	24	CA	369	VAL	24	CG2	2171	4.29E-01	-1.30E-01	-1.48E-01	-1.11E-01	1.88E-02
VAL	24	CG1	373	VAL	24	CG2	2171	4.14E-01	-1.50E-01	-1.64E-01	-1.35E-01	1.45E-02
GLY	25	CA	385	SER	26	N	2188	4.41E-01	-1.35E-01	-1.55E-01	-1.16E-01	1.92E-02
GLY	25	C	386	SER	26	CA	2189	4.35E-01	-1.11E-01	-1.14E-01	-1.08E-01	3.30E-03
SER	26	N	391	SER	26	CB	2192	4.10E-01	-1.81E-01	-1.86E-01	-1.76E-01	5.18E-03
SER	26	C	393	SER	26	CB	2192	4.12E-01	-1.35E-01	-1.45E-01	-1.26E-01	9.38E-03
SER	26	O	394	SER	26	CB	2192	3.98E-01	-1.52E-01	-1.69E-01	-1.36E-01	1.65E-02
SER	26	O	394	ASN	27	O	2202	4.19E-01	-1.51E-01	-1.66E-01	-1.35E-01	1.56E-02
ASN	27	CA	403	ASN	27	O	2202	3.63E-01	-1.59E-01	-1.79E-01	-1.40E-01	1.98E-02
ASN	27	C	404	ASN	27	O	2202	4.14E-01	-1.42E-01	-1.53E-01	-1.32E-01	1.06E-02
LYS	28	C	418	GLY	29	N	2235	4.16E-01	-1.51E-01	-1.59E-01	-1.43E-01	8.01E-03
LYS	28	O	419	LYS	28	CA	2214	4.15E-01	-1.59E-01	-1.95E-01	-1.23E-01	3.58E-02
LYS	28	O	419	LYS	28	C	2215	4.01E-01	-1.62E-01	-1.76E-01	-1.49E-01	1.33E-02
LYS	28	O	419	GLY	29	CA	2236	3.62E-01	-1.84E-01	-2.13E-01	-1.55E-01	2.87E-02
ALA	30	CA	446	ALA	30	O	2245	3.59E-01	-2.41E-01	-2.44E-01	-2.39E-01	2.60E-03
ALA	30	C	447	ALA	30	O	2245	4.09E-01	-1.52E-01	-1.67E-01	-1.37E-01	1.46E-02
ILE	31	N	455	ALA	30	O	2245	3.88E-01	-2.21E-01	-2.64E-01	-1.79E-01	4.21E-02
ILE	31	O	458	ALA	30	O	2245	3.96E-01	-2.06E-01	-2.61E-01	-1.52E-01	5.46E-02
ILE	31	O	458	ILE	31	CA	2253	3.44E-01	-2.18E-01	-2.28E-01	-2.08E-01	1.01E-02
ILE	31	O	458	ILE	31	C	2254	3.85E-01	-1.89E-01	-1.96E-01	-1.82E-01	6.91E-03
ILE	31	O	458	ILE	32	O	2274	3.72E-01	-2.59E-01	-2.79E-01	-2.38E-01	2.03E-02
ILE	32	N	474	ILE	32	O	2274	4.40E-01	-1.35E-01	-1.44E-01	-1.25E-01	9.40E-03
ILE	32	CA	475	ILE	32	O	2274	3.45E-01	-2.09E-01	-2.18E-01	-1.99E-01	9.34E-03
ILE	32	C	476	ILE	32	O	2274	4.08E-01	-1.52E-01	-1.64E-01	-1.40E-01	1.17E-02
LEU	34	C	502	GLY	33	O	2293	3.81E-01	-1.89E-01	-2.07E-01	-1.71E-01	1.77E-02
LEU	34	O	503	GLY	33	C	2292	3.72E-01	-2.02E-01	-2.12E-01	-1.93E-01	9.36E-03
LEU	34	O	503	LEU	34	N	2297	4.05E-01	-2.00E-01	-2.13E-01	-1.87E-01	1.33E-02
LEU	34	O	503	LEU	34	CA	2298	3.44E-01	-2.28E-01	-2.34E-01	-2.21E-01	6.74E-03
LEU	34	O	503	LEU	34	C	2299	3.93E-01	-1.76E-01	-1.85E-01	-1.67E-01	9.23E-03

LEU	34	O	503	MET	35	N	2316	3.51E-01	-2.66E-01	-2.91E-01	-2.42E-01	2.45E-02
MET	35	CA	520	MET	35	O	2319	3.43E-01	-2.09E-01	-2.26E-01	-1.92E-01	1.73E-02
MET	35	C	521	MET	35	O	2319	4.01E-01	-1.63E-01	-1.66E-01	-1.60E-01	3.12E-03
MET	35	CB	523	MET	35	O	2319	3.99E-01	-1.68E-01	-1.93E-01	-1.42E-01	2.53E-02
MET	35	CG	524	MET	35	O	2319	3.69E-01	-2.04E-01	-2.21E-01	-1.87E-01	1.69E-02
MET	35	SD	525	MET	35	SD	2322	5.08E-01	-1.71E-01	-1.78E-01	-1.63E-01	7.43E-03
VAL	36	N	536	MET	35	O	2319	3.61E-01	-2.80E-01	-2.89E-01	-2.70E-01	9.55E-03
VAL	36	O	539	VAL	36	C	2335	3.96E-01	-1.72E-01	-1.81E-01	-1.63E-01	9.23E-03
VAL	36	O	539	VAL	36	CB	2337	3.90E-01	-1.80E-01	-2.08E-01	-1.52E-01	2.81E-02
VAL	36	O	539	GLY	37	N	2349	3.55E-01	-2.53E-01	-2.81E-01	-2.25E-01	2.78E-02
GLY	37	O	555	GLY	37	CA	2350	3.62E-01	-2.20E-01	-2.39E-01	-2.01E-01	1.89E-02
GLY	37	O	555	GLY	37	C	2351	3.91E-01	-1.79E-01	-1.84E-01	-1.75E-01	4.62E-03
GLY	38	C	561	GLY	38	O	2359	4.18E-01	-1.37E-01	-1.51E-01	-1.23E-01	1.41E-02
VAL	39	O	569	VAL	39	C	2365	4.00E-01	-1.65E-01	-1.71E-01	-1.59E-01	6.07E-03
VAL	39	CG2	572	VAL	39	CB	2367	4.04E-01	-1.53E-01	-1.60E-01	-1.45E-01	7.82E-03

Supplementary Table 2a: Mapping results for Aβ42's (PDB ID: 2MXU, by Xiao et al.) short range (1:2) dominant atom-atom Coulombic interactions across ensemble structures. Columns for each chain correspond to: residue abbreviation, residue number in peptide sequence, atom identity (IUPAC naming convention) and atom number in PDB file. Energy in *kT*, distance in *nm*. Mapping analysis began on the 11th residue for both isoforms because original structure data for Aβ42 begins with the 11th residue.

Chain A				Chain B				Average Distance	Average Coulombic Values	Lower 95% Confidence Interval Bound	Upper 95% Confidence Interval Bound	Margin of Error
GLU	11	N	1	GLU	11	C	480	5.26E-01	-5.80E-01	-7.48E-01	-4.12E-01	1.68E-01
GLU	11	N	1	GLU	11	CD	484	6.60E-01	-5.24E-01	-5.80E-01	-4.69E-01	5.58E-02
GLU	11	C	3	GLU	11	O	481	5.12E-01	-1.01E+00	-1.40E+00	-6.21E-01	3.90E-01
GLU	11	C	3	GLU	11	OE1	485	7.36E-01	-5.11E-01	-5.73E-01	-4.49E-01	6.18E-02
GLU	11	C	3	GLU	11	OE2	486	8.05E-01	-4.28E-01	-4.58E-01	-3.98E-01	2.99E-02
GLU	11	C	3	VAL	12	N	493	4.82E-01	-7.58E-01	-9.29E-01	-5.87E-01	1.71E-01
GLU	11	C	3	HIS	13	N	509	6.56E-01	-3.99E-01	-4.08E-01	-3.89E-01	9.19E-03
GLU	11	O	4	GLU	11	C	480	4.67E-01	-1.25E+00	-1.65E+00	-8.59E-01	3.94E-01
GLU	11	O	4	GLU	11	CD	484	7.31E-01	-6.61E-01	-8.03E-01	-5.19E-01	1.42E-01
GLU	11	O	4	VAL	12	C	495	5.78E-01	-7.49E-01	-9.89E-01	-5.09E-01	2.40E-01
GLU	11	O	4	HIS	13	C	511	8.38E-01	-4.06E-01	-4.38E-01	-3.73E-01	3.25E-02
GLU	11	CD	7	GLU	11	N	478	5.52E-01	-8.94E-01	-1.17E+00	-6.14E-01	2.80E-01
GLU	11	CD	7	GLU	11	O	481	6.51E-01	-9.53E-01	-1.30E+00	-6.09E-01	3.44E-01
GLU	11	CD	7	GLU	11	OE1	485	4.96E-01	-1.93E+00	-2.15E+00	-1.71E+00	2.20E-01
GLU	11	CD	7	GLU	11	OE2	486	5.36E-01	-1.62E+00	-1.84E+00	-1.39E+00	2.21E-01
GLU	11	CD	7	VAL	12	N	493	6.73E-01	-5.42E-01	-6.11E-01	-4.72E-01	6.92E-02
GLU	11	CD	7	HIS	13	N	509	9.21E-01	-3.56E-01	-3.78E-01	-3.34E-01	2.19E-02
GLU	11	OE1	8	GLU	11	C	480	5.97E-01	-9.43E-01	-1.26E+00	-6.29E-01	3.14E-01
GLU	11	OE1	8	GLU	11	CD	484	5.00E-01	-1.91E+00	-2.17E+00	-1.64E+00	2.62E-01
GLU	11	OE1	8	VAL	12	C	495	8.26E-01	-3.93E-01	-4.33E-01	-3.53E-01	3.96E-02
GLU	11	OE1	8	VAL	12	H	500	6.38E-01	-6.70E-01	-8.42E-01	-4.98E-01	1.72E-01
GLU	11	OE2	9	GLU	11	C	480	6.70E-01	-6.25E-01	-7.22E-01	-5.28E-01	9.71E-02
GLU	11	OE2	9	GLU	11	CD	484	4.65E-01	-2.27E+00	-2.57E+00	-1.97E+00	2.99E-01
GLU	11	OE2	9	VAL	12	H	500	7.24E-01	-5.03E-01	-5.82E-01	-4.24E-01	7.91E-02
VAL	12	N	16	GLU	11	C	480	5.15E-01	-6.38E-01	-7.93E-01	-4.83E-01	1.55E-01
VAL	12	N	16	GLU	11	CD	484	7.87E-01	-3.92E-01	-4.07E-01	-3.76E-01	1.53E-02
VAL	12	N	16	VAL	12	C	495	5.42E-01	-4.89E-01	-5.10E-01	-4.67E-01	2.16E-02
VAL	12	N	16	VAL	12	CB	497	5.40E-01	-4.34E-01	-4.55E-01	-4.13E-01	2.09E-02
VAL	12	N	16	VAL	12	H	500	4.67E-01	-8.26E-01	-1.05E+00	-5.97E-01	2.29E-01
VAL	12	N	16	HIS	13	C	511	7.80E-01	-3.48E-01	-3.75E-01	-3.21E-01	2.72E-02
VAL	12	C	18	VAL	12	N	493	5.48E-01	-4.75E-01	-4.92E-01	-4.58E-01	1.68E-02
VAL	12	C	18	VAL	12	O	496	4.62E-01	-8.46E-01	-1.12E+00	-5.76E-01	2.70E-01
VAL	12	C	18	HIS	13	N	509	5.13E-01	-7.29E-01	-9.17E-01	-5.42E-01	1.87E-01
VAL	12	C	18	HIS	14	N	526	6.57E-01	-3.78E-01	-3.88E-01	-3.69E-01	9.50E-03
VAL	12	O	19	VAL	12	C	495	5.08E-01	-6.75E-01	-9.39E-01	-4.11E-01	2.64E-01
VAL	12	O	19	VAL	12	H	500	5.59E-01	-4.06E-01	-4.39E-01	-3.73E-01	3.26E-02

VAL	12	CB	20	VAL	12	N	493	5.50E-01	-4.17E-01	-4.40E-01	-3.95E-01	2.25E-02
VAL	12	CB	20	HIS	13	N	509	6.05E-01	-4.04E-01	-4.45E-01	-3.62E-01	4.12E-02
VAL	12	H	23	GLU	11	OE1	485	7.98E-01	-4.14E-01	-4.62E-01	-3.66E-01	4.77E-02
VAL	12	H	23	VAL	12	N	493	5.04E-01	-6.74E-01	-8.86E-01	-4.63E-01	2.11E-01
VAL	12	H	23	VAL	12	O	496	5.45E-01	-4.34E-01	-4.75E-01	-3.93E-01	4.13E-02
HIS	13	N	32	GLU	11	C	480	6.61E-01	-3.93E-01	-4.01E-01	-3.84E-01	8.07E-03
HIS	13	N	32	VAL	12	C	495	4.79E-01	-8.57E-01	-1.05E+00	-6.64E-01	1.92E-01
HIS	13	N	32	VAL	12	CB	497	5.73E-01	-4.58E-01	-5.10E-01	-4.06E-01	5.17E-02
HIS	13	N	32	HIS	13	C	511	5.47E-01	-8.29E-01	-8.62E-01	-7.95E-01	3.32E-02
HIS	13	N	32	HIS	13	H	519	5.05E-01	-5.16E-01	-6.85E-01	-3.47E-01	1.69E-01
HIS	13	N	32	HIS	14	C	528	7.61E-01	-4.29E-01	-4.67E-01	-3.91E-01	3.81E-02
HIS	13	C	34	GLU	11	O	481	8.60E-01	-3.90E-01	-4.25E-01	-3.54E-01	3.51E-02
HIS	13	C	34	VAL	12	N	493	7.59E-01	-3.66E-01	-3.95E-01	-3.37E-01	2.87E-02
HIS	13	C	34	VAL	12	O	496	5.70E-01	-8.04E-01	-1.07E+00	-5.37E-01	2.67E-01
HIS	13	C	34	HIS	13	N	509	5.40E-01	-8.53E-01	-8.85E-01	-8.20E-01	3.22E-02
HIS	13	C	34	HIS	13	O	512	5.07E-01	-1.34E+00	-1.87E+00	-7.99E-01	5.37E-01
HIS	13	C	34	HIS	13	ND1	515	6.05E-01	-6.24E-01	-8.24E-01	-4.23E-01	2.00E-01
HIS	13	C	34	HIS	14	N	526	4.76E-01	-1.30E+00	-1.60E+00	-9.97E-01	3.03E-01
HIS	13	C	34	HIS	14	O	529	5.84E-01	-9.82E-01	-1.30E+00	-6.63E-01	3.18E-01
HIS	13	C	34	GLN	15	N	543	6.59E-01	-4.08E-01	-4.24E-01	-3.93E-01	1.54E-02
HIS	13	C	34	GLN	15	O	546	8.20E-01	-4.13E-01	-4.75E-01	-3.51E-01	6.17E-02
HIS	13	O	35	HIS	13	C	511	4.61E-01	-1.60E+00	-2.09E+00	-1.11E+00	4.93E-01
HIS	13	O	35	HIS	13	H	519	5.46E-01	-3.58E-01	-3.85E-01	-3.30E-01	2.74E-02
HIS	13	O	35	HIS	14	C	528	5.74E-01	-1.05E+00	-1.40E+00	-6.96E-01	3.49E-01
HIS	13	ND1	38	HIS	13	C	511	7.36E-01	-3.64E-01	-3.94E-01	-3.34E-01	3.04E-02
HIS	13	H	42	HIS	13	N	509	4.66E-01	-6.15E-01	-7.81E-01	-4.49E-01	1.66E-01
HIS	14	N	49	VAL	12	C	495	6.56E-01	-3.79E-01	-3.91E-01	-3.68E-01	1.14E-02
HIS	14	N	49	HIS	13	C	511	5.09E-01	-1.11E+00	-1.40E+00	-8.14E-01	2.95E-01
HIS	14	N	49	HIS	14	C	528	5.39E-01	-8.60E-01	-9.12E-01	-8.08E-01	5.17E-02
HIS	14	N	49	HIS	14	H	536	4.62E-01	-6.39E-01	-8.16E-01	-4.61E-01	1.78E-01
HIS	14	C	51	HIS	13	N	509	7.80E-01	-4.11E-01	-4.50E-01	-3.72E-01	3.90E-02
HIS	14	C	51	HIS	13	O	512	6.36E-01	-8.93E-01	-1.29E+00	-4.98E-01	3.95E-01
HIS	14	C	51	HIS	14	N	526	5.45E-01	-8.40E-01	-8.88E-01	-7.92E-01	4.81E-02
HIS	14	C	51	HIS	14	O	529	4.58E-01	-1.66E+00	-2.19E+00	-1.13E+00	5.29E-01
HIS	14	C	51	HIS	14	ND1	532	6.54E-01	-5.06E-01	-6.48E-01	-3.64E-01	1.42E-01
HIS	14	C	51	GLN	15	N	543	5.09E-01	-8.09E-01	-1.02E+00	-5.98E-01	2.11E-01
HIS	14	C	51	GLN	15	O	546	5.85E-01	-1.00E+00	-1.32E+00	-6.90E-01	3.15E-01
HIS	14	C	51	GLN	15	OE1	550	7.40E-01	-5.73E-01	-6.81E-01	-4.64E-01	1.09E-01
HIS	14	C	51	GLN	15	NE2	551	7.70E-01	-6.95E-01	-7.39E-01	-6.50E-01	4.46E-02
HIS	14	C	51	LYS	16	N	560	6.58E-01	-4.62E-01	-4.80E-01	-4.44E-01	1.82E-02
HIS	14	O	52	HIS	13	C	511	6.40E-01	-8.55E-01	-1.22E+00	-4.90E-01	3.65E-01
HIS	14	O	52	HIS	14	C	528	5.07E-01	-1.29E+00	-1.79E+00	-8.01E-01	4.93E-01

HIS	14	O	52	GLN	15	CD	549	7.22E-01	-5.33E-01	-6.69E-01	-3.97E-01	1.36E-01
HIS	14	O	52	LYS	16	C	562	8.70E-01	-3.75E-01	-4.13E-01	-3.37E-01	3.82E-02
HIS	14	ND1	55	HIS	14	C	528	7.01E-01	-4.28E-01	-5.26E-01	-3.31E-01	9.74E-02
HIS	14	H	59	HIS	14	N	526	5.02E-01	-5.10E-01	-6.68E-01	-3.53E-01	1.57E-01
GLN	15	N	66	HIS	13	C	511	6.60E-01	-4.08E-01	-4.27E-01	-3.89E-01	1.93E-02
GLN	15	N	66	HIS	14	C	528	4.79E-01	-9.28E-01	-1.13E+00	-7.21E-01	2.07E-01
GLN	15	N	66	GLN	15	C	545	5.22E-01	-4.35E-01	-4.81E-01	-3.88E-01	4.62E-02
GLN	15	N	66	GLN	15	CD	549	6.55E-01	-4.21E-01	-4.56E-01	-3.86E-01	3.52E-02
GLN	15	C	68	GLN	15	O	546	4.60E-01	-1.11E+00	-1.46E+00	-7.59E-01	3.53E-01
GLN	15	C	68	GLN	15	OE1	550	6.94E-01	-3.89E-01	-4.32E-01	-3.45E-01	4.37E-02
GLN	15	C	68	GLN	15	NE2	551	7.52E-01	-4.69E-01	-5.24E-01	-4.14E-01	5.49E-02
GLN	15	C	68	LYS	16	N	560	5.10E-01	-5.81E-01	-7.39E-01	-4.22E-01	1.58E-01
GLN	15	O	69	HIS	14	C	528	6.38E-01	-8.38E-01	-1.15E+00	-5.25E-01	3.13E-01
GLN	15	O	69	GLN	15	C	545	5.09E-01	-8.65E-01	-1.20E+00	-5.35E-01	3.31E-01
GLN	15	O	69	GLN	15	CD	549	7.26E-01	-5.22E-01	-6.06E-01	-4.39E-01	8.37E-02
GLN	15	O	69	LYS	16	C	562	6.54E-01	-8.49E-01	-1.15E+00	-5.45E-01	3.04E-01
GLN	15	CD	72	HIS	14	N	526	7.68E-01	-4.25E-01	-4.60E-01	-3.90E-01	3.47E-02
GLN	15	CD	72	HIS	14	O	529	6.41E-01	-7.33E-01	-9.53E-01	-5.14E-01	2.20E-01
GLN	15	CD	72	GLN	15	N	543	6.46E-01	-4.33E-01	-4.67E-01	-3.99E-01	3.39E-02
GLN	15	CD	72	GLN	15	O	546	6.86E-01	-6.03E-01	-7.18E-01	-4.89E-01	1.14E-01
GLN	15	CD	72	GLN	15	OE1	550	4.84E-01	-1.65E+00	-2.18E+00	-1.12E+00	5.29E-01
GLN	15	CD	72	GLN	15	NE2	551	5.04E-01	-1.94E+00	-2.46E+00	-1.43E+00	5.18E-01
GLN	15	CD	72	LYS	16	N	560	7.52E-01	-3.62E-01	-3.87E-01	-3.38E-01	2.43E-02
GLN	15	OE1	73	HIS	13	C	511	8.34E-01	-4.34E-01	-4.73E-01	-3.95E-01	3.89E-02
GLN	15	OE1	73	HIS	14	C	528	6.93E-01	-7.29E-01	-1.00E+00	-4.52E-01	2.76E-01
GLN	15	OE1	73	GLN	15	CD	549	5.00E-01	-1.51E+00	-1.98E+00	-1.03E+00	4.75E-01
GLN	15	OE1	73	GLN	15	HE22	559	5.31E-01	-1.02E+00	-1.47E+00	-5.70E-01	4.51E-01
GLN	15	NE2	74	HIS	13	C	511	8.62E-01	-5.73E-01	-6.11E-01	-5.36E-01	3.77E-02
GLN	15	NE2	74	HIS	14	C	528	7.15E-01	-8.57E-01	-1.07E+00	-6.48E-01	2.09E-01
GLN	15	NE2	74	GLN	15	C	545	7.41E-01	-4.88E-01	-5.56E-01	-4.21E-01	6.73E-02
GLN	15	NE2	74	GLN	15	CD	549	4.99E-01	-1.99E+00	-2.52E+00	-1.47E+00	5.24E-01
GLN	15	NE2	74	GLN	15	HE21	558	5.11E-01	-1.14E+00	-1.43E+00	-8.48E-01	2.89E-01
GLN	15	NE2	74	GLN	15	HE22	559	5.06E-01	-1.03E+00	-1.10E+00	-9.63E-01	6.86E-02
GLN	15	H	75	GLN	15	O	546	5.04E-01	-5.98E-01	-8.40E-01	-3.55E-01	2.43E-01
GLN	15	HE21	81	GLN	15	NE2	551	4.96E-01	-1.24E+00	-1.57E+00	-9.11E-01	3.28E-01
GLN	15	HE22	82	GLN	15	OE1	550	5.19E-01	-1.08E+00	-1.53E+00	-6.22E-01	4.53E-01
GLN	15	HE22	82	GLN	15	NE2	551	5.15E-01	-9.92E-01	-1.08E+00	-9.08E-01	8.34E-02
LYS	16	N	83	HIS	14	C	528	6.44E-01	-4.91E-01	-5.50E-01	-4.33E-01	5.82E-02
LYS	16	N	83	GLN	15	C	545	4.77E-01	-6.75E-01	-8.31E-01	-5.20E-01	1.55E-01
LYS	16	N	83	GLN	15	CD	549	7.16E-01	-3.99E-01	-4.32E-01	-3.66E-01	3.32E-02
LYS	16	N	83	LYS	16	C	562	5.84E-01	-6.53E-01	-6.98E-01	-6.08E-01	4.51E-02
LYS	16	N	83	LEU	17	C	584	7.15E-01	-3.39E-01	-3.52E-01	-3.27E-01	1.26E-02

LYS	16	N	83	LEU	17	H	590	4.67E-01	-3.97E-01	-4.28E-01	-3.67E-01	3.08E-02
LYS	16	C	85	HIS	14	O	529	7.73E-01	-4.84E-01	-5.65E-01	-4.03E-01	8.10E-02
LYS	16	C	85	GLN	15	N	543	7.18E-01	-3.77E-01	-3.85E-01	-3.70E-01	7.14E-03
LYS	16	C	85	GLN	15	O	546	5.17E-01	-1.39E+00	-1.81E+00	-9.73E-01	4.18E-01
LYS	16	C	85	GLN	15	OE1	550	8.32E-01	-4.96E-01	-5.72E-01	-4.20E-01	7.59E-02
LYS	16	C	85	GLN	15	NE2	551	8.93E-01	-5.94E-01	-6.35E-01	-5.53E-01	4.09E-02
LYS	16	C	85	LYS	16	N	560	4.98E-01	-9.49E-01	-1.05E+00	-8.45E-01	1.04E-01
LYS	16	C	85	LYS	16	O	563	6.05E-01	-7.74E-01	-7.79E-01	-7.70E-01	4.41E-03
LYS	16	C	85	LEU	17	N	582	4.10E-01	-1.23E+00	-1.25E+00	-1.21E+00	2.05E-02
LYS	16	C	85	LEU	17	O	585	4.33E-01	-1.68E+00	-1.75E+00	-1.62E+00	6.40E-02
LYS	16	C	85	VAL	18	N	601	6.30E-01	-5.69E-01	-5.87E-01	-5.51E-01	1.81E-02
LYS	16	O	86	HIS	14	C	528	7.49E-01	-4.69E-01	-5.05E-01	-4.33E-01	3.65E-02
LYS	16	O	86	GLN	15	C	545	4.70E-01	-8.14E-01	-8.97E-01	-7.31E-01	8.27E-02
LYS	16	O	86	GLN	15	CD	549	7.83E-01	-4.49E-01	-5.09E-01	-3.89E-01	5.99E-02
LYS	16	O	86	LYS	16	C	562	3.68E-01	-2.59E+00	-2.65E+00	-2.52E+00	6.18E-02
LYS	16	O	86	LYS	16	H	569	4.54E-01	-6.74E-01	-9.44E-01	-4.05E-01	2.69E-01
LYS	16	O	86	LEU	17	C	584	4.08E-01	-1.57E+00	-1.66E+00	-1.48E+00	9.13E-02
LYS	16	O	86	LEU	17	H	590	1.96E-01	-5.86E+00	-6.39E+00	-5.33E+00	5.31E-01
LYS	16	O	86	VAL	18	CB	605	6.56E-01	-3.60E-01	-3.84E-01	-3.37E-01	2.34E-02
LYS	16	O	86	VAL	18	H	608	5.87E-01	-5.03E-01	-5.24E-01	-4.81E-01	2.13E-02
LEU	17	N	105	LYS	16	C	562	5.77E-01	-5.41E-01	-5.48E-01	-5.33E-01	7.37E-03
LEU	17	N	105	LEU	17	C	584	5.28E-01	-5.23E-01	-5.32E-01	-5.13E-01	9.59E-03
LEU	17	N	105	LEU	17	H	590	3.84E-01	-5.27E-01	-5.37E-01	-5.16E-01	1.07E-02
LEU	17	C	107	LYS	16	O	563	7.77E-01	-3.75E-01	-3.82E-01	-3.68E-01	6.62E-03
LEU	17	C	107	LEU	17	N	582	5.58E-01	-4.62E-01	-4.72E-01	-4.51E-01	1.03E-02
LEU	17	C	107	LEU	17	O	585	3.65E-01	-2.08E+00	-2.11E+00	-2.05E+00	2.94E-02
LEU	17	C	107	VAL	18	N	601	5.81E-01	-5.33E-01	-5.38E-01	-5.27E-01	5.57E-03
LEU	17	C	107	PHE	19	N	617	6.64E-01	-3.34E-01	-3.46E-01	-3.21E-01	1.22E-02
LEU	17	O	108	LYS	16	C	562	7.82E-01	-4.65E-01	-4.72E-01	-4.58E-01	7.00E-03
LEU	17	O	108	LEU	17	C	584	6.04E-01	-6.08E-01	-6.13E-01	-6.03E-01	4.63E-03
LEU	17	H	113	LEU	17	O	585	5.30E-01	-3.73E-01	-3.84E-01	-3.61E-01	1.13E-02
LEU	17	HA	114	LEU	17	O	585	2.59E-01	-1.33E+00	-1.48E+00	-1.19E+00	1.45E-01
VAL	18	N	124	LYS	16	C	562	6.59E-01	-5.19E-01	-5.35E-01	-5.02E-01	1.65E-02
VAL	18	N	124	LEU	17	C	584	4.11E-01	-1.22E+00	-1.26E+00	-1.19E+00	3.73E-02
VAL	18	N	124	LEU	17	H	590	4.79E-01	-3.81E-01	-3.94E-01	-3.68E-01	1.29E-02
VAL	18	N	124	VAL	18	C	603	5.63E-01	-4.47E-01	-4.62E-01	-4.32E-01	1.51E-02
VAL	18	N	124	VAL	18	CB	605	5.66E-01	-3.91E-01	-4.04E-01	-3.78E-01	1.33E-02
VAL	18	N	124	VAL	18	H	608	5.85E-01	-4.03E-01	-4.07E-01	-4.00E-01	3.72E-03
VAL	18	N	124	PHE	19	H	628	4.48E-01	-4.05E-01	-4.32E-01	-3.77E-01	2.77E-02
VAL	18	C	126	LEU	17	O	585	4.20E-01	-1.13E+00	-1.20E+00	-1.05E+00	7.27E-02
VAL	18	C	126	VAL	18	N	601	5.32E-01	-5.08E-01	-5.29E-01	-4.88E-01	2.04E-02
VAL	18	C	126	VAL	18	O	604	6.11E-01	-3.36E-01	-3.38E-01	-3.34E-01	1.79E-03

VAL	18	C	126	PHE	19	N	617	4.11E-01	-7.84E-01	-8.05E-01	-7.63E-01	2.09E-02
VAL	18	C	126	PHE	19	O	620	5.80E-01	-4.73E-01	-4.95E-01	-4.50E-01	2.27E-02
VAL	18	O	127	LYS	16	C	562	7.82E-01	-3.38E-01	-3.49E-01	-3.26E-01	1.11E-02
VAL	18	O	127	LEU	17	C	584	4.44E-01	-9.12E-01	-9.79E-01	-8.44E-01	6.74E-02
VAL	18	O	127	VAL	18	C	603	3.73E-01	-1.11E+00	-1.13E+00	-1.09E+00	2.05E-02
VAL	18	O	127	VAL	18	CB	605	4.70E-01	-5.44E-01	-5.77E-01	-5.12E-01	3.25E-02
VAL	18	O	127	VAL	18	H	608	5.33E-01	-4.51E-01	-4.75E-01	-4.26E-01	2.43E-02
VAL	18	O	127	PHE	19	C	619	4.76E-01	-7.31E-01	-7.69E-01	-6.94E-01	3.76E-02
VAL	18	O	127	PHE	19	H	628	1.92E-01	-3.95E+00	-4.13E+00	-3.77E+00	1.83E-01
VAL	18	CB	128	LEU	17	O	585	3.95E-01	-1.18E+00	-1.32E+00	-1.04E+00	1.41E-01
VAL	18	CB	128	VAL	18	N	601	5.21E-01	-4.73E-01	-4.97E-01	-4.49E-01	2.39E-02
VAL	18	CB	128	PHE	19	N	617	5.14E-01	-4.02E-01	-4.25E-01	-3.79E-01	2.30E-02
VAL	18	CB	128	PHE	19	O	620	6.46E-01	-3.33E-01	-3.47E-01	-3.18E-01	1.45E-02
VAL	18	H	131	LYS	16	O	563	6.60E-01	-3.93E-01	-4.09E-01	-3.78E-01	1.55E-02
VAL	18	H	131	LEU	17	N	582	4.56E-01	-5.72E-01	-6.02E-01	-5.42E-01	2.99E-02
VAL	18	H	131	LEU	17	O	585	1.93E-01	-1.01E+01	-1.08E+01	-9.43E+00	6.77E-01
VAL	18	H	131	VAL	18	N	601	3.90E-01	-1.07E+00	-1.09E+00	-1.06E+00	1.65E-02
VAL	18	H	131	VAL	18	O	604	5.99E-01	-3.47E-01	-3.59E-01	-3.35E-01	1.21E-02
VAL	18	H	131	PHE	19	N	617	4.75E-01	-5.43E-01	-5.78E-01	-5.07E-01	3.51E-02
PHE	19	N	140	LEU	17	C	584	6.42E-01	-3.57E-01	-3.70E-01	-3.44E-01	1.32E-02
PHE	19	N	140	VAL	18	C	603	5.89E-01	-3.34E-01	-3.38E-01	-3.29E-01	4.18E-03
PHE	19	N	140	PHE	19	C	619	6.17E-01	-3.71E-01	-3.81E-01	-3.60E-01	1.06E-02
PHE	19	N	140	PHE	19	H	628	3.94E-01	-4.61E-01	-4.67E-01	-4.54E-01	6.40E-03
PHE	19	C	142	LEU	17	O	585	7.09E-01	-4.22E-01	-4.35E-01	-4.08E-01	1.36E-02
PHE	19	C	142	PHE	19	N	617	4.76E-01	-6.71E-01	-7.08E-01	-6.34E-01	3.70E-02
PHE	19	C	142	PHE	19	O	620	5.07E-01	-7.83E-01	-8.09E-01	-7.57E-01	2.57E-02
PHE	19	C	142	PHE	20	N	637	5.47E-01	-4.81E-01	-4.93E-01	-4.70E-01	1.12E-02
PHE	19	C	142	PHE	20	O	640	5.39E-01	-6.85E-01	-7.22E-01	-6.48E-01	3.70E-02
PHE	19	O	143	VAL	18	C	603	5.91E-01	-4.54E-01	-4.77E-01	-4.32E-01	2.24E-02
PHE	19	O	143	PHE	19	C	619	4.96E-01	-8.28E-01	-8.54E-01	-8.02E-01	2.61E-02
PHE	19	O	143	PHE	19	H	628	4.15E-01	-5.55E-01	-6.00E-01	-5.09E-01	4.51E-02
PHE	19	O	143	PHE	20	C	639	6.14E-01	-5.12E-01	-5.37E-01	-4.88E-01	2.41E-02
PHE	20	N	160	PHE	19	C	619	4.67E-01	-7.02E-01	-7.41E-01	-6.64E-01	3.87E-02
PHE	20	N	160	PHE	20	C	639	5.67E-01	-4.45E-01	-4.56E-01	-4.34E-01	1.11E-02
PHE	20	C	162	PHE	19	N	617	6.39E-01	-3.48E-01	-3.76E-01	-3.20E-01	2.77E-02
PHE	20	C	162	PHE	19	O	620	5.16E-01	-7.74E-01	-8.86E-01	-6.61E-01	1.12E-01
PHE	20	C	162	PHE	20	N	637	5.47E-01	-4.83E-01	-4.99E-01	-4.68E-01	1.58E-02
PHE	20	C	162	PHE	20	O	640	3.73E-01	-1.71E+00	-1.73E+00	-1.68E+00	2.68E-02
PHE	20	C	162	ALA	21	N	657	5.90E-01	-4.44E-01	-4.49E-01	-4.40E-01	4.53E-03
PHE	20	C	162	ALA	21	O	660	5.92E-01	-6.05E-01	-6.15E-01	-5.95E-01	1.03E-02
PHE	20	C	162	GLU	22	N	667	6.56E-01	-3.72E-01	-3.77E-01	-3.66E-01	5.45E-03
PHE	20	C	162	ASN	27	ND2	735	9.07E-01	-3.87E-01	-4.11E-01	-3.62E-01	2.47E-02

PHE	20	O	163	PHE	19	C	619	6.41E-01	-4.69E-01	-4.95E-01	-4.43E-01	2.59E-02
PHE	20	O	163	PHE	20	C	639	6.16E-01	-5.08E-01	-5.12E-01	-5.03E-01	4.55E-03
PHE	20	O	163	ALA	21	C	659	7.00E-01	-4.07E-01	-4.11E-01	-4.04E-01	3.54E-03
PHE	20	HA	172	PHE	20	O	640	2.71E-01	-7.77E-01	-8.27E-01	-7.27E-01	4.97E-02
ALA	21	N	180	PHE	19	C	619	5.21E-01	-5.89E-01	-6.24E-01	-5.55E-01	3.42E-02
ALA	21	N	180	PHE	20	C	639	4.19E-01	-1.00E+00	-1.02E+00	-9.83E-01	1.74E-02
ALA	21	N	180	ALA	21	C	659	4.79E-01	-7.46E-01	-7.57E-01	-7.36E-01	1.05E-02
ALA	21	C	182	PHE	19	O	620	7.13E-01	-3.95E-01	-4.17E-01	-3.73E-01	2.21E-02
ALA	21	C	182	PHE	20	O	640	4.83E-01	-9.15E-01	-9.38E-01	-8.91E-01	2.37E-02
ALA	21	C	182	ALA	21	N	657	6.28E-01	-4.04E-01	-4.09E-01	-4.00E-01	4.10E-03
ALA	21	C	182	ALA	21	O	660	4.99E-01	-9.28E-01	-9.42E-01	-9.13E-01	1.45E-02
ALA	21	C	182	GLU	22	N	667	4.89E-01	-7.41E-01	-7.54E-01	-7.28E-01	1.30E-02
ALA	21	C	182	GLU	22	O	670	6.83E-01	-5.01E-01	-5.08E-01	-4.94E-01	6.61E-03
ALA	21	C	182	GLU	22	OE1	674	7.06E-01	-6.68E-01	-7.50E-01	-5.86E-01	8.19E-02
ALA	21	C	182	GLU	22	OE2	675	7.60E-01	-5.73E-01	-6.09E-01	-5.36E-01	3.64E-02
ALA	21	C	182	ASP	23	N	682	5.94E-01	-6.28E-01	-6.47E-01	-6.10E-01	1.85E-02
ALA	21	O	183	PHE	19	C	619	7.79E-01	-3.52E-01	-3.64E-01	-3.40E-01	1.19E-02
ALA	21	O	183	PHE	20	C	639	6.03E-01	-5.81E-01	-5.94E-01	-5.67E-01	1.37E-02
ALA	21	O	183	ALA	21	C	659	5.29E-01	-8.10E-01	-8.25E-01	-7.95E-01	1.51E-02
ALA	21	O	183	GLU	22	C	669	5.93E-01	-5.17E-01	-5.33E-01	-5.02E-01	1.53E-02
ALA	21	O	183	GLU	22	CD	673	7.73E-01	-5.03E-01	-5.40E-01	-4.65E-01	3.76E-02
ALA	21	O	183	GLU	22	H	676	5.94E-01	-3.37E-01	-3.46E-01	-3.27E-01	9.26E-03
ALA	21	O	183	ASP	23	CG	687	9.59E-01	-3.39E-01	-3.45E-01	-3.32E-01	6.64E-03
ALA	21	O	183	ASP	23	H	690	5.19E-01	-4.76E-01	-5.01E-01	-4.51E-01	2.51E-02
ALA	21	O	183	VAL	24	H	701	6.46E-01	-4.06E-01	-4.24E-01	-3.87E-01	1.85E-02
ALA	21	CB	184	PHE	20	C	639	4.54E-01	-4.63E-01	-4.76E-01	-4.50E-01	1.29E-02
ALA	21	CB	184	ALA	21	C	659	3.75E-01	-7.93E-01	-8.06E-01	-7.79E-01	1.33E-02
ALA	21	CB	184	GLU	22	H	676	3.67E-01	-4.52E-01	-4.71E-01	-4.33E-01	1.91E-02
ALA	21	H	185	PHE	19	O	620	4.10E-01	-7.28E-01	-8.33E-01	-6.22E-01	1.06E-01
ALA	21	H	185	PHE	20	N	637	4.46E-01	-4.20E-01	-4.34E-01	-4.06E-01	1.41E-02
ALA	21	H	185	PHE	20	O	640	1.96E-01	-5.87E+00	-6.09E+00	-5.65E+00	2.20E-01
ALA	21	H	185	ALA	21	N	657	3.97E-01	-6.16E-01	-6.26E-01	-6.07E-01	9.73E-03
ALA	21	H	185	ALA	21	O	660	4.09E-01	-7.86E-01	-8.05E-01	-7.68E-01	1.88E-02
ALA	21	H	185	GLU	22	N	667	4.75E-01	-4.11E-01	-4.21E-01	-4.01E-01	9.92E-03
GLU	22	N	190	PHE	20	C	639	6.92E-01	-3.34E-01	-3.38E-01	-3.30E-01	4.18E-03
GLU	22	N	190	ALA	21	C	659	5.46E-01	-5.73E-01	-5.79E-01	-5.68E-01	5.52E-03
GLU	22	N	190	GLU	22	C	669	5.71E-01	-4.28E-01	-4.35E-01	-4.20E-01	7.52E-03
GLU	22	N	190	GLU	22	CD	673	6.17E-01	-6.00E-01	-6.64E-01	-5.35E-01	6.46E-02
GLU	22	N	190	GLU	22	H	676	5.30E-01	-3.30E-01	-3.34E-01	-3.25E-01	4.33E-03
GLU	22	N	190	ASP	23	H	690	4.91E-01	-4.13E-01	-4.29E-01	-3.96E-01	1.65E-02
GLU	22	C	192	ALA	21	O	660	5.90E-01	-5.23E-01	-5.36E-01	-5.11E-01	1.26E-02
GLU	22	C	192	GLU	22	N	667	5.49E-01	-4.67E-01	-4.73E-01	-4.61E-01	6.12E-03

GLU	22	C	192	GLU	22	O	670	6.16E-01	-5.08E-01	-5.12E-01	-5.05E-01	3.29E-03
GLU	22	C	192	GLU	22	OE1	674	6.73E-01	-5.91E-01	-6.16E-01	-5.66E-01	2.51E-02
GLU	22	C	192	GLU	22	OE2	675	6.52E-01	-6.32E-01	-6.62E-01	-6.02E-01	2.98E-02
GLU	22	C	192	ASP	23	N	682	4.37E-01	-1.07E+00	-1.09E+00	-1.04E+00	2.90E-02
GLU	22	C	192	ASP	23	OD1	688	7.66E-01	-4.10E-01	-4.25E-01	-3.94E-01	1.50E-02
GLU	22	C	192	ASP	23	OD2	689	8.37E-01	-3.50E-01	-3.58E-01	-3.41E-01	8.31E-03
GLU	22	C	192	VAL	24	N	694	6.11E-01	-3.94E-01	-4.03E-01	-3.85E-01	9.44E-03
GLU	22	O	193	PHE	20	C	639	7.34E-01	-4.20E-01	-4.29E-01	-4.10E-01	9.61E-03
GLU	22	O	193	ALA	21	C	659	5.01E-01	-9.81E-01	-1.01E+00	-9.55E-01	2.55E-02
GLU	22	O	193	GLU	22	C	669	3.75E-01	-1.69E+00	-1.72E+00	-1.65E+00	3.42E-02
GLU	22	O	193	GLU	22	CD	673	5.61E-01	-1.02E+00	-1.05E+00	-9.79E-01	3.78E-02
GLU	22	O	193	GLU	22	H	676	5.06E-01	-5.15E-01	-5.23E-01	-5.06E-01	8.38E-03
GLU	22	O	193	GLU	22	HA	677	2.44E-01	-7.77E-01	-7.83E-01	-7.72E-01	5.49E-03
GLU	22	O	193	ASP	23	C	684	5.24E-01	-6.88E-01	-7.08E-01	-6.68E-01	2.01E-02
GLU	22	O	193	ASP	23	CG	687	6.63E-01	-6.94E-01	-7.16E-01	-6.72E-01	2.19E-02
GLU	22	O	193	ASP	23	H	690	2.29E-01	-4.72E+00	-5.22E+00	-4.22E+00	4.98E-01
GLU	22	O	193	VAL	24	C	696	7.12E-01	-3.62E-01	-3.68E-01	-3.56E-01	6.20E-03
GLU	22	O	193	VAL	24	CB	698	6.34E-01	-4.04E-01	-4.20E-01	-3.87E-01	1.63E-02
GLU	22	O	193	VAL	24	H	701	4.47E-01	-1.00E+00	-1.04E+00	-9.60E-01	4.11E-02
GLU	22	CD	196	PHE	20	O	640	9.51E-01	-3.23E-01	-3.40E-01	-3.07E-01	1.65E-02
GLU	22	CD	196	ALA	21	O	660	8.48E-01	-4.24E-01	-4.35E-01	-4.13E-01	1.07E-02
GLU	22	CD	196	GLU	22	N	667	6.92E-01	-4.68E-01	-4.81E-01	-4.55E-01	1.34E-02
GLU	22	CD	196	GLU	22	O	670	8.10E-01	-4.90E-01	-5.03E-01	-4.77E-01	1.29E-02
GLU	22	CD	196	GLU	22	OE1	674	4.95E-01	-1.91E+00	-2.09E+00	-1.73E+00	1.78E-01
GLU	22	CD	196	GLU	22	OE2	675	4.81E-01	-2.03E+00	-2.14E+00	-1.92E+00	1.08E-01
GLU	22	CD	196	ASP	23	N	682	6.84E-01	-6.33E-01	-6.64E-01	-6.03E-01	3.05E-02
GLU	22	OE1	197	ALA	21	C	659	8.47E-01	-4.79E-01	-5.30E-01	-4.28E-01	5.08E-02
GLU	22	OE1	197	GLU	22	C	669	7.52E-01	-4.79E-01	-4.96E-01	-4.62E-01	1.70E-02
GLU	22	OE1	197	GLU	22	CD	673	5.15E-01	-1.74E+00	-1.89E+00	-1.58E+00	1.59E-01
GLU	22	OE1	197	ASP	23	H	690	6.58E-01	-4.25E-01	-4.57E-01	-3.92E-01	3.22E-02
GLU	22	OE2	198	ALA	21	C	659	8.55E-01	-4.64E-01	-4.82E-01	-4.45E-01	1.83E-02
GLU	22	OE2	198	GLU	22	C	669	7.60E-01	-4.69E-01	-4.87E-01	-4.51E-01	1.77E-02
GLU	22	OE2	198	GLU	22	CD	673	5.15E-01	-1.72E+00	-1.80E+00	-1.64E+00	8.23E-02
GLU	22	OE2	198	ASP	23	H	690	6.71E-01	-4.11E-01	-4.47E-01	-3.74E-01	3.63E-02
GLU	22	H	199	ALA	21	O	660	5.87E-01	-3.46E-01	-3.54E-01	-3.37E-01	8.74E-03
GLU	22	H	199	GLU	22	N	667	4.95E-01	-3.88E-01	-3.93E-01	-3.83E-01	4.94E-03
GLU	22	H	199	GLU	22	OE1	674	5.72E-01	-5.55E-01	-6.17E-01	-4.93E-01	6.19E-02
GLU	22	H	199	GLU	22	OE2	675	6.28E-01	-4.51E-01	-4.95E-01	-4.07E-01	4.43E-02
GLU	22	H	199	ASP	23	N	682	6.08E-01	-3.22E-01	-3.30E-01	-3.14E-01	7.93E-03
ASP	23	N	205	ALA	21	C	659	7.24E-01	-4.20E-01	-4.26E-01	-4.15E-01	5.64E-03
ASP	23	N	205	GLU	22	C	669	5.75E-01	-5.56E-01	-5.62E-01	-5.51E-01	5.31E-03
ASP	23	N	205	GLU	22	CD	673	7.10E-01	-5.87E-01	-6.03E-01	-5.70E-01	1.61E-02

ASP	23	N	205	ASP	23	C	684	6.52E-01	-4.01E-01	-4.08E-01	-3.95E-01	6.52E-03
ASP	23	N	205	ASP	23	CG	687	7.45E-01	-5.21E-01	-5.31E-01	-5.12E-01	9.58E-03
ASP	23	N	205	ASP	23	H	690	3.91E-01	-9.64E-01	-9.77E-01	-9.51E-01	1.30E-02
ASP	23	N	205	VAL	24	H	701	5.30E-01	-6.24E-01	-6.44E-01	-6.04E-01	2.02E-02
ASP	23	C	207	ALA	21	O	660	6.65E-01	-3.84E-01	-3.92E-01	-3.77E-01	7.55E-03
ASP	23	C	207	GLU	22	O	670	6.34E-01	-4.52E-01	-4.60E-01	-4.43E-01	8.27E-03
ASP	23	C	207	GLU	22	OE1	674	8.81E-01	-3.45E-01	-3.66E-01	-3.23E-01	2.16E-02
ASP	23	C	207	GLU	22	OE2	675	8.21E-01	-3.87E-01	-4.08E-01	-3.67E-01	2.07E-02
ASP	23	C	207	ASP	23	N	682	4.24E-01	-1.08E+00	-1.12E+00	-1.05E+00	3.51E-02
ASP	23	C	207	ASP	23	O	685	6.09E-01	-4.16E-01	-4.19E-01	-4.13E-01	2.90E-03
ASP	23	C	207	ASP	23	OD1	688	5.87E-01	-6.59E-01	-6.90E-01	-6.27E-01	3.18E-02
ASP	23	C	207	ASP	23	OD2	689	7.24E-01	-4.28E-01	-4.43E-01	-4.14E-01	1.47E-02
ASP	23	C	207	VAL	24	N	694	4.21E-01	-8.86E-01	-9.09E-01	-8.63E-01	2.30E-02
ASP	23	C	207	VAL	24	O	697	4.55E-01	-6.59E-01	-6.83E-01	-6.35E-01	2.38E-02
ASP	23	O	208	ALA	21	C	659	6.58E-01	-4.57E-01	-4.73E-01	-4.41E-01	1.59E-02
ASP	23	O	208	GLU	22	C	669	4.44E-01	-9.21E-01	-9.68E-01	-8.74E-01	4.72E-02
ASP	23	O	208	GLU	22	CD	673	7.64E-01	-4.59E-01	-4.73E-01	-4.45E-01	1.41E-02
ASP	23	O	208	ASP	23	C	684	3.65E-01	-1.44E+00	-1.47E+00	-1.41E+00	3.14E-02
ASP	23	O	208	ASP	23	CG	687	5.22E-01	-9.87E-01	-1.01E+00	-9.59E-01	2.78E-02
ASP	23	O	208	ASP	23	H	690	2.78E-01	-2.27E+00	-2.53E+00	-2.01E+00	2.58E-01
ASP	23	O	208	ASP	23	HA	691	2.54E-01	-7.48E-01	-7.81E-01	-7.15E-01	3.30E-02
ASP	23	O	208	VAL	24	C	696	4.41E-01	-8.90E-01	-9.29E-01	-8.52E-01	3.81E-02
ASP	23	O	208	VAL	24	CB	698	4.56E-01	-7.30E-01	-7.89E-01	-6.72E-01	5.85E-02
ASP	23	O	208	VAL	24	H	701	2.06E-01	-7.59E+00	-8.34E+00	-6.85E+00	7.46E-01
ASP	23	O	208	GLY	25	C	712	6.95E-01	-4.17E-01	-4.26E-01	-4.08E-01	8.75E-03
ASP	23	CG	210	ALA	21	O	660	8.48E-01	-4.12E-01	-4.19E-01	-4.06E-01	6.84E-03
ASP	23	CG	210	GLU	22	N	667	8.08E-01	-3.42E-01	-3.50E-01	-3.34E-01	7.98E-03
ASP	23	CG	210	GLU	22	O	670	6.93E-01	-6.36E-01	-6.50E-01	-6.23E-01	1.35E-02
ASP	23	CG	210	GLU	22	OE1	674	8.48E-01	-6.32E-01	-7.22E-01	-5.43E-01	8.95E-02
ASP	23	CG	210	GLU	22	OE2	675	7.36E-01	-8.08E-01	-8.88E-01	-7.29E-01	7.99E-02
ASP	23	CG	210	ASP	23	N	682	4.75E-01	-1.37E+00	-1.42E+00	-1.32E+00	5.36E-02
ASP	23	CG	210	ASP	23	O	685	6.52E-01	-6.06E-01	-6.18E-01	-5.94E-01	1.19E-02
ASP	23	CG	210	ASP	23	OD1	688	4.75E-01	-1.82E+00	-1.97E+00	-1.67E+00	1.50E-01
ASP	23	CG	210	ASP	23	OD2	689	5.58E-01	-1.25E+00	-1.35E+00	-1.15E+00	1.01E-01
ASP	23	CG	210	VAL	24	N	694	5.38E-01	-8.29E-01	-8.63E-01	-7.94E-01	3.47E-02
ASP	23	CG	210	VAL	24	O	697	6.08E-01	-5.70E-01	-6.02E-01	-5.39E-01	3.17E-02
ASP	23	CG	210	GLY	25	N	710	7.49E-01	-3.46E-01	-3.59E-01	-3.34E-01	1.24E-02
ASP	23	CG	210	GLY	25	O	713	8.60E-01	-3.70E-01	-3.84E-01	-3.57E-01	1.35E-02
ASP	23	OD1	211	ALA	21	C	659	9.43E-01	-3.50E-01	-3.61E-01	-3.39E-01	1.09E-02
ASP	23	OD1	211	GLU	22	C	669	6.73E-01	-5.28E-01	-5.64E-01	-4.93E-01	3.56E-02
ASP	23	OD1	211	GLU	22	CD	673	8.74E-01	-5.34E-01	-5.63E-01	-5.05E-01	2.90E-02
ASP	23	OD1	211	ASP	23	C	684	5.63E-01	-7.31E-01	-8.09E-01	-6.54E-01	7.73E-02

ASP	23	OD1	211	ASP	23	CG	687	5.33E-01	-1.41E+00	-1.61E+00	-1.20E+00	2.04E-01
ASP	23	OD1	211	ASP	23	H	690	4.91E-01	-7.24E-01	-8.00E-01	-6.48E-01	7.58E-02
ASP	23	OD1	211	VAL	24	C	696	6.10E-01	-6.19E-01	-6.76E-01	-5.62E-01	5.72E-02
ASP	23	OD1	211	VAL	24	CB	698	7.30E-01	-3.79E-01	-4.02E-01	-3.55E-01	2.31E-02
ASP	23	OD1	211	VAL	24	H	701	4.55E-01	-1.21E+00	-1.37E+00	-1.06E+00	1.57E-01
ASP	23	OD1	211	GLY	25	C	712	7.93E-01	-4.79E-01	-5.07E-01	-4.50E-01	2.89E-02
ASP	23	OD2	212	ALA	21	C	659	9.26E-01	-3.61E-01	-3.72E-01	-3.50E-01	1.11E-02
ASP	23	OD2	212	GLU	22	C	669	6.28E-01	-6.08E-01	-6.49E-01	-5.66E-01	4.14E-02
ASP	23	OD2	212	GLU	22	CD	673	7.47E-01	-7.05E-01	-7.57E-01	-6.54E-01	5.15E-02
ASP	23	OD2	212	ASP	23	C	684	5.68E-01	-7.19E-01	-7.88E-01	-6.49E-01	6.97E-02
ASP	23	OD2	212	ASP	23	CG	687	4.45E-01	-2.17E+00	-2.43E+00	-1.91E+00	2.62E-01
ASP	23	OD2	212	ASP	23	H	690	4.66E-01	-8.21E-01	-8.99E-01	-7.43E-01	7.81E-02
ASP	23	OD2	212	VAL	24	C	696	7.16E-01	-4.45E-01	-4.73E-01	-4.18E-01	2.78E-02
ASP	23	OD2	212	VAL	24	H	701	5.27E-01	-8.47E-01	-9.29E-01	-7.65E-01	8.22E-02
ASP	23	OD2	212	GLY	25	C	712	9.33E-01	-3.64E-01	-3.78E-01	-3.49E-01	1.45E-02
ASP	23	H	213	ASP	23	N	682	5.90E-01	-3.58E-01	-3.61E-01	-3.55E-01	3.17E-03
VAL	24	N	217	GLU	22	C	669	6.42E-01	-3.55E-01	-3.69E-01	-3.42E-01	1.34E-02
VAL	24	N	217	ASP	23	C	684	5.72E-01	-4.28E-01	-4.33E-01	-4.22E-01	5.44E-03
VAL	24	N	217	ASP	23	CG	687	7.26E-01	-4.41E-01	-4.49E-01	-4.33E-01	8.23E-03
VAL	24	N	217	ASP	23	H	690	4.71E-01	-4.87E-01	-5.15E-01	-4.58E-01	2.83E-02
VAL	24	N	217	VAL	24	C	696	5.35E-01	-5.01E-01	-5.10E-01	-4.93E-01	8.65E-03
VAL	24	N	217	VAL	24	CB	698	5.61E-01	-3.98E-01	-4.14E-01	-3.82E-01	1.59E-02
VAL	24	N	217	VAL	24	H	701	3.84E-01	-1.12E+00	-1.14E+00	-1.11E+00	1.43E-02
VAL	24	N	217	GLY	25	C	712	7.10E-01	-3.59E-01	-3.65E-01	-3.53E-01	5.94E-03
VAL	24	C	219	ASP	23	N	682	7.18E-01	-3.36E-01	-3.45E-01	-3.27E-01	9.22E-03
VAL	24	C	219	ASP	23	OD1	688	7.47E-01	-4.08E-01	-4.19E-01	-3.96E-01	1.18E-02
VAL	24	C	219	VAL	24	N	694	5.51E-01	-4.68E-01	-4.76E-01	-4.60E-01	8.05E-03
VAL	24	C	219	VAL	24	O	697	3.62E-01	-1.20E+00	-1.21E+00	-1.19E+00	1.01E-02
VAL	24	C	219	GLY	25	N	710	5.76E-01	-3.52E-01	-3.56E-01	-3.48E-01	3.71E-03
VAL	24	C	219	GLY	25	O	713	4.82E-01	-7.24E-01	-7.51E-01	-6.97E-01	2.70E-02
VAL	24	C	219	SER	26	N	717	6.20E-01	-4.36E-01	-4.51E-01	-4.21E-01	1.50E-02
VAL	24	O	220	VAL	24	C	696	6.05E-01	-3.43E-01	-3.44E-01	-3.41E-01	1.50E-03
VAL	24	O	220	VAL	24	H	701	5.80E-01	-3.70E-01	-3.77E-01	-3.63E-01	7.02E-03
VAL	24	O	220	GLY	25	C	712	6.45E-01	-3.91E-01	-3.96E-01	-3.86E-01	5.33E-03
VAL	24	CB	221	ASP	23	N	682	6.53E-01	-3.59E-01	-3.77E-01	-3.40E-01	1.86E-02
VAL	24	CB	221	VAL	24	N	694	5.21E-01	-4.72E-01	-4.94E-01	-4.50E-01	2.21E-02
VAL	24	CB	221	VAL	24	O	697	4.13E-01	-7.54E-01	-8.21E-01	-6.88E-01	6.65E-02
VAL	24	CB	221	GLY	25	O	713	6.04E-01	-3.85E-01	-4.04E-01	-3.66E-01	1.89E-02
VAL	24	CB	221	SER	26	N	717	6.62E-01	-3.38E-01	-3.52E-01	-3.23E-01	1.46E-02
VAL	24	CB	221	ASN	27	ND2	735	7.42E-01	-3.95E-01	-4.24E-01	-3.66E-01	2.91E-02
VAL	24	H	224	ASP	23	N	682	6.16E-01	-4.49E-01	-4.68E-01	-4.31E-01	1.83E-02
VAL	24	H	224	ASP	23	OD1	688	7.62E-01	-3.87E-01	-3.99E-01	-3.75E-01	1.18E-02

VAL	24	H	224	VAL	24	N	694	5.82E-01	-4.08E-01	-4.11E-01	-4.04E-01	3.73E-03
VAL	24	H	224	VAL	24	O	697	5.28E-01	-4.57E-01	-4.67E-01	-4.47E-01	9.99E-03
GLY	25	N	233	VAL	24	C	696	4.13E-01	-7.80E-01	-7.88E-01	-7.72E-01	8.24E-03
GLY	25	N	233	VAL	24	CB	698	5.54E-01	-3.40E-01	-3.51E-01	-3.29E-01	1.06E-02
GLY	25	N	233	VAL	24	H	701	4.71E-01	-5.54E-01	-5.76E-01	-5.31E-01	2.25E-02
GLY	25	N	233	GLY	25	C	712	4.25E-01	-9.44E-01	-9.73E-01	-9.15E-01	2.90E-02
GLY	25	C	235	ASP	23	OD1	688	9.39E-01	-3.60E-01	-3.69E-01	-3.50E-01	9.28E-03
GLY	25	C	235	VAL	24	N	694	7.72E-01	-3.07E-01	-3.11E-01	-3.03E-01	4.03E-03
GLY	25	C	235	VAL	24	O	697	4.98E-01	-6.94E-01	-7.06E-01	-6.82E-01	1.24E-02
GLY	25	C	235	GLY	25	N	710	6.43E-01	-3.63E-01	-3.67E-01	-3.59E-01	3.65E-03
GLY	25	C	235	GLY	25	O	713	3.71E-01	-1.83E+00	-1.88E+00	-1.78E+00	5.33E-02
GLY	25	C	235	SER	26	N	717	5.60E-01	-7.07E-01	-7.18E-01	-6.95E-01	1.20E-02
GLY	25	C	235	SER	26	O	720	7.67E-01	-4.01E-01	-4.08E-01	-3.94E-01	7.02E-03
GLY	25	C	235	SER	26	OG	722	6.70E-01	-5.94E-01	-6.82E-01	-5.05E-01	8.87E-02
GLY	25	C	235	ASN	27	N	728	6.51E-01	-4.07E-01	-4.19E-01	-3.96E-01	1.15E-02
GLY	25	C	235	ASN	27	O	731	8.00E-01	-3.36E-01	-3.41E-01	-3.30E-01	5.45E-03
GLY	25	C	235	ASN	27	ND2	735	9.72E-01	-3.65E-01	-3.75E-01	-3.55E-01	1.03E-02
GLY	25	O	236	GLY	25	C	712	5.98E-01	-5.75E-01	-5.81E-01	-5.68E-01	6.06E-03
GLY	25	H	237	VAL	24	N	694	4.51E-01	-4.28E-01	-4.43E-01	-4.13E-01	1.52E-02
GLY	25	H	237	VAL	24	O	697	1.92E-01	-4.30E+00	-4.48E+00	-4.13E+00	1.74E-01
GLY	25	H	237	GLY	25	N	710	3.85E-01	-5.35E-01	-5.43E-01	-5.27E-01	7.72E-03
GLY	25	H	237	GLY	25	O	713	3.08E-01	-1.36E+00	-1.46E+00	-1.25E+00	1.06E-01
GLY	25	H	237	SER	26	N	717	4.40E-01	-5.52E-01	-5.85E-01	-5.18E-01	3.36E-02
SER	26	N	240	VAL	24	C	696	5.84E-01	-4.95E-01	-5.07E-01	-4.83E-01	1.16E-02
SER	26	N	240	VAL	24	H	701	7.17E-01	-3.21E-01	-3.29E-01	-3.13E-01	8.27E-03
SER	26	N	240	GLY	25	C	712	4.41E-01	-1.24E+00	-1.28E+00	-1.21E+00	3.35E-02
SER	26	N	240	SER	26	C	719	5.44E-01	-6.27E-01	-6.42E-01	-6.11E-01	1.54E-02
SER	26	N	240	SER	26	H	723	5.85E-01	-3.80E-01	-3.84E-01	-3.77E-01	3.27E-03
SER	26	N	240	SER	26	HA	724	3.40E-01	-6.08E-01	-6.33E-01	-5.82E-01	2.53E-02
SER	26	N	240	SER	26	HG	727	6.09E-01	-4.76E-01	-5.63E-01	-3.88E-01	8.78E-02
SER	26	N	240	ASN	27	C	730	7.14E-01	-4.54E-01	-4.62E-01	-4.45E-01	8.50E-03
SER	26	N	240	ASN	27	CG	733	8.14E-01	-3.39E-01	-3.50E-01	-3.27E-01	1.16E-02
SER	26	N	240	ASN	27	H	736	4.43E-01	-5.42E-01	-5.68E-01	-5.16E-01	2.61E-02
SER	26	C	242	VAL	24	O	697	6.09E-01	-3.66E-01	-3.79E-01	-3.53E-01	1.31E-02
SER	26	C	242	GLY	25	O	713	4.98E-01	-7.27E-01	-7.68E-01	-6.86E-01	4.10E-02
SER	26	C	242	SER	26	N	717	5.54E-01	-6.02E-01	-6.14E-01	-5.89E-01	1.22E-02
SER	26	C	242	SER	26	O	720	6.11E-01	-5.20E-01	-5.24E-01	-5.16E-01	3.96E-03
SER	26	C	242	SER	26	OG	722	5.76E-01	-7.11E-01	-8.81E-01	-5.41E-01	1.70E-01
SER	26	C	242	ASN	27	N	728	4.18E-01	-9.43E-01	-9.58E-01	-9.27E-01	1.52E-02
SER	26	C	242	ASN	27	O	731	4.64E-01	-8.85E-01	-9.24E-01	-8.47E-01	3.89E-02
SER	26	C	242	ASN	27	OD1	734	7.34E-01	-3.29E-01	-3.37E-01	-3.21E-01	8.12E-03
SER	26	C	242	ASN	27	ND2	735	6.52E-01	-6.21E-01	-6.64E-01	-5.78E-01	4.32E-02

SER	26	C	242	LYS	28	N	742	6.65E-01	-3.29E-01	-3.35E-01	-3.22E-01	6.52E-03
SER	26	O	243	VAL	24	C	696	6.23E-01	-4.65E-01	-4.80E-01	-4.49E-01	1.56E-02
SER	26	O	243	VAL	24	CB	698	6.44E-01	-3.84E-01	-4.02E-01	-3.66E-01	1.78E-02
SER	26	O	243	GLY	25	C	712	4.92E-01	-1.03E+00	-1.07E+00	-9.83E-01	4.51E-02
SER	26	O	243	SER	26	CA	718	3.43E-01	-5.28E-01	-5.55E-01	-5.01E-01	2.69E-02
SER	26	O	243	SER	26	C	719	3.68E-01	-1.79E+00	-1.82E+00	-1.75E+00	3.18E-02
SER	26	O	243	SER	26	CB	721	3.97E-01	-4.49E-01	-4.94E-01	-4.03E-01	4.53E-02
SER	26	O	243	SER	26	H	723	5.36E-01	-4.97E-01	-5.06E-01	-4.87E-01	9.23E-03
SER	26	O	243	SER	26	HA	724	2.53E-01	-1.52E+00	-1.61E+00	-1.42E+00	9.43E-02
SER	26	O	243	SER	26	HG	727	5.41E-01	-6.63E-01	-7.84E-01	-5.41E-01	1.21E-01
SER	26	O	243	ASN	27	C	730	4.35E-01	-1.47E+00	-1.52E+00	-1.42E+00	5.39E-02
SER	26	O	243	ASN	27	CG	733	5.47E-01	-8.09E-01	-8.59E-01	-7.59E-01	4.97E-02
SER	26	O	243	ASN	27	H	736	1.96E-01	-5.84E+00	-6.21E+00	-5.46E+00	3.78E-01
SER	26	O	243	ASN	27	HD21	740	5.37E-01	-5.22E-01	-5.73E-01	-4.71E-01	5.09E-02
SER	26	O	243	LYS	28	C	744	7.81E-01	-4.84E-01	-4.94E-01	-4.75E-01	9.28E-03
SER	26	OG	245	GLY	25	C	712	6.25E-01	-7.23E-01	-8.77E-01	-5.68E-01	1.55E-01
SER	26	OG	245	SER	26	C	719	6.14E-01	-6.10E-01	-7.28E-01	-4.93E-01	1.17E-01
SER	26	OG	245	SER	26	HG	727	5.87E-01	-7.04E-01	-9.31E-01	-4.78E-01	2.27E-01
SER	26	OG	245	ASN	27	C	730	6.80E-01	-6.03E-01	-6.64E-01	-5.43E-01	6.04E-02
SER	26	OG	245	ASN	27	H	736	5.04E-01	-4.94E-01	-5.82E-01	-4.06E-01	8.81E-02
SER	26	H	246	VAL	24	O	697	4.01E-01	-7.07E-01	-7.43E-01	-6.71E-01	3.61E-02
SER	26	H	246	GLY	25	N	710	5.26E-01	-3.34E-01	-3.44E-01	-3.24E-01	1.03E-02
SER	26	H	246	GLY	25	O	713	2.67E-01	-2.86E+00	-3.30E+00	-2.43E+00	4.32E-01
SER	26	H	246	SER	26	N	717	3.96E-01	-9.73E-01	-9.96E-01	-9.50E-01	2.33E-02
SER	26	H	246	SER	26	O	720	5.72E-01	-4.29E-01	-4.39E-01	-4.19E-01	1.02E-02
SER	26	H	246	SER	26	OG	722	5.17E-01	-6.35E-01	-7.66E-01	-5.03E-01	1.32E-01
SER	26	H	246	ASN	27	N	728	4.52E-01	-5.53E-01	-5.83E-01	-5.24E-01	2.92E-02
SER	26	HG	250	GLY	25	O	713	5.39E-01	-8.10E-01	-1.20E+00	-4.19E-01	3.91E-01
SER	26	HG	250	SER	26	OG	722	5.43E-01	-1.13E+00	-2.00E+00	-2.67E-01	8.67E-01
ASN	27	N	251	GLY	25	C	712	6.93E-01	-3.60E-01	-3.69E-01	-3.50E-01	9.31E-03
ASN	27	N	251	SER	26	C	719	5.83E-01	-4.27E-01	-4.33E-01	-4.20E-01	6.55E-03
ASN	27	N	251	ASN	27	C	730	5.60E-01	-5.97E-01	-6.14E-01	-5.80E-01	1.72E-02
ASN	27	N	251	ASN	27	CG	733	6.25E-01	-4.47E-01	-4.68E-01	-4.27E-01	2.07E-02
ASN	27	N	251	ASN	27	H	736	3.91E-01	-5.90E-01	-6.00E-01	-5.80E-01	1.02E-02
ASN	27	C	253	GLY	25	O	713	8.17E-01	-3.34E-01	-3.42E-01	-3.26E-01	8.37E-03
ASN	27	C	253	SER	26	N	717	8.15E-01	-3.56E-01	-3.61E-01	-3.52E-01	4.39E-03
ASN	27	C	253	SER	26	O	720	7.55E-01	-4.38E-01	-4.41E-01	-4.35E-01	3.17E-03
ASN	27	C	253	SER	26	OG	722	7.61E-01	-5.00E-01	-5.85E-01	-4.15E-01	8.49E-02
ASN	27	C	253	ASN	27	N	728	5.40E-01	-6.47E-01	-6.59E-01	-6.35E-01	1.16E-02
ASN	27	C	253	ASN	27	O	731	3.80E-01	-1.89E+00	-1.98E+00	-1.79E+00	9.49E-02
ASN	27	C	253	ASN	27	OD1	734	6.67E-01	-5.05E-01	-5.21E-01	-4.88E-01	1.67E-02
ASN	27	C	253	ASN	27	ND2	735	5.33E-01	-1.29E+00	-1.46E+00	-1.12E+00	1.67E-01

ASN	27	C	253	LYS	28	N	742	5.91E-01	-5.36E-01	-5.46E-01	-5.27E-01	9.47E-03
ASN	27	C	253	LYS	28	O	745	8.20E-01	-3.67E-01	-3.75E-01	-3.60E-01	7.57E-03
ASN	27	C	253	GLY	29	N	764	6.86E-01	-3.40E-01	-3.53E-01	-3.27E-01	1.30E-02
ASN	27	O	254	SER	26	C	719	7.54E-01	-3.10E-01	-3.14E-01	-3.06E-01	3.53E-03
ASN	27	O	254	ASN	27	C	730	5.99E-01	-6.27E-01	-6.34E-01	-6.20E-01	7.01E-03
ASN	27	O	254	ASN	27	CG	733	6.88E-01	-4.50E-01	-4.77E-01	-4.24E-01	2.66E-02
ASN	27	O	254	LYS	28	C	744	8.02E-01	-4.17E-01	-4.25E-01	-4.10E-01	7.58E-03
ASN	27	CG	256	SER	26	O	720	8.39E-01	-3.46E-01	-3.62E-01	-3.30E-01	1.58E-02
ASN	27	CG	256	ASN	27	N	728	6.20E-01	-4.57E-01	-4.95E-01	-4.19E-01	3.78E-02
ASN	27	CG	256	ASN	27	O	731	5.25E-01	-8.29E-01	-9.68E-01	-6.90E-01	1.39E-01
ASN	27	CG	256	ASN	27	OD1	734	6.09E-01	-5.78E-01	-6.08E-01	-5.47E-01	3.06E-02
ASN	27	CG	256	ASN	27	ND2	735	4.23E-01	-2.08E+00	-2.30E+00	-1.85E+00	2.22E-01
ASN	27	CG	256	LYS	28	N	742	6.82E-01	-3.83E-01	-4.15E-01	-3.50E-01	3.24E-02
ASN	27	CG	256	ALA	30	O	774	7.29E-01	-4.29E-01	-4.65E-01	-3.93E-01	3.60E-02
ASN	27	OD1	257	SER	26	C	719	6.97E-01	-3.63E-01	-3.80E-01	-3.47E-01	1.64E-02
ASN	27	OD1	257	ASN	27	C	730	5.08E-01	-9.20E-01	-9.75E-01	-8.66E-01	5.45E-02
ASN	27	OD1	257	ASN	27	CG	733	3.91E-01	-1.67E+00	-1.72E+00	-1.61E+00	5.82E-02
ASN	27	OD1	257	ASN	27	H	736	5.20E-01	-3.61E-01	-3.88E-01	-3.34E-01	2.70E-02
ASN	27	OD1	257	ASN	27	HD21	740	2.16E-01	-7.14E+00	-8.53E+00	-5.75E+00	1.39E+00
ASN	27	OD1	257	ASN	27	HD22	741	3.57E-01	-1.39E+00	-1.59E+00	-1.19E+00	1.97E-01
ASN	27	OD1	257	LYS	28	C	744	6.65E-01	-5.99E-01	-6.24E-01	-5.73E-01	2.56E-02
ASN	27	OD1	257	GLY	29	C	766	6.96E-01	-4.40E-01	-4.66E-01	-4.14E-01	2.61E-02
ASN	27	OD1	257	ALA	30	C	773	7.08E-01	-4.19E-01	-4.50E-01	-3.87E-01	3.15E-02
ASN	27	OD1	257	ALA	30	H	776	5.58E-01	-3.55E-01	-3.86E-01	-3.23E-01	3.16E-02
ASN	27	ND2	258	SER	26	C	719	8.73E-01	-3.62E-01	-3.88E-01	-3.36E-01	2.64E-02
ASN	27	ND2	258	ASN	27	C	730	6.95E-01	-7.26E-01	-8.74E-01	-5.78E-01	1.48E-01
ASN	27	ND2	258	ASN	27	CG	733	5.97E-01	-8.97E-01	-9.48E-01	-8.46E-01	5.10E-02
ASN	27	ND2	258	ASN	27	HD21	740	4.26E-01	-1.28E+00	-1.45E+00	-1.12E+00	1.67E-01
ASN	27	ND2	258	ASN	27	HD22	741	5.43E-01	-6.78E-01	-7.29E-01	-6.27E-01	5.08E-02
ASN	27	ND2	258	LYS	28	C	744	8.36E-01	-6.08E-01	-7.23E-01	-4.93E-01	1.15E-01
ASN	27	ND2	258	GLY	29	C	766	8.47E-01	-4.81E-01	-5.82E-01	-3.80E-01	1.01E-01
ASN	27	ND2	258	ALA	30	C	773	8.12E-01	-4.96E-01	-5.73E-01	-4.19E-01	7.71E-02
ASN	27	HD21	263	ASN	27	ND2	735	6.05E-01	-5.31E-01	-5.61E-01	-5.02E-01	2.91E-02
ASN	27	HD22	264	ASN	27	ND2	735	4.97E-01	-8.25E-01	-8.68E-01	-7.82E-01	4.29E-02
LYS	28	N	265	SER	26	C	719	6.34E-01	-3.63E-01	-3.70E-01	-3.56E-01	6.90E-03
LYS	28	N	265	ASN	27	C	730	4.05E-01	-1.32E+00	-1.35E+00	-1.29E+00	2.94E-02
LYS	28	N	265	ASN	27	CG	733	4.97E-01	-7.78E-01	-8.63E-01	-6.93E-01	8.52E-02
LYS	28	N	265	ASN	27	H	736	4.61E-01	-3.96E-01	-4.13E-01	-3.79E-01	1.69E-02
LYS	28	N	265	LYS	28	C	744	5.81E-01	-6.55E-01	-6.78E-01	-6.32E-01	2.30E-02
LYS	28	N	265	GLY	29	C	766	7.40E-01	-3.23E-01	-3.33E-01	-3.12E-01	1.07E-02
LYS	28	N	265	GLY	29	H	768	4.68E-01	-3.81E-01	-4.06E-01	-3.56E-01	2.50E-02
LYS	28	C	267	SER	26	O	720	8.52E-01	-4.17E-01	-4.26E-01	-4.08E-01	8.63E-03

LYS	28	C	267	SER	26	OG	722	9.19E-01	-4.15E-01	-4.57E-01	-3.72E-01	4.22E-02
LYS	28	C	267	ASN	27	N	728	6.74E-01	-4.75E-01	-4.91E-01	-4.59E-01	1.59E-02
LYS	28	C	267	ASN	27	O	731	4.07E-01	-1.86E+00	-1.98E+00	-1.75E+00	1.18E-01
LYS	28	C	267	ASN	27	OD1	734	5.89E-01	-7.79E-01	-8.41E-01	-7.17E-01	6.20E-02
LYS	28	C	267	ASN	27	ND2	735	4.70E-01	-2.21E+00	-2.63E+00	-1.79E+00	4.18E-01
LYS	28	C	267	LYS	28	N	742	5.10E-01	-8.82E-01	-9.12E-01	-8.52E-01	3.00E-02
LYS	28	C	267	LYS	28	O	745	6.07E-01	-7.68E-01	-7.72E-01	-7.65E-01	3.44E-03
LYS	28	C	267	GLY	29	N	764	4.12E-01	-1.28E+00	-1.30E+00	-1.25E+00	2.40E-02
LYS	28	C	267	GLY	29	O	767	6.99E-01	-5.22E-01	-5.35E-01	-5.10E-01	1.23E-02
LYS	28	C	267	ALA	30	N	771	5.72E-01	-6.32E-01	-6.56E-01	-6.07E-01	2.46E-02
LYS	28	C	267	ALA	30	O	774	6.93E-01	-5.79E-01	-5.93E-01	-5.66E-01	1.36E-02
LYS	28	C	267	ILE	31	N	781	8.57E-01	-3.20E-01	-3.24E-01	-3.16E-01	4.02E-03
LYS	28	O	268	SER	26	C	719	7.39E-01	-3.47E-01	-3.57E-01	-3.37E-01	9.94E-03
LYS	28	O	268	ASN	27	C	730	4.11E-01	-1.67E+00	-1.78E+00	-1.55E+00	1.13E-01
LYS	28	O	268	ASN	27	CG	733	4.53E-01	-1.28E+00	-1.47E+00	-1.10E+00	1.88E-01
LYS	28	O	268	LYS	28	C	744	3.66E-01	-2.63E+00	-2.67E+00	-2.60E+00	3.32E-02
LYS	28	O	268	LYS	28	H	751	4.97E-01	-4.20E-01	-4.40E-01	-3.99E-01	2.04E-02
LYS	28	O	268	LYS	28	HA	752	2.55E-01	-1.31E+00	-1.39E+00	-1.22E+00	8.32E-02
LYS	28	O	268	GLY	29	C	766	4.79E-01	-1.06E+00	-1.11E+00	-1.02E+00	4.67E-02
LYS	28	O	268	GLY	29	H	768	1.93E-01	-5.90E+00	-6.26E+00	-5.53E+00	3.67E-01
LYS	28	O	268	ALA	30	C	773	6.67E-01	-4.98E-01	-5.11E-01	-4.85E-01	1.25E-02
LYS	28	O	268	ALA	30	H	776	4.08E-01	-8.08E-01	-8.73E-01	-7.44E-01	6.44E-02
LYS	28	H	274	ASN	27	N	728	4.37E-01	-4.40E-01	-4.64E-01	-4.15E-01	2.43E-02
LYS	28	H	274	ASN	27	O	731	2.20E-01	-3.89E+00	-4.64E+00	-3.14E+00	7.49E-01
LYS	28	H	274	ASN	27	OD1	734	4.87E-01	-4.14E-01	-4.44E-01	-3.84E-01	3.02E-02
LYS	28	H	274	ASN	27	ND2	735	3.76E-01	-1.38E+00	-1.65E+00	-1.11E+00	2.70E-01
LYS	28	H	274	LYS	28	N	742	4.04E-01	-5.43E-01	-5.68E-01	-5.18E-01	2.49E-02
GLY	29	N	287	ASN	27	C	730	6.11E-01	-4.30E-01	-4.45E-01	-4.15E-01	1.46E-02
GLY	29	N	287	LYS	28	C	744	5.79E-01	-5.66E-01	-5.72E-01	-5.60E-01	6.20E-03
GLY	29	N	287	GLY	29	C	766	6.24E-01	-3.86E-01	-3.93E-01	-3.79E-01	7.08E-03
GLY	29	N	287	GLY	29	H	768	3.85E-01	-5.33E-01	-5.40E-01	-5.26E-01	6.95E-03
GLY	29	N	287	ALA	30	H	776	4.99E-01	-3.24E-01	-3.38E-01	-3.10E-01	1.40E-02
GLY	29	C	289	ASN	27	O	731	6.34E-01	-5.26E-01	-5.50E-01	-5.02E-01	2.39E-02
GLY	29	C	289	ASN	27	OD1	734	6.07E-01	-5.90E-01	-6.53E-01	-5.27E-01	6.33E-02
GLY	29	C	289	ASN	27	ND2	735	4.70E-01	-1.79E+00	-2.22E+00	-1.37E+00	4.22E-01
GLY	29	C	289	LYS	28	N	742	6.70E-01	-3.90E-01	-4.03E-01	-3.76E-01	1.36E-02
GLY	29	C	289	LYS	28	O	745	6.67E-01	-5.08E-01	-5.22E-01	-4.94E-01	1.38E-02
GLY	29	C	289	GLY	29	N	764	4.54E-01	-8.04E-01	-8.41E-01	-7.68E-01	3.66E-02
GLY	29	C	289	GLY	29	O	767	6.06E-01	-5.59E-01	-5.63E-01	-5.55E-01	4.06E-03
GLY	29	C	289	ALA	30	N	771	4.10E-01	-1.12E+00	-1.14E+00	-1.10E+00	2.06E-02
GLY	29	C	289	ALA	30	O	774	4.23E-01	-1.43E+00	-1.53E+00	-1.33E+00	9.98E-02
GLY	29	C	289	ILE	31	N	781	6.00E-01	-5.06E-01	-5.17E-01	-4.95E-01	1.10E-02

GLY	29	C	289	ILE	31	O	784	8.75E-01	-3.41E-01	-3.47E-01	-3.36E-01	5.12E-03
GLY	29	O	290	ASN	27	C	730	6.20E-01	-5.70E-01	-6.04E-01	-5.35E-01	3.49E-02
GLY	29	O	290	ASN	27	CG	733	5.05E-01	-8.95E-01	-1.06E+00	-7.32E-01	1.63E-01
GLY	29	O	290	LYS	28	C	744	4.87E-01	-1.15E+00	-1.21E+00	-1.09E+00	5.96E-02
GLY	29	O	290	GLY	29	C	766	3.65E-01	-1.91E+00	-1.97E+00	-1.86E+00	5.37E-02
GLY	29	O	290	GLY	29	H	768	3.20E-01	-1.22E+00	-1.33E+00	-1.11E+00	1.07E-01
GLY	29	O	290	GLY	29	HA2	769	2.82E-01	-6.22E-01	-7.00E-01	-5.45E-01	7.75E-02
GLY	29	O	290	ALA	30	C	773	4.00E-01	-1.48E+00	-1.54E+00	-1.42E+00	6.26E-02
GLY	29	O	290	ALA	30	H	776	1.90E-01	-6.39E+00	-6.65E+00	-6.14E+00	2.53E-01
GLY	29	O	290	ILE	31	C	783	7.28E-01	-3.79E-01	-3.87E-01	-3.71E-01	7.86E-03
GLY	29	O	290	ILE	31	H	789	5.71E-01	-3.60E-01	-3.71E-01	-3.49E-01	1.11E-02
ALA	30	N	294	LYS	28	C	744	6.88E-01	-4.29E-01	-4.42E-01	-4.16E-01	1.33E-02
ALA	30	N	294	GLY	29	C	766	5.81E-01	-4.87E-01	-4.95E-01	-4.79E-01	7.92E-03
ALA	30	N	294	ALA	30	C	773	5.09E-01	-6.44E-01	-6.55E-01	-6.34E-01	1.04E-02
ALA	30	N	294	ALA	30	H	776	3.89E-01	-6.52E-01	-6.66E-01	-6.38E-01	1.38E-02
ALA	30	C	296	ASN	27	ND2	735	6.53E-01	-7.56E-01	-8.45E-01	-6.66E-01	8.94E-02
ALA	30	C	296	GLY	29	O	767	8.11E-01	-3.13E-01	-3.18E-01	-3.07E-01	5.13E-03
ALA	30	C	296	ALA	30	N	771	5.89E-01	-4.64E-01	-4.76E-01	-4.53E-01	1.19E-02
ALA	30	C	296	ALA	30	O	774	3.80E-01	-1.85E+00	-1.94E+00	-1.77E+00	8.29E-02
ALA	30	C	296	ILE	31	N	781	5.72E-01	-5.52E-01	-5.66E-01	-5.37E-01	1.44E-02
ALA	30	C	296	ILE	31	O	784	7.76E-01	-4.12E-01	-4.22E-01	-4.02E-01	1.03E-02
ALA	30	C	296	ILE	32	N	800	6.68E-01	-3.98E-01	-4.10E-01	-3.86E-01	1.20E-02
ALA	30	C	296	ILE	32	O	803	7.86E-01	-4.03E-01	-4.13E-01	-3.92E-01	1.10E-02
ALA	30	O	297	LYS	28	C	744	9.70E-01	-3.24E-01	-3.30E-01	-3.18E-01	6.21E-03
ALA	30	O	297	GLY	29	C	766	8.16E-01	-3.43E-01	-3.48E-01	-3.39E-01	4.82E-03
ALA	30	O	297	ALA	30	C	773	6.17E-01	-5.76E-01	-5.80E-01	-5.71E-01	4.68E-03
ALA	30	O	297	ILE	31	C	783	7.75E-01	-3.68E-01	-3.76E-01	-3.60E-01	7.89E-03
ALA	30	CB	298	ALA	30	C	773	4.84E-01	-4.33E-01	-5.02E-01	-3.63E-01	6.97E-02
ALA	30	H	299	ALA	30	O	774	5.07E-01	-4.62E-01	-4.76E-01	-4.48E-01	1.39E-02
ALA	30	HA	300	ALA	30	O	774	2.43E-01	-1.39E+00	-1.41E+00	-1.38E+00	1.57E-02
ILE	31	N	304	GLY	29	C	766	7.08E-01	-3.63E-01	-3.77E-01	-3.48E-01	1.44E-02
ILE	31	N	304	ALA	30	C	773	4.50E-01	-9.77E-01	-1.04E+00	-9.16E-01	6.08E-02
ILE	31	N	304	ALA	30	H	776	5.23E-01	-3.50E-01	-3.70E-01	-3.30E-01	2.00E-02
ILE	31	N	304	ILE	31	C	783	5.56E-01	-5.86E-01	-6.01E-01	-5.71E-01	1.51E-02
ILE	31	N	304	ILE	31	HA	790	3.55E-01	-5.51E-01	-5.72E-01	-5.30E-01	2.10E-02
ILE	31	N	304	ILE	32	C	802	7.08E-01	-3.55E-01	-3.66E-01	-3.43E-01	1.16E-02
ILE	31	N	304	ILE	32	H	808	4.63E-01	-5.23E-01	-5.47E-01	-4.99E-01	2.37E-02
ILE	31	C	306	ALA	30	O	774	4.85E-01	-1.00E+00	-1.10E+00	-9.08E-01	9.71E-02
ILE	31	C	306	ILE	31	N	781	5.61E-01	-5.76E-01	-5.91E-01	-5.60E-01	1.55E-02
ILE	31	C	306	ILE	31	O	784	6.19E-01	-6.37E-01	-6.40E-01	-6.34E-01	2.73E-03
ILE	31	C	306	ILE	32	N	800	4.28E-01	-1.10E+00	-1.12E+00	-1.07E+00	2.13E-02
ILE	31	C	306	ILE	32	O	803	4.43E-01	-1.39E+00	-1.46E+00	-1.31E+00	7.71E-02

ILE	31	C	306	GLY	33	N	819	6.45E-01	-3.54E-01	-3.67E-01	-3.41E-01	1.31E-02
ILE	31	O	307	GLY	29	C	766	8.19E-01	-3.82E-01	-3.96E-01	-3.67E-01	1.46E-02
ILE	31	O	307	ALA	30	C	773	4.87E-01	-1.10E+00	-1.17E+00	-1.03E+00	6.95E-02
ILE	31	O	307	ILE	31	C	783	3.77E-01	-2.10E+00	-2.14E+00	-2.07E+00	3.31E-02
ILE	31	O	307	ILE	31	H	789	5.48E-01	-4.81E-01	-4.90E-01	-4.72E-01	8.82E-03
ILE	31	O	307	ILE	31	HA	790	2.58E-01	-1.89E+00	-1.99E+00	-1.78E+00	1.05E-01
ILE	31	O	307	ILE	32	C	802	4.35E-01	-1.45E+00	-1.56E+00	-1.35E+00	1.09E-01
ILE	31	O	307	ILE	32	H	808	2.12E-01	-6.42E+00	-6.95E+00	-5.90E+00	5.27E-01
ILE	31	O	307	GLY	33	C	821	7.70E-01	-4.27E-01	-4.46E-01	-4.08E-01	1.88E-02
ILE	31	H	312	ALA	30	N	771	5.09E-01	-3.75E-01	-4.02E-01	-3.48E-01	2.69E-02
ILE	31	H	312	ALA	30	O	774	2.51E-01	-3.86E+00	-4.88E+00	-2.85E+00	1.01E+00
ILE	31	H	312	ILE	31	N	781	3.98E-01	-7.66E-01	-7.76E-01	-7.57E-01	9.23E-03
ILE	31	H	312	ILE	31	O	784	5.80E-01	-4.24E-01	-4.36E-01	-4.12E-01	1.19E-02
ILE	31	H	312	ILE	32	N	800	4.76E-01	-4.88E-01	-5.12E-01	-4.63E-01	2.43E-02
ILE	31	H	312	ILE	32	O	803	6.18E-01	-3.72E-01	-3.91E-01	-3.52E-01	1.93E-02
ILE	32	N	323	ALA	30	C	773	6.97E-01	-3.66E-01	-3.80E-01	-3.52E-01	1.38E-02
ILE	32	N	323	ILE	31	C	783	5.87E-01	-5.20E-01	-5.24E-01	-5.15E-01	4.64E-03
ILE	32	N	323	ILE	32	C	802	5.37E-01	-6.34E-01	-6.49E-01	-6.20E-01	1.48E-02
ILE	32	N	323	ILE	32	H	808	3.96E-01	-7.73E-01	-7.85E-01	-7.62E-01	1.14E-02
ILE	32	C	325	ALA	30	O	774	8.19E-01	-3.36E-01	-3.49E-01	-3.22E-01	1.38E-02
ILE	32	C	325	ILE	31	O	784	7.92E-01	-3.96E-01	-4.02E-01	-3.89E-01	6.67E-03
ILE	32	C	325	ILE	32	N	800	5.71E-01	-5.53E-01	-5.69E-01	-5.36E-01	1.67E-02
ILE	32	C	325	ILE	32	O	803	3.76E-01	-2.11E+00	-2.16E+00	-2.05E+00	5.22E-02
ILE	32	C	325	GLY	33	N	819	5.88E-01	-4.29E-01	-4.32E-01	-4.26E-01	2.98E-03
ILE	32	C	325	GLY	33	O	822	7.90E-01	-3.51E-01	-4.37E-01	-2.64E-01	8.66E-02
ILE	32	O	326	ILE	31	C	783	7.92E-01	-3.96E-01	-4.03E-01	-3.89E-01	7.12E-03
ILE	32	O	326	ILE	32	C	802	6.18E-01	-6.41E-01	-6.50E-01	-6.31E-01	9.58E-03
ILE	32	O	326	ILE	32	H	808	5.96E-01	-4.00E-01	-4.12E-01	-3.88E-01	1.23E-02
ILE	32	O	326	GLY	33	C	821	8.16E-01	-3.89E-01	-4.20E-01	-3.57E-01	3.17E-02
ILE	32	H	331	ILE	32	O	803	5.26E-01	-5.28E-01	-5.40E-01	-5.17E-01	1.15E-02
ILE	32	HA	332	ILE	32	N	800	3.75E-01	-4.76E-01	-4.95E-01	-4.56E-01	1.92E-02
ILE	32	HA	332	ILE	32	O	803	2.52E-01	-2.00E+00	-2.15E+00	-1.85E+00	1.48E-01
GLY	33	N	342	ILE	31	C	783	6.76E-01	-3.22E-01	-3.31E-01	-3.13E-01	8.92E-03
GLY	33	N	342	ILE	32	C	802	4.32E-01	-8.89E-01	-9.31E-01	-8.48E-01	4.16E-02
GLY	33	N	342	ILE	32	H	808	4.93E-01	-3.72E-01	-3.90E-01	-3.54E-01	1.78E-02
GLY	33	N	342	GLY	33	C	821	5.95E-01	-4.37E-01	-4.96E-01	-3.79E-01	5.83E-02
GLY	33	C	344	ILE	31	O	784	8.82E-01	-3.37E-01	-3.46E-01	-3.28E-01	9.32E-03
GLY	33	C	344	ILE	32	N	800	7.01E-01	-3.70E-01	-3.80E-01	-3.59E-01	1.06E-02
GLY	33	C	344	ILE	32	O	803	4.25E-01	-1.57E+00	-1.67E+00	-1.47E+00	9.99E-02
GLY	33	C	344	GLY	33	N	819	5.20E-01	-5.84E-01	-6.26E-01	-5.42E-01	4.17E-02
GLY	33	C	344	GLY	33	O	822	5.92E-01	-6.67E-01	-9.16E-01	-4.18E-01	2.49E-01
GLY	33	C	344	LEU	34	N	826	4.93E-01	-6.32E-01	-6.84E-01	-5.79E-01	5.25E-02

GLY	33	C	344	LEU	34	O	829	7.90E-01	-3.74E-01	-4.19E-01	-3.29E-01	4.49E-02
GLY	33	O	345	ILE	32	C	802	4.43E-01	-1.24E+00	-1.43E+00	-1.05E+00	1.89E-01
GLY	33	O	345	GLY	33	C	821	4.11E-01	-1.51E+00	-1.72E+00	-1.30E+00	2.12E-01
GLY	33	O	345	LEU	34	C	828	6.15E-01	-5.45E-01	-5.92E-01	-4.99E-01	4.65E-02
GLY	33	H	346	ILE	32	N	800	4.78E-01	-3.75E-01	-3.99E-01	-3.51E-01	2.38E-02
GLY	33	H	346	ILE	32	O	803	2.18E-01	-4.65E+00	-5.36E+00	-3.94E+00	7.10E-01
GLY	33	H	346	GLY	33	N	819	4.01E-01	-4.79E-01	-4.89E-01	-4.70E-01	9.63E-03
LEU	34	N	349	ILE	32	C	802	6.15E-01	-3.74E-01	-4.02E-01	-3.45E-01	2.85E-02
LEU	34	N	349	GLY	33	C	821	5.36E-01	-5.31E-01	-6.28E-01	-4.34E-01	9.71E-02
LEU	34	C	351	ILE	32	O	803	6.24E-01	-6.39E-01	-6.88E-01	-5.90E-01	4.91E-02
LEU	34	C	351	GLY	33	O	822	5.58E-01	-6.98E-01	-8.53E-01	-5.43E-01	1.55E-01
LEU	34	C	351	LEU	34	N	826	4.04E-01	-1.07E+00	-1.22E+00	-9.18E-01	1.50E-01
LEU	34	C	351	LEU	34	O	829	5.93E-01	-6.77E-01	-8.46E-01	-5.09E-01	1.68E-01
LEU	34	C	351	MET	35	N	845	4.42E-01	-9.40E-01	-1.06E+00	-8.22E-01	1.18E-01
LEU	34	C	351	MET	35	O	848	6.47E-01	-6.75E-01	-9.30E-01	-4.19E-01	2.55E-01
LEU	34	C	351	VAL	36	N	862	6.06E-01	-4.95E-01	-5.35E-01	-4.54E-01	4.06E-02
LEU	34	O	352	ILE	32	C	802	6.80E-01	-4.88E-01	-5.39E-01	-4.36E-01	5.13E-02
LEU	34	O	352	GLY	33	C	821	4.28E-01	-1.51E+00	-1.74E+00	-1.28E+00	2.27E-01
LEU	34	O	352	LEU	34	C	828	3.95E-01	-1.82E+00	-2.08E+00	-1.56E+00	2.63E-01
LEU	34	O	352	MET	35	C	847	5.04E-01	-1.07E+00	-1.27E+00	-8.67E-01	2.00E-01
LEU	34	O	352	VAL	36	H	869	4.87E-01	-9.11E-01	-1.16E+00	-6.65E-01	2.46E-01
MET	35	N	368	GLY	33	C	821	5.46E-01	-5.61E-01	-6.36E-01	-4.85E-01	7.55E-02
MET	35	N	368	LEU	34	C	828	5.60E-01	-5.42E-01	-6.72E-01	-4.11E-01	1.30E-01
MET	35	N	368	MET	35	C	847	6.00E-01	-4.70E-01	-5.29E-01	-4.11E-01	5.87E-02
MET	35	C	370	ILE	32	O	803	8.06E-01	-4.10E-01	-4.41E-01	-3.79E-01	3.11E-02
MET	35	C	370	GLY	33	O	822	7.18E-01	-4.27E-01	-4.96E-01	-3.58E-01	6.89E-02
MET	35	C	370	LEU	34	N	826	6.01E-01	-4.16E-01	-4.54E-01	-3.79E-01	3.74E-02
MET	35	C	370	LEU	34	O	829	6.54E-01	-7.68E-01	-1.30E+00	-2.36E-01	5.32E-01
MET	35	C	370	MET	35	N	845	4.81E-01	-8.01E-01	-9.29E-01	-6.73E-01	1.28E-01
MET	35	C	370	MET	35	O	848	5.33E-01	-1.11E+00	-1.57E+00	-6.47E-01	4.61E-01
MET	35	C	370	VAL	36	N	862	4.63E-01	-1.06E+00	-1.27E+00	-8.44E-01	2.13E-01
MET	35	C	370	GLY	37	N	878	6.44E-01	-3.74E-01	-3.89E-01	-3.60E-01	1.44E-02
MET	35	O	371	GLY	33	C	821	6.65E-01	-5.27E-01	-5.80E-01	-4.73E-01	5.38E-02
MET	35	O	371	LEU	34	C	828	5.61E-01	-9.25E-01	-1.18E+00	-6.67E-01	2.58E-01
MET	35	O	371	MET	35	C	847	4.39E-01	-1.71E+00	-2.18E+00	-1.25E+00	4.65E-01
MET	35	O	371	MET	35	H	853	4.20E-01	-1.10E+00	-1.53E+00	-6.73E-01	4.30E-01
VAL	36	N	385	LEU	34	C	828	6.46E-01	-4.37E-01	-4.90E-01	-3.83E-01	5.36E-02
VAL	36	N	385	MET	35	C	847	5.28E-01	-7.77E-01	-9.88E-01	-5.67E-01	2.11E-01
VAL	36	N	385	VAL	36	C	864	5.38E-01	-4.96E-01	-5.19E-01	-4.72E-01	2.36E-02
VAL	36	N	385	VAL	36	CB	866	5.46E-01	-4.24E-01	-4.45E-01	-4.03E-01	2.10E-02
VAL	36	N	385	VAL	36	H	869	4.46E-01	-8.94E-01	-1.11E+00	-6.82E-01	2.12E-01
VAL	36	C	387	VAL	36	N	862	5.50E-01	-4.73E-01	-4.99E-01	-4.47E-01	2.59E-02

VAL	36	C	387	VAL	36	O	865	4.37E-01	-9.25E-01	-1.17E+00	-6.75E-01	2.50E-01
VAL	36	C	387	GLY	37	N	878	5.29E-01	-4.77E-01	-6.04E-01	-3.49E-01	1.28E-01
VAL	36	O	388	VAL	36	C	864	5.32E-01	-5.91E-01	-8.36E-01	-3.46E-01	2.45E-01
VAL	36	O	388	VAL	36	H	869	5.66E-01	-3.97E-01	-4.36E-01	-3.59E-01	3.86E-02
VAL	36	CB	389	VAL	36	N	862	5.46E-01	-4.25E-01	-4.49E-01	-4.01E-01	2.40E-02
VAL	36	H	392	VAL	36	N	862	5.23E-01	-6.11E-01	-8.10E-01	-4.11E-01	1.99E-01
VAL	36	H	392	VAL	36	O	865	5.46E-01	-4.31E-01	-4.69E-01	-3.92E-01	3.86E-02
GLY	37	N	401	MET	35	C	847	6.63E-01	-3.54E-01	-3.67E-01	-3.40E-01	1.37E-02
GLY	37	N	401	VAL	36	C	864	4.64E-01	-6.41E-01	-7.64E-01	-5.18E-01	1.23E-01
GLY	37	N	401	GLY	37	C	880	5.62E-01	-4.91E-01	-5.32E-01	-4.49E-01	4.15E-02
GLY	37	C	403	MET	35	O	848	8.29E-01	-3.46E-01	-3.78E-01	-3.14E-01	3.19E-02
GLY	37	C	403	GLY	37	N	878	5.32E-01	-5.64E-01	-6.42E-01	-4.85E-01	7.83E-02
GLY	37	C	403	GLY	37	O	881	5.54E-01	-8.15E-01	-1.13E+00	-5.03E-01	3.13E-01
GLY	37	C	403	GLY	38	N	885	4.55E-01	-8.44E-01	-9.75E-01	-7.13E-01	1.31E-01
GLY	37	C	403	GLY	38	O	888	4.30E-01	-1.27E+00	-1.38E+00	-1.16E+00	1.10E-01
GLY	37	C	403	VAL	39	N	892	6.13E-01	-4.87E-01	-5.22E-01	-4.52E-01	3.49E-02
GLY	37	O	404	GLY	37	C	880	4.33E-01	-1.43E+00	-1.74E+00	-1.12E+00	3.10E-01
GLY	37	O	404	GLY	38	C	887	4.57E-01	-1.19E+00	-1.43E+00	-9.49E-01	2.41E-01
GLY	38	N	408	GLY	37	C	880	5.40E-01	-5.47E-01	-6.25E-01	-4.69E-01	7.77E-02
GLY	38	N	408	GLY	38	C	887	4.96E-01	-6.89E-01	-8.39E-01	-5.40E-01	1.49E-01
GLY	38	C	410	GLY	37	O	881	7.27E-01	-4.10E-01	-4.81E-01	-3.38E-01	7.18E-02
GLY	38	C	410	GLY	38	N	885	5.93E-01	-4.40E-01	-4.82E-01	-3.99E-01	4.14E-02
GLY	38	C	410	GLY	38	O	888	3.71E-01	-1.84E+00	-1.92E+00	-1.76E+00	7.92E-02
GLY	38	C	410	VAL	39	N	892	5.72E-01	-5.61E-01	-5.74E-01	-5.47E-01	1.36E-02
GLY	38	C	410	VAL	40	N	908	6.56E-01	-4.20E-01	-4.33E-01	-4.07E-01	1.31E-02
GLY	38	O	411	GLY	37	C	880	7.82E-01	-3.40E-01	-3.51E-01	-3.29E-01	1.06E-02
GLY	38	O	411	GLY	38	C	887	6.09E-01	-5.54E-01	-5.60E-01	-5.47E-01	6.35E-03
GLY	38	O	411	VAL	40	H	915	6.59E-01	-3.58E-01	-3.74E-01	-3.41E-01	1.66E-02
GLY	38	H	412	GLY	38	O	888	4.61E-01	-9.63E-01	-1.81E+00	-1.13E-01	8.51E-01
GLY	38	HA2	413	GLY	38	O	888	2.60E-01	-7.87E-01	-8.81E-01	-6.93E-01	9.42E-02
VAL	39	N	415	GLY	37	C	880	6.65E-01	-4.13E-01	-4.43E-01	-3.83E-01	2.97E-02
VAL	39	N	415	GLY	38	C	887	4.32E-01	-1.11E+00	-1.19E+00	-1.02E+00	8.68E-02
VAL	39	N	415	VAL	39	C	894	5.54E-01	-4.64E-01	-4.75E-01	-4.53E-01	1.10E-02
VAL	39	N	415	VAL	39	CB	896	5.18E-01	-4.95E-01	-5.70E-01	-4.20E-01	7.48E-02
VAL	39	N	415	VAL	39	H	899	5.88E-01	-4.00E-01	-4.07E-01	-3.92E-01	7.36E-03
VAL	39	N	415	VAL	40	H	915	4.52E-01	-7.38E-01	-7.66E-01	-7.10E-01	2.76E-02
VAL	39	C	417	GLY	38	O	888	4.53E-01	-8.68E-01	-9.74E-01	-7.62E-01	1.06E-01
VAL	39	C	417	VAL	39	N	892	5.43E-01	-4.86E-01	-5.04E-01	-4.67E-01	1.86E-02
VAL	39	C	417	VAL	39	O	895	6.09E-01	-3.39E-01	-3.41E-01	-3.36E-01	2.63E-03
VAL	39	C	417	VAL	40	N	908	4.15E-01	-9.31E-01	-9.60E-01	-9.03E-01	2.86E-02
VAL	39	C	417	VAL	40	O	911	4.45E-01	-7.05E-01	-7.43E-01	-6.67E-01	3.83E-02
VAL	39	O	418	GLY	38	C	887	4.61E-01	-8.46E-01	-9.17E-01	-7.75E-01	7.10E-02

VAL	39	O	418	VAL	39	C	894	3.68E-01	-1.15E+00	-1.16E+00	-1.14E+00	1.31E-02
VAL	39	O	418	VAL	39	CB	896	4.21E-01	-7.57E-01	-9.08E-01	-6.06E-01	1.51E-01
VAL	39	O	418	VAL	39	H	899	5.34E-01	-4.46E-01	-4.59E-01	-4.33E-01	1.32E-02
VAL	39	O	418	VAL	40	C	910	4.29E-01	-7.72E-01	-8.17E-01	-7.27E-01	4.48E-02
VAL	39	O	418	VAL	40	CB	912	4.19E-01	-7.42E-01	-8.33E-01	-6.50E-01	9.16E-02
VAL	39	O	418	VAL	40	H	915	1.97E-01	-6.94E+00	-7.46E+00	-6.42E+00	5.18E-01
VAL	39	CB	419	GLY	38	O	888	4.73E-01	-7.35E-01	-8.84E-01	-5.85E-01	1.50E-01
VAL	39	CB	419	VAL	40	N	908	5.54E-01	-4.18E-01	-4.62E-01	-3.74E-01	4.43E-02
VAL	39	H	422	GLY	38	N	885	4.98E-01	-4.97E-01	-5.54E-01	-4.40E-01	5.70E-02
VAL	39	H	422	GLY	38	O	888	2.27E-01	-6.52E+00	-8.20E+00	-4.83E+00	1.69E+00
VAL	39	H	422	VAL	39	N	892	3.92E-01	-1.06E+00	-1.10E+00	-1.03E+00	3.54E-02
VAL	39	H	422	VAL	39	O	895	5.85E-01	-3.65E-01	-3.77E-01	-3.53E-01	1.21E-02
VAL	39	H	422	VAL	40	N	908	4.75E-01	-6.53E-01	-6.80E-01	-6.27E-01	2.64E-02
VAL	40	N	431	GLY	38	C	887	6.68E-01	-4.07E-01	-4.28E-01	-3.86E-01	2.13E-02
VAL	40	N	431	VAL	39	C	894	5.81E-01	-4.17E-01	-4.22E-01	-4.13E-01	4.35E-03
VAL	40	N	431	VAL	40	C	910	5.32E-01	-5.07E-01	-5.21E-01	-4.93E-01	1.40E-02
VAL	40	N	431	VAL	40	CB	912	5.40E-01	-4.36E-01	-4.62E-01	-4.11E-01	2.57E-02
VAL	40	N	431	VAL	40	H	915	3.88E-01	-1.09E+00	-1.10E+00	-1.08E+00	1.12E-02
VAL	40	C	433	VAL	40	N	908	5.58E-01	-4.57E-01	-4.69E-01	-4.44E-01	1.27E-02
VAL	40	C	433	VAL	40	O	911	3.65E-01	-1.18E+00	-1.20E+00	-1.16E+00	1.77E-02
VAL	40	C	433	ILE	41	N	924	5.81E-01	-4.18E-01	-4.22E-01	-4.14E-01	3.63E-03
VAL	40	C	433	ILE	41	O	927	7.90E-01	-3.12E-01	-3.16E-01	-3.08E-01	3.79E-03
VAL	40	O	434	VAL	40	C	910	6.05E-01	-3.44E-01	-3.47E-01	-3.40E-01	3.51E-03
VAL	40	O	434	VAL	40	H	915	5.85E-01	-3.64E-01	-3.74E-01	-3.53E-01	1.02E-02
VAL	40	CB	435	VAL	40	N	908	5.53E-01	-4.11E-01	-4.26E-01	-3.96E-01	1.50E-02
VAL	40	CB	435	VAL	40	O	911	4.42E-01	-6.34E-01	-6.70E-01	-5.97E-01	3.69E-02
VAL	40	H	438	GLY	38	O	888	6.39E-01	-3.84E-01	-4.12E-01	-3.55E-01	2.86E-02
VAL	40	H	438	VAL	40	N	908	5.84E-01	-4.05E-01	-4.09E-01	-4.01E-01	3.61E-03
VAL	40	H	438	VAL	40	O	911	5.26E-01	-4.63E-01	-4.77E-01	-4.49E-01	1.41E-02
ILE	41	N	447	VAL	39	C	894	6.59E-01	-3.20E-01	-3.30E-01	-3.10E-01	9.87E-03
ILE	41	N	447	VAL	40	C	910	4.12E-01	-9.50E-01	-9.74E-01	-9.25E-01	2.45E-02
ILE	41	N	447	VAL	40	CB	912	5.36E-01	-4.47E-01	-4.79E-01	-4.15E-01	3.20E-02
ILE	41	N	447	VAL	40	H	915	4.79E-01	-6.43E-01	-6.74E-01	-6.12E-01	3.12E-02
ILE	41	N	447	ILE	41	C	926	5.60E-01	-5.77E-01	-5.87E-01	-5.66E-01	1.03E-02
ILE	41	N	447	ILE	41	HA	933	3.75E-01	-4.75E-01	-4.94E-01	-4.56E-01	1.90E-02
ILE	41	N	447	ALA	42	C	945	7.10E-01	-3.54E-01	-3.67E-01	-3.41E-01	1.30E-02
ILE	41	N	447	ALA	42	H	949	4.50E-01	-5.02E-01	-5.25E-01	-4.79E-01	2.30E-02
ILE	41	C	449	VAL	40	N	908	7.09E-01	-3.53E-01	-3.63E-01	-3.42E-01	1.02E-02
ILE	41	C	449	VAL	40	O	911	4.20E-01	-1.04E+00	-1.10E+00	-9.79E-01	6.01E-02
ILE	41	C	449	ILE	41	N	924	5.29E-01	-6.56E-01	-6.73E-01	-6.38E-01	1.78E-02
ILE	41	C	449	ILE	41	O	927	6.07E-01	-6.65E-01	-6.71E-01	-6.58E-01	6.25E-03
ILE	41	C	449	ALA	42	N	943	4.10E-01	-1.10E+00	-1.12E+00	-1.08E+00	2.09E-02

ILE	41	C	449	ALA	42	O	946	5.91E-01	-6.77E-01	-8.24E-01	-5.30E-01	1.47E-01
ILE	41	C	449	ALA	42	CB	947	4.95E-01	-3.97E-01	-4.33E-01	-3.61E-01	3.59E-02
ILE	41	C	449	ALA	42	OXT	948	5.00E-01	-9.53E-01	-1.09E+00	-8.17E-01	1.36E-01
ILE	41	O	450	VAL	39	C	894	7.76E-01	-3.23E-01	-3.32E-01	-3.13E-01	9.11E-03
ILE	41	O	450	VAL	40	C	910	4.36E-01	-1.14E+00	-1.21E+00	-1.06E+00	7.20E-02
ILE	41	O	450	VAL	40	CB	912	5.88E-01	-5.02E-01	-5.48E-01	-4.56E-01	4.57E-02
ILE	41	O	450	VAL	40	H	915	6.38E-01	-4.65E-01	-4.82E-01	-4.47E-01	1.76E-02
ILE	41	O	450	ILE	41	C	926	3.70E-01	-2.21E+00	-2.25E+00	-2.16E+00	4.29E-02
ILE	41	O	450	ILE	41	H	932	5.26E-01	-5.28E-01	-5.47E-01	-5.10E-01	1.86E-02
ILE	41	O	450	ILE	41	HA	933	2.63E-01	-1.79E+00	-1.96E+00	-1.62E+00	1.70E-01
ILE	41	O	450	ALA	42	C	945	4.27E-01	-1.54E+00	-1.67E+00	-1.41E+00	1.29E-01
ILE	41	O	450	ALA	42	H	949	1.96E-01	-7.14E+00	-7.58E+00	-6.69E+00	4.44E-01
ILE	41	H	455	VAL	40	N	908	4.56E-01	-5.42E-01	-5.68E-01	-5.16E-01	2.59E-02
ILE	41	H	455	VAL	40	O	911	1.94E-01	-5.38E+00	-5.73E+00	-5.03E+00	3.49E-01
ILE	41	H	455	ILE	41	N	924	3.90E-01	-8.09E-01	-8.23E-01	-7.95E-01	1.40E-02
ILE	41	H	455	ILE	41	O	927	5.91E-01	-4.08E-01	-4.15E-01	-4.00E-01	7.48E-03
ILE	41	H	455	ALA	42	N	943	4.71E-01	-4.50E-01	-4.68E-01	-4.31E-01	1.84E-02
ALA	42	N	466	ILE	41	C	926	5.81E-01	-4.77E-01	-4.84E-01	-4.70E-01	7.23E-03
ALA	42	N	466	ALA	42	C	945	5.31E-01	-5.96E-01	-6.53E-01	-5.39E-01	5.71E-02
ALA	42	N	466	ALA	42	H	949	3.88E-01	-6.54E-01	-6.70E-01	-6.38E-01	1.57E-02
ALA	42	C	468	ILE	41	O	927	7.86E-01	-4.06E-01	-4.33E-01	-3.79E-01	2.68E-02
ALA	42	C	468	ALA	42	N	943	5.64E-01	-5.24E-01	-5.87E-01	-4.60E-01	6.32E-02
ALA	42	C	468	ALA	42	O	946	5.31E-01	-8.15E-01	-8.99E-01	-7.31E-01	8.43E-02
ALA	42	C	468	ALA	42	OXT	948	4.52E-01	-1.20E+00	-1.33E+00	-1.07E+00	1.30E-01
ALA	42	O	469	ILE	41	C	926	7.01E-01	-4.49E-01	-4.86E-01	-4.13E-01	3.65E-02
ALA	42	O	469	ALA	42	C	945	4.64E-01	-1.11E+00	-1.19E+00	-1.03E+00	7.62E-02
ALA	42	O	469	ALA	42	H	949	5.17E-01	-4.51E-01	-5.06E-01	-3.96E-01	5.50E-02
ALA	42	OXT	471	ILE	41	C	926	7.63E-01	-3.89E-01	-4.35E-01	-3.44E-01	4.57E-02
ALA	42	OXT	471	ALA	42	C	945	5.89E-01	-6.42E-01	-6.91E-01	-5.94E-01	4.84E-02

Supplementary Table 2b: Mapping results for Aβ42's (PDB ID: 2MXU) short range (1:2) dominant atom-atom Lennard-Jones interactions across ensemble structures. Columns for each chain correspond to: residue abbreviation, residue number in peptide sequence, atom identity (IUPAC naming convention) and atom number in PDB file. Energy in *kT*, distance in *nm*. Mapping analysis began on the 11th residue for both isoforms because original structure data for Aβ42 begins with the 11th residue.

Chain A				Chain B				Average Distance	Average L-J Values	Lower 95% Confidence Interval Bound	Upper 95% Confidence Interval Bound	Margin of Error
LYS	16	C	85	LEU	17	N	582	4.10E-01	-1.59E-01	-1.63E-01	-1.56E-01	3.45E-03
LYS	16	O	86	LYS	16	CA	561	3.51E-01	-2.33E-01	-2.42E-01	-2.24E-01	9.07E-03
LYS	16	O	86	LYS	16	C	562	3.68E-01	-2.11E-01	-2.14E-01	-2.08E-01	3.12E-03
LYS	16	O	86	LEU	17	CA	583	3.90E-01	-2.04E-01	-2.18E-01	-1.90E-01	1.38E-02
LYS	16	O	86	LEU	17	C	584	4.08E-01	-1.53E-01	-1.67E-01	-1.39E-01	1.38E-02
LYS	16	O	86	LEU	17	O	585	3.53E-01	-3.05E-01	-3.26E-01	-2.84E-01	2.11E-02
LEU	17	N	105	LEU	17	O	585	4.35E-01	-1.41E-01	-1.49E-01	-1.33E-01	7.98E-03
LEU	17	CA	106	LEU	17	O	585	3.50E-01	-2.28E-01	-2.38E-01	-2.17E-01	1.04E-02
LEU	17	C	107	LEU	17	O	585	3.65E-01	-2.14E-01	-2.16E-01	-2.13E-01	1.39E-03
VAL	18	N	124	LEU	17	C	584	4.11E-01	-1.59E-01	-1.65E-01	-1.52E-01	6.53E-03
VAL	18	CA	125	LEU	17	O	585	3.82E-01	-2.15E-01	-2.30E-01	-1.99E-01	1.56E-02
VAL	18	C	126	LEU	17	O	585	4.20E-01	-1.35E-01	-1.49E-01	-1.20E-01	1.45E-02
VAL	18	C	126	PHE	19	N	617	4.11E-01	-1.58E-01	-1.63E-01	-1.52E-01	5.56E-03
VAL	18	O	127	LEU	17	O	585	3.72E-01	-2.59E-01	-2.85E-01	-2.32E-01	2.65E-02
VAL	18	O	127	VAL	18	CA	602	3.63E-01	-2.34E-01	-2.43E-01	-2.25E-01	9.02E-03
VAL	18	O	127	VAL	18	C	603	3.73E-01	-2.06E-01	-2.09E-01	-2.03E-01	2.93E-03
VAL	18	O	127	PHE	19	CA	618	3.81E-01	-2.15E-01	-2.29E-01	-2.00E-01	1.43E-02
VAL	18	CB	128	LEU	17	O	585	3.95E-01	-1.91E-01	-2.17E-01	-1.65E-01	2.59E-02
PHE	19	CA	141	PHE	19	CB	621	3.94E-01	-1.69E-01	-1.73E-01	-1.64E-01	4.51E-03
PHE	20	CA	161	PHE	20	O	640	3.51E-01	-2.36E-01	-2.43E-01	-2.29E-01	7.12E-03
PHE	20	C	162	PHE	20	O	640	3.73E-01	-2.07E-01	-2.09E-01	-2.04E-01	2.45E-03
PHE	20	CB	164	PHE	20	O	640	3.80E-01	-2.10E-01	-2.39E-01	-1.82E-01	2.83E-02
ALA	21	N	180	PHE	20	C	639	4.19E-01	-1.47E-01	-1.51E-01	-1.43E-01	3.67E-03
ALA	21	CA	181	PHE	20	O	640	3.91E-01	-2.03E-01	-2.10E-01	-1.96E-01	6.54E-03
ALA	21	CB	184	PHE	20	O	640	3.65E-01	-2.40E-01	-2.45E-01	-2.34E-01	5.36E-03
ALA	21	CB	184	ALA	21	CA	658	3.85E-01	-1.76E-01	-1.77E-01	-1.75E-01	9.19E-04
ALA	21	CB	184	ALA	21	C	659	3.75E-01	-1.55E-01	-1.56E-01	-1.53E-01	1.31E-03
ALA	21	CB	184	ALA	21	O	660	4.30E-01	-1.35E-01	-1.41E-01	-1.29E-01	6.19E-03
ALA	21	CB	184	GLU	22	N	667	3.66E-01	-2.16E-01	-2.19E-01	-2.12E-01	3.41E-03
ALA	21	CB	184	GLU	22	CA	668	4.16E-01	-1.49E-01	-1.56E-01	-1.41E-01	7.19E-03
GLU	22	N	190	GLU	22	CA	668	4.53E-01	-1.17E-01	-1.21E-01	-1.12E-01	4.60E-03
GLU	22	C	192	GLU	22	CA	668	4.38E-01	-1.07E-01	-1.09E-01	-1.06E-01	1.58E-03
GLU	22	C	192	ASP	23	N	682	4.37E-01	-1.23E-01	-1.29E-01	-1.18E-01	5.56E-03
GLU	22	O	193	GLU	22	N	667	4.43E-01	-1.28E-01	-1.32E-01	-1.23E-01	4.54E-03
GLU	22	O	193	GLU	22	C	669	3.75E-01	-2.04E-01	-2.07E-01	-2.01E-01	3.23E-03
GLU	22	O	193	GLU	22	CB	671	3.50E-01	-2.29E-01	-2.38E-01	-2.20E-01	8.86E-03

ASP	23	CA	206	ASP	23	N	682	4.59E-01	-1.10E-01	-1.14E-01	-1.05E-01	4.57E-03
ASP	23	C	207	ASP	23	N	682	4.24E-01	-1.41E-01	-1.47E-01	-1.34E-01	6.92E-03
ASP	23	C	207	ASP	23	CA	683	4.35E-01	-1.11E-01	-1.14E-01	-1.08E-01	2.74E-03
ASP	23	C	207	VAL	24	N	694	4.21E-01	-1.44E-01	-1.50E-01	-1.38E-01	5.64E-03
ASP	23	O	208	ASP	23	C	684	3.65E-01	-2.14E-01	-2.17E-01	-2.11E-01	2.84E-03
ASP	23	O	208	VAL	24	CA	695	4.13E-01	-1.64E-01	-1.79E-01	-1.49E-01	1.47E-02
ASP	23	O	208	VAL	24	O	697	3.90E-01	-2.14E-01	-2.32E-01	-1.96E-01	1.83E-02
ASP	23	CB	209	ASP	23	N	682	4.15E-01	-1.73E-01	-1.83E-01	-1.63E-01	9.61E-03
ASP	23	CB	209	ASP	23	CA	683	4.28E-01	-1.34E-01	-1.40E-01	-1.28E-01	5.68E-03
VAL	24	N	217	VAL	24	O	697	4.40E-01	-1.33E-01	-1.40E-01	-1.27E-01	6.61E-03
VAL	24	CA	218	VAL	24	N	694	4.55E-01	-1.15E-01	-1.20E-01	-1.09E-01	5.65E-03
VAL	24	CA	218	VAL	24	O	697	3.39E-01	-2.08E-01	-2.23E-01	-1.92E-01	1.58E-02
VAL	24	C	219	VAL	24	O	697	3.62E-01	-2.17E-01	-2.17E-01	-2.16E-01	5.58E-04
VAL	24	CB	221	VAL	24	O	697	4.13E-01	-1.62E-01	-1.80E-01	-1.43E-01	1.88E-02
GLY	25	N	233	VAL	24	C	696	4.13E-01	-1.55E-01	-1.57E-01	-1.53E-01	2.23E-03
GLY	25	N	233	GLY	25	C	712	4.25E-01	-1.39E-01	-1.45E-01	-1.32E-01	6.85E-03
GLY	25	N	233	GLY	25	O	713	3.56E-01	-2.96E-01	-3.01E-01	-2.90E-01	5.57E-03
GLY	25	CA	234	VAL	24	O	697	3.95E-01	-1.94E-01	-2.02E-01	-1.87E-01	7.62E-03
GLY	25	C	235	GLY	25	O	713	3.71E-01	-2.07E-01	-2.11E-01	-2.03E-01	3.97E-03
SER	26	N	240	GLY	25	C	712	4.41E-01	-1.17E-01	-1.23E-01	-1.12E-01	5.50E-03
SER	26	N	240	SER	26	CA	718	4.41E-01	-1.33E-01	-1.39E-01	-1.27E-01	5.98E-03
SER	26	C	242	ASN	27	N	728	4.18E-01	-1.49E-01	-1.52E-01	-1.45E-01	3.53E-03
SER	26	O	243	SER	26	CA	718	3.43E-01	-2.14E-01	-2.39E-01	-1.88E-01	2.51E-02
SER	26	O	243	SER	26	C	719	3.68E-01	-2.12E-01	-2.15E-01	-2.10E-01	2.53E-03
SER	26	O	243	SER	26	CB	721	3.97E-01	-1.88E-01	-2.08E-01	-1.68E-01	2.00E-02
SER	26	O	243	ASN	27	CA	729	3.93E-01	-1.97E-01	-2.08E-01	-1.87E-01	1.02E-02
SER	26	O	243	ASN	27	O	731	3.84E-01	-2.28E-01	-2.49E-01	-2.07E-01	2.10E-02
ASN	27	CA	252	ASN	27	O	731	4.03E-01	-1.78E-01	-2.00E-01	-1.56E-01	2.23E-02
ASN	27	C	253	ASN	27	O	731	3.80E-01	-1.95E-01	-2.05E-01	-1.85E-01	1.03E-02
ASN	27	OD1	257	ASN	27	CB	732	3.97E-01	-1.90E-01	-2.03E-01	-1.76E-01	1.33E-02
ASN	27	OD1	257	ASN	27	CG	733	3.91E-01	-1.80E-01	-1.88E-01	-1.71E-01	8.52E-03
LYS	28	N	265	ASN	27	C	730	4.05E-01	-1.67E-01	-1.71E-01	-1.62E-01	4.84E-03
LYS	28	CA	266	ASN	27	O	731	3.70E-01	-2.32E-01	-2.41E-01	-2.23E-01	9.02E-03
LYS	28	C	267	ASN	27	O	731	4.07E-01	-1.55E-01	-1.70E-01	-1.40E-01	1.49E-02
LYS	28	C	267	GLY	29	N	764	4.12E-01	-1.57E-01	-1.61E-01	-1.53E-01	4.04E-03
LYS	28	O	268	ASN	27	O	731	3.58E-01	-2.93E-01	-3.16E-01	-2.69E-01	2.35E-02
LYS	28	O	268	LYS	28	N	742	4.13E-01	-1.84E-01	-2.02E-01	-1.67E-01	1.74E-02
LYS	28	O	268	LYS	28	CA	743	3.46E-01	-2.29E-01	-2.38E-01	-2.19E-01	9.96E-03
LYS	28	O	268	LYS	28	C	744	3.66E-01	-2.14E-01	-2.15E-01	-2.13E-01	1.31E-03
LYS	28	O	268	GLY	29	CA	765	3.90E-01	-2.03E-01	-2.15E-01	-1.92E-01	1.18E-02
LYS	28	CB	269	ASN	27	O	731	3.65E-01	-2.22E-01	-2.41E-01	-2.04E-01	1.84E-02
GLY	29	C	289	ALA	30	N	771	4.10E-01	-1.59E-01	-1.63E-01	-1.55E-01	3.80E-03

GLY	29	O	290	GLY	29	N	764	3.68E-01	-2.78E-01	-2.99E-01	-2.57E-01	2.08E-02
GLY	29	O	290	GLY	29	CA	765	3.48E-01	-2.15E-01	-2.31E-01	-1.98E-01	1.66E-02
GLY	29	O	290	GLY	29	C	766	3.65E-01	-2.13E-01	-2.17E-01	-2.09E-01	4.19E-03
GLY	29	O	290	ALA	30	CA	772	3.86E-01	-2.09E-01	-2.23E-01	-1.95E-01	1.38E-02
GLY	29	O	290	ALA	30	C	773	4.00E-01	-1.65E-01	-1.74E-01	-1.55E-01	9.68E-03
GLY	29	O	290	ALA	30	O	774	3.54E-01	-2.97E-01	-3.19E-01	-2.75E-01	2.16E-02
ALA	30	N	294	ALA	30	O	774	4.16E-01	-1.76E-01	-1.87E-01	-1.65E-01	1.09E-02
ALA	30	C	296	ALA	30	O	774	3.80E-01	-1.96E-01	-2.05E-01	-1.88E-01	8.56E-03
ILE	31	C	306	ILE	32	N	800	4.28E-01	-1.35E-01	-1.39E-01	-1.31E-01	3.96E-03
ILE	31	O	307	ILE	31	CA	782	3.48E-01	-2.31E-01	-2.42E-01	-2.20E-01	1.11E-02
ILE	31	O	307	ILE	31	C	783	3.77E-01	-2.02E-01	-2.04E-01	-1.99E-01	2.96E-03
ILE	31	O	307	ILE	31	CB	785	3.94E-01	-1.87E-01	-2.15E-01	-1.59E-01	2.81E-02
ILE	31	O	307	ILE	32	CA	801	4.12E-01	-1.65E-01	-1.77E-01	-1.53E-01	1.19E-02
ILE	31	O	307	ILE	32	O	803	3.78E-01	-2.44E-01	-2.78E-01	-2.11E-01	3.35E-02
ILE	32	N	323	ILE	32	O	803	4.39E-01	-1.36E-01	-1.46E-01	-1.26E-01	9.79E-03
ILE	32	CA	324	ILE	32	O	803	3.49E-01	-2.33E-01	-2.40E-01	-2.25E-01	7.86E-03
ILE	32	C	325	ILE	32	O	803	3.76E-01	-2.01E-01	-2.05E-01	-1.97E-01	3.91E-03
GLY	33	N	342	ILE	32	C	802	4.32E-01	-1.29E-01	-1.39E-01	-1.20E-01	9.22E-03
GLY	33	CA	343	ILE	32	O	803	4.18E-01	-1.55E-01	-1.74E-01	-1.36E-01	1.89E-02
GLY	38	C	410	GLY	38	O	888	3.71E-01	-2.07E-01	-2.14E-01	-1.99E-01	7.82E-03
VAL	39	C	417	VAL	40	N	908	4.15E-01	-1.53E-01	-1.60E-01	-1.46E-01	6.61E-03
VAL	39	O	418	VAL	39	N	892	4.45E-01	-1.27E-01	-1.38E-01	-1.15E-01	1.13E-02
VAL	39	O	418	VAL	39	CA	893	3.47E-01	-2.20E-01	-2.36E-01	-2.05E-01	1.58E-02
VAL	39	O	418	VAL	39	C	894	3.68E-01	-2.12E-01	-2.14E-01	-2.10E-01	1.66E-03
VAL	39	O	418	VAL	40	CA	909	3.94E-01	-1.97E-01	-2.14E-01	-1.79E-01	1.75E-02
VAL	39	O	418	VAL	40	O	911	3.78E-01	-2.45E-01	-2.70E-01	-2.20E-01	2.53E-02
VAL	39	O	418	VAL	40	CB	912	4.19E-01	-1.57E-01	-1.86E-01	-1.27E-01	2.92E-02
VAL	40	CA	432	VAL	40	O	911	3.49E-01	-2.34E-01	-2.40E-01	-2.28E-01	6.02E-03
VAL	40	C	433	VAL	40	O	911	3.65E-01	-2.15E-01	-2.16E-01	-2.13E-01	1.74E-03
ILE	41	N	447	VAL	40	C	910	4.12E-01	-1.57E-01	-1.63E-01	-1.51E-01	5.66E-03
ILE	41	CA	448	VAL	40	O	911	3.86E-01	-2.11E-01	-2.24E-01	-1.98E-01	1.31E-02
ILE	41	C	449	VAL	40	O	911	4.20E-01	-1.34E-01	-1.47E-01	-1.22E-01	1.28E-02
ILE	41	C	449	ALA	42	N	943	4.10E-01	-1.60E-01	-1.63E-01	-1.56E-01	3.93E-03
ILE	41	O	450	VAL	40	O	911	3.66E-01	-2.73E-01	-2.96E-01	-2.49E-01	2.32E-02
ILE	41	O	450	ILE	41	N	924	4.35E-01	-1.42E-01	-1.54E-01	-1.30E-01	1.22E-02
ILE	41	O	450	ILE	41	CA	925	3.56E-01	-2.39E-01	-2.44E-01	-2.34E-01	4.80E-03
ILE	41	O	450	ILE	41	C	926	3.70E-01	-2.10E-01	-2.13E-01	-2.07E-01	2.80E-03
ILE	41	O	450	ALA	42	CA	944	3.83E-01	-2.16E-01	-2.25E-01	-2.06E-01	9.43E-03
ILE	41	O	450	ALA	42	CB	947	3.91E-01	-2.00E-01	-2.26E-01	-1.74E-01	2.61E-02
ILE	41	CB	451	VAL	40	O	911	4.07E-01	-1.75E-01	-1.97E-01	-1.54E-01	2.16E-02

Supplementary Table 3: Mapping results for Aβ40's (PDB ID: 2M4J) long range (1:3) dominant atom-atom Coulombic interactions across ensemble structures. Columns for each chain correspond to: residue abbreviation, residue number in peptide sequence, atom identity (IUPAC naming convention) and atom number in PDB file. Energy in *kT*, distance in *nm*. Mapping analysis began on the 11th residue for both isoforms because original structure data for Aβ42 begins with the 11th residue.

Chain A				Chain G				Average Distance	Average Coulombic Values	Lower 95% Confidence Interval Bound	Upper 95% Confidence Interval Bound	Margin of Error
GLU	11	O	155	GLU	11	CD	3752	9.93E-01	-3.53E-01	-3.55E-01	-3.50E-01	2.17E-03
GLU	11	O	155	VAL	12	H	3768	7.60E-01	-3.15E-01	-3.17E-01	-3.13E-01	2.34E-03
HIS	13	C	185	HIS	14	O	3797	8.65E-01	-3.38E-01	-3.39E-01	-3.36E-01	1.55E-03
HIS	13	O	186	HIS	13	C	3779	8.95E-01	-3.20E-01	-3.20E-01	-3.19E-01	9.70E-04
HIS	13	O	186	HIS	14	C	3796	8.32E-01	-3.61E-01	-3.63E-01	-3.58E-01	2.48E-03
HIS	14	C	202	HIS	14	O	3797	9.21E-01	-3.05E-01	-3.06E-01	-3.04E-01	7.57E-04
GLN	15	O	220	LYS	16	C	3830	8.92E-01	-3.76E-01	-3.81E-01	-3.71E-01	4.85E-03
LYS	16	C	236	LYS	16	O	3831	9.06E-01	-3.65E-01	-3.66E-01	-3.64E-01	1.04E-03
LEU	17	O	259	LYS	16	C	3830	8.99E-01	-3.66E-01	-3.69E-01	-3.63E-01	3.09E-03
GLU	22	CD	347	GLU	22	O	3938	9.63E-01	-3.83E-01	-4.30E-01	-3.35E-01	4.78E-02
ASP	23	O	359	ASP	23	CG	3955	8.79E-01	-3.51E-01	-3.55E-01	-3.47E-01	3.95E-03
ASP	23	CG	361	GLU	22	O	3938	8.98E-01	-4.01E-01	-4.12E-01	-3.91E-01	1.07E-02
ASP	23	OD2	363	ASP	23	CG	3955	9.80E-01	-4.31E-01	-4.42E-01	-4.19E-01	1.15E-02
SER	26	O	394	ASN	27	C	3998	9.12E-01	-3.17E-01	-3.23E-01	-3.11E-01	5.75E-03
LYS	28	O	419	LYS	28	C	4012	9.09E-01	-3.64E-01	-3.73E-01	-3.55E-01	8.57E-03
ILE	31	C	457	ILE	32	O	4071	8.73E-01	-3.36E-01	-3.42E-01	-3.29E-01	6.85E-03
ILE	31	O	458	ILE	31	C	4051	8.89E-01	-3.26E-01	-3.31E-01	-3.20E-01	5.17E-03
ILE	31	O	458	ILE	32	C	4070	8.66E-01	-3.40E-01	-3.47E-01	-3.33E-01	6.91E-03
LEU	34	O	503	GLY	33	C	4089	8.12E-01	-3.49E-01	-3.58E-01	-3.39E-01	9.19E-03

Supplementary Table 4: Mapping results for Aβ42's (PDB ID: 2MXU) long range (1:3) dominant atom-atom Coulombic interactions across ensemble structures. Columns for each chain correspond to: residue abbreviation, residue number in peptide sequence, atom identity (IUPAC naming convention) and atom number in PDB file. Energy in *kT*, distance in *nm*. Mapping analysis began on the 11th residue for both isoforms because original structure data for Aβ42 begins with the 11th residue.

Chain A				Chain C				Average Distance	Average Coulombic Values	Lower 95% Confidence Interval Bound	Upper 95% Confidence Interval Bound	Margin of Error
GLU	11	CD	7	GLU	11	OE1	962	9.76E-01	-5.04E-01	-5.21E-01	-4.87E-01	1.74E-02
GLU	11	CD	7	GLU	11	OE2	963	9.82E-01	-5.03E-01	-5.32E-01	-4.74E-01	2.87E-02
GLU	11	OE1	8	GLU	11	CD	961	9.51E-01	-5.26E-01	-5.51E-01	-5.00E-01	2.59E-02
GLU	11	OE2	9	GLU	11	CD	961	9.24E-01	-5.50E-01	-5.76E-01	-5.24E-01	2.57E-02
LYS	16	C	85	LEU	17	O	1062	8.18E-01	-4.29E-01	-4.36E-01	-4.23E-01	6.53E-03
LYS	16	O	86	LYS	16	C	1039	8.54E-01	-4.02E-01	-4.05E-01	-3.99E-01	2.71E-03
LYS	16	O	86	LEU	17	C	1061	8.07E-01	-3.50E-01	-3.56E-01	-3.45E-01	5.44E-03
LEU	17	C	107	LEU	17	O	1062	8.49E-01	-3.18E-01	-3.20E-01	-3.16E-01	2.01E-03
VAL	18	H	131	LEU	17	O	1062	6.70E-01	-3.77E-01	-3.82E-01	-3.71E-01	5.57E-03
GLU	22	O	193	GLU	22	CD	1150	9.23E-01	-3.95E-01	-4.08E-01	-3.83E-01	1.26E-02
GLU	22	CD	196	GLU	22	OE1	1151	9.60E-01	-5.17E-01	-5.29E-01	-5.04E-01	1.23E-02
GLU	22	CD	196	GLU	22	OE2	1152	9.72E-01	-5.07E-01	-5.19E-01	-4.94E-01	1.23E-02
GLU	22	OE1	197	GLU	22	CD	1150	9.95E-01	-4.89E-01	-5.06E-01	-4.73E-01	1.65E-02
GLU	22	OE2	198	GLU	22	CD	1150	9.94E-01	-4.90E-01	-5.03E-01	-4.76E-01	1.34E-02
ASP	23	O	208	ASP	23	CG	1164	9.49E-01	-3.11E-01	-3.15E-01	-3.07E-01	3.83E-03
ASP	23	O	208	VAL	24	H	1178	6.79E-01	-3.30E-01	-3.35E-01	-3.25E-01	5.24E-03
ASP	23	CG	210	ASP	23	N	1159	8.85E-01	-3.87E-01	-3.93E-01	-3.80E-01	6.60E-03
ASP	23	CG	210	ASP	23	OD1	1165	9.65E-01	-4.42E-01	-4.52E-01	-4.32E-01	1.05E-02
ASP	23	OD2	212	ASP	23	CG	1164	9.25E-01	-4.72E-01	-4.88E-01	-4.57E-01	1.56E-02
SER	26	O	243	GLY	25	C	1189	8.78E-01	-3.18E-01	-3.23E-01	-3.12E-01	5.56E-03
SER	26	O	243	ASN	27	C	1207	8.48E-01	-3.58E-01	-3.63E-01	-3.53E-01	4.90E-03
LYS	28	C	267	ASN	27	O	1208	7.95E-01	-4.23E-01	-4.33E-01	-4.14E-01	9.20E-03
LYS	28	C	267	ALA	30	O	1251	9.87E-01	-3.16E-01	-3.23E-01	-3.09E-01	6.66E-03
LYS	28	O	268	ASN	27	C	1207	7.94E-01	-3.89E-01	-3.99E-01	-3.79E-01	9.77E-03
LYS	28	O	268	LYS	28	C	1221	8.53E-01	-4.03E-01	-4.05E-01	-4.01E-01	1.77E-03
GLY	29	C	289	ALA	30	O	1251	8.11E-01	-3.47E-01	-3.55E-01	-3.40E-01	7.40E-03
GLY	29	O	290	LYS	28	C	1221	9.04E-01	-3.32E-01	-3.39E-01	-3.25E-01	6.80E-03
GLY	29	O	290	ALA	30	C	1250	7.97E-01	-3.22E-01	-3.27E-01	-3.18E-01	4.48E-03
ILE	31	C	306	ILE	32	O	1280	8.46E-01	-3.53E-01	-3.58E-01	-3.48E-01	5.16E-03
ILE	31	O	307	ALA	30	C	1250	8.87E-01	-3.28E-01	-3.36E-01	-3.19E-01	8.49E-03
ILE	31	O	307	ILE	31	C	1260	8.71E-01	-3.36E-01	-3.37E-01	-3.35E-01	1.11E-03
ILE	31	O	307	ILE	32	C	1279	8.43E-01	-3.56E-01	-3.62E-01	-3.50E-01	6.09E-03
ILE	32	C	325	ILE	32	O	1280	8.72E-01	-3.35E-01	-3.39E-01	-3.32E-01	3.19E-03
GLY	33	C	344	ILE	32	O	1280	8.22E-01	-3.80E-01	-3.92E-01	-3.68E-01	1.20E-02
ILE	41	O	450	ILE	41	C	1403	8.54E-01	-3.48E-01	-3.51E-01	-3.44E-01	3.31E-03
ILE	41	O	450	ALA	42	C	1422	8.24E-01	-3.73E-01	-3.92E-01	-3.53E-01	1.95E-02

Supplementary Table 5: Ramachandran angle data for Aβ40 and Aβ42 for chains A-D (Aβ40, PDB ID: 2M4J) and chains A-B (Aβ42, PDB ID: 2MXU) for the 1:2 interaction configuration across ensemble structures.

	Aβ40				Aβ42			
	Chain A		Chain D		Chain A		Chain B	
	φ	ψ	φ	ψ	φ	ψ	φ	ψ
GLU 11	-	152.910 ± 0.711	-	151.350 ± 0.487	-	85.980 ± 48.329	-	81.200 ± 48.360
VAL 12	-153.140 ± 0.201	128.500 ± 0.357	-149.530 ± 0.042	130.000 ± 0.072	-130.860 ± 4.541	132.920 ± 2.150	-126.470 ± 2.793	130.310 ± 3.024
HIS 13	-135.180 ± 0.296	146.43 ± 0.743	-129.880 ± 0.120	149.470 ± 0.774	-130.400 ± 4.486	138.120 ± 3.514	-126.780 ± 2.397	125.750 ± 2.675
HIS 14	-165.320 ± 0.569	97.570 ± 0.373	-165.060 ± 0.128	104.570 ± 0.113	-139.520 ± 1.844	135.130 ± 3.390	-128.600 ± 3.178	129.880 ± 3.248
GLN 15	-107.590 ± 2.008	-173.870 ± 0.751	-111.070 ± 2.030	-176.020 ± 0.175	-99.300 ± 49.056	121.450 ± 16.646	-96.660 ± 50.682	120.580 ± 8.974
LYS 16	-166.350 ± 1.700	97.950 ± 0.938	-162.970 ± 1.638	105.720 ± 0.414	-66.370 ± 51.461	74.450 ± 41.173	-62.910 ± 52.091	85.320 ± 40.351
LEU 17	-117.790 ± 5.123	158.580 ± 2.486	-124.310 ± 5.075	156.280 ± 2.852	-106.350 ± 8.094	128.920 ± 5.349	-119.950 ± 7.398	121.110 ± 6.335
VAL 18	-120.610 ± 9.229	124.710 ± 2.477	-112.980 ± 11.974	129.400 ± 2.469	-131.080 ± 1.889	121.890 ± 3.649	-127.230 ± 3.123	117.580 ± 2.471
PHE 19	-109.630 ± 16.980	133.770 ± 10.920	-110.030 ± 20.201	133.850 ± 7.521	-81.020 ± 3.879	158.850 ± 30.529	-76.250 ± 3.486	174.450 ± 0.906
PHE 20	-13.800 ± 67.407	34.610 ± 39.682	-12.140 ± 67.546	30.070 ± 41.404	-29.740 ± 43.085	146.220 ± 6.041	-45.570 ± 5.273	139.410 ± 5.855
ALA 21	-67.680 ± 12.282	-27.670 ± 50.357	-68.800 ± 13.231	-22.500 ± 50.612	-82.390 ± 2.151	-71.630 ± 106.672	-79.190 ± 1.994	-107.310 ± 93.302
GLU 22	-99.030 ± 13.950	117.940 ± 17.139	-102.000 ± 14.163	120.760 ± 16.175	-64.210 ± 1.979	164.500 ± 2.354	-67.340 ± 2.329	162.850 ± 2.212
ASP 23	-153.20 ± 5.623	137.940 ± 1.318	-148.460 ± 3.594	139.130 ± 1.409	56.740 ± 0.957	80.680 ± 7.616	59.740 ± 0.785	82.380 ± 1.732
VAL 24	-67.650 ± 8.347	79.540 ± 83.862	-70.530 ± 8.460	80.410 ± 85.689	-120.910 ± 5.218	129.850 ± 4.328	-126.500 ± 1.964	125.550 ± 1.838
GLY 25	-114.140 ± 3.094	50.210 ± 2.290	-114.730 ± 2.429	54.310 ± 3.261	-69.410 ± 1.837	-48.370 ± 3.181	-64.700 ± 0.905	-53.870 ± 3.950
SER 26	-67.480 ± 6.196	-79.970 ± 87.939	-61.550 ± 4.009	-83.240 ± 87.483	-151.180 ± 2.861	135.570 ± 0.234	-148.780 ± 2.944	135.650 ± 0.221
ASN 27	-69.580 ± 1.699	86.480 ± 5.961	-69.260 ± 3.501	91.230 ± 4.565	-99.240 ± 0.078	135.810 ± 2.815	-99.170 ± 0.106	132.980 ± 2.102
LYS 28	-82.690 ± 5.998	135.220 ± 7.876	-87.910 ± 5.247	137.660 ± 8.087	-133.890 ± 4.937	116.620 ± 2.013	-128.180 ± 4.710	115.470 ± 1.180
GLY 29	-62.690 ± 0.809	-153.170 ± 10.428	-60.700 ± 2.630	-151.460 ± 9.486	67.370 ± 1.613	83.100 ± 6.157	67.890 ± 1.968	86.690 ± 4.054
ALA 30	-104.030 ± 6.560	122.180 ± 4.709	-105.320 ± 5.670	123.850 ± 5.584	-131.240 ± 7.569	119.080 ± 4.617	-132.880 ± 8.833	115.120 ± 3.048
ILE 31	-100.480 ± 8.486	130.830 ± 4.794	-103.190 ± 8.379	137.610 ± 3.972	-143.340 ± 1.497	152.560 ± 5.527	-139.650 ± 2.449	149.700 ± 5.180
ILE 32	-147.380 ± 4.687	-78.070 ± 95.222	-152.980 ± 5.812	-77.810 ± 96.409	-119.270 ± 4.820	130.550 ± 6.560	-118.550 ± 2.265	126.340 ± 6.973
GLY 33	63.670 ± 0.773	21.120 ± 16.942	63.990 ± 0.473	20.500 ± 16.506	125.780 ± 69.398	54.270 ± 93.645	60.750 ± 102.966	52.780 ± 89.964
LEU 34	-83.930 ± 8.423	126.710 ± 3.472	-84.830 ± 7.589	134.400 ± 2.025	-72.280 ± 4.931	-36.940 ± 5.140	-72.560 ± 6.023	-39.810 ± 2.733
MET 35	-159.790 ± 2.975	150.550 ± 3.927	-157.400 ± 0.722	160.480 ± 2.626	-1.880 ± 56.148	76.720 ± 34.012	1.610 ± 55.991	80.430 ± 32.922
VAL 36	-154.990 ± 2.092	160.240 ± 4.458	-163.110 ± 1.703	161.350 ± 2.732	-122.660 ± 8.607	131.660 ± 5.849	-127.770 ± 3.156	121.400 ± 4.922
GLY 37	55.960 ± 0.472	69.450 ± 2.115	60.710 ± 0.370	64.440 ± 0.783	33.380 ± 78.191	-86.060 ± 80.912	44.710 ± 76.271	-71.030 ± 70.644
GLY 38	152.790 ± 7.822	7.410 ± 109.716	150.820 ± 7.315	8.980 ± 110.119	-73.180 ± 50.299	112.540 ± 28.090	-57.330 ± 76.223	114.640 ± 24.428
VAL 39	-122.680 ± 5.114	153.420 ± 4.657	-123.45 ± 4.172	151.310 ± 3.842	-130.890 ± 8.936	138.080 ± 7.006	-125.860 ± 5.951	134.690 ± 7.081
VAL 40	65.280 ± 0.705	-	73.420 ± 1.158	-	-121.460 ± 2.146	130.960 ± 2.269	-121.830 ± 2.170	125.290 ± 2.384
ILE 41	-	-	-	-	-126.650 ± 3.064	128.990 ± 6.494	-121.150 ± 3.527	121.410 ± 3.893
ALA 42	-	-	-	-	-114.710 ± 41.491	-	-121.570 ± 12.900	-

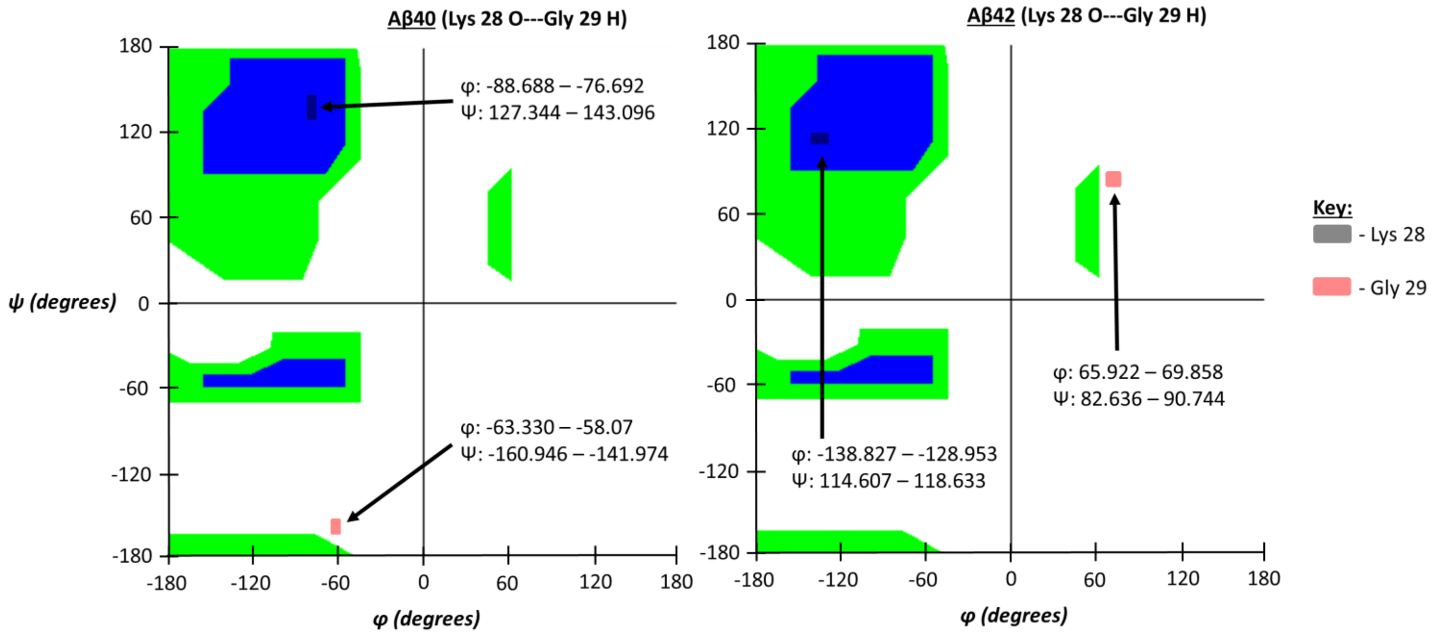

1a **1b**

Supplementary Figure 1a & 1b: Ramachandran angle profiles for an exceptionally strong atom-atom interaction (Lys 28 O interacting with Gly 29 H) for Aβ40 (PDB ID: 2M4J, 1a) and Aβ42 (PDB ID: 2MXU, 1b). Ranges for φ and ψ correspond to data spread according to 95% confidence interval analysis for all ensemble members as previously described. As stated before, the first atom is from the A chain of both isoforms and the second corresponds to the partner atom on the appropriate 1:2 interaction chain configuration. Note the increase in left-handed α-helix secondary structure in Aβ42 compared to Aβ40.

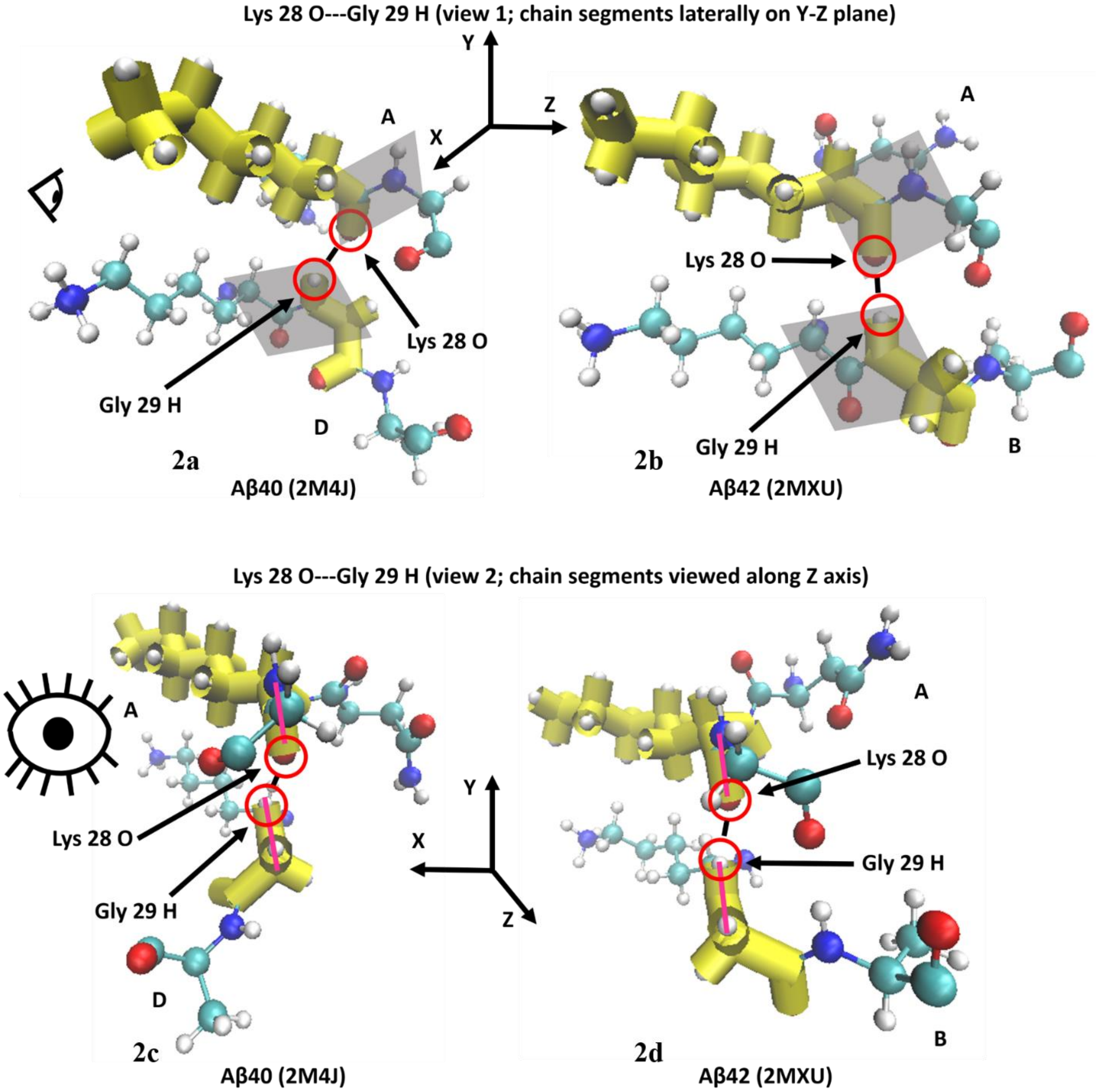

Supplementary Figure 2a-2d: Molecule representations of peptide plane alignment for Aβ40 (2a & 2c) and Aβ42 (2b & 2d). Shaded parallelograms in Figures 2a and 2b are the peptide planes for the residues whose atoms are participating in the hydrogen bonding. Figures 2c and 2d correspond to a view down the peptide bonds showing the peptide plane profile orientation in magenta. Eye icons indicate view perspective.

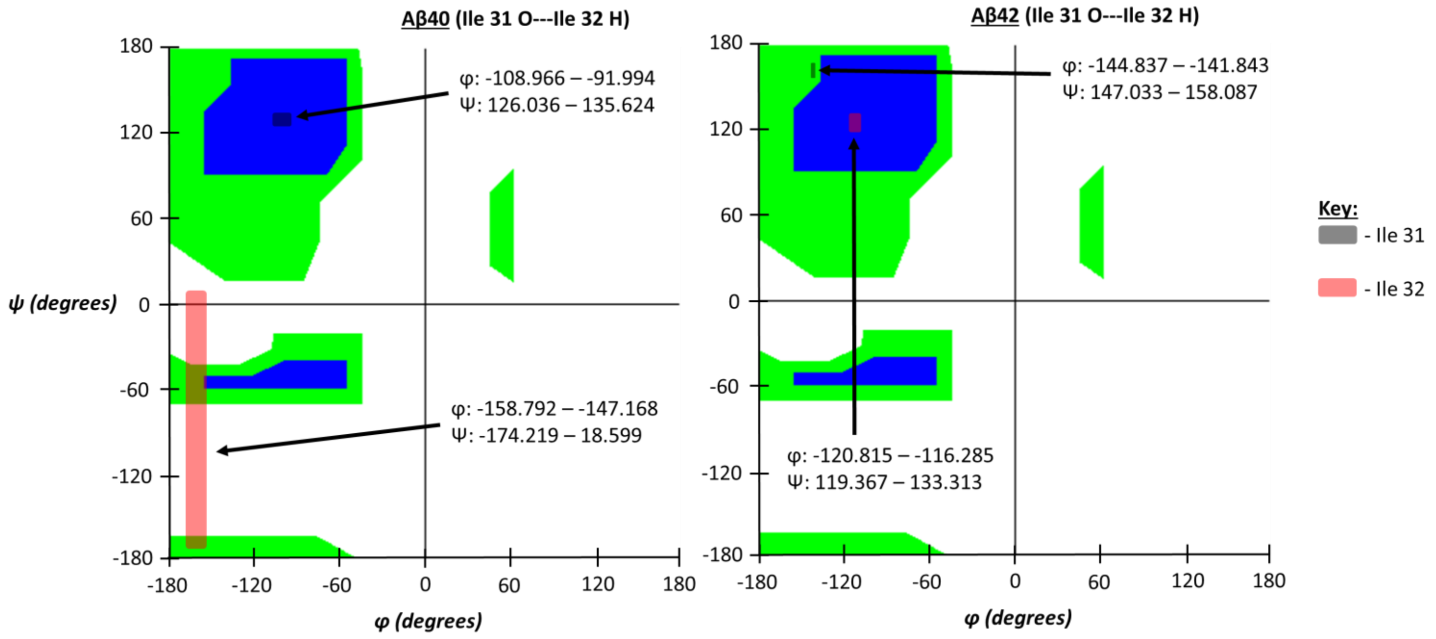

3a **3b**

Supplementary Figure 3a & 3b: Ramachandran angle profiles for an exceptionally strong atom-atom interaction (Ile 31 O interacting with Ile 32 H) for Aβ40 (PDB ID: 2M4J, 3a) and Aβ42 (PDB ID: 2MXU, 3b). Ranges for φ and ψ correspond to data spread according to 95% confidence interval analysis for all ensemble members as previously described. As stated before, the first atom is from the A chain of both isoforms and the second corresponds to the partner atom on the appropriate 1:2 interaction chain configuration. Note the decrease in β-sheet and increase in β-sheet Ramachandran angle values for Ile 31 and Ile 32 respectively in Aβ42 compared to Aβ40.

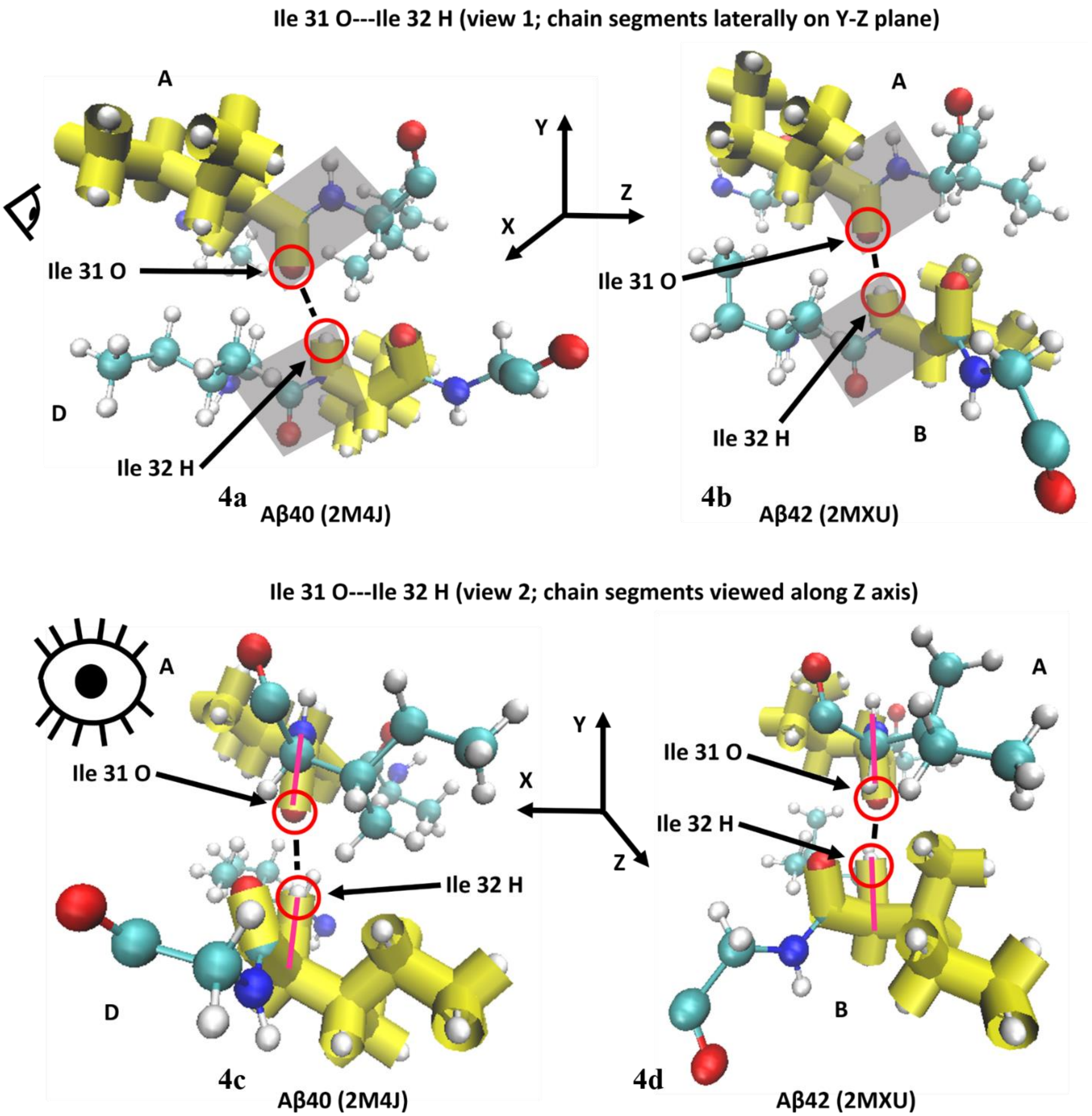

Supplementary Figure 4a-4d: Molecule representations of peptide plane alignment for Aβ40 (4a & 4c) and Aβ42 (4b & 4d). Shaded parallelograms in Figures 4a and 4b are the peptide planes for the residues whose atoms are participating in the hydrogen bonding. Figures 4c and 4d correspond to a view down the peptide bonds showing the peptide plane profile orientation in magenta. Eye icons indicate view perspective.

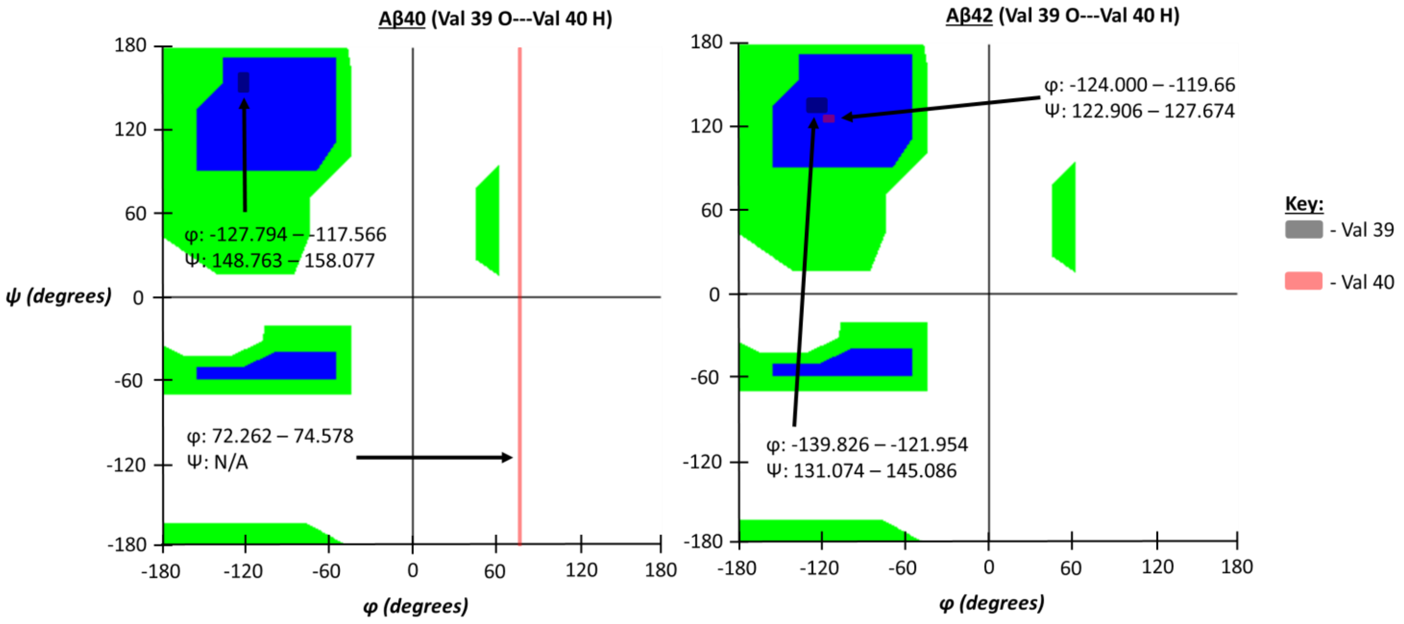

5a **5b**

Supplementary Figure 5a & 5b: Ramachandran angle profiles for an exceptionally strong atom-atom interaction (Val 39 O interacting with Val 40 H) for Aβ40 (PDB ID: 2M4J, 5a) and Aβ42 (PDB ID: 2MXU, 5b). Ranges for φ and ψ correspond to data spread according to 95% confidence interval analysis for all ensemble members as previously described. As stated before, the first atom is from the A chain of both isoforms and the second corresponds to the partner atom on the appropriate 1:2 interaction chain configuration. Note the retention of β-sheet secondary structure for Val 39 and the acquisition of β-sheet Ramachandran angle values for Val 40 in Aβ42 compared to Aβ40. We also note the lack of Ramachandran ψ angles in Aβ40 due to it being the C-terminus residue which lacks a peptide plane.

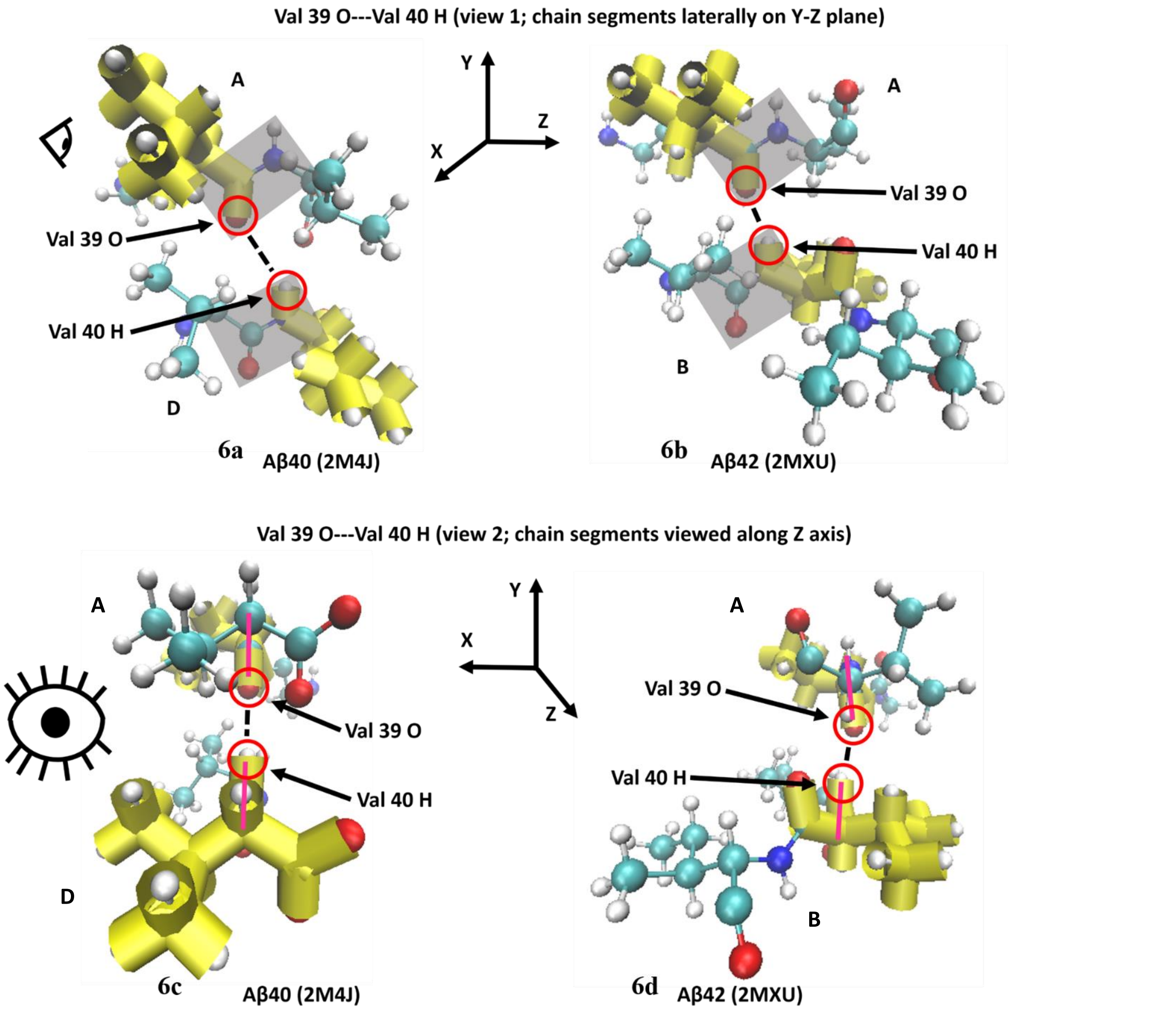

Supplementary Figure 6a-6d: Molecule representations of peptide plane alignment for Aβ40 (6a & 6c) and Aβ42 (6b & 6d). Shaded parallelograms in Figures 6a and 6b are the peptide planes for the residues whose atoms are participating in the hydrogen bonding. Figures 6c and 6d correspond to a view down the peptide bonds showing the peptide plane profile orientation in magenta. Eye icons indicate view perspective.

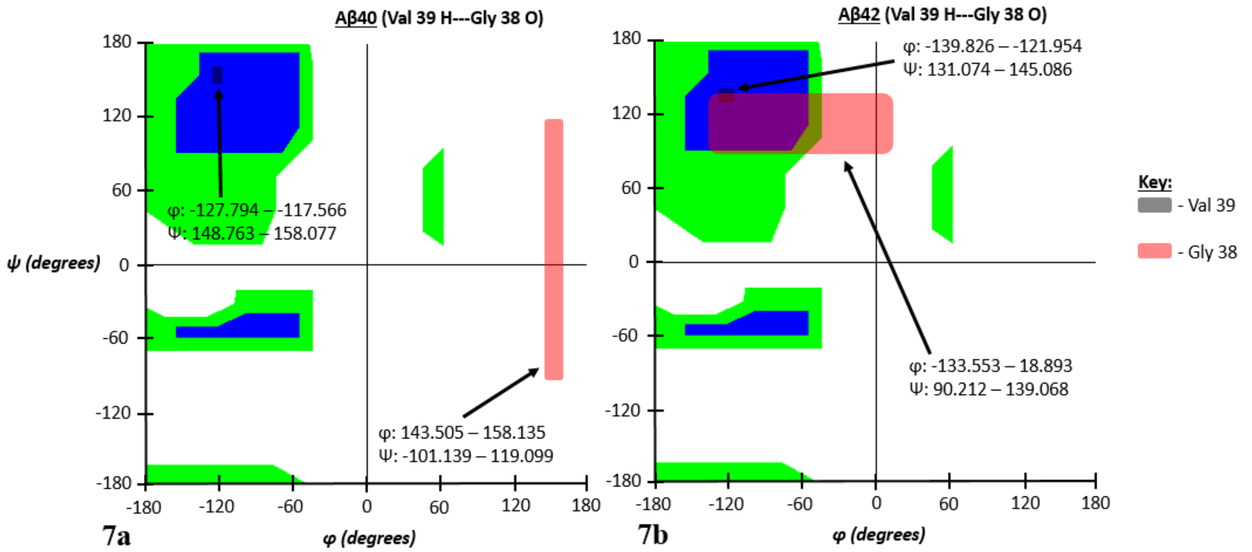

Supplementary Figure 7a & 7b: Ramachandran angle profiles for an exceptionally strong atom-atom interaction (Val 39 H interacting with Gly 38 O) for Aβ40 (PDB ID: 2M4J, 7a) and Aβ42 (PDB ID: 2MXU, 7b). Ranges for φ and ψ correspond to data spread according to 95% confidence interval analysis for all ensemble members as previously described. As stated before, the first atom is from the A chain of both isoforms and the second corresponds to the partner atom on the appropriate 1:2 interaction chain configuration. Note the retention of β-sheet secondary structure for Val 39 and the acquisition of β-sheet Ramachandran angle values for Gly 38 in Aβ42 compared to Aβ40.

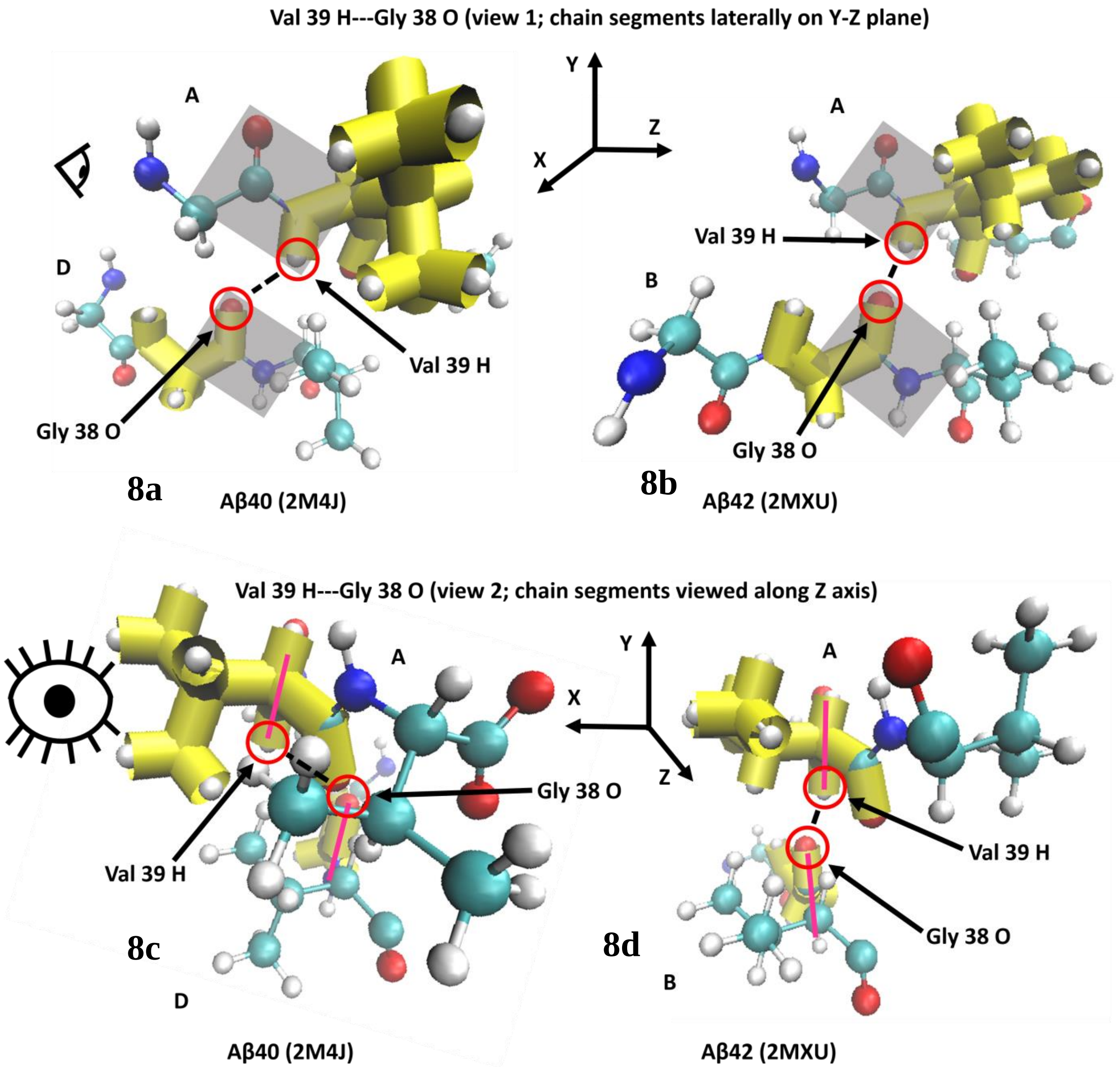

Supplementary Figure 8a-8d: Molecule representations of peptide plane alignment for Aβ40 (8a & 8c) and Aβ42 (8b & 8d). Shaded parallelograms in Figures 8a and 8b are the peptide planes for the residues whose atoms are participating in the hydrogen bonding. Figures 8c and 8d correspond to a view down the peptide bonds showing the peptide plane profile orientation in magenta. Eye icons indicate view perspective.

Supplementary Table 6a: Mapping results for Aβ42's (PDB ID: 2NAO by Walti et al.) short range (1:2) dominant atom-atom Coulombic interactions across ensemble structures. Columns for each chain correspond to: residue abbreviation, residue number in peptide sequence, atom identity (IUPAC naming convention) and atom number in PDB file. Energy in *kT*, distance in *nm*. Mapping analysis began on the 11th residue for both isoforms because original structure data for Aβ42 begins with the 11th residue.

Chain 1				Chain 2				Average Distance	Average Coulombic Values	Lower 95% Confidence Interval Bound	Upper 95% Confidence Interval Bound	Margin of Error
HIS	13	C	183	HIS	13	N	806	7.48E-01	-4.57E-01	-5.22E-01	-3.91E-01	6.51E-02
HIS	13	C	183	HIS	13	O	809	6.04E-01	-7.31E-01	-8.79E-01	-5.83E-01	1.48E-01
HIS	13	C	183	HIS	14	N	823	6.57E-01	-5.92E-01	-6.84E-01	-5.00E-01	9.22E-02
HIS	13	C	183	HIS	14	O	826	5.87E-01	-7.45E-01	-8.72E-01	-6.17E-01	1.28E-01
HIS	13	O	184	HIS	13	C	808	6.81E-01	-5.30E-01	-5.78E-01	-4.81E-01	4.85E-02
HIS	13	O	184	HIS	14	C	825	6.88E-01	-5.15E-01	-5.45E-01	-4.85E-01	3.00E-02
HIS	14	N	198	HIS	13	C	808	5.81E-01	-8.20E-01	-1.03E+00	-6.10E-01	2.11E-01
HIS	14	N	198	HIS	14	C	825	5.66E-01	-8.37E-01	-1.01E+00	-6.59E-01	1.78E-01
HIS	14	C	200	HIS	13	N	806	7.82E-01	-4.16E-01	-4.74E-01	-3.58E-01	5.78E-02
HIS	14	C	200	HIS	13	O	809	5.99E-01	-7.03E-01	-7.92E-01	-6.15E-01	8.87E-02
HIS	14	C	200	HIS	14	N	823	5.94E-01	-7.10E-01	-8.10E-01	-6.10E-01	9.98E-02
HIS	14	C	200	HIS	14	O	826	3.86E-01	-1.96E+00	-2.04E+00	-1.88E+00	8.00E-02
HIS	14	C	200	GLN	15	N	840	6.06E-01	-4.85E-01	-4.97E-01	-4.72E-01	1.28E-02
HIS	14	C	200	GLN	15	O	843	8.28E-01	-3.89E-01	-3.98E-01	-3.80E-01	8.79E-03
HIS	14	C	200	GLN	15	OE1	847	8.13E-01	-4.62E-01	-5.22E-01	-4.03E-01	5.94E-02
HIS	14	C	200	GLN	15	NE2	848	8.91E-01	-5.57E-01	-6.30E-01	-4.83E-01	7.38E-02
HIS	14	C	200	LYS	16	N	857	6.76E-01	-4.36E-01	-4.45E-01	-4.26E-01	9.60E-03
HIS	14	C	200	LYS	16	O	860	8.05E-01	-4.07E-01	-4.20E-01	-3.93E-01	1.32E-02
HIS	14	O	201	HIS	13	C	808	7.41E-01	-4.46E-01	-4.70E-01	-4.22E-01	2.42E-02
HIS	14	O	201	HIS	14	C	825	6.04E-01	-6.66E-01	-6.78E-01	-6.54E-01	1.24E-02
HIS	14	O	201	LYS	16	C	859	8.93E-01	-3.51E-01	-3.58E-01	-3.45E-01	6.77E-03
HIS	14	ND1	204	HIS	13	C	808	5.69E-01	-7.11E-01	-9.14E-01	-5.09E-01	2.03E-01
HIS	14	ND1	204	HIS	14	C	825	6.10E-01	-5.69E-01	-6.84E-01	-4.53E-01	1.16E-01
GLN	15	N	215	HIS	13	C	808	5.76E-01	-5.49E-01	-5.96E-01	-5.02E-01	4.69E-02
GLN	15	N	215	HIS	14	C	825	3.89E-01	-1.41E+00	-1.43E+00	-1.38E+00	2.63E-02
GLN	15	N	215	GLN	15	C	842	5.89E-01	-3.27E-01	-3.38E-01	-3.16E-01	1.08E-02
GLN	15	N	215	LYS	16	C	859	7.31E-01	-3.65E-01	-3.73E-01	-3.57E-01	7.79E-03
GLN	15	N	215	LYS	16	H	866	4.71E-01	-3.28E-01	-3.41E-01	-3.15E-01	1.31E-02
GLN	15	C	217	HIS	14	N	823	6.26E-01	-3.92E-01	-4.07E-01	-3.76E-01	1.52E-02
GLN	15	C	217	HIS	14	O	826	3.44E-01	-1.75E+00	-1.96E+00	-1.53E+00	2.15E-01
GLN	15	C	217	GLN	15	N	840	5.08E-01	-4.55E-01	-4.70E-01	-4.41E-01	1.47E-02
GLN	15	C	217	GLN	15	O	843	6.13E-01	-4.37E-01	-4.44E-01	-4.30E-01	6.83E-03
GLN	15	C	217	GLN	15	NE2	848	7.35E-01	-5.72E-01	-7.99E-01	-3.45E-01	2.27E-01
GLN	15	C	217	LYS	16	N	857	4.12E-01	-8.60E-01	-8.81E-01	-8.39E-01	2.09E-02
GLN	15	C	217	LYS	16	O	860	4.73E-01	-7.94E-01	-8.53E-01	-7.36E-01	5.87E-02
GLN	15	O	218	HIS	13	C	808	6.27E-01	-6.76E-01	-7.52E-01	-6.00E-01	7.58E-02
GLN	15	O	218	HIS	14	C	825	3.84E-01	-2.15E+00	-2.34E+00	-1.96E+00	1.90E-01
GLN	15	O	218	GLN	15	C	842	3.77E-01	-1.41E+00	-1.45E+00	-1.37E+00	4.26E-02

GLN	15	O	218	GLN	15	CD	846	5.89E-01	-8.59E-01	-1.08E+00	-6.41E-01	2.18E-01
GLN	15	O	218	GLN	15	H	849	5.10E-01	-4.74E-01	-4.90E-01	-4.57E-01	1.68E-02
GLN	15	O	218	GLN	15	HA	850	2.89E-01	-1.09E+00	-1.21E+00	-9.65E-01	1.25E-01
GLN	15	O	218	LYS	16	C	859	4.30E-01	-1.74E+00	-1.83E+00	-1.65E+00	8.91E-02
GLN	15	O	218	LYS	16	H	866	2.00E-01	-5.20E+00	-5.25E+00	-5.16E+00	4.67E-02
GLN	15	O	218	LEU	17	C	881	7.61E-01	-3.90E-01	-4.00E-01	-3.80E-01	9.98E-03
GLN	15	O	218	VAL	18	H	905	7.22E-01	-3.31E-01	-3.42E-01	-3.20E-01	1.13E-02
GLN	15	CD	221	HIS	14	N	823	6.85E-01	-6.92E-01	-1.01E+00	-3.78E-01	3.14E-01
GLN	15	CD	221	HIS	14	O	826	4.30E-01	-1.75E+00	-2.28E+00	-1.22E+00	5.28E-01
GLN	15	CD	221	GLN	15	N	840	5.36E-01	-7.21E-01	-8.99E-01	-5.42E-01	1.78E-01
GLN	15	CD	221	GLN	15	O	843	6.95E-01	-5.56E-01	-6.32E-01	-4.80E-01	7.63E-02
GLN	15	CD	221	GLN	15	OE1	847	4.80E-01	-1.57E+00	-2.02E+00	-1.12E+00	4.54E-01
GLN	15	CD	221	GLN	15	NE2	848	5.40E-01	-1.61E+00	-2.13E+00	-1.10E+00	5.14E-01
GLN	15	CD	221	LYS	16	N	857	5.71E-01	-6.84E-01	-8.62E-01	-5.07E-01	1.78E-01
GLN	15	CD	221	LYS	16	O	860	6.67E-01	-7.30E-01	-9.54E-01	-5.06E-01	2.24E-01
GLN	15	OE1	222	HIS	13	C	808	7.31E-01	-1.01E+00	-1.75E+00	-2.69E-01	7.42E-01
GLN	15	OE1	222	HIS	14	C	825	5.04E-01	-2.14E+00	-3.29E+00	-9.92E-01	1.15E+00
GLN	15	OE1	222	GLN	15	C	842	6.01E-01	-6.31E-01	-9.20E-01	-3.42E-01	2.89E-01
GLN	15	OE1	222	GLN	15	CD	846	5.03E-01	-1.33E+00	-1.63E+00	-1.04E+00	2.94E-01
GLN	15	OE1	222	GLN	15	HE22	856	6.02E-01	-8.64E-01	-1.64E+00	-8.86E-02	7.75E-01
GLN	15	OE1	222	LYS	16	C	859	7.09E-01	-8.47E-01	-1.29E+00	-4.09E-01	4.39E-01
GLN	15	NE2	223	HIS	13	C	808	7.80E-01	-8.24E-01	-1.13E+00	-5.16E-01	3.07E-01
GLN	15	NE2	223	HIS	14	C	825	5.39E-01	-1.72E+00	-2.37E+00	-1.07E+00	6.46E-01
GLN	15	NE2	223	GLN	15	C	842	5.95E-01	-7.92E-01	-9.43E-01	-6.41E-01	1.51E-01
GLN	15	NE2	223	GLN	15	CD	846	4.40E-01	-2.66E+00	-3.23E+00	-2.09E+00	5.71E-01
GLN	15	NE2	223	GLN	15	HE21	855	4.90E-01	-1.18E+00	-1.40E+00	-9.62E-01	2.21E-01
GLN	15	NE2	223	GLN	15	HE22	856	5.16E-01	-9.94E-01	-1.10E+00	-8.92E-01	1.02E-01
GLN	15	NE2	223	LYS	16	C	859	7.06E-01	-1.18E+00	-1.61E+00	-7.43E-01	4.32E-01
GLN	15	H	224	HIS	13	O	809	4.84E-01	-5.32E-01	-6.26E-01	-4.38E-01	9.39E-02
GLN	15	H	224	HIS	14	N	823	4.34E-01	-6.90E-01	-8.27E-01	-5.54E-01	1.37E-01
GLN	15	H	224	HIS	14	O	826	1.98E-01	-6.09E+00	-6.38E+00	-5.81E+00	2.85E-01
GLN	15	H	224	GLN	15	N	840	4.10E-01	-5.58E-01	-5.79E-01	-5.37E-01	2.12E-02
GLN	15	H	224	LYS	16	N	857	5.15E-01	-3.59E-01	-3.79E-01	-3.38E-01	2.03E-02
GLN	15	HA	225	HIS	14	O	826	4.26E-01	-3.49E-01	-3.61E-01	-3.36E-01	1.24E-02
GLN	15	HE21	230	HIS	14	O	826	4.95E-01	-9.66E-01	-1.69E+00	-2.39E-01	7.27E-01
GLN	15	HE21	230	GLN	15	NE2	848	4.78E-01	-1.27E+00	-1.55E+00	-9.98E-01	2.76E-01
GLN	15	HE22	231	HIS	14	O	826	5.24E-01	-7.24E-01	-1.10E+00	-3.48E-01	3.76E-01
GLN	15	HE22	231	GLN	15	OE1	847	4.28E-01	-1.74E+00	-2.56E+00	-9.22E-01	8.18E-01
GLN	15	HE22	231	GLN	15	NE2	848	4.57E-01	-1.40E+00	-1.67E+00	-1.13E+00	2.71E-01
LYS	16	N	232	HIS	14	C	825	5.80E-01	-6.02E-01	-6.31E-01	-5.74E-01	2.84E-02
LYS	16	N	232	GLN	15	C	842	5.95E-01	-3.59E-01	-3.67E-01	-3.52E-01	7.44E-03
LYS	16	N	232	LYS	16	C	859	5.58E-01	-7.15E-01	-7.32E-01	-6.99E-01	1.66E-02
LYS	16	N	232	LYS	16	H	866	4.03E-01	-5.47E-01	-5.62E-01	-5.31E-01	1.56E-02
LYS	16	C	234	HIS	14	O	826	6.68E-01	-5.97E-01	-6.30E-01	-5.65E-01	3.26E-02
LYS	16	C	234	GLN	15	O	843	7.72E-01	-4.81E-01	-4.94E-01	-4.69E-01	1.27E-02
LYS	16	C	234	LYS	16	N	857	5.49E-01	-7.44E-01	-7.79E-01	-7.08E-01	3.55E-02

LYS	16	C	234	LYS	16	O	860	3.86E-01	-2.27E+00	-2.30E+00	-2.25E+00	2.58E-02
LYS	16	C	234	LEU	17	N	879	6.07E-01	-4.85E-01	-4.90E-01	-4.80E-01	4.64E-03
LYS	16	C	234	LEU	17	O	882	8.29E-01	-4.20E-01	-4.27E-01	-4.12E-01	7.53E-03
LYS	16	C	234	VAL	18	N	898	6.68E-01	-5.05E-01	-5.22E-01	-4.88E-01	1.72E-02
LYS	16	C	234	VAL	18	O	901	7.05E-01	-4.09E-01	-4.25E-01	-3.94E-01	1.54E-02
LYS	16	O	235	HIS	14	C	825	8.61E-01	-3.63E-01	-3.73E-01	-3.53E-01	1.00E-02
LYS	16	O	235	LYS	16	C	859	6.14E-01	-7.52E-01	-7.68E-01	-7.37E-01	1.53E-02
LYS	16	O	235	LEU	17	C	881	8.01E-01	-3.55E-01	-3.59E-01	-3.50E-01	4.52E-03
LYS	16	O	235	VAL	18	H	905	6.54E-01	-4.00E-01	-4.10E-01	-3.89E-01	1.04E-02
LYS	16	HA	242	LYS	16	O	860	3.26E-01	-6.67E-01	-7.67E-01	-5.67E-01	1.00E-01
LEU	17	N	254	LYS	16	C	859	4.02E-01	-1.30E+00	-1.38E+00	-1.23E+00	7.32E-02
LEU	17	N	254	LEU	17	C	881	5.86E-01	-4.15E-01	-4.28E-01	-4.02E-01	1.32E-02
LEU	17	N	254	VAL	18	H	905	4.58E-01	-5.69E-01	-6.13E-01	-5.26E-01	4.32E-02
LEU	17	C	256	LYS	16	N	857	6.61E-01	-4.00E-01	-4.30E-01	-3.69E-01	3.06E-02
LEU	17	C	256	LYS	16	O	860	3.70E-01	-2.16E+00	-2.57E+00	-1.74E+00	4.17E-01
LEU	17	C	256	LEU	17	N	879	5.25E-01	-5.33E-01	-5.70E-01	-4.96E-01	3.70E-02
LEU	17	C	256	LEU	17	O	882	6.23E-01	-5.71E-01	-5.77E-01	-5.65E-01	5.70E-03
LEU	17	C	256	VAL	18	N	898	4.21E-01	-1.15E+00	-1.16E+00	-1.14E+00	1.01E-02
LEU	17	C	256	VAL	18	O	901	3.84E-01	-1.34E+00	-1.46E+00	-1.21E+00	1.24E-01
LEU	17	C	256	PHE	19	N	914	5.85E-01	-4.36E-01	-4.57E-01	-4.16E-01	2.06E-02
LEU	17	C	256	ILE	31	O	1081	8.54E-01	-3.51E-01	-3.62E-01	-3.39E-01	1.18E-02
LEU	17	C	256	ILE	32	O	1100	7.56E-01	-4.38E-01	-4.71E-01	-4.06E-01	3.24E-02
LEU	17	O	257	LYS	16	C	859	4.15E-01	-1.96E+00	-2.26E+00	-1.65E+00	3.05E-01
LEU	17	O	257	LEU	17	C	881	3.84E-01	-1.81E+00	-1.88E+00	-1.75E+00	6.44E-02
LEU	17	O	257	LEU	17	H	887	5.30E-01	-3.75E-01	-4.02E-01	-3.48E-01	2.67E-02
LEU	17	O	257	LEU	17	HA	888	2.86E-01	-1.01E+00	-1.16E+00	-8.61E-01	1.51E-01
LEU	17	O	257	VAL	18	C	900	3.73E-01	-1.55E+00	-1.70E+00	-1.40E+00	1.53E-01
LEU	17	O	257	VAL	18	CB	902	4.54E-01	-8.21E-01	-8.83E-01	-7.59E-01	6.21E-02
LEU	17	O	257	VAL	18	H	905	2.02E-01	-8.74E+00	-8.83E+00	-8.64E+00	9.96E-02
LEU	17	O	257	PHE	19	C	916	6.50E-01	-5.03E-01	-5.32E-01	-4.74E-01	2.90E-02
LEU	17	O	257	ILE	32	C	1099	7.32E-01	-4.14E-01	-4.34E-01	-3.95E-01	1.96E-02
LEU	17	CB	258	LYS	16	C	859	4.47E-01	-4.07E-01	-4.46E-01	-3.67E-01	3.94E-02
LEU	17	H	262	LYS	16	N	857	4.10E-01	-5.65E-01	-6.44E-01	-4.87E-01	7.83E-02
LEU	17	H	262	LYS	16	O	860	1.98E-01	-5.58E+00	-5.80E+00	-5.36E+00	2.18E-01
LEU	17	H	262	LEU	17	N	879	4.11E-01	-4.42E-01	-4.49E-01	-4.35E-01	6.74E-03
VAL	18	N	273	LYS	16	C	859	5.99E-01	-6.41E-01	-6.94E-01	-5.89E-01	5.25E-02
VAL	18	N	273	LEU	17	C	881	6.06E-01	-4.89E-01	-5.00E-01	-4.77E-01	1.12E-02
VAL	18	N	273	VAL	18	C	900	5.01E-01	-5.84E-01	-6.02E-01	-5.65E-01	1.88E-02
VAL	18	N	273	VAL	18	H	905	4.13E-01	-9.28E-01	-9.55E-01	-9.01E-01	2.70E-02
VAL	18	N	273	PHE	19	C	916	6.77E-01	-3.74E-01	-3.94E-01	-3.54E-01	1.98E-02
VAL	18	C	275	VAL	18	N	898	6.26E-01	-3.56E-01	-3.66E-01	-3.46E-01	1.01E-02
VAL	18	C	275	VAL	18	O	901	4.18E-01	-8.28E-01	-8.77E-01	-7.80E-01	4.86E-02
VAL	18	C	275	PHE	19	N	914	5.59E-01	-3.74E-01	-3.88E-01	-3.60E-01	1.40E-02
VAL	18	C	275	PHE	19	O	917	5.66E-01	-5.47E-01	-6.58E-01	-4.35E-01	1.11E-01
VAL	18	O	276	VAL	18	C	900	6.19E-01	-3.28E-01	-3.39E-01	-3.17E-01	1.08E-02
VAL	18	CB	277	LYS	16	O	860	5.92E-01	-4.60E-01	-5.27E-01	-3.92E-01	6.70E-02

VAL	18	CB	277	VAL	18	N	898	5.28E-01	-4.63E-01	-5.08E-01	-4.19E-01	4.42E-02
VAL	18	CB	277	VAL	18	O	901	3.87E-01	-1.08E+00	-1.48E+00	-6.85E-01	3.97E-01
VAL	18	CB	277	PHE	19	N	914	5.19E-01	-3.96E-01	-4.24E-01	-3.67E-01	2.83E-02
VAL	18	H	280	LYS	16	O	860	5.44E-01	-6.05E-01	-6.66E-01	-5.44E-01	6.11E-02
VAL	18	H	280	VAL	18	N	898	6.01E-01	-3.81E-01	-3.85E-01	-3.78E-01	3.23E-03
VAL	18	H	280	VAL	18	O	901	4.96E-01	-5.31E-01	-5.59E-01	-5.04E-01	2.76E-02
PHE	19	N	289	VAL	18	C	900	5.08E-01	-4.67E-01	-4.86E-01	-4.49E-01	1.86E-02
PHE	19	N	289	PHE	19	C	916	4.68E-01	-7.01E-01	-7.49E-01	-6.52E-01	4.89E-02
PHE	19	C	291	VAL	18	O	901	6.33E-01	-3.90E-01	-4.25E-01	-3.54E-01	3.54E-02
PHE	19	C	291	PHE	19	O	917	5.06E-01	-9.47E-01	-1.20E+00	-6.91E-01	2.56E-01
PHE	19	C	291	PHE	20	N	934	5.55E-01	-5.51E-01	-7.18E-01	-3.84E-01	1.67E-01
PHE	19	O	292	PHE	19	C	916	5.65E-01	-7.03E-01	-8.97E-01	-5.09E-01	1.94E-01
PHE	19	H	300	VAL	18	O	901	3.79E-01	-6.03E-01	-7.33E-01	-4.72E-01	1.30E-01
PHE	19	H	300	PHE	19	N	914	4.41E-01	-3.44E-01	-3.61E-01	-3.27E-01	1.70E-02
PHE	19	H	300	PHE	19	O	917	3.90E-01	-8.84E-01	-1.21E+00	-5.56E-01	3.28E-01
PHE	20	N	309	PHE	19	C	916	5.23E-01	-6.32E-01	-7.97E-01	-4.68E-01	1.64E-01
PHE	20	N	309	PHE	20	C	936	6.14E-01	-3.77E-01	-4.00E-01	-3.54E-01	2.32E-02
PHE	20	C	311	PHE	19	O	917	5.59E-01	-9.80E-01	-1.35E+00	-6.05E-01	3.75E-01
PHE	20	C	311	PHE	20	N	934	5.75E-01	-4.38E-01	-4.78E-01	-3.98E-01	4.02E-02
PHE	20	C	311	PHE	20	O	937	6.23E-01	-5.02E-01	-5.42E-01	-4.61E-01	4.04E-02
PHE	20	C	311	ALA	21	N	954	5.23E-01	-6.00E-01	-6.79E-01	-5.21E-01	7.93E-02
PHE	20	C	311	ALA	21	O	957	7.02E-01	-4.61E-01	-5.50E-01	-3.71E-01	8.94E-02
PHE	20	C	311	GLU	22	N	964	6.11E-01	-4.45E-01	-5.01E-01	-3.89E-01	5.57E-02
PHE	20	O	312	PHE	19	C	916	5.37E-01	-8.79E-01	-1.19E+00	-5.63E-01	3.16E-01
PHE	20	O	312	PHE	20	C	936	4.91E-01	-8.78E-01	-9.97E-01	-7.58E-01	1.20E-01
PHE	20	O	312	ALA	21	C	956	5.93E-01	-6.41E-01	-7.90E-01	-4.93E-01	1.49E-01
ALA	21	N	329	PHE	20	C	936	6.08E-01	-4.22E-01	-4.56E-01	-3.88E-01	3.37E-02
ALA	21	N	329	ALA	21	C	956	6.13E-01	-4.34E-01	-4.73E-01	-3.94E-01	3.97E-02
ALA	21	C	331	ALA	21	N	954	6.11E-01	-4.37E-01	-4.77E-01	-3.96E-01	4.09E-02
ALA	21	C	331	ALA	21	O	957	6.00E-01	-6.37E-01	-7.31E-01	-5.43E-01	9.43E-02
ALA	21	C	331	GLU	22	N	964	5.33E-01	-6.21E-01	-6.91E-01	-5.51E-01	6.98E-02
ALA	21	C	331	GLU	22	O	967	6.50E-01	-6.59E-01	-8.82E-01	-4.36E-01	2.23E-01
ALA	21	C	331	GLU	22	OE1	971	5.32E-01	-1.56E+00	-2.11E+00	-1.00E+00	5.55E-01
ALA	21	C	331	GLU	22	OE2	972	5.99E-01	-1.15E+00	-1.55E+00	-7.42E-01	4.03E-01
ALA	21	C	331	ASP	23	N	979	6.32E-01	-5.59E-01	-6.10E-01	-5.09E-01	5.06E-02
ALA	21	C	331	ASP	23	O	982	6.56E-01	-4.76E-01	-5.44E-01	-4.08E-01	6.82E-02
ALA	21	C	331	ASP	23	OD1	985	9.51E-01	-3.48E-01	-3.72E-01	-3.25E-01	2.34E-02
ALA	21	O	332	ALA	21	C	956	5.60E-01	-7.53E-01	-8.80E-01	-6.25E-01	1.28E-01
ALA	21	O	332	GLU	22	C	966	6.11E-01	-5.32E-01	-6.51E-01	-4.14E-01	1.19E-01
ALA	21	O	332	GLU	22	CD	970	4.65E-01	-1.72E+00	-2.16E+00	-1.29E+00	4.35E-01
ALA	21	O	332	ASP	23	CG	984	9.04E-01	-3.83E-01	-4.29E-01	-3.36E-01	4.65E-02
GLU	22	N	339	ALA	21	C	956	6.27E-01	-4.31E-01	-4.71E-01	-3.90E-01	4.06E-02
GLU	22	N	339	GLU	22	C	966	5.89E-01	-4.11E-01	-4.59E-01	-3.64E-01	4.75E-02
GLU	22	N	339	GLU	22	CD	970	5.66E-01	-8.71E-01	-1.18E+00	-5.61E-01	3.10E-01
GLU	22	C	341	GLU	22	O	967	5.98E-01	-5.99E-01	-7.46E-01	-4.51E-01	1.48E-01

GLU	22	C	341	GLU	22	OE1	971	6.18E-01	-8.42E-01	-1.14E+00	-5.47E-01	2.96E-01
GLU	22	C	341	GLU	22	OE2	972	6.87E-01	-6.55E-01	-8.73E-01	-4.38E-01	2.18E-01
GLU	22	C	341	ASP	23	N	979	5.58E-01	-6.30E-01	-7.28E-01	-5.32E-01	9.80E-02
GLU	22	C	341	ASP	23	O	982	4.58E-01	-9.44E-01	-1.22E+00	-6.67E-01	2.76E-01
GLU	22	C	341	ASP	23	OD1	985	7.73E-01	-4.12E-01	-4.53E-01	-3.71E-01	4.06E-02
GLU	22	O	342	GLU	22	C	966	5.57E-01	-6.96E-01	-8.40E-01	-5.53E-01	1.43E-01
GLU	22	O	342	GLU	22	CD	970	6.30E-01	-1.04E+00	-1.47E+00	-6.02E-01	4.36E-01
GLU	22	O	342	ASP	23	C	981	5.32E-01	-7.87E-01	-9.90E-01	-5.83E-01	2.04E-01
GLU	22	O	342	ASP	23	CG	984	6.83E-01	-7.55E-01	-9.64E-01	-5.47E-01	2.09E-01
GLU	22	CD	345	ALA	21	O	957	8.71E-01	-4.14E-01	-4.56E-01	-3.72E-01	4.23E-02
GLU	22	CD	345	GLU	22	O	967	8.61E-01	-5.36E-01	-7.75E-01	-2.97E-01	2.39E-01
GLU	22	CD	345	GLU	22	OE1	971	5.80E-01	-1.40E+00	-1.66E+00	-1.14E+00	2.60E-01
GLU	22	CD	345	GLU	22	OE2	972	6.20E-01	-1.24E+00	-1.52E+00	-9.66E-01	2.76E-01
GLU	22	CD	345	ASP	23	N	979	8.31E-01	-4.79E-01	-5.97E-01	-3.62E-01	1.17E-01
GLU	22	CD	345	ASP	23	O	982	8.42E-01	-4.13E-01	-5.01E-01	-3.25E-01	8.80E-02
GLU	22	OE1	346	ALA	21	C	956	9.08E-01	-4.28E-01	-4.68E-01	-3.87E-01	4.03E-02
GLU	22	OE1	346	GLU	22	CD	970	6.16E-01	-1.21E+00	-1.41E+00	-1.02E+00	1.95E-01
GLU	22	OE2	347	GLU	22	CD	970	5.76E-01	-1.45E+00	-1.73E+00	-1.16E+00	2.86E-01
ASP	23	N	354	ALA	21	C	956	7.96E-01	-3.59E-01	-3.89E-01	-3.29E-01	3.00E-02
ASP	23	N	354	GLU	22	C	966	6.05E-01	-5.29E-01	-6.28E-01	-4.31E-01	9.86E-02
ASP	23	N	354	GLU	22	CD	970	6.81E-01	-6.72E-01	-7.83E-01	-5.61E-01	1.11E-01
ASP	23	N	354	ASP	23	C	981	5.29E-01	-6.49E-01	-7.35E-01	-5.64E-01	8.56E-02
ASP	23	N	354	ASP	23	CG	984	6.76E-01	-6.56E-01	-7.62E-01	-5.50E-01	1.06E-01
ASP	23	C	356	ASP	23	N	979	6.90E-01	-3.61E-01	-3.84E-01	-3.38E-01	2.30E-02
ASP	23	C	356	ASP	23	O	982	4.48E-01	-8.53E-01	-9.04E-01	-8.01E-01	5.17E-02
ASP	23	C	356	ASP	23	OD1	985	8.07E-01	-3.52E-01	-3.67E-01	-3.37E-01	1.53E-02
ASP	23	C	356	VAL	24	N	991	6.27E-01	-3.52E-01	-3.70E-01	-3.34E-01	1.78E-02
ASP	23	O	357	ASP	23	C	981	6.77E-01	-3.34E-01	-3.40E-01	-3.27E-01	6.44E-03
ASP	23	O	357	ASP	23	CG	984	8.52E-01	-3.74E-01	-4.03E-01	-3.44E-01	2.94E-02
ASP	23	CG	359	GLU	22	O	967	7.34E-01	-7.34E-01	-1.10E+00	-3.66E-01	3.68E-01
ASP	23	CG	359	GLU	22	OE1	971	8.45E-01	-6.36E-01	-7.11E-01	-5.61E-01	7.53E-02
ASP	23	CG	359	GLU	22	OE2	972	9.11E-01	-5.54E-01	-6.02E-01	-5.07E-01	4.77E-02
ASP	23	CG	359	ASP	23	N	979	6.55E-01	-6.94E-01	-7.91E-01	-5.98E-01	9.63E-02
ASP	23	CG	359	ASP	23	O	982	4.76E-01	-1.56E+00	-2.20E+00	-9.20E-01	6.39E-01
ASP	23	CG	359	ASP	23	OD1	985	6.44E-01	-9.29E-01	-1.04E+00	-8.20E-01	1.09E-01
ASP	23	CG	359	ASP	23	OD2	986	5.72E-01	-1.23E+00	-1.45E+00	-1.02E+00	2.14E-01
ASP	23	CG	359	VAL	24	N	991	6.64E-01	-6.13E-01	-8.13E-01	-4.14E-01	2.00E-01
ASP	23	OD1	360	GLU	22	C	966	6.96E-01	-5.01E-01	-5.56E-01	-4.46E-01	5.51E-02
ASP	23	OD1	360	GLU	22	CD	970	8.46E-01	-5.85E-01	-6.73E-01	-4.98E-01	8.75E-02
ASP	23	OD1	360	ASP	23	C	981	5.26E-01	-9.75E-01	-1.27E+00	-6.81E-01	2.93E-01
ASP	23	OD1	360	ASP	23	CG	984	5.22E-01	-1.48E+00	-1.67E+00	-1.29E+00	1.88E-01
ASP	23	OD1	360	VAL	24	H	998	6.86E-01	-5.36E-01	-6.78E-01	-3.94E-01	1.42E-01
ASP	23	OD2	361	GLU	22	CD	970	8.84E-01	-5.39E-01	-6.09E-01	-4.69E-01	6.99E-02
ASP	23	OD2	361	ASP	23	C	981	6.35E-01	-6.88E-01	-1.00E+00	-3.73E-01	3.15E-01
ASP	23	OD2	361	ASP	23	CG	984	6.01E-01	-1.11E+00	-1.32E+00	-9.00E-01	2.12E-01
ASP	23	H	362	ASP	23	O	982	4.67E-01	-7.38E-01	-1.08E+00	-3.98E-01	3.40E-01

VAL	24	N	366	ASP	23	C	981	5.12E-01	-5.54E-01	-5.97E-01	-5.11E-01	4.27E-02
VAL	24	N	366	ASP	23	CG	984	7.38E-01	-4.37E-01	-4.85E-01	-3.89E-01	4.77E-02
VAL	24	N	366	VAL	24	C	993	5.60E-01	-4.61E-01	-5.11E-01	-4.10E-01	5.07E-02
VAL	24	N	366	VAL	24	CB	995	5.70E-01	-4.02E-01	-4.68E-01	-3.36E-01	6.56E-02
VAL	24	N	366	VAL	24	H	998	6.48E-01	-3.26E-01	-3.32E-01	-3.20E-01	6.32E-03
VAL	24	C	368	ASP	23	O	982	5.47E-01	-5.64E-01	-6.69E-01	-4.60E-01	1.05E-01
VAL	24	CB	370	ASP	23	O	982	5.48E-01	-4.92E-01	-5.71E-01	-4.13E-01	7.89E-02
VAL	24	H	373	ASP	23	N	979	5.92E-01	-5.06E-01	-5.74E-01	-4.37E-01	6.82E-02
VAL	24	H	373	ASP	23	O	982	3.31E-01	-2.10E+00	-2.64E+00	-1.55E+00	5.49E-01
VAL	24	H	373	ASP	23	OD1	985	7.10E-01	-4.47E-01	-4.80E-01	-4.14E-01	3.31E-02
VAL	24	H	373	ASP	23	OD2	986	7.11E-01	-4.76E-01	-5.89E-01	-3.63E-01	1.13E-01
VAL	24	H	373	VAL	24	N	991	4.64E-01	-6.96E-01	-7.31E-01	-6.60E-01	3.51E-02
VAL	24	H	373	GLY	25	N	1007	4.83E-01	-5.42E-01	-6.26E-01	-4.58E-01	8.41E-02
GLY	25	C	384	VAL	24	O	994	6.49E-01	-5.00E-01	-7.97E-01	-2.03E-01	2.97E-01
GLY	25	C	384	GLY	25	N	1007	4.72E-01	-7.41E-01	-8.12E-01	-6.70E-01	7.13E-02
GLY	25	C	384	GLY	25	O	1010	6.47E-01	-4.87E-01	-4.94E-01	-4.80E-01	7.01E-03
GLY	25	C	384	SER	26	N	1014	4.94E-01	-9.46E-01	-9.82E-01	-9.10E-01	3.63E-02
GLY	25	C	384	SER	26	O	1017	5.72E-01	-7.28E-01	-7.68E-01	-6.88E-01	4.00E-02
GLY	25	C	384	SER	26	OG	1019	6.16E-01	-7.10E-01	-8.15E-01	-6.04E-01	1.06E-01
GLY	25	O	385	VAL	24	C	993	4.80E-01	-7.76E-01	-9.30E-01	-6.22E-01	1.54E-01
GLY	25	O	385	GLY	25	C	1009	4.15E-01	-1.37E+00	-1.41E+00	-1.32E+00	4.61E-02
GLY	25	O	385	GLY	25	H	1011	3.34E-01	-1.33E+00	-1.77E+00	-8.85E-01	4.40E-01
GLY	25	O	385	SER	26	C	1016	5.64E-01	-5.50E-01	-5.88E-01	-5.12E-01	3.79E-02
GLY	25	O	385	SER	26	H	1020	3.19E-01	-1.72E+00	-1.98E+00	-1.46E+00	2.59E-01
GLY	25	O	385	SER	26	HG	1024	5.91E-01	-4.65E-01	-5.24E-01	-4.07E-01	5.82E-02
SER	26	N	389	VAL	24	C	993	6.73E-01	-3.79E-01	-4.30E-01	-3.28E-01	5.07E-02
SER	26	N	389	GLY	25	C	1009	5.86E-01	-6.39E-01	-6.58E-01	-6.20E-01	1.92E-02
SER	26	N	389	SER	26	C	1016	6.26E-01	-4.62E-01	-4.73E-01	-4.51E-01	1.13E-02
SER	26	N	389	SER	26	H	1020	4.29E-01	-7.93E-01	-8.12E-01	-7.74E-01	1.90E-02
SER	26	N	389	ASN	27	C	1027	8.78E-01	-3.14E-01	-3.20E-01	-3.09E-01	5.36E-03
SER	26	N	389	LYS	28	C	1041	9.60E-01	-3.21E-01	-3.26E-01	-3.16E-01	5.05E-03
SER	26	C	391	GLY	25	O	1010	7.21E-01	-3.29E-01	-3.44E-01	-3.14E-01	1.53E-02
SER	26	C	391	SER	26	N	1014	5.11E-01	-7.26E-01	-7.59E-01	-6.92E-01	3.36E-02
SER	26	C	391	SER	26	O	1017	3.99E-01	-1.44E+00	-1.46E+00	-1.42E+00	2.03E-02
SER	26	C	391	SER	26	OG	1019	5.73E-01	-7.42E-01	-9.31E-01	-5.52E-01	1.89E-01
SER	26	C	391	ASN	27	N	1025	6.23E-01	-3.70E-01	-3.73E-01	-3.67E-01	2.81E-03
SER	26	C	391	ASN	27	ND2	1032	8.72E-01	-3.73E-01	-4.21E-01	-3.24E-01	4.83E-02
SER	26	C	391	LYS	28	N	1039	6.14E-01	-3.87E-01	-3.98E-01	-3.77E-01	1.02E-02
SER	26	C	391	LYS	28	O	1042	7.02E-01	-3.83E-01	-4.00E-01	-3.67E-01	1.65E-02
SER	26	O	392	GLY	25	C	1009	7.28E-01	-4.44E-01	-4.64E-01	-4.24E-01	2.02E-02
SER	26	O	392	SER	26	C	1016	6.24E-01	-4.98E-01	-5.01E-01	-4.95E-01	2.94E-03
SER	26	O	392	SER	26	H	1020	5.46E-01	-4.77E-01	-4.96E-01	-4.59E-01	1.82E-02
SER	26	O	392	ASN	27	C	1027	7.77E-01	-4.16E-01	-4.21E-01	-4.11E-01	4.95E-03
SER	26	O	392	LYS	28	C	1041	7.73E-01	-4.95E-01	-5.07E-01	-4.82E-01	1.27E-02
SER	26	OG	394	GLY	25	C	1009	6.63E-01	-6.02E-01	-6.74E-01	-5.29E-01	7.27E-02
SER	26	OG	394	SER	26	C	1016	6.47E-01	-5.26E-01	-5.96E-01	-4.56E-01	6.95E-02

SER	26	OG	394	SER	26	H	1020	4.94E-01	-6.98E-01	-8.08E-01	-5.87E-01	1.10E-01
SER	26	OG	394	SER	26	HG	1024	5.25E-01	-7.61E-01	-8.42E-01	-6.80E-01	8.14E-02
SER	26	OG	394	ASN	27	C	1027	8.66E-01	-3.84E-01	-4.09E-01	-3.58E-01	2.54E-02
SER	26	OG	394	LYS	28	C	1041	9.09E-01	-4.16E-01	-4.38E-01	-3.94E-01	2.20E-02
SER	26	H	395	SER	26	N	1014	6.16E-01	-3.40E-01	-3.44E-01	-3.36E-01	3.91E-03
SER	26	H	395	SER	26	O	1017	6.20E-01	-3.61E-01	-3.71E-01	-3.52E-01	9.13E-03
SER	26	HA	396	SER	26	N	1014	3.44E-01	-5.89E-01	-6.26E-01	-5.52E-01	3.72E-02
SER	26	HA	396	SER	26	O	1017	3.55E-01	-5.82E-01	-6.20E-01	-5.43E-01	3.88E-02
SER	26	HG	399	SER	26	N	1014	6.00E-01	-5.01E-01	-6.04E-01	-3.98E-01	1.03E-01
SER	26	HG	399	SER	26	O	1017	6.03E-01	-5.04E-01	-5.54E-01	-4.55E-01	4.94E-02
SER	26	HG	399	SER	26	OG	1019	5.12E-01	-8.20E-01	-9.58E-01	-6.83E-01	1.37E-01
ASN	27	N	400	GLY	25	C	1009	5.66E-01	-5.53E-01	-5.93E-01	-5.14E-01	3.95E-02
ASN	27	N	400	SER	26	C	1016	4.05E-01	-1.02E+00	-1.04E+00	-1.00E+00	1.62E-02
ASN	27	N	400	SER	26	H	1020	3.87E-01	-8.31E-01	-9.00E-01	-7.63E-01	6.85E-02
ASN	27	N	400	ASN	27	C	1027	5.65E-01	-5.85E-01	-5.99E-01	-5.72E-01	1.35E-02
ASN	27	N	400	ASN	27	CG	1030	6.87E-01	-3.67E-01	-3.71E-01	-3.63E-01	4.13E-03
ASN	27	N	400	LYS	28	C	1041	6.22E-01	-5.60E-01	-5.78E-01	-5.41E-01	1.83E-02
ASN	27	N	400	LYS	28	H	1048	3.96E-01	-5.65E-01	-5.95E-01	-5.35E-01	3.01E-02
ASN	27	C	402	GLY	25	O	1010	8.38E-01	-3.20E-01	-3.31E-01	-3.09E-01	1.10E-02
ASN	27	C	402	SER	26	N	1014	6.39E-01	-5.67E-01	-5.90E-01	-5.44E-01	2.31E-02
ASN	27	C	402	SER	26	O	1017	3.85E-01	-2.03E+00	-2.15E+00	-1.90E+00	1.24E-01
ASN	27	C	402	SER	26	OG	1019	6.59E-01	-6.90E-01	-8.48E-01	-5.33E-01	1.57E-01
ASN	27	C	402	ASN	27	N	1025	5.53E-01	-6.14E-01	-6.28E-01	-6.00E-01	1.40E-02
ASN	27	C	402	ASN	27	O	1028	6.07E-01	-6.09E-01	-6.13E-01	-6.04E-01	4.34E-03
ASN	27	C	402	ASN	27	OD1	1031	7.38E-01	-4.28E-01	-4.74E-01	-3.82E-01	4.61E-02
ASN	27	C	402	ASN	27	ND2	1032	8.05E-01	-5.40E-01	-5.99E-01	-4.80E-01	5.90E-02
ASN	27	C	402	LYS	28	N	1039	3.89E-01	-1.47E+00	-1.50E+00	-1.44E+00	2.78E-02
ASN	27	C	402	LYS	28	O	1042	3.86E-01	-1.98E+00	-2.22E+00	-1.74E+00	2.38E-01
ASN	27	C	402	GLY	29	N	1061	5.14E-01	-6.35E-01	-6.80E-01	-5.91E-01	4.47E-02
ASN	27	C	402	GLY	29	O	1064	6.04E-01	-6.06E-01	-6.55E-01	-5.57E-01	4.94E-02
ASN	27	C	402	ALA	30	N	1068	6.19E-01	-4.54E-01	-4.75E-01	-4.33E-01	2.11E-02
ASN	27	C	402	ILE	41	O	1224	9.21E-01	-3.34E-01	-3.41E-01	-3.27E-01	7.48E-03
ASN	27	O	403	GLY	25	C	1009	7.56E-01	-3.72E-01	-3.85E-01	-3.59E-01	1.29E-02
ASN	27	O	403	SER	26	C	1016	4.55E-01	-9.32E-01	-9.74E-01	-8.89E-01	4.25E-02
ASN	27	O	403	ASN	27	C	1027	3.90E-01	-1.76E+00	-1.78E+00	-1.74E+00	2.26E-02
ASN	27	O	403	ASN	27	CG	1030	6.61E-01	-4.84E-01	-4.95E-01	-4.73E-01	1.10E-02
ASN	27	O	403	LYS	28	C	1041	3.11E-01	-3.87E+00	-4.17E+00	-3.57E+00	3.03E-01
ASN	27	O	403	LYS	28	H	1048	2.00E-01	-4.83E+00	-4.89E+00	-4.76E+00	6.25E-02
ASN	27	O	403	GLY	29	C	1063	5.02E-01	-8.87E-01	-9.55E-01	-8.18E-01	6.86E-02
ASN	27	O	403	GLY	29	H	1065	4.49E-01	-5.13E-01	-5.67E-01	-4.59E-01	5.41E-02
ASN	27	O	403	ALA	30	C	1070	7.55E-01	-3.66E-01	-3.78E-01	-3.54E-01	1.20E-02
ASN	27	O	403	ALA	30	H	1073	5.31E-01	-3.94E-01	-4.22E-01	-3.66E-01	2.82E-02
ASN	27	CG	405	GLY	25	N	1007	5.19E-01	-5.89E-01	-6.38E-01	-5.41E-01	4.83E-02
ASN	27	CG	405	GLY	25	O	1010	6.13E-01	-5.60E-01	-6.20E-01	-5.01E-01	5.94E-02
ASN	27	CG	405	SER	26	N	1014	4.64E-01	-1.11E+00	-1.17E+00	-1.04E+00	6.39E-02
ASN	27	CG	405	SER	26	O	1017	2.94E-01	-4.05E+00	-4.30E+00	-3.80E+00	2.55E-01

ASN	27	CG	405	SER	26	OG	1019	6.44E-01	-6.51E-01	-7.52E-01	-5.51E-01	1.00E-01
ASN	27	CG	405	ASN	27	N	1025	4.43E-01	-9.83E-01	-1.02E+00	-9.43E-01	4.07E-02
ASN	27	CG	405	ASN	27	O	1028	6.40E-01	-5.17E-01	-5.31E-01	-5.03E-01	1.42E-02
ASN	27	CG	405	ASN	27	OD1	1031	4.88E-01	-1.08E+00	-1.31E+00	-8.48E-01	2.33E-01
ASN	27	CG	405	ASN	27	ND2	1032	5.72E-01	-1.15E+00	-1.53E+00	-7.77E-01	3.78E-01
ASN	27	CG	405	LYS	28	N	1039	5.05E-01	-7.29E-01	-7.67E-01	-6.92E-01	3.75E-02
ASN	27	CG	405	LYS	28	O	1042	6.79E-01	-4.96E-01	-5.30E-01	-4.62E-01	3.40E-02
ASN	27	CG	405	GLY	29	O	1064	7.48E-01	-3.73E-01	-3.96E-01	-3.50E-01	2.31E-02
ASN	27	CG	405	ALA	30	N	1068	6.88E-01	-3.47E-01	-3.64E-01	-3.31E-01	1.64E-02
ASN	27	OD1	406	VAL	24	C	993	5.44E-01	-5.96E-01	-6.90E-01	-5.03E-01	9.39E-02
ASN	27	OD1	406	VAL	24	CB	995	4.69E-01	-8.15E-01	-1.02E+00	-6.10E-01	2.05E-01
ASN	27	OD1	406	GLY	25	C	1009	5.25E-01	-8.42E-01	-9.83E-01	-7.00E-01	1.41E-01
ASN	27	OD1	406	SER	26	C	1016	4.23E-01	-1.38E+00	-1.90E+00	-8.63E-01	5.16E-01
ASN	27	OD1	406	SER	26	H	1020	4.24E-01	-8.26E-01	-9.42E-01	-7.10E-01	1.16E-01
ASN	27	OD1	406	ASN	27	C	1027	5.90E-01	-7.31E-01	-9.27E-01	-5.36E-01	1.96E-01
ASN	27	OD1	406	ASN	27	CG	1030	5.46E-01	-8.85E-01	-1.20E+00	-5.72E-01	3.13E-01
ASN	27	OD1	406	LYS	28	C	1041	7.36E-01	-5.00E-01	-5.51E-01	-4.49E-01	5.12E-02
ASN	27	OD1	406	GLY	29	C	1063	7.99E-01	-3.43E-01	-3.70E-01	-3.15E-01	2.74E-02
ASN	27	ND2	407	PHE	19	C	916	7.97E-01	-4.83E-01	-5.20E-01	-4.45E-01	3.74E-02
ASN	27	ND2	407	PHE	20	C	936	8.83E-01	-4.02E-01	-4.19E-01	-3.84E-01	1.77E-02
ASN	27	ND2	407	ASP	23	C	981	7.42E-01	-4.41E-01	-4.69E-01	-4.13E-01	2.82E-02
ASN	27	ND2	407	VAL	24	C	993	5.24E-01	-9.34E-01	-1.05E+00	-8.22E-01	1.12E-01
ASN	27	ND2	407	VAL	24	CB	995	4.31E-01	-1.41E+00	-1.73E+00	-1.09E+00	3.19E-01
ASN	27	ND2	407	VAL	24	H	998	6.82E-01	-5.28E-01	-5.95E-01	-4.61E-01	6.69E-02
ASN	27	ND2	407	GLY	25	C	1009	5.01E-01	-1.36E+00	-1.52E+00	-1.19E+00	1.66E-01
ASN	27	ND2	407	GLY	25	H	1011	4.31E-01	-8.61E-01	-9.67E-01	-7.55E-01	1.06E-01
ASN	27	ND2	407	SER	26	C	1016	3.77E-01	-2.56E+00	-3.15E+00	-1.97E+00	5.91E-01
ASN	27	ND2	407	SER	26	H	1020	4.18E-01	-1.31E+00	-1.57E+00	-1.05E+00	2.60E-01
ASN	27	ND2	407	SER	26	HG	1024	6.92E-01	-5.57E-01	-6.91E-01	-4.22E-01	1.34E-01
ASN	27	ND2	407	ASN	27	C	1027	5.32E-01	-1.30E+00	-1.50E+00	-1.09E+00	2.04E-01
ASN	27	ND2	407	ASN	27	CG	1030	4.60E-01	-1.96E+00	-2.45E+00	-1.48E+00	4.84E-01
ASN	27	ND2	407	ASN	27	H	1033	5.03E-01	-6.34E-01	-7.60E-01	-5.09E-01	1.26E-01
ASN	27	ND2	407	ASN	27	HD21	1037	5.37E-01	-7.62E-01	-9.59E-01	-5.64E-01	1.98E-01
ASN	27	ND2	407	ASN	27	HD22	1038	5.42E-01	-6.71E-01	-6.82E-01	-6.60E-01	1.07E-02
ASN	27	ND2	407	LYS	28	C	1041	7.05E-01	-7.93E-01	-8.37E-01	-7.50E-01	4.36E-02
ASN	27	ND2	407	LYS	28	H	1048	4.63E-01	-7.10E-01	-7.92E-01	-6.27E-01	8.23E-02
ASN	27	ND2	407	GLY	29	C	1063	7.60E-01	-5.56E-01	-5.96E-01	-5.15E-01	4.05E-02
ASN	27	ND2	407	ALA	30	C	1070	8.42E-01	-4.56E-01	-4.91E-01	-4.22E-01	3.43E-02
ASN	27	H	408	SER	26	N	1014	3.66E-01	-9.01E-01	-9.81E-01	-8.21E-01	7.98E-02
ASN	27	H	408	SER	26	O	1017	2.00E-01	-5.42E+00	-5.57E+00	-5.27E+00	1.50E-01
ASN	27	H	408	SER	26	OG	1019	4.48E-01	-7.75E-01	-1.06E+00	-4.89E-01	2.85E-01
ASN	27	H	408	ASN	27	N	1025	4.24E-01	-4.81E-01	-4.86E-01	-4.75E-01	5.91E-03
ASN	27	H	408	LYS	28	N	1039	4.51E-01	-4.17E-01	-4.38E-01	-3.97E-01	2.06E-02
ASN	27	HB2	410	SER	26	O	1017	2.60E-01	-4.27E-01	-4.60E-01	-3.94E-01	3.30E-02
ASN	27	HD21	412	SER	26	N	1014	4.78E-01	-6.88E-01	-8.44E-01	-5.32E-01	1.56E-01
ASN	27	HD21	412	SER	26	O	1017	3.14E-01	-2.85E+00	-3.94E+00	-1.77E+00	1.09E+00

ASN	27	HD21	412	ASN	27	ND2	1032	4.94E-01	-9.05E-01	-1.07E+00	-7.43E-01	1.62E-01
ASN	27	HD22	413	GLY	25	N	1007	3.99E-01	-6.84E-01	-7.22E-01	-6.46E-01	3.78E-02
ASN	27	HD22	413	GLY	25	O	1010	5.25E-01	-5.03E-01	-6.05E-01	-4.00E-01	1.02E-01
ASN	27	HD22	413	SER	26	N	1014	4.28E-01	-8.57E-01	-9.91E-01	-7.22E-01	1.34E-01
ASN	27	HD22	413	SER	26	O	1017	3.60E-01	-1.47E+00	-1.71E+00	-1.23E+00	2.41E-01
ASN	27	HD22	413	ASN	27	N	1025	4.58E-01	-6.05E-01	-7.24E-01	-4.86E-01	1.19E-01
ASN	27	HD22	413	ASN	27	ND2	1032	5.08E-01	-7.80E-01	-7.90E-01	-7.69E-01	1.07E-02
LYS	28	N	414	SER	26	C	1016	6.14E-01	-3.88E-01	-4.00E-01	-3.76E-01	1.17E-02
LYS	28	N	414	ASN	27	C	1027	6.12E-01	-4.98E-01	-5.03E-01	-4.94E-01	4.24E-03
LYS	28	N	414	LYS	28	C	1041	4.83E-01	-1.00E+00	-1.04E+00	-9.63E-01	3.78E-02
LYS	28	N	414	LYS	28	H	1048	4.15E-01	-5.05E-01	-5.11E-01	-5.00E-01	5.59E-03
LYS	28	N	414	GLY	29	C	1063	6.44E-01	-4.25E-01	-4.49E-01	-4.01E-01	2.43E-02
LYS	28	C	416	SER	26	N	1014	9.68E-01	-3.17E-01	-3.23E-01	-3.10E-01	6.63E-03
LYS	28	C	416	SER	26	O	1017	7.15E-01	-5.71E-01	-5.87E-01	-5.55E-01	1.60E-02
LYS	28	C	416	ASN	27	O	1028	8.09E-01	-4.11E-01	-4.18E-01	-4.04E-01	6.81E-03
LYS	28	C	416	LYS	28	N	1039	6.03E-01	-6.03E-01	-6.16E-01	-5.91E-01	1.26E-02
LYS	28	C	416	LYS	28	O	1042	3.75E-01	-2.47E+00	-2.55E+00	-2.38E+00	8.45E-02
LYS	28	C	416	GLY	29	N	1061	5.64E-01	-6.00E-01	-6.18E-01	-5.82E-01	1.82E-02
LYS	28	C	416	GLY	29	O	1064	5.71E-01	-8.04E-01	-8.66E-01	-7.41E-01	6.23E-02
LYS	28	C	416	ALA	30	N	1068	6.89E-01	-4.27E-01	-4.40E-01	-4.15E-01	1.27E-02
LYS	28	C	416	ALA	30	O	1071	9.78E-01	-3.20E-01	-3.26E-01	-3.14E-01	5.81E-03
LYS	28	C	416	ILE	41	O	1224	7.39E-01	-5.75E-01	-6.07E-01	-5.44E-01	3.14E-02
LYS	28	C	416	ALA	42	O	1243	8.52E-01	-4.03E-01	-4.33E-01	-3.74E-01	2.94E-02
LYS	28	O	417	ASN	27	C	1027	8.47E-01	-3.47E-01	-3.51E-01	-3.43E-01	4.18E-03
LYS	28	O	417	LYS	28	C	1041	5.95E-01	-8.02E-01	-8.19E-01	-7.85E-01	1.72E-02
LYS	28	O	417	GLY	29	C	1063	6.93E-01	-4.72E-01	-4.87E-01	-4.56E-01	1.56E-02
LYS	28	H	423	SER	26	O	1017	5.45E-01	-3.49E-01	-3.62E-01	-3.36E-01	1.30E-02
LYS	28	H	423	LYS	28	O	1042	5.09E-01	-3.99E-01	-4.28E-01	-3.70E-01	2.91E-02
LYS	28	HA	424	LYS	28	O	1042	2.59E-01	-1.26E+00	-1.38E+00	-1.13E+00	1.27E-01
GLY	29	N	436	ASN	27	C	1027	6.85E-01	-3.40E-01	-3.53E-01	-3.28E-01	1.26E-02
GLY	29	N	436	LYS	28	C	1041	4.27E-01	-1.17E+00	-1.22E+00	-1.12E+00	5.00E-02
GLY	29	N	436	GLY	29	C	1063	4.75E-01	-7.19E-01	-7.57E-01	-6.82E-01	3.76E-02
GLY	29	C	438	SER	26	O	1017	8.60E-01	-3.30E-01	-3.40E-01	-3.19E-01	1.07E-02
GLY	29	C	438	LYS	28	O	1042	5.15E-01	-9.05E-01	-9.86E-01	-8.24E-01	8.08E-02
GLY	29	C	438	GLY	29	N	1061	5.97E-01	-4.24E-01	-4.36E-01	-4.12E-01	1.20E-02
GLY	29	C	438	GLY	29	O	1064	3.91E-01	-1.61E+00	-1.69E+00	-1.52E+00	8.60E-02
GLY	29	C	438	ALA	30	N	1068	5.80E-01	-4.90E-01	-4.98E-01	-4.82E-01	8.47E-03
GLY	29	C	438	ALA	30	O	1071	8.06E-01	-3.51E-01	-3.56E-01	-3.46E-01	5.07E-03
GLY	29	C	438	ILE	31	N	1078	6.61E-01	-4.14E-01	-4.22E-01	-4.05E-01	8.55E-03
GLY	29	C	438	ILE	31	O	1081	7.41E-01	-4.56E-01	-4.67E-01	-4.46E-01	1.06E-02
GLY	29	C	438	ILE	41	O	1224	8.52E-01	-3.58E-01	-3.69E-01	-3.46E-01	1.17E-02
GLY	29	O	439	LYS	28	C	1041	7.12E-01	-5.06E-01	-5.23E-01	-4.88E-01	1.74E-02
GLY	29	O	439	GLY	29	C	1063	5.79E-01	-6.18E-01	-6.31E-01	-6.05E-01	1.31E-02
GLY	29	O	439	ALA	30	C	1070	7.62E-01	-3.49E-01	-3.58E-01	-3.40E-01	9.01E-03
GLY	29	H	440	LYS	28	O	1042	2.63E-01	-2.68E+00	-3.49E+00	-1.88E+00	8.06E-01
GLY	29	H	440	GLY	29	N	1061	3.98E-01	-4.90E-01	-5.03E-01	-4.76E-01	1.36E-02

GLY	29	H	440	GLY	29	O	1064	3.89E-01	-7.35E-01	-8.45E-01	-6.24E-01	1.10E-01
ALA	30	N	443	LYS	28	C	1041	5.40E-01	-7.21E-01	-7.64E-01	-6.79E-01	4.23E-02
ALA	30	N	443	GLY	29	C	1063	4.00E-01	-1.20E+00	-1.24E+00	-1.15E+00	4.86E-02
ALA	30	N	443	ALA	30	C	1070	5.93E-01	-4.57E-01	-4.62E-01	-4.51E-01	5.71E-03
ALA	30	N	443	ILE	31	H	1086	4.91E-01	-4.07E-01	-4.12E-01	-4.02E-01	4.90E-03
ALA	30	C	445	PHE	19	CE2	923	4.30E-01	-3.81E-01	-4.03E-01	-3.59E-01	2.16E-02
ALA	30	C	445	PHE	19	CZ	924	3.58E-01	-3.90E-01	-4.07E-01	-3.73E-01	1.68E-02
ALA	30	C	445	LYS	28	O	1042	6.70E-01	-5.00E-01	-5.41E-01	-4.60E-01	4.05E-02
ALA	30	C	445	GLY	29	N	1061	6.48E-01	-3.51E-01	-3.60E-01	-3.41E-01	9.87E-03
ALA	30	C	445	GLY	29	O	1064	3.29E-01	-2.50E+00	-2.62E+00	-2.38E+00	1.23E-01
ALA	30	C	445	ALA	30	N	1068	4.84E-01	-7.27E-01	-7.42E-01	-7.12E-01	1.47E-02
ALA	30	C	445	ALA	30	O	1071	6.06E-01	-5.99E-01	-6.02E-01	-5.95E-01	3.44E-03
ALA	30	C	445	ALA	30	CB	1072	5.41E-01	-3.18E-01	-3.20E-01	-3.15E-01	2.81E-03
ALA	30	C	445	ILE	31	N	1078	4.17E-01	-1.17E+00	-1.18E+00	-1.16E+00	1.02E-02
ALA	30	C	445	ILE	31	O	1081	4.41E-01	-1.40E+00	-1.43E+00	-1.38E+00	2.34E-02
ALA	30	C	445	ILE	32	N	1097	6.47E-01	-4.23E-01	-4.26E-01	-4.20E-01	3.19E-03
ALA	30	O	446	PHE	19	HE2	932	3.66E-01	-4.53E-01	-5.01E-01	-4.04E-01	4.87E-02
ALA	30	O	446	PHE	19	HZ	933	2.50E-01	-1.21E+00	-1.21E+00	-1.21E+00	1.45E-16
ALA	30	O	446	ASN	27	C	1027	7.83E-01	-3.94E-01	-4.10E-01	-3.78E-01	1.62E-02
ALA	30	O	446	LYS	28	C	1041	6.49E-01	-6.61E-01	-6.90E-01	-6.32E-01	2.90E-02
ALA	30	O	446	GLY	29	C	1063	3.53E-01	-2.30E+00	-2.37E+00	-2.22E+00	7.43E-02
ALA	30	O	446	ALA	30	C	1070	3.61E-01	-2.11E+00	-2.13E+00	-2.08E+00	2.32E-02
ALA	30	O	446	ALA	30	H	1073	4.82E-01	-5.21E-01	-5.32E-01	-5.09E-01	1.14E-02
ALA	30	O	446	ALA	30	HA	1074	2.42E-01	-1.40E+00	-1.40E+00	-1.40E+00	1.45E-16
ALA	30	O	446	ILE	31	C	1080	4.33E-01	-1.31E+00	-1.33E+00	-1.29E+00	1.71E-02
ALA	30	O	446	ILE	31	H	1086	2.04E-01	-6.35E+00	-6.45E+00	-6.25E+00	9.96E-02
ALA	30	O	446	ILE	32	C	1099	7.82E-01	-3.63E-01	-3.66E-01	-3.60E-01	2.92E-03
ALA	30	O	446	ILE	32	H	1105	6.18E-01	-3.31E-01	-3.33E-01	-3.28E-01	2.63E-03
ALA	30	CB	447	PHE	19	HE2	932	2.78E-01	-3.99E-01	-4.26E-01	-3.72E-01	2.70E-02
ALA	30	CB	447	GLY	29	C	1063	4.69E-01	-4.54E-01	-4.68E-01	-4.40E-01	1.39E-02
ALA	30	CB	447	ALA	30	C	1070	5.49E-01	-3.07E-01	-3.09E-01	-3.05E-01	1.83E-03
ALA	30	H	448	LYS	28	O	1042	4.00E-01	-8.96E-01	-1.07E+00	-7.21E-01	1.75E-01
ALA	30	H	448	GLY	29	N	1061	4.46E-01	-4.24E-01	-4.38E-01	-4.09E-01	1.47E-02
ALA	30	H	448	GLY	29	O	1064	2.76E-01	-2.52E+00	-3.40E+00	-1.64E+00	8.79E-01
ALA	30	H	448	ALA	30	N	1068	4.14E-01	-5.55E-01	-5.76E-01	-5.33E-01	2.12E-02
ILE	31	N	453	PHE	19	HZ	933	3.15E-01	-5.12E-01	-5.54E-01	-4.70E-01	4.15E-02
ILE	31	N	453	LYS	28	C	1041	8.15E-01	-3.50E-01	-3.62E-01	-3.38E-01	1.19E-02
ILE	31	N	453	GLY	29	C	1063	5.50E-01	-6.14E-01	-6.31E-01	-5.97E-01	1.70E-02
ILE	31	N	453	ALA	30	C	1070	5.73E-01	-5.48E-01	-5.52E-01	-5.44E-01	4.08E-03
ILE	31	N	453	ILE	31	C	1080	5.30E-01	-6.53E-01	-6.61E-01	-6.45E-01	8.18E-03
ILE	31	N	453	ILE	31	H	1086	3.87E-01	-8.23E-01	-8.32E-01	-8.14E-01	8.74E-03
ILE	31	C	455	GLY	29	O	1064	6.31E-01	-5.04E-01	-5.20E-01	-4.87E-01	1.64E-02
ILE	31	C	455	ALA	30	O	1071	7.79E-01	-3.65E-01	-3.66E-01	-3.64E-01	1.38E-03
ILE	31	C	455	ILE	31	N	1078	5.55E-01	-5.88E-01	-5.91E-01	-5.84E-01	3.45E-03
ILE	31	C	455	ILE	31	O	1081	3.63E-01	-2.33E+00	-2.37E+00	-2.29E+00	4.13E-02
ILE	31	C	455	ILE	32	N	1097	5.75E-01	-5.43E-01	-5.50E-01	-5.36E-01	7.09E-03

ILE	31	C	455	ILE	32	O	1100	7.93E-01	-3.95E-01	-3.98E-01	-3.92E-01	3.08E-03
ILE	31	O	456	GLY	29	C	1063	8.23E-01	-3.78E-01	-3.84E-01	-3.72E-01	6.20E-03
ILE	31	O	456	ALA	30	C	1070	7.83E-01	-4.05E-01	-4.07E-01	-4.03E-01	1.98E-03
ILE	31	O	456	ILE	31	C	1080	6.07E-01	-6.65E-01	-6.71E-01	-6.60E-01	5.57E-03
ILE	31	O	456	ILE	31	H	1086	5.84E-01	-4.17E-01	-4.21E-01	-4.14E-01	3.40E-03
ILE	31	O	456	ILE	32	C	1099	7.96E-01	-3.92E-01	-3.99E-01	-3.85E-01	7.18E-03
ILE	31	H	461	GLY	29	O	1064	5.07E-01	-4.73E-01	-4.89E-01	-4.58E-01	1.56E-02
ILE	31	H	461	ILE	31	N	1078	5.80E-01	-3.08E-01	-3.10E-01	-3.06E-01	1.92E-03
ILE	31	H	461	ILE	31	O	1081	5.15E-01	-5.54E-01	-5.65E-01	-5.44E-01	1.03E-02
ILE	31	HA	462	ILE	31	N	1078	3.51E-01	-5.67E-01	-5.77E-01	-5.57E-01	1.01E-02
ILE	31	HA	462	ILE	31	O	1081	2.44E-01	-2.18E+00	-2.18E+00	-2.18E+00	0.00E+00
ILE	32	N	472	PHE	19	HE1	931	3.47E-01	-4.20E-01	-4.52E-01	-3.88E-01	3.20E-02
ILE	32	N	472	ALA	30	C	1070	6.67E-01	-3.99E-01	-4.01E-01	-3.96E-01	2.51E-03
ILE	32	N	472	ILE	31	C	1080	4.18E-01	-1.17E+00	-1.18E+00	-1.15E+00	1.10E-02
ILE	32	N	472	ILE	31	H	1086	4.79E-01	-4.79E-01	-4.83E-01	-4.76E-01	3.50E-03
ILE	32	N	472	ILE	32	C	1099	5.72E-01	-5.51E-01	-5.68E-01	-5.34E-01	1.72E-02
ILE	32	N	472	ILE	32	HA	1106	3.61E-01	-5.27E-01	-5.54E-01	-4.99E-01	2.74E-02
ILE	32	C	474	ILE	31	N	1078	7.06E-01	-3.56E-01	-3.60E-01	-3.51E-01	4.63E-03
ILE	32	C	474	ILE	31	O	1081	4.23E-01	-1.55E+00	-1.60E+00	-1.50E+00	4.72E-02
ILE	32	C	474	ILE	32	N	1097	5.16E-01	-6.95E-01	-7.05E-01	-6.84E-01	1.05E-02
ILE	32	C	474	ILE	32	O	1100	5.73E-01	-7.57E-01	-7.92E-01	-7.22E-01	3.48E-02
ILE	32	C	474	GLY	33	N	1116	4.87E-01	-6.79E-01	-7.51E-01	-6.07E-01	7.18E-02
ILE	32	C	474	GLY	33	O	1119	5.39E-01	-7.45E-01	-8.51E-01	-6.40E-01	1.06E-01
ILE	32	O	475	LYS	16	C	859	8.95E-01	-4.14E-01	-4.38E-01	-3.90E-01	2.42E-02
ILE	32	O	475	LEU	17	C	881	8.06E-01	-3.89E-01	-4.09E-01	-3.70E-01	1.96E-02
ILE	32	O	475	VAL	18	H	905	7.23E-01	-3.63E-01	-3.81E-01	-3.45E-01	1.77E-02
ILE	32	O	475	ALA	30	C	1070	8.39E-01	-3.60E-01	-3.71E-01	-3.48E-01	1.13E-02
ILE	32	O	475	ILE	31	C	1080	5.09E-01	-1.00E+00	-1.08E+00	-9.23E-01	7.90E-02
ILE	32	O	475	ILE	32	C	1099	4.19E-01	-1.64E+00	-1.84E+00	-1.44E+00	1.97E-01
ILE	32	O	475	ILE	32	H	1105	5.72E-01	-4.42E-01	-4.74E-01	-4.09E-01	3.25E-02
ILE	32	O	475	ILE	32	HA	1106	2.83E-01	-1.53E+00	-1.79E+00	-1.27E+00	2.61E-01
ILE	32	O	475	GLY	33	C	1118	5.36E-01	-9.38E-01	-1.06E+00	-8.11E-01	1.27E-01
ILE	32	O	475	LEU	34	C	1125	7.79E-01	-4.13E-01	-4.35E-01	-3.91E-01	2.21E-02
ILE	32	H	480	ILE	31	N	1078	4.65E-01	-5.14E-01	-5.19E-01	-5.09E-01	4.75E-03
ILE	32	H	480	ILE	31	O	1081	2.04E-01	-7.02E+00	-7.14E+00	-6.89E+00	1.25E-01
ILE	32	H	480	ILE	32	N	1097	3.89E-01	-8.13E-01	-8.29E-01	-7.97E-01	1.62E-02
ILE	32	H	480	ILE	32	O	1100	5.94E-01	-4.02E-01	-4.07E-01	-3.97E-01	5.14E-03
ILE	32	HA	481	ILE	31	O	1081	4.95E-01	-3.22E-01	-3.24E-01	-3.20E-01	2.03E-03
GLY	33	N	491	ILE	31	C	1080	5.55E-01	-5.06E-01	-5.84E-01	-4.28E-01	7.81E-02
GLY	33	N	491	ILE	32	C	1099	5.19E-01	-5.79E-01	-6.41E-01	-5.17E-01	6.19E-02
GLY	33	N	491	ILE	32	HA	1106	3.74E-01	-4.39E-01	-5.67E-01	-3.10E-01	1.28E-01
GLY	33	N	491	GLY	33	C	1118	5.33E-01	-5.50E-01	-5.85E-01	-5.15E-01	3.48E-02
GLY	33	C	493	ILE	31	O	1081	6.92E-01	-5.28E-01	-5.81E-01	-4.75E-01	5.30E-02
GLY	33	C	493	ILE	32	N	1097	7.32E-01	-3.47E-01	-3.87E-01	-3.07E-01	4.00E-02
GLY	33	C	493	ILE	32	O	1100	6.98E-01	-5.39E-01	-6.39E-01	-4.40E-01	9.96E-02
GLY	33	C	493	GLY	33	N	1116	5.56E-01	-4.97E-01	-5.18E-01	-4.75E-01	2.15E-02

GLY	33	C	493	GLY	33	O	1119	4.20E-01	-1.36E+00	-1.50E+00	-1.22E+00	1.42E-01
GLY	33	C	493	LEU	34	N	1123	5.15E-01	-5.62E-01	-5.88E-01	-5.37E-01	2.56E-02
GLY	33	C	493	LEU	34	O	1126	6.81E-01	-4.84E-01	-4.99E-01	-4.68E-01	1.56E-02
GLY	33	C	493	MET	35	N	1142	5.50E-01	-5.43E-01	-5.86E-01	-5.00E-01	4.29E-02
GLY	33	C	493	VAL	36	N	1159	6.78E-01	-3.97E-01	-4.26E-01	-3.69E-01	2.86E-02
GLY	33	O	494	ILE	32	C	1099	7.03E-01	-4.18E-01	-4.74E-01	-3.61E-01	5.67E-02
GLY	33	O	494	GLY	33	C	1118	5.71E-01	-6.39E-01	-6.67E-01	-6.11E-01	2.78E-02
GLY	33	O	494	LEU	34	C	1125	6.42E-01	-4.93E-01	-5.23E-01	-4.63E-01	2.99E-02
GLY	33	H	495	ILE	31	O	1081	4.94E-01	-5.34E-01	-7.03E-01	-3.66E-01	1.68E-01
LEU	34	N	498	GLY	33	C	1118	4.93E-01	-6.28E-01	-6.66E-01	-5.89E-01	3.87E-02
LEU	34	N	498	LEU	34	C	1125	5.17E-01	-5.55E-01	-6.00E-01	-5.11E-01	4.44E-02
LEU	34	C	500	GLY	33	O	1119	5.48E-01	-7.01E-01	-7.66E-01	-6.35E-01	6.55E-02
LEU	34	C	500	LEU	34	N	1123	5.71E-01	-4.38E-01	-4.56E-01	-4.21E-01	1.77E-02
LEU	34	C	500	LEU	34	O	1126	5.93E-01	-6.44E-01	-7.16E-01	-5.73E-01	7.19E-02
LEU	34	C	500	MET	35	N	1142	4.22E-01	-1.06E+00	-1.20E+00	-9.12E-01	1.46E-01
LEU	34	C	500	MET	35	O	1145	7.25E-01	-4.34E-01	-4.67E-01	-4.02E-01	3.22E-02
LEU	34	C	500	VAL	36	N	1159	6.09E-01	-4.91E-01	-5.30E-01	-4.52E-01	3.92E-02
LEU	34	O	501	GLY	33	C	1118	5.44E-01	-8.11E-01	-9.19E-01	-7.02E-01	1.09E-01
LEU	34	O	501	LEU	34	C	1125	3.86E-01	-1.87E+00	-2.10E+00	-1.64E+00	2.31E-01
LEU	34	O	501	LEU	34	HA	1132	3.02E-01	-9.62E-01	-1.21E+00	-7.09E-01	2.53E-01
LEU	34	O	501	MET	35	C	1144	5.23E-01	-9.51E-01	-1.09E+00	-8.09E-01	1.42E-01
LEU	34	O	501	VAL	36	H	1166	4.65E-01	-9.40E-01	-1.10E+00	-7.78E-01	1.62E-01
MET	35	N	517	LEU	34	C	1125	5.71E-01	-5.03E-01	-5.85E-01	-4.20E-01	8.27E-02
MET	35	N	517	MET	35	C	1144	6.46E-01	-3.93E-01	-4.05E-01	-3.81E-01	1.17E-02
MET	35	N	517	MET	35	H	1150	4.07E-01	-5.53E-01	-6.08E-01	-4.99E-01	5.47E-02
MET	35	N	517	VAL	36	H	1166	5.62E-01	-3.89E-01	-4.08E-01	-3.69E-01	1.99E-02
MET	35	C	519	GLY	33	O	1119	6.26E-01	-6.03E-01	-7.43E-01	-4.63E-01	1.40E-01
MET	35	C	519	LEU	34	O	1126	6.22E-01	-6.23E-01	-7.28E-01	-5.17E-01	1.05E-01
MET	35	C	519	MET	35	N	1142	4.19E-01	-1.07E+00	-1.13E+00	-1.02E+00	5.40E-02
MET	35	C	519	MET	35	O	1145	5.86E-01	-6.92E-01	-7.29E-01	-6.56E-01	3.63E-02
MET	35	C	519	VAL	36	N	1159	4.71E-01	-9.18E-01	-9.95E-01	-8.41E-01	7.69E-02
MET	35	C	519	VAL	36	O	1162	5.02E-01	-7.29E-01	-8.29E-01	-6.29E-01	9.99E-02
MET	35	O	520	GLY	33	C	1118	6.87E-01	-5.20E-01	-6.21E-01	-4.20E-01	1.00E-01
MET	35	O	520	LEU	34	C	1125	4.94E-01	-1.03E+00	-1.20E+00	-8.51E-01	1.74E-01
MET	35	O	520	MET	35	C	1144	3.97E-01	-1.78E+00	-1.92E+00	-1.64E+00	1.39E-01
MET	35	O	520	MET	35	H	1150	3.40E-01	-1.51E+00	-2.08E+00	-9.46E-01	5.69E-01
MET	35	O	520	MET	35	HA	1151	2.52E-01	-1.31E+00	-1.42E+00	-1.20E+00	1.12E-01
MET	35	O	520	VAL	36	C	1161	5.07E-01	-7.34E-01	-8.27E-01	-6.41E-01	9.32E-02
MET	35	O	520	VAL	36	CB	1163	5.95E-01	-4.63E-01	-5.40E-01	-3.85E-01	7.75E-02
MET	35	O	520	VAL	36	H	1166	3.58E-01	-2.28E+00	-3.34E+00	-1.22E+00	1.06E+00
MET	35	O	520	GLY	37	C	1177	7.47E-01	-4.12E-01	-4.30E-01	-3.94E-01	1.79E-02
VAL	36	N	534	GLY	33	C	1118	6.90E-01	-3.90E-01	-4.41E-01	-3.39E-01	5.05E-02
VAL	36	N	534	LEU	34	C	1125	5.85E-01	-5.34E-01	-5.82E-01	-4.86E-01	4.78E-02
VAL	36	N	534	MET	35	C	1144	5.32E-01	-6.90E-01	-7.55E-01	-6.26E-01	6.44E-02
VAL	36	N	534	MET	35	H	1150	4.15E-01	-6.17E-01	-7.23E-01	-5.12E-01	1.05E-01
VAL	36	N	534	VAL	36	C	1161	5.60E-01	-4.54E-01	-4.78E-01	-4.30E-01	2.39E-02

VAL	36	N	534	VAL	36	H	1166	4.13E-01	-9.33E-01	-9.83E-01	-8.84E-01	4.97E-02
VAL	36	C	536	VAL	36	N	1159	5.26E-01	-5.26E-01	-5.71E-01	-4.81E-01	4.49E-02
VAL	36	C	536	VAL	36	O	1162	3.72E-01	-1.13E+00	-1.22E+00	-1.04E+00	8.63E-02
VAL	36	C	536	GLY	37	N	1175	5.74E-01	-3.56E-01	-3.70E-01	-3.42E-01	1.40E-02
VAL	36	O	537	VAL	36	C	1161	6.01E-01	-3.49E-01	-3.59E-01	-3.38E-01	1.01E-02
VAL	36	O	537	VAL	36	H	1166	5.87E-01	-3.62E-01	-3.74E-01	-3.49E-01	1.25E-02
VAL	36	CB	538	MET	35	N	1142	5.44E-01	-3.83E-01	-4.29E-01	-3.38E-01	4.53E-02
VAL	36	CB	538	VAL	36	N	1159	4.62E-01	-6.34E-01	-6.89E-01	-5.79E-01	5.47E-02
VAL	36	CB	538	VAL	36	O	1162	3.93E-01	-8.69E-01	-9.61E-01	-7.76E-01	9.27E-02
VAL	36	H	541	MET	35	N	1142	5.37E-01	-4.43E-01	-5.05E-01	-3.82E-01	6.14E-02
VAL	36	H	541	VAL	36	N	1159	5.66E-01	-4.36E-01	-4.54E-01	-4.17E-01	1.82E-02
VAL	36	H	541	VAL	36	O	1162	5.52E-01	-4.19E-01	-4.52E-01	-3.85E-01	3.38E-02
GLY	37	N	550	MET	35	C	1144	5.81E-01	-4.98E-01	-6.06E-01	-3.91E-01	1.07E-01
GLY	37	N	550	VAL	36	C	1161	4.18E-01	-7.70E-01	-8.41E-01	-7.00E-01	7.06E-02
GLY	37	N	550	VAL	36	H	1166	4.86E-01	-5.19E-01	-5.55E-01	-4.83E-01	3.64E-02
GLY	37	N	550	GLY	37	C	1177	4.83E-01	-7.37E-01	-8.77E-01	-5.96E-01	1.41E-01
GLY	37	C	552	VAL	36	N	1159	7.19E-01	-3.54E-01	-3.80E-01	-3.29E-01	2.56E-02
GLY	37	C	552	VAL	36	O	1162	4.68E-01	-8.25E-01	-9.27E-01	-7.22E-01	1.02E-01
GLY	37	C	552	GLY	37	N	1175	5.92E-01	-4.44E-01	-5.01E-01	-3.88E-01	5.65E-02
GLY	37	C	552	GLY	37	O	1178	4.81E-01	-1.11E+00	-1.42E+00	-7.92E-01	3.14E-01
GLY	37	C	552	GLY	38	N	1182	4.99E-01	-6.84E-01	-8.44E-01	-5.25E-01	1.60E-01
GLY	37	C	552	GLY	38	O	1185	7.07E-01	-4.16E-01	-4.56E-01	-3.75E-01	4.02E-02
GLY	37	C	552	VAL	39	N	1189	6.62E-01	-4.18E-01	-4.56E-01	-3.80E-01	3.79E-02
GLY	37	O	553	VAL	36	C	1161	5.94E-01	-4.83E-01	-5.82E-01	-3.84E-01	9.94E-02
GLY	37	O	553	GLY	37	C	1177	5.02E-01	-9.88E-01	-1.28E+00	-6.94E-01	2.93E-01
GLY	37	O	553	GLY	38	C	1184	6.46E-01	-5.43E-01	-7.01E-01	-3.85E-01	1.58E-01
GLY	37	H	554	VAL	36	N	1159	4.35E-01	-4.93E-01	-5.86E-01	-3.99E-01	9.34E-02
GLY	37	H	554	VAL	36	O	1162	2.25E-01	-3.43E+00	-4.23E+00	-2.63E+00	8.04E-01
GLY	37	H	554	GLY	37	N	1175	3.95E-01	-5.02E-01	-5.33E-01	-4.72E-01	3.08E-02
GLY	38	N	557	GLY	37	C	1177	4.97E-01	-6.85E-01	-8.27E-01	-5.44E-01	1.41E-01
GLY	38	N	557	GLY	38	C	1184	5.91E-01	-4.41E-01	-4.80E-01	-4.03E-01	3.84E-02
GLY	38	C	559	VAL	36	O	1162	6.19E-01	-4.54E-01	-5.45E-01	-3.62E-01	9.11E-02
GLY	38	C	559	GLY	37	O	1178	5.30E-01	-9.00E-01	-1.21E+00	-5.89E-01	3.11E-01
GLY	38	C	559	GLY	38	N	1182	4.86E-01	-7.17E-01	-8.54E-01	-5.79E-01	1.37E-01
GLY	38	C	559	GLY	38	O	1185	5.84E-01	-6.06E-01	-6.23E-01	-5.89E-01	1.69E-02
GLY	38	C	559	VAL	39	N	1189	4.52E-01	-9.74E-01	-1.01E+00	-9.34E-01	3.98E-02
GLY	38	C	559	VAL	39	O	1192	4.97E-01	-7.04E-01	-7.52E-01	-6.55E-01	4.84E-02
GLY	38	C	559	VAL	40	N	1205	6.99E-01	-3.70E-01	-3.83E-01	-3.57E-01	1.30E-02
GLY	38	O	560	VAL	36	C	1161	5.98E-01	-4.70E-01	-5.55E-01	-3.84E-01	8.55E-02
GLY	38	O	560	GLY	37	C	1177	4.50E-01	-1.15E+00	-1.31E+00	-9.95E-01	1.60E-01
GLY	38	O	560	GLY	38	C	1184	3.94E-01	-1.58E+00	-1.73E+00	-1.44E+00	1.43E-01
GLY	38	O	560	GLY	38	H	1186	4.26E-01	-1.01E+00	-1.72E+00	-2.89E-01	7.18E-01
GLY	38	O	560	VAL	39	C	1191	5.22E-01	-6.07E-01	-6.56E-01	-5.57E-01	4.96E-02
GLY	38	O	560	VAL	39	CB	1193	4.80E-01	-6.53E-01	-6.98E-01	-6.08E-01	4.49E-02
GLY	38	O	560	VAL	39	H	1196	3.39E-01	-2.00E+00	-2.56E+00	-1.44E+00	5.57E-01
VAL	39	N	564	GLY	37	C	1177	6.46E-01	-4.38E-01	-4.72E-01	-4.03E-01	3.44E-02

VAL	39	N	564	GLY	38	C	1184	5.44E-01	-6.26E-01	-6.41E-01	-6.10E-01	1.54E-02
VAL	39	N	564	VAL	39	C	1191	5.45E-01	-4.82E-01	-5.05E-01	-4.59E-01	2.27E-02
VAL	39	N	564	VAL	39	CB	1193	5.75E-01	-3.78E-01	-3.91E-01	-3.64E-01	1.36E-02
VAL	39	N	564	VAL	39	H	1196	4.09E-01	-9.54E-01	-1.02E+00	-8.92E-01	6.13E-02
VAL	39	C	566	VAL	39	N	1189	5.37E-01	-5.00E-01	-5.26E-01	-4.74E-01	2.62E-02
VAL	39	C	566	VAL	39	O	1192	3.63E-01	-1.20E+00	-1.25E+00	-1.14E+00	5.48E-02
VAL	39	C	566	VAL	40	N	1205	5.77E-01	-4.23E-01	-4.34E-01	-4.11E-01	1.17E-02
VAL	39	C	566	ILE	41	O	1224	7.69E-01	-3.29E-01	-3.44E-01	-3.15E-01	1.48E-02
VAL	39	O	567	VAL	39	C	1191	6.02E-01	-3.48E-01	-3.52E-01	-3.43E-01	4.99E-03
VAL	39	O	567	VAL	39	H	1196	5.80E-01	-3.75E-01	-4.02E-01	-3.47E-01	2.76E-02
VAL	39	CB	568	VAL	39	N	1189	5.09E-01	-5.03E-01	-5.45E-01	-4.61E-01	4.20E-02
VAL	39	CB	568	VAL	39	O	1192	4.23E-01	-7.32E-01	-8.32E-01	-6.31E-01	1.01E-01
VAL	39	H	571	VAL	39	N	1189	5.63E-01	-4.40E-01	-4.51E-01	-4.29E-01	1.13E-02
VAL	39	H	571	VAL	39	O	1192	5.14E-01	-4.91E-01	-5.25E-01	-4.56E-01	3.43E-02
VAL	40	N	580	GLY	38	C	1184	6.34E-01	-4.52E-01	-4.74E-01	-4.30E-01	2.23E-02
VAL	40	N	580	VAL	39	C	1191	4.06E-01	-9.85E-01	-1.02E+00	-9.52E-01	3.24E-02
VAL	40	N	580	VAL	39	CB	1193	5.41E-01	-4.38E-01	-4.78E-01	-3.98E-01	3.99E-02
VAL	40	N	580	VAL	39	H	1196	4.54E-01	-7.44E-01	-8.30E-01	-6.57E-01	8.62E-02
VAL	40	N	580	VAL	40	C	1207	5.57E-01	-4.60E-01	-4.86E-01	-4.34E-01	2.61E-02
VAL	40	N	580	VAL	40	CB	1209	5.40E-01	-4.36E-01	-4.61E-01	-4.12E-01	2.47E-02
VAL	40	N	580	VAL	40	H	1212	5.76E-01	-4.19E-01	-4.25E-01	-4.12E-01	6.53E-03
VAL	40	N	580	ILE	41	C	1223	7.05E-01	-3.58E-01	-3.77E-01	-3.39E-01	1.91E-02
VAL	40	N	580	ILE	41	H	1229	4.53E-01	-5.62E-01	-6.33E-01	-4.91E-01	7.10E-02
VAL	40	C	582	VAL	39	O	1192	4.11E-01	-8.70E-01	-9.57E-01	-7.84E-01	8.64E-02
VAL	40	C	582	VAL	40	N	1205	5.21E-01	-5.34E-01	-5.57E-01	-5.10E-01	2.40E-02
VAL	40	C	582	VAL	40	O	1208	6.01E-01	-3.48E-01	-3.55E-01	-3.41E-01	6.84E-03
VAL	40	C	582	ILE	41	N	1221	4.08E-01	-9.74E-01	-1.02E+00	-9.32E-01	4.21E-02
VAL	40	C	582	ILE	41	O	1224	4.30E-01	-1.19E+00	-1.30E+00	-1.07E+00	1.16E-01
VAL	40	O	583	LYS	28	C	1041	8.00E-01	-3.24E-01	-3.30E-01	-3.17E-01	6.20E-03
VAL	40	O	583	VAL	39	C	1191	4.31E-01	-7.74E-01	-8.57E-01	-6.90E-01	8.36E-02
VAL	40	O	583	VAL	40	C	1207	3.59E-01	-1.23E+00	-1.24E+00	-1.21E+00	1.97E-02
VAL	40	O	583	VAL	40	CB	1209	4.26E-01	-6.95E-01	-7.33E-01	-6.56E-01	3.84E-02
VAL	40	O	583	VAL	40	H	1212	5.13E-01	-4.91E-01	-5.23E-01	-4.60E-01	3.14E-02
VAL	40	O	583	ILE	41	C	1223	4.15E-01	-1.09E+00	-1.24E+00	-9.47E-01	1.46E-01
VAL	40	O	583	ILE	41	H	1229	1.96E-01	-5.24E+00	-5.59E+00	-4.88E+00	3.54E-01
VAL	40	CB	584	VAL	39	O	1192	4.14E-01	-7.88E-01	-9.51E-01	-6.25E-01	1.63E-01
VAL	40	CB	584	VAL	40	N	1205	5.39E-01	-4.38E-01	-4.66E-01	-4.10E-01	2.78E-02
VAL	40	CB	584	ILE	41	N	1221	5.38E-01	-4.40E-01	-4.65E-01	-4.15E-01	2.50E-02
VAL	40	CB	584	ILE	41	O	1224	6.00E-01	-4.77E-01	-5.08E-01	-4.45E-01	3.16E-02
VAL	40	H	587	GLY	38	O	1185	6.45E-01	-3.72E-01	-3.89E-01	-3.56E-01	1.66E-02
VAL	40	H	587	VAL	39	N	1189	4.36E-01	-8.18E-01	-8.96E-01	-7.39E-01	7.85E-02
VAL	40	H	587	VAL	39	O	1192	1.96E-01	-6.96E+00	-7.28E+00	-6.63E+00	3.27E-01
VAL	40	H	587	VAL	40	N	1205	3.88E-01	-1.10E+00	-1.14E+00	-1.05E+00	4.46E-02
VAL	40	H	587	ILE	41	N	1221	4.76E-01	-6.62E-01	-7.36E-01	-5.88E-01	7.41E-02
VAL	40	H	587	ILE	41	O	1224	6.31E-01	-4.77E-01	-5.07E-01	-4.47E-01	3.01E-02
ILE	41	N	596	LYS	28	C	1041	8.05E-01	-3.57E-01	-3.69E-01	-3.46E-01	1.13E-02

ILE	41	N	596	VAL	39	C	1191	6.25E-01	-3.59E-01	-3.76E-01	-3.41E-01	1.73E-02
ILE	41	N	596	VAL	40	C	1207	5.74E-01	-4.29E-01	-4.34E-01	-4.25E-01	4.34E-03
ILE	41	N	596	ILE	41	C	1223	5.24E-01	-6.70E-01	-6.91E-01	-6.49E-01	2.06E-02
ILE	41	N	596	ILE	41	H	1229	3.85E-01	-8.33E-01	-8.44E-01	-8.21E-01	1.15E-02
ILE	41	C	598	LYS	28	O	1042	6.35E-01	-5.52E-01	-5.74E-01	-5.30E-01	2.18E-02
ILE	41	C	598	ILE	41	N	1221	5.54E-01	-5.91E-01	-5.98E-01	-5.84E-01	6.88E-03
ILE	41	C	598	ILE	41	O	1224	3.59E-01	-2.39E+00	-2.44E+00	-2.34E+00	5.08E-02
ILE	41	C	598	ALA	42	N	1240	5.72E-01	-4.93E-01	-5.00E-01	-4.86E-01	7.22E-03
ILE	41	C	598	ALA	42	O	1243	6.74E-01	-4.85E-01	-5.19E-01	-4.51E-01	3.37E-02
ILE	41	O	599	LYS	28	C	1041	8.14E-01	-4.82E-01	-5.02E-01	-4.63E-01	1.95E-02
ILE	41	O	599	VAL	40	C	1207	7.81E-01	-3.19E-01	-3.21E-01	-3.16E-01	2.50E-03
ILE	41	O	599	ILE	41	C	1223	6.01E-01	-6.78E-01	-6.88E-01	-6.69E-01	9.50E-03
ILE	41	O	599	ILE	41	H	1229	5.88E-01	-4.12E-01	-4.22E-01	-4.03E-01	9.29E-03
ILE	41	O	599	ALA	42	C	1242	6.72E-01	-5.43E-01	-5.71E-01	-5.14E-01	2.86E-02
ILE	41	H	604	ILE	41	N	1221	5.76E-01	-3.13E-01	-3.19E-01	-3.08E-01	5.66E-03
ILE	41	H	604	ILE	41	O	1224	5.14E-01	-5.57E-01	-5.69E-01	-5.44E-01	1.24E-02
ILE	41	HA	605	ILE	41	N	1221	3.55E-01	-5.49E-01	-5.73E-01	-5.24E-01	2.48E-02
ILE	41	HA	605	ILE	41	O	1224	2.45E-01	-2.16E+00	-2.19E+00	-2.12E+00	3.56E-02
ALA	42	N	615	LYS	28	C	1041	7.00E-01	-4.15E-01	-4.29E-01	-4.01E-01	1.43E-02
ALA	42	N	615	ILE	41	C	1223	4.10E-01	-1.10E+00	-1.12E+00	-1.08E+00	2.36E-02
ALA	42	N	615	ILE	41	H	1229	4.80E-01	-4.30E-01	-4.45E-01	-4.14E-01	1.57E-02
ALA	42	N	615	ALA	42	C	1242	4.52E-01	-8.79E-01	-9.67E-01	-7.90E-01	8.82E-02
ALA	42	C	617	LYS	28	O	1042	6.42E-01	-5.42E-01	-5.78E-01	-5.07E-01	3.52E-02
ALA	42	C	617	ILE	41	O	1224	4.82E-01	-1.13E+00	-1.20E+00	-1.06E+00	7.12E-02
ALA	42	C	617	ALA	42	N	1240	6.13E-01	-4.28E-01	-4.51E-01	-4.05E-01	2.26E-02
ALA	42	C	617	ALA	42	O	1243	5.40E-01	-7.84E-01	-8.50E-01	-7.19E-01	6.53E-02
ALA	42	O	618	LYS	28	C	1041	7.39E-01	-5.23E-01	-5.77E-01	-4.68E-01	5.45E-02
ALA	42	O	618	ILE	41	C	1223	6.10E-01	-6.09E-01	-7.00E-01	-5.19E-01	9.04E-02
ALA	42	O	618	ALA	42	C	1242	4.44E-01	-1.27E+00	-1.47E+00	-1.08E+00	1.94E-01
ALA	42	CB	619	ILE	41	C	1223	4.78E-01	-4.27E-01	-4.50E-01	-4.05E-01	2.27E-02
ALA	42	CB	619	ALA	42	C	1242	3.89E-01	-7.77E-01	-9.24E-01	-6.29E-01	1.48E-01
ALA	42	H	620	ILE	41	N	1221	4.59E-01	-4.76E-01	-4.85E-01	-4.66E-01	9.23E-03
ALA	42	H	620	ILE	41	O	1224	1.98E-01	-6.92E+00	-7.19E+00	-6.65E+00	2.70E-01
ALA	42	H	620	ALA	42	N	1240	3.85E-01	-6.71E-01	-6.85E-01	-6.57E-01	1.38E-02
ALA	42	H	620	ALA	42	O	1243	4.86E-01	-5.22E-01	-5.80E-01	-4.64E-01	5.79E-02

Supplementary Table 6b: Mapping results for Aβ42's (PDB ID: 2NAO) short range (1:2) dominant atom-atom Lennard-Jones interactions across ensemble structures. Columns for each chain correspond to: residue abbreviation, residue number in peptide sequence, atom identity (IUPAC naming convention) and atom number in PDB file. Energy in *kT*, distance in *nm*. Mapping analysis began on the 11th residue for both isoforms because original structure data for Aβ42 begins with the 11th residue.

Chain 1				Chain 2				Average Distance	Average L-J Values	Lower 95% Confidence Interval Bound	Upper 95% Confidence Interval Bound	Margin of Error
HIS	14	CA	199	HIS	14	O	826	4.19E-01	-1.54E-01	-1.71E-01	-1.37E-01	1.70E-02
HIS	14	C	200	HIS	14	O	826	3.86E-01	-1.88E-01	-1.96E-01	-1.79E-01	8.81E-03
GLN	15	N	215	HIS	14	C	825	3.89E-01	-1.87E-01	-1.90E-01	-1.84E-01	2.82E-03
GLN	15	C	217	LYS	16	N	857	4.12E-01	-1.57E-01	-1.62E-01	-1.52E-01	5.08E-03
GLN	15	O	218	HIS	14	C	825	3.84E-01	-1.88E-01	-2.07E-01	-1.69E-01	1.90E-02
GLN	15	O	218	GLN	15	N	840	4.19E-01	-1.72E-01	-1.88E-01	-1.56E-01	1.59E-02
GLN	15	O	218	GLN	15	CA	841	3.70E-01	-2.31E-01	-2.42E-01	-2.21E-01	1.05E-02
GLN	15	O	218	GLN	15	C	842	3.77E-01	-2.01E-01	-2.06E-01	-1.95E-01	5.70E-03
GLN	15	O	218	LYS	16	CA	858	3.80E-01	-2.20E-01	-2.29E-01	-2.11E-01	9.28E-03
GLN	15	O	218	LYS	16	O	860	3.97E-01	-1.99E-01	-2.34E-01	-1.65E-01	3.41E-02
GLN	15	CB	219	HIS	14	C	825	4.15E-01	-1.33E-01	-1.42E-01	-1.23E-01	9.21E-03
GLN	15	CB	219	GLN	15	CA	841	4.18E-01	-1.46E-01	-1.50E-01	-1.42E-01	3.99E-03
GLN	15	CG	220	HIS	14	O	826	4.18E-01	-1.55E-01	-1.81E-01	-1.30E-01	2.58E-02
LYS	16	CA	233	LYS	16	O	860	4.04E-01	-1.79E-01	-1.98E-01	-1.60E-01	1.88E-02
LYS	16	C	234	LYS	16	O	860	3.86E-01	-1.87E-01	-1.90E-01	-1.84E-01	2.54E-03
LEU	17	N	254	LYS	16	C	859	4.02E-01	-1.71E-01	-1.82E-01	-1.60E-01	1.09E-02
LEU	17	C	256	VAL	18	N	898	4.21E-01	-1.45E-01	-1.47E-01	-1.43E-01	2.18E-03
LEU	17	C	256	VAL	18	O	901	3.84E-01	-1.86E-01	-2.03E-01	-1.69E-01	1.70E-02
LEU	17	O	257	LEU	17	CA	880	3.74E-01	-2.21E-01	-2.35E-01	-2.06E-01	1.45E-02
LEU	17	O	257	LEU	17	C	881	3.84E-01	-1.90E-01	-1.98E-01	-1.83E-01	7.42E-03
LEU	17	O	257	VAL	18	CA	899	3.86E-01	-2.09E-01	-2.23E-01	-1.96E-01	1.32E-02
LEU	17	O	257	VAL	18	C	900	3.73E-01	-1.94E-01	-2.05E-01	-1.83E-01	1.11E-02
LEU	17	CB	258	LEU	17	CA	880	4.33E-01	-1.28E-01	-1.35E-01	-1.21E-01	7.25E-03
LEU	17	CB	258	LEU	17	CG	884	4.23E-01	-1.40E-01	-1.50E-01	-1.30E-01	1.02E-02
LEU	17	CB	258	LEU	17	CD1	885	3.78E-01	-1.68E-01	-1.75E-01	-1.60E-01	7.58E-03
LEU	17	CG	259	LEU	17	CD1	885	4.22E-01	-1.42E-01	-1.46E-01	-1.37E-01	4.84E-03
LEU	17	CD2	261	LEU	17	CG	884	4.04E-01	-1.61E-01	-1.68E-01	-1.53E-01	7.52E-03
VAL	18	N	273	VAL	18	O	901	4.03E-01	-2.06E-01	-2.29E-01	-1.83E-01	2.31E-02
VAL	18	CA	274	VAL	18	C	900	4.26E-01	-1.20E-01	-1.27E-01	-1.14E-01	6.83E-03
VAL	18	C	275	VAL	18	O	901	4.18E-01	-1.38E-01	-1.51E-01	-1.25E-01	1.32E-02
PHE	19	N	289	PHE	19	CA	915	4.45E-01	-1.28E-01	-1.35E-01	-1.20E-01	7.68E-03
PHE	19	CB	293	PHE	19	CG	919	4.28E-01	-1.18E-01	-1.24E-01	-1.13E-01	5.44E-03
PHE	19	CB	293	PHE	19	CD1	920	4.18E-01	-1.29E-01	-1.40E-01	-1.19E-01	1.03E-02
PHE	19	CB	293	PHE	19	CE1	922	3.93E-01	-1.40E-01	-1.48E-01	-1.32E-01	7.84E-03
PHE	20	CZ	319	PHE	19	CE2	923	4.10E-01	-1.22E-01	-1.29E-01	-1.16E-01	6.20E-03
ASP	23	CA	355	ASP	23	O	982	3.86E-01	-1.81E-01	-2.07E-01	-1.56E-01	2.56E-02
VAL	24	N	366	ASP	23	O	982	4.09E-01	-1.96E-01	-2.40E-01	-1.53E-01	4.34E-02
GLY	25	O	385	GLY	25	C	1009	4.15E-01	-1.41E-01	-1.49E-01	-1.33E-01	7.98E-03
GLY	25	O	385	SER	26	N	1014	3.94E-01	-2.25E-01	-2.52E-01	-1.98E-01	2.67E-02

SER	26	CA	390	SER	26	O	1017	4.26E-01	-1.41E-01	-1.49E-01	-1.32E-01	8.23E-03
SER	26	C	391	SER	26	O	1017	3.99E-01	-1.67E-01	-1.70E-01	-1.63E-01	3.20E-03
ASN	27	N	400	SER	26	C	1016	4.05E-01	-1.66E-01	-1.69E-01	-1.63E-01	3.23E-03
ASN	27	CA	401	SER	26	O	1017	3.36E-01	-1.84E-01	-2.12E-01	-1.56E-01	2.81E-02
ASN	27	C	402	SER	26	O	1017	3.85E-01	-1.88E-01	-2.01E-01	-1.75E-01	1.30E-02
ASN	27	C	402	LYS	28	N	1039	3.89E-01	-1.87E-01	-1.89E-01	-1.84E-01	2.72E-03
ASN	27	C	402	LYS	28	O	1042	3.86E-01	-1.78E-01	-1.98E-01	-1.59E-01	1.95E-02
ASN	27	O	403	SER	26	O	1017	3.62E-01	-2.85E-01	-3.10E-01	-2.60E-01	2.52E-02
ASN	27	O	403	ASN	27	CA	1026	4.29E-01	-1.38E-01	-1.46E-01	-1.29E-01	8.38E-03
ASN	27	O	403	ASN	27	C	1027	3.90E-01	-1.81E-01	-1.84E-01	-1.79E-01	2.68E-03
ASN	27	O	403	GLY	29	N	1061	4.01E-01	-2.10E-01	-2.41E-01	-1.79E-01	3.14E-02
ASN	27	CB	404	SER	26	C	1016	4.09E-01	-1.39E-01	-1.45E-01	-1.32E-01	6.76E-03
ASN	27	CB	404	ASN	27	CA	1026	4.05E-01	-1.61E-01	-1.64E-01	-1.59E-01	2.60E-03
ASN	27	CB	404	LYS	28	N	1039	4.15E-01	-1.72E-01	-1.85E-01	-1.60E-01	1.24E-02
ASN	27	CG	405	SER	26	C	1016	3.85E-01	-1.36E-01	-1.39E-01	-1.32E-01	3.57E-03
ASN	27	CG	405	ASN	27	CA	1026	4.24E-01	-1.23E-01	-1.30E-01	-1.15E-01	7.61E-03
LYS	28	N	414	LYS	28	O	1042	4.14E-01	-1.85E-01	-2.12E-01	-1.57E-01	2.78E-02
LYS	28	CA	415	LYS	28	O	1042	3.49E-01	-2.17E-01	-2.34E-01	-2.00E-01	1.69E-02
LYS	28	C	416	LYS	28	O	1042	3.75E-01	-2.03E-01	-2.08E-01	-1.97E-01	5.65E-03
LYS	28	CD	420	LYS	28	CB	1043	3.96E-01	-1.68E-01	-1.75E-01	-1.62E-01	6.47E-03
GLY	29	N	436	GLY	29	CA	1062	4.49E-01	-1.23E-01	-1.31E-01	-1.14E-01	9.00E-03
GLY	29	CA	437	GLY	29	O	1064	4.20E-01	-1.52E-01	-1.68E-01	-1.35E-01	1.65E-02
GLY	29	C	438	GLY	29	O	1064	3.91E-01	-1.79E-01	-1.91E-01	-1.68E-01	1.13E-02
ALA	30	N	443	GLY	29	C	1063	4.00E-01	-1.72E-01	-1.79E-01	-1.65E-01	6.63E-03
ALA	30	CA	444	PHE	19	CZ	924	4.20E-01	-1.27E-01	-1.33E-01	-1.21E-01	5.78E-03
ALA	30	CA	444	GLY	29	O	1064	3.66E-01	-2.16E-01	-2.34E-01	-1.97E-01	1.83E-02
ALA	30	C	445	ALA	30	CA	1069	4.38E-01	-1.08E-01	-1.10E-01	-1.06E-01	1.96E-03
ALA	30	C	445	ILE	31	N	1078	4.17E-01	-1.50E-01	-1.52E-01	-1.48E-01	1.82E-03
ALA	30	O	446	PHE	19	CE1	922	4.05E-01	-1.58E-01	-1.63E-01	-1.53E-01	5.05E-03
ALA	30	O	446	PHE	19	CE2	923	3.80E-01	-1.93E-01	-2.07E-01	-1.80E-01	1.34E-02
ALA	30	O	446	GLY	29	C	1063	3.53E-01	-2.14E-01	-2.16E-01	-2.11E-01	2.24E-03
ALA	30	O	446	ALA	30	N	1068	3.85E-01	-2.45E-01	-2.54E-01	-2.36E-01	9.08E-03
ALA	30	O	446	ALA	30	C	1070	3.61E-01	-2.17E-01	-2.17E-01	-2.16E-01	5.85E-04
ALA	30	O	446	ALA	30	CB	1072	4.44E-01	-1.16E-01	-1.18E-01	-1.13E-01	2.54E-03
ALA	30	O	446	ILE	31	CA	1079	4.06E-01	-1.74E-01	-1.77E-01	-1.71E-01	3.10E-03
ALA	30	O	446	ILE	31	C	1080	4.33E-01	-1.16E-01	-1.19E-01	-1.13E-01	2.80E-03
ALA	30	O	446	ILE	31	O	1081	3.74E-01	-2.53E-01	-2.60E-01	-2.46E-01	7.27E-03
ALA	30	O	446	ILE	31	CG1	1083	3.98E-01	-1.89E-01	-1.99E-01	-1.80E-01	9.64E-03
ALA	30	CB	447	GLY	29	O	1064	4.09E-01	-1.70E-01	-1.93E-01	-1.46E-01	2.37E-02
ALA	30	CB	447	ALA	30	CA	1069	4.43E-01	-1.16E-01	-1.18E-01	-1.14E-01	1.92E-03
ILE	31	N	453	GLY	29	O	1064	4.36E-01	-1.41E-01	-1.51E-01	-1.30E-01	1.04E-02
ILE	31	N	453	ILE	31	O	1081	4.29E-01	-1.52E-01	-1.58E-01	-1.46E-01	5.94E-03
ILE	31	CA	454	ILE	31	N	1078	4.51E-01	-1.19E-01	-1.22E-01	-1.16E-01	2.91E-03
ILE	31	CA	454	ILE	31	CG1	1083	3.67E-01	-1.64E-01	-1.66E-01	-1.62E-01	2.46E-03
ILE	31	CA	454	ILE	31	CD1	1085	3.92E-01	-1.73E-01	-1.75E-01	-1.70E-01	2.29E-03
ILE	31	C	455	ILE	31	O	1081	3.63E-01	-2.16E-01	-2.17E-01	-2.14E-01	1.51E-03

ILE	31	CB	457	ILE	31	O	1081	4.06E-01	-1.74E-01	-1.82E-01	-1.67E-01	7.53E-03
ILE	31	CG2	459	GLY	29	O	1064	4.03E-01	-1.81E-01	-2.05E-01	-1.57E-01	2.44E-02
ILE	31	CG2	459	ILE	31	CB	1082	4.49E-01	-1.09E-01	-1.11E-01	-1.06E-01	2.43E-03
ILE	31	CG2	459	ILE	31	CD1	1085	3.81E-01	-1.75E-01	-1.78E-01	-1.73E-01	2.49E-03
ILE	32	N	472	ILE	31	C	1080	4.18E-01	-1.49E-01	-1.51E-01	-1.47E-01	2.15E-03
ILE	32	N	472	ILE	31	CG1	1083	4.60E-01	-1.09E-01	-1.11E-01	-1.06E-01	2.24E-03
ILE	32	N	472	ILE	31	CD1	1085	4.16E-01	-1.71E-01	-1.77E-01	-1.66E-01	5.25E-03
ILE	32	CA	473	ILE	31	O	1081	4.07E-01	-1.74E-01	-1.76E-01	-1.71E-01	2.47E-03
ILE	32	C	474	ILE	31	O	1081	4.23E-01	-1.29E-01	-1.36E-01	-1.22E-01	6.96E-03
ILE	32	C	474	ILE	32	CA	1098	4.23E-01	-1.24E-01	-1.28E-01	-1.21E-01	3.29E-03
ILE	32	O	475	ILE	32	CA	1098	3.69E-01	-2.17E-01	-2.45E-01	-1.88E-01	2.85E-02
ILE	32	O	475	ILE	32	CB	1101	3.88E-01	-2.05E-01	-2.29E-01	-1.80E-01	2.41E-02
ILE	32	O	475	ILE	32	CD1	1104	3.99E-01	-1.86E-01	-2.09E-01	-1.62E-01	2.32E-02
ILE	32	CG2	478	PHE	19	CD1	920	4.01E-01	-1.44E-01	-1.50E-01	-1.38E-01	5.90E-03
ILE	32	CG2	478	ILE	31	O	1081	3.92E-01	-2.00E-01	-2.19E-01	-1.82E-01	1.88E-02
GLY	33	CA	492	GLY	33	N	1116	4.30E-01	-1.49E-01	-1.56E-01	-1.41E-01	7.72E-03
MET	35	C	519	MET	35	N	1142	4.19E-01	-1.47E-01	-1.58E-01	-1.36E-01	1.06E-02
MET	35	C	519	MET	35	CA	1143	4.14E-01	-1.31E-01	-1.41E-01	-1.21E-01	9.75E-03
MET	35	O	520	MET	35	C	1144	3.97E-01	-1.69E-01	-1.85E-01	-1.53E-01	1.64E-02
MET	35	SD	523	MET	35	SD	1148	4.87E-01	-2.13E-01	-2.29E-01	-1.96E-01	1.68E-02
VAL	36	CA	535	VAL	36	N	1159	4.26E-01	-1.56E-01	-1.72E-01	-1.39E-01	1.65E-02
VAL	36	C	536	VAL	36	O	1162	3.72E-01	-2.04E-01	-2.23E-01	-1.86E-01	1.85E-02
VAL	36	CB	538	VAL	36	O	1162	3.93E-01	-1.95E-01	-2.16E-01	-1.74E-01	2.12E-02
GLY	38	O	560	GLY	38	C	1184	3.94E-01	-1.73E-01	-1.90E-01	-1.55E-01	1.70E-02
GLY	38	O	560	VAL	39	N	1189	3.80E-01	-2.41E-01	-2.67E-01	-2.16E-01	2.54E-02
VAL	39	CA	565	VAL	39	N	1189	4.43E-01	-1.31E-01	-1.43E-01	-1.19E-01	1.21E-02
VAL	39	C	566	VAL	39	O	1192	3.63E-01	-2.12E-01	-2.16E-01	-2.08E-01	4.03E-03
VAL	40	N	580	VAL	39	C	1191	4.06E-01	-1.65E-01	-1.71E-01	-1.59E-01	6.27E-03
VAL	40	CA	581	VAL	39	O	1192	3.84E-01	-2.01E-01	-2.13E-01	-1.88E-01	1.21E-02
VAL	40	C	582	VAL	39	O	1192	4.11E-01	-1.47E-01	-1.65E-01	-1.29E-01	1.84E-02
VAL	40	C	582	ILE	41	N	1221	4.08E-01	-1.62E-01	-1.69E-01	-1.54E-01	7.75E-03
VAL	40	O	583	VAL	39	O	1192	3.61E-01	-2.71E-01	-3.05E-01	-2.37E-01	3.41E-02
VAL	40	O	583	VAL	40	CA	1206	3.36E-01	-1.68E-01	-1.96E-01	-1.41E-01	2.74E-02
VAL	40	O	583	VAL	40	C	1207	3.59E-01	-2.17E-01	-2.18E-01	-2.17E-01	5.58E-04
VAL	40	O	583	VAL	40	CB	1209	4.26E-01	-1.42E-01	-1.56E-01	-1.29E-01	1.38E-02
VAL	40	O	583	ILE	41	CA	1222	3.92E-01	-1.98E-01	-2.12E-01	-1.83E-01	1.42E-02
VAL	40	O	583	ILE	41	C	1223	4.15E-01	-1.38E-01	-1.57E-01	-1.20E-01	1.82E-02
ILE	41	N	596	ILE	41	O	1224	4.26E-01	-1.58E-01	-1.69E-01	-1.46E-01	1.18E-02
ILE	41	CA	597	ILE	41	O	1224	3.33E-01	-1.74E-01	-1.94E-01	-1.55E-01	1.98E-02
ILE	41	C	598	ILE	41	O	1224	3.59E-01	-2.17E-01	-2.18E-01	-2.16E-01	7.57E-04
ILE	41	CB	600	ILE	41	O	1224	4.21E-01	-1.51E-01	-1.67E-01	-1.35E-01	1.59E-02
ILE	41	CG2	602	LYS	28	O	1042	3.67E-01	-2.33E-01	-2.41E-01	-2.25E-01	7.73E-03
ILE	41	CG2	602	ILE	41	O	1224	3.97E-01	-1.91E-01	-2.18E-01	-1.65E-01	2.68E-02
ILE	41	CG2	602	ILE	41	CB	1225	4.39E-01	-1.21E-01	-1.29E-01	-1.12E-01	8.10E-03
ALA	42	N	615	ILE	41	C	1223	4.10E-01	-1.60E-01	-1.65E-01	-1.56E-01	4.55E-03
ALA	42	N	615	ALA	42	CA	1241	4.53E-01	-1.17E-01	-1.21E-01	-1.12E-01	4.61E-03

ALA	42	CA	616	ILE	41	O	1224	3.95E-01	-1.95E-01	-2.02E-01	-1.87E-01	7.58E-03
ALA	42	CB	619	ILE	41	O	1224	3.89E-01	-2.05E-01	-2.22E-01	-1.88E-01	1.69E-02
ALA	42	CB	619	ALA	42	CA	1241	3.91E-01	-1.63E-01	-1.75E-01	-1.52E-01	1.16E-02

Supplementary Table 7: Mapping results for Aβ42's (PDB ID: 2NAO) long range (1:3) dominant atom-atom Coulombic interactions across ensemble structures. Columns for each chain correspond to: residue abbreviation, residue number in peptide sequence, atom identity (IUPAC naming convention) and atom number in PDB file. Energy in *kT*, distance in *nm*. Mapping analysis began on the 11th residue for both isoforms because original structure data for Aβ42 begins with the 11th residue.

Chain 1				Chain 2				Average Distance	Average Coulombic Values	Lower 95% Confidence Interval Bound	Upper 95% Confidence Interval Bound	Margin of Error
ALA	2	C	15	GLU	3	O	1276	8.54E-01	-3.33E-01	-3.40E-01	-3.26E-01	6.72E-03
ALA	2	O	16	GLU	3	CD	1279	8.97E-01	-3.91E-01	-4.23E-01	-3.59E-01	3.21E-02
PHE	4	C	40	GLU	3	O	1276	8.34E-01	-3.33E-01	-3.40E-01	-3.27E-01	6.82E-03
PHE	4	O	41	ARG	5	C	1310	8.32E-01	-3.81E-01	-3.89E-01	-3.73E-01	7.97E-03
ARG	5	C	60	ARG	5	O	1311	9.04E-01	-3.79E-01	-3.80E-01	-3.77E-01	1.81E-03
HIS	6	N	82	ARG	5	C	1310	9.18E-01	-3.37E-01	-3.39E-01	-3.36E-01	1.63E-03
HIS	6	C	84	ARG	5	O	1311	8.18E-01	-4.07E-01	-4.25E-01	-3.90E-01	1.75E-02
HIS	6	O	85	ARG	5	C	1310	8.57E-01	-3.80E-01	-3.97E-01	-3.63E-01	1.72E-02
HIS	6	O	85	HIS	6	C	1334	9.11E-01	-3.11E-01	-3.13E-01	-3.08E-01	2.40E-03
HIS	14	C	200	HIS	14	O	1451	8.75E-01	-3.32E-01	-3.38E-01	-3.25E-01	6.43E-03
GLN	15	O	218	HIS	14	C	1450	7.77E-01	-4.35E-01	-4.49E-01	-4.21E-01	1.41E-02
GLN	15	O	218	LYS	16	C	1484	8.48E-01	-4.09E-01	-4.14E-01	-4.03E-01	5.26E-03
GLN	15	CD	221	HIS	14	O	1451	7.68E-01	-4.33E-01	-4.85E-01	-3.81E-01	5.18E-02
LYS	16	C	234	LYS	16	O	1485	8.80E-01	-3.83E-01	-3.87E-01	-3.79E-01	4.14E-03
LEU	17	C	256	LYS	16	O	1485	7.90E-01	-3.66E-01	-3.88E-01	-3.45E-01	2.14E-02
LEU	17	O	257	LYS	16	C	1484	8.09E-01	-4.42E-01	-4.69E-01	-4.14E-01	2.74E-02
LEU	17	O	257	VAL	18	H	1530	7.00E-01	-3.46E-01	-3.51E-01	-3.41E-01	5.25E-03
ASN	27	C	402	SER	26	O	1642	8.24E-01	-3.75E-01	-3.82E-01	-3.69E-01	6.64E-03
ASN	27	C	402	LYS	28	O	1667	7.57E-01	-4.24E-01	-4.38E-01	-4.10E-01	1.38E-02
ASN	27	O	403	LYS	28	C	1666	7.25E-01	-5.01E-01	-5.13E-01	-4.90E-01	1.18E-02
ASN	27	CG	405	SER	26	O	1642	7.07E-01	-4.70E-01	-4.80E-01	-4.60E-01	9.73E-03
ASN	27	ND2	407	GLY	25	C	1634	8.51E-01	-4.63E-01	-5.12E-01	-4.14E-01	4.87E-02
ASN	27	ND2	407	SER	26	C	1641	7.84E-01	-4.48E-01	-5.00E-01	-3.96E-01	5.21E-02
ASN	27	ND2	407	LYS	28	C	1666	9.35E-01	-4.88E-01	-5.21E-01	-4.56E-01	3.28E-02
LYS	28	C	416	LYS	28	O	1667	8.50E-01	-4.06E-01	-4.09E-01	-4.02E-01	3.20E-03
ALA	30	C	445	GLY	29	O	1689	7.48E-01	-3.61E-01	-3.68E-01	-3.55E-01	6.83E-03
ALA	30	C	445	ILE	31	O	1706	8.30E-01	-3.66E-01	-3.69E-01	-3.62E-01	3.76E-03
ALA	30	O	446	LYS	28	C	1666	9.22E-01	-3.51E-01	-3.61E-01	-3.41E-01	9.87E-03
ALA	30	O	446	GLY	29	C	1688	7.42E-01	-4.08E-01	-4.11E-01	-4.04E-01	3.94E-03
ALA	30	O	446	ALA	30	C	1695	8.42E-01	-3.19E-01	-3.21E-01	-3.17E-01	1.73E-03
ALA	30	O	446	ILE	31	C	1705	8.25E-01	-3.30E-01	-3.33E-01	-3.27E-01	2.64E-03
ILE	31	C	455	ILE	31	O	1706	8.47E-01	-3.53E-01	-3.56E-01	-3.49E-01	3.49E-03
ILE	32	C	474	ILE	31	O	1706	8.11E-01	-3.80E-01	-3.82E-01	-3.77E-01	2.81E-03
MET	35	O	520	MET	35	C	1769	8.77E-01	-3.20E-01	-3.26E-01	-3.14E-01	6.36E-03
ILE	41	C	598	ILE	41	O	1849	8.36E-01	-3.61E-01	-3.65E-01	-3.56E-01	4.59E-03
ALA	42	C	617	ILE	41	O	1849	9.02E-01	-3.19E-01	-3.27E-01	-3.11E-01	8.02E-03

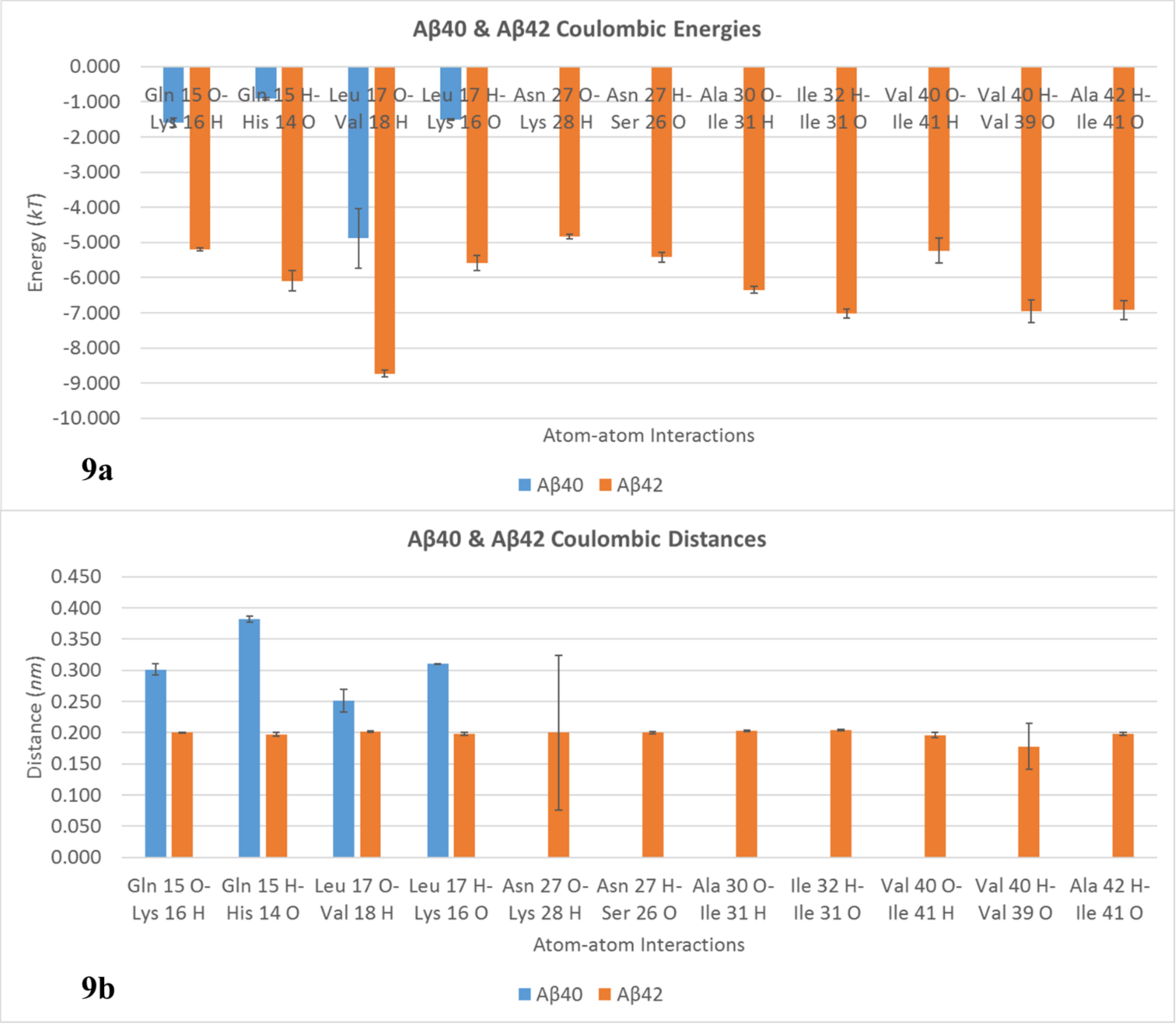

Supplementary Figure 9a & 9b: Atom-atom interactions imparting exceptionally strong hydrogen bonding energies in the 1:2 configuration of Aβ42 (PDB ID: 2NAO by Walti et al.) compared to Aβ40 (Aβ40 PDB ID: 2M4J by Lu et al.; Figure 9a) and their respective atom-atom interaction distances (Figure 9b). 95% confidence interval error bars included for analysis across all ensemble members. The last seven interactions were not observed in Aβ40. Interaction partners are presented as the residue, residue number in the sequence and the residue's atom of one chain (chain A for both strains) interacting with its partner atom in the 1:2 configuration (on the D-chain in Aβ40 or on the B-chain in Aβ42).

Supplementary Table 8: Ramachandran angle data for Aβ40 and Aβ42 for chains A-D (Aβ40, PDB ID: 2M4J) and chains A-B (Aβ42, PDB ID: 2NAO) for the 1:2 interaction configuration across ensemble structures. Data shown starts with residue 11 since structure data for previous Aβ42 structure, 2MXU, begins at residue 11.

	Aβ40				Aβ42			
	Chain A		Chain D		Chain A		Chain B	
	φ	ψ	φ	ψ	φ	ψ	φ	ψ
GLU 11	-	152.910 ± 0.711	-	151.350 ± 0.487	-	-4.240 ± 81.181	-	72.450 ± 59.949
VAL 12	-153.140 ± 0.201	128.500 ± 0.357	-149.530 ± 0.042	130.000 ± 0.072	-93.290 ± 55.607	113.060 ± 35.490	-116.130 ± 22.275	117.79 ± 45.136
HIS 13	-135.180 ± 0.296	146.43 ± 0.743	-129.880 ± 0.120	149.470 ± 0.774	-116.280 ± 29.775	-19.750 ± 91.517	-50.350 ± 61.484	81.920 ± 64.146
HIS 14	-165.320 ± 0.569	97.570 ± 0.373	-165.060 ± 0.128	104.570 ± 0.113	-84.090 ± 52.149	-57.370 ± 75.291	-128.220 ± 19.676	-31.780 ± 46.998
GLN 15	-107.590 ± 2.008	-173.870 ± 0.751	-111.070 ± 2.030	-176.020 ± 0.175	-107.820 ± 6.268	107.650 ± 3.996	-108.000 ± 6.295	107.510 ± 3.976
LYS 16	-166.350 ± 1.700	97.950 ± 0.938	-162.970 ± 1.638	105.720 ± 0.414	-115.340 ± 2.808	108.290 ± 6.669	-115.170 ± 2.661	107.720 ± 6.858
LEU 17	-117.790 ± 5.123	158.580 ± 2.486	-124.310 ± 5.075	156.280 ± 2.852	-110.710 ± 5.090	102.140 ± 2.852	-111.320 ± 4.949	101.870 ± 2.856
VAL 18	-120.610 ± 9.229	124.710 ± 2.477	-112.980 ± 11.974	129.400 ± 2.469	-145.400 ± 6.726	57.450 ± 99.285	-145.160 ± 6.407	57.160 ± 99.162
PHE 19	-109.630 ± 16.980	133.770 ± 10.920	-110.030 ± 20.201	133.850 ± 7.521	-123.310 ± 13.684	34.390 ± 63.122	-123.000 ± 13.958	33.890 ± 62.975
PHE 20	-13.800 ± 67.407	34.610 ± 39.682	-12.140 ± 67.546	30.070 ± 41.404	-50.620 ± 62.655	51.240 ± 95.121	-50.790 ± 62.556	50.620 ± 95.154
ALA 21	-67.680 ± 12.282	-27.670 ± 50.357	-68.800 ± 13.231	-22.500 ± 50.612	-164.370 ± 12.877	2.730 ± 28.591	-164.770 ± 13.002	2.540 ± 28.334
GLU 22	-99.030 ± 13.950	117.940 ± 17.139	-102.000 ± 14.163	120.760 ± 16.175	-83.830 ± 87.209	-55.680 ± 65.302	-84.460 ± 87.238	-55.520 ± 65.156
ASP 23	-153.20 ± 5.623	137.940 ± 1.318	-148.460 ± 3.594	139.130 ± 1.409	-67.700 ± 62.877	102.250 ± 18.593	-67.680 ± 62.879	102.520 ± 18.424
VAL 24	-67.650 ± 8.347	79.540 ± 83.862	-70.530 ± 8.460	80.410 ± 85.689	-111.600 ± 13.845	66.450 ± 87.869	-111.540 ± 13.905	30.280 ± 95.969
GLY 25	-114.140 ± 3.094	50.210 ± 2.290	-114.730 ± 2.429	54.310 ± 3.261	-58.600 ± 39.646	-96.630 ± 12.439	-58.700 ± 39.501	-96.650 ± 12.330
SER 26	-67.480 ± 6.196	-79.970 ± 87.939	-61.550 ± 4.009	-83.240 ± 87.483	-119.940 ± 9.193	134.660 ± 4.032	-120.500 ± 9.244	133.710 ± 3.919
ASN 27	-69.580 ± 1.699	86.480 ± 5.961	-69.260 ± 3.501	91.230 ± 4.565	-95.740 ± 2.109	86.080 ± 3.222	-95.780 ± 2.151	86.650 ± 3.217
LYS 28	-82.690 ± 5.998	135.220 ± 7.876	-87.910 ± 5.247	137.660 ± 8.087	-107.230 ± 4.571	74.870 ± 9.705	-107.790 ± 4.589	75.250 ± 9.670
GLY 29	-62.690 ± 0.809	-153.170 ± 10.428	-60.700 ± 2.630	-151.460 ± 9.486	115.200 ± 10.074	38.470 ± 4.640	114.800 ± 10.144	37.910 ± 4.824
ALA 30	-104.030 ± 6.560	122.180 ± 4.709	-105.320 ± 5.670	123.850 ± 5.584	-85.960 ± 5.934	109.160 ± 3.274	-87.170 ± 6.321	109.000 ± 2.977
ILE 31	-100.480 ± 8.486	130.830 ± 4.794	-103.190 ± 8.379	137.610 ± 3.972	-134.990 ± 2.373	134.630 ± 1.413	-134.880 ± 2.296	134.760 ± 1.463
ILE 32	-147.380 ± 4.687	-78.070 ± 95.222	-152.980 ± 5.812	-77.810 ± 96.409	-146.220 ± 2.943	76.470 ± 11.805	-146.440 ± 2.902	75.930 ± 11.898
GLY 33	63.670 ± 0.773	21.120 ± 16.942	63.990 ± 0.473	20.500 ± 16.506	-129.870 ± 15.586	83.040 ± 66.649	-129.870 ± 15.317	83.050 ± 66.889
LEU 34	-83.930 ± 8.423	126.710 ± 3.472	-84.830 ± 7.589	134.400 ± 2.025	-98.330 ± 27.120	119.26 ± 19.960	-98.540 ± 27.052	119.300 ± 20.052
MET 35	-159.790 ± 2.975	150.550 ± 3.927	-157.400 ± 0.722	160.480 ± 2.626	75.600 ± 22.512	49.090 ± 13.019	75.480 ± 22.453	49.030 ± 13.049
VAL 36	-154.990 ± 2.092	160.240 ± 4.458	-163.110 ± 1.703	161.350 ± 2.732	-99.820 ± 18.036	136.710 ± 11.130	-99.780 ± 18.086	136.680 ± 11.262
GLY 37	55.960 ± 0.472	69.450 ± 2.115	60.710 ± 0.370	64.440 ± 0.783	-88.700 ± 16.855	-43.430 ± 71.753	-88.560 ± 16.874	-43.380 ± 71.808
GLY 38	152.790 ± 7.822	7.410 ± 109.716	150.820 ± 7.315	8.980 ± 110.119	-8.570 ± 65.125	150.790 ± 14.656	-8.720 ± 65.157	150.540 ± 14.674
VAL 39	-122.680 ± 5.114	153.420 ± 4.657	-123.450 ± 4.172	151.310 ± 3.842	-163.010 ± 13.144	133.610 ± 6.114	-163.140 ± 13.185	133.530 ± 6.077
VAL 40	65.280 ± 0.705	-	73.420 ± 1.158	-	-127.460 ± 4.471	120.360 ± 6.579	-127.240 ± 4.432	120.490 ± 6.625
ILE 41	-	-	-	-	-126.180 ± 6.807	129.900 ± 2.870	-125.960 ± 6.786	129.880 ± 2.861
ALA 42	-	-	-	-	-85.260 ± 6.248	-	-85.240 ± 6.240	-

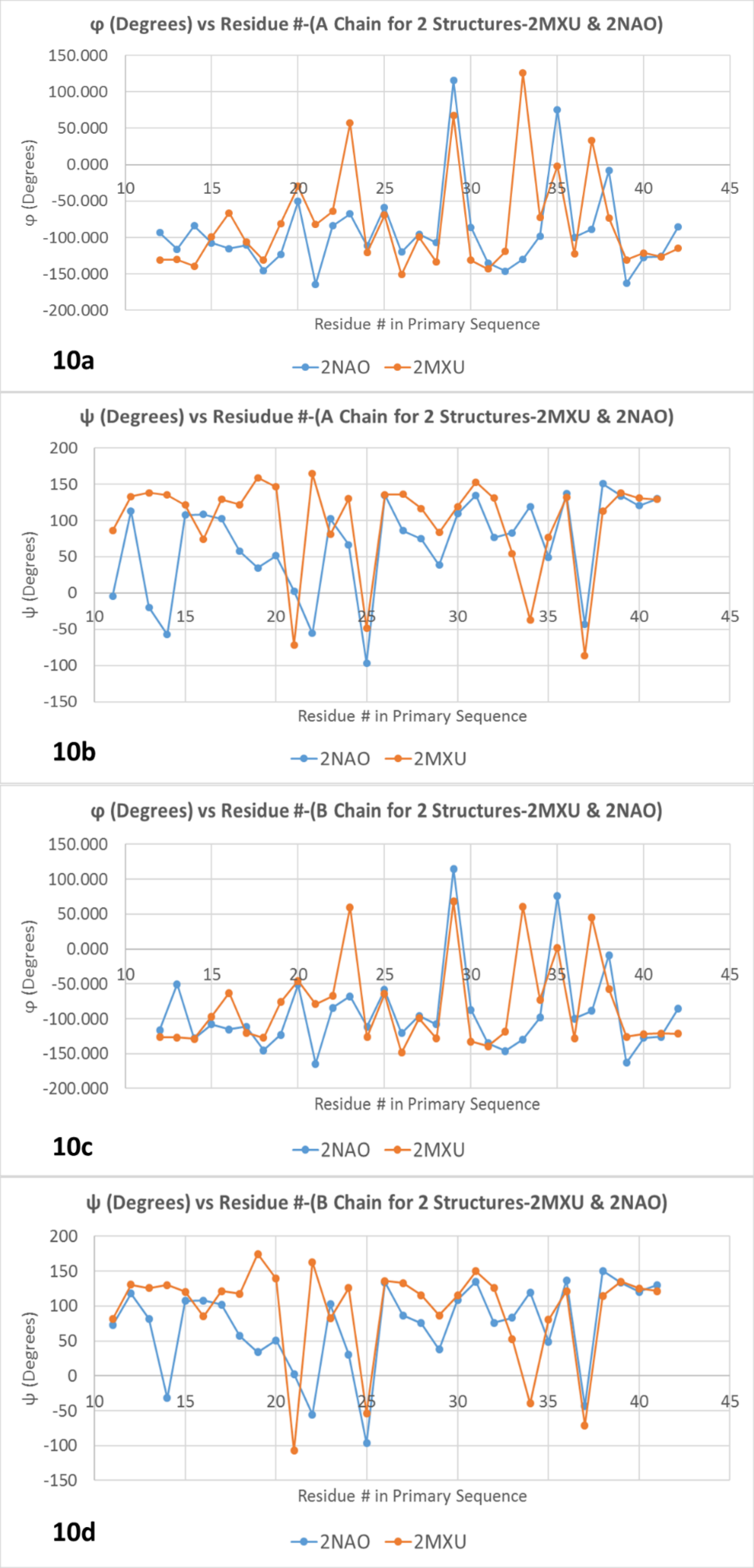

Supplementary Figure 10a-10d: Ramachandran angle profiles for Aβ42 structures 2MXU and 2NAO featured as stacked curves for φ and ψ angle value distribution comparison.

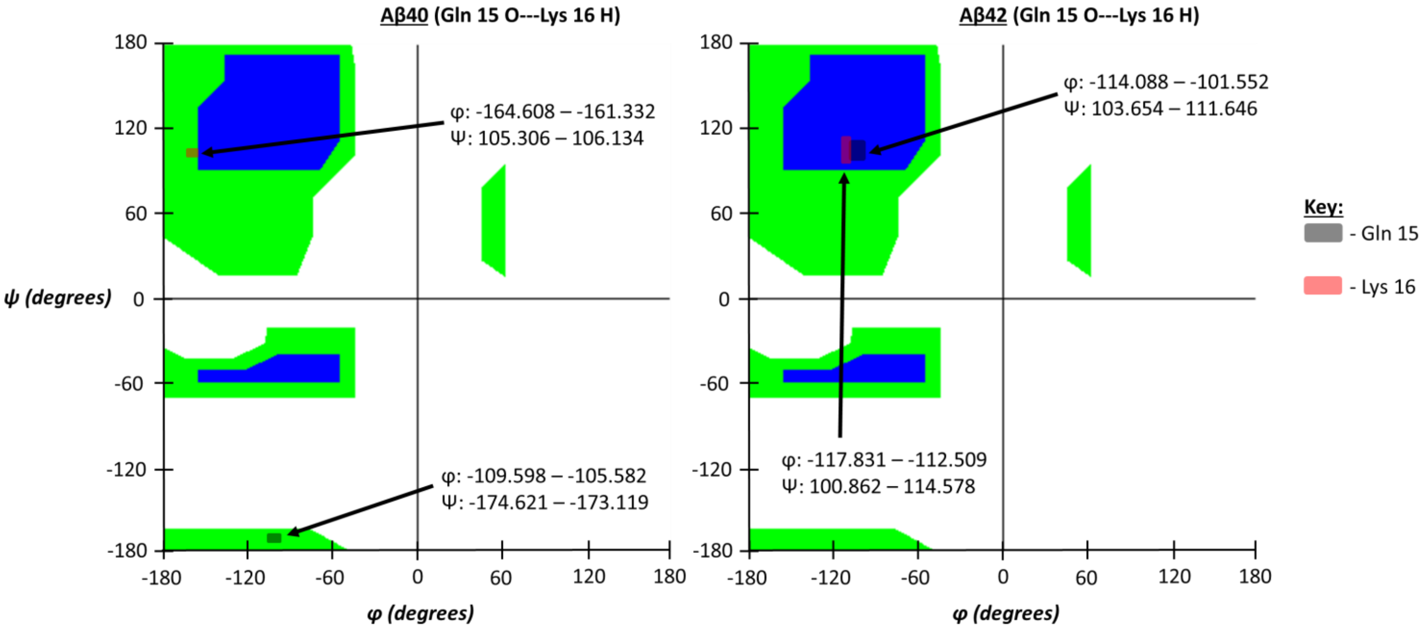

11a **11b**

Supplementary Figure 11a-11b: Ramachandran angle profiles for an exceptionally strong atom-atom interaction (Gln 15 O interacting with Lys 16 H) for Aβ40 (PDB ID: 2M4J, 11a) and Aβ42 (PDB ID: 2NAO, 11b). Ranges for φ and ψ correspond to data spread according to 95% confidence interval analysis for all ensemble members as previously described. As stated before, the first atom is from the A chain of both isoforms and the second corresponds to the partner atom on the appropriate 1:2 interaction chain configuration. Note the acquisition of β-sheet secondary structure for Gln 15 and the more well-defined β-sheet Ramachandran angle values for Lys 16 in Aβ42 compared to Aβ40.

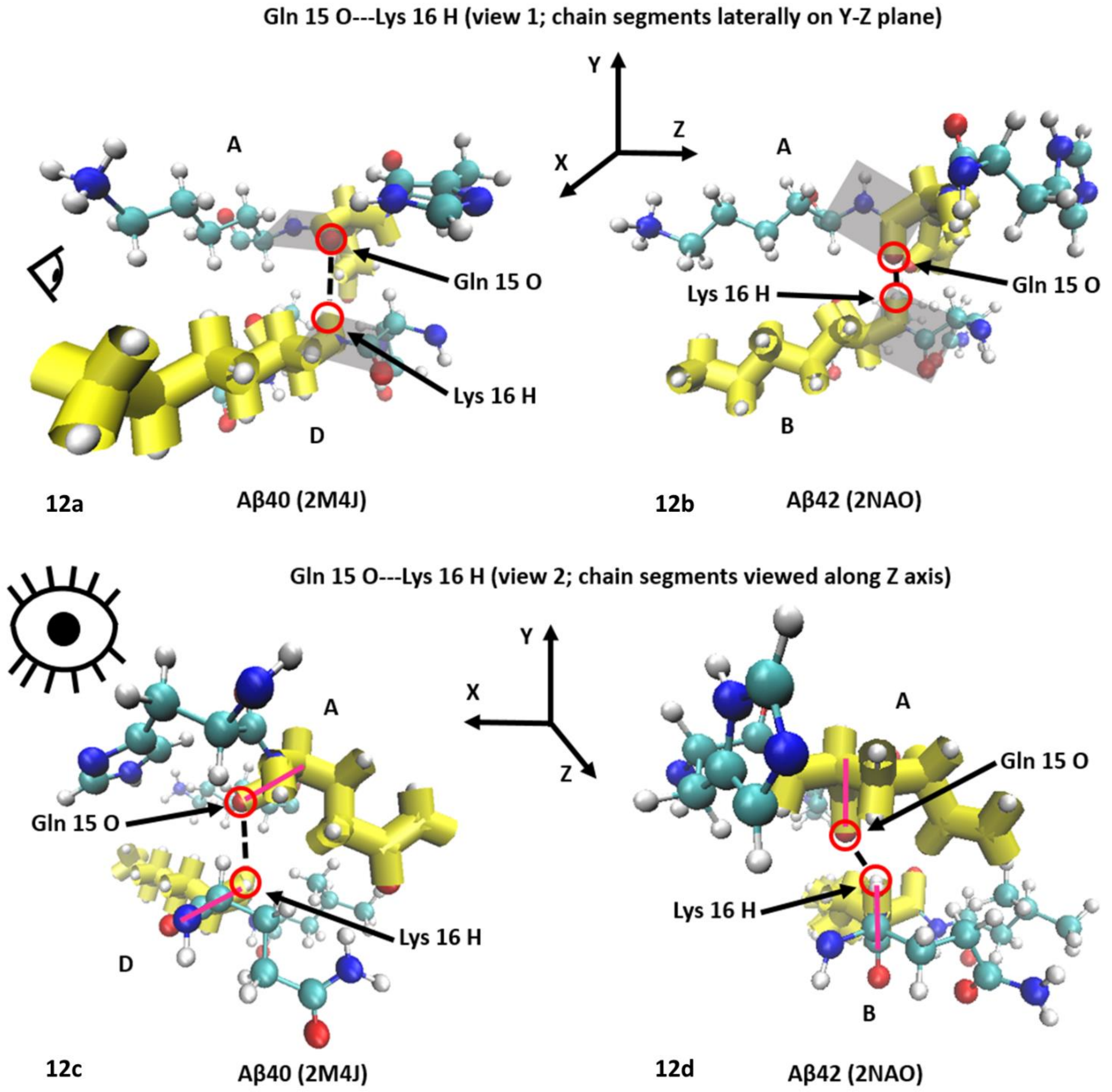

Supplementary Figure 12a-12d: Molecule representations of peptide plane alignment for Aβ40 (12a & 12c) and Aβ42 (12b & 12d). Aβ42 structure here is 2NAO. Shaded parallelograms in Figures 12a and 12b are the peptide planes for the residues whose atoms are participating in the hydrogen bonding. Figures 12c and 12d correspond to a view down the peptide bonds showing the peptide plane profile orientation in magenta. Eye icons indicate view perspective.

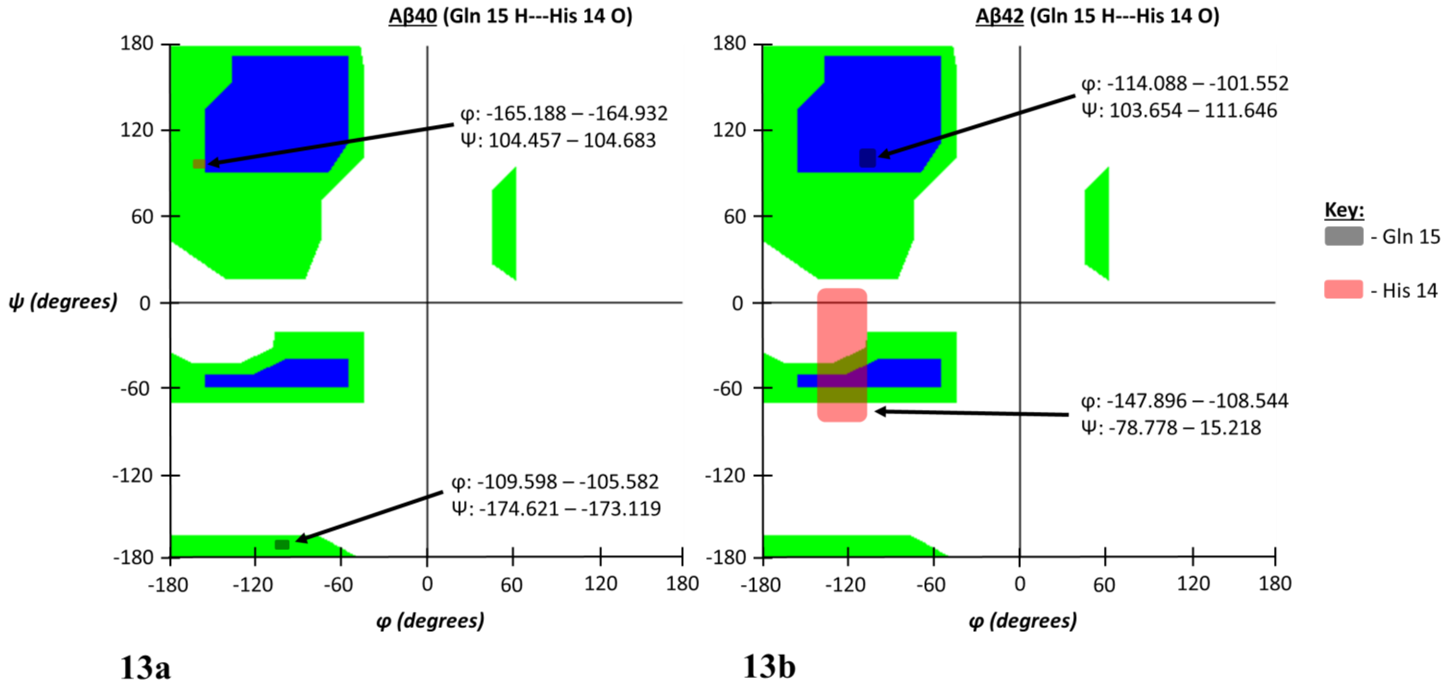

Supplementary Figure 13a-13b: Ramachandran angle profiles for an exceptionally strong atom-atom interaction (Gln 15 H interacting with His 14 O) for Aβ40 (PDB ID: 2M4J, 13a) and Aβ42 (PDB ID: 2NAO, 13b). Ranges for φ and ψ correspond to data spread according to 95% confidence interval analysis for all ensemble members as previously described. As stated before, the first atom is from the A chain of both isoforms and the second corresponds to the partner atom on the appropriate 1:2 interaction chain configuration. Note the acquisition of β-sheet secondary structure for Gln 15 and the acquisition of right-handed α-helix Ramachandran angle values for His 14 in Aβ42 compared to Aβ40.

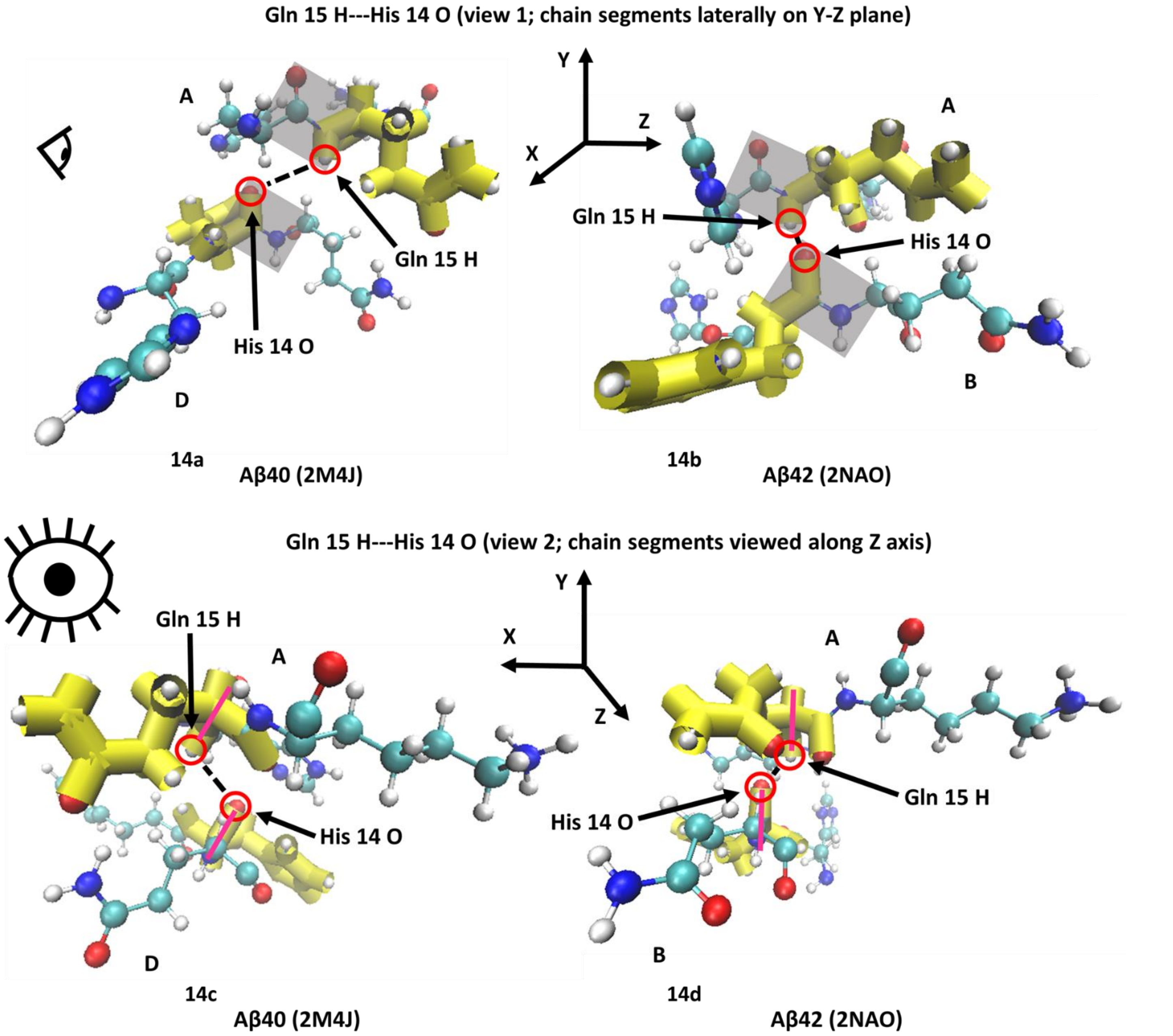

Supplementary Figure 14a-14d: Molecule representations of peptide plane alignment for Aβ40 (14a & 14c) and Aβ42 (14b & 14d). Aβ42 structure here is 2NAO. Shaded parallelograms in Figures 14a and 14b are the peptide planes for the residues whose atoms are participating in the hydrogen bonding. Figures 14c and 14d correspond to a view down the peptide bonds showing the peptide plane profile orientation in magenta. Eye icons indicate view perspective.

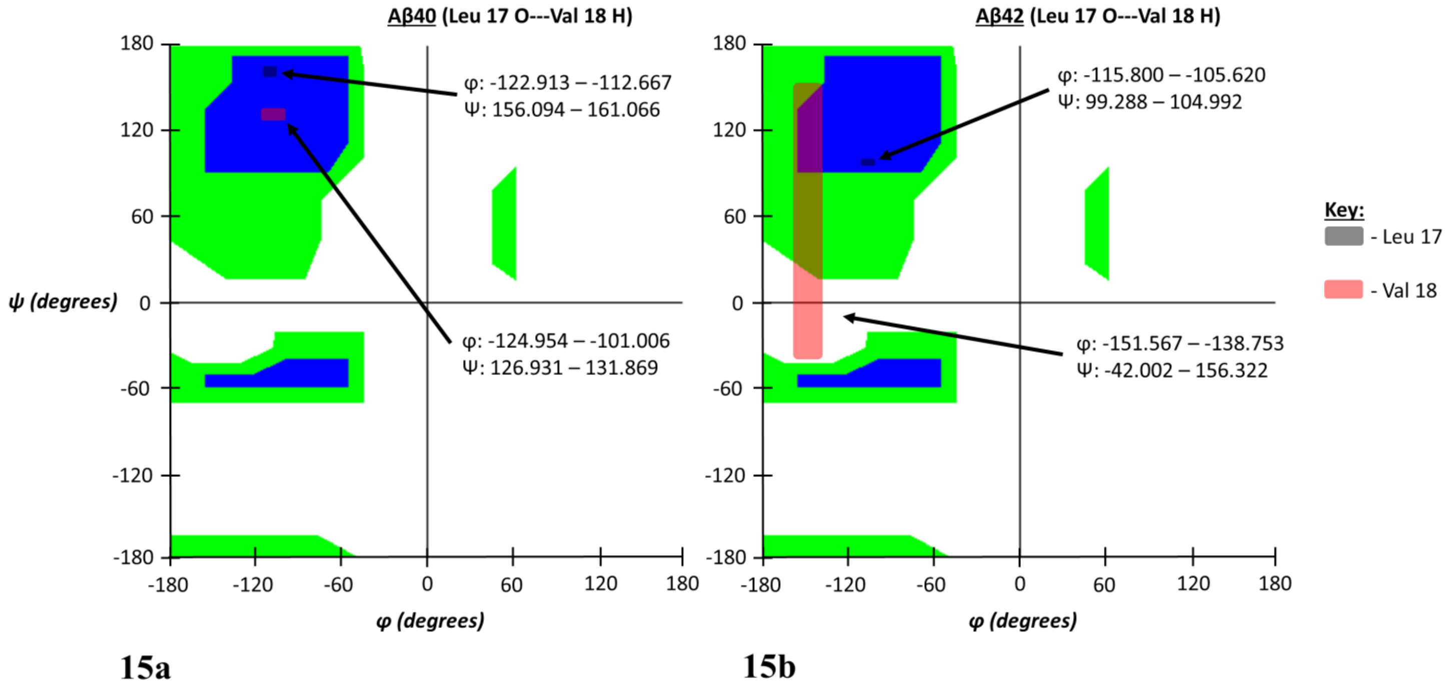

Supplementary Figure 15a-15b: Ramachandran angle profiles for an exceptionally strong atom-atom interaction (Leu 17 O interacting with Val 18 H) for Aβ40 (PDB ID: 2M4J, 15a) and Aβ42 (PDB ID: 2NAO, 15b). Ranges for φ and ψ correspond to data spread according to 95% confidence interval analysis for all ensemble members as previously described. As stated before, the first atom is from the A chain of both isoforms and the second corresponds to the partner atom on the appropriate 1:2 interaction chain configuration. Note the retention of β-sheet secondary structure for Leu 17 and the broader range of Ramachandran angle values for Val 18 in Aβ42 compared to Aβ40.

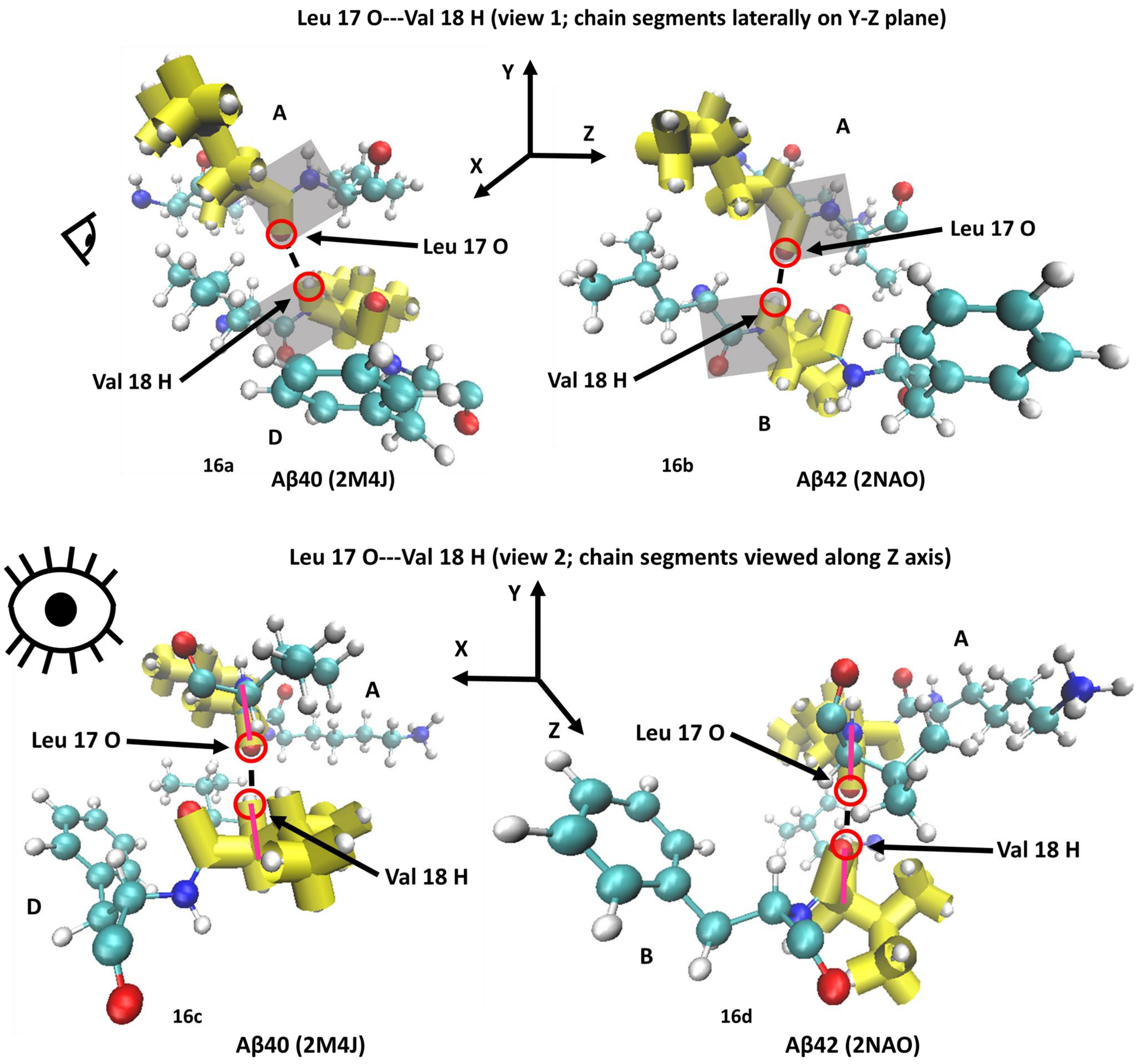

Supplementary Figure 16a-16d: Molecule representations of peptide plane alignment for Aβ40 (16a & 16c) and Aβ42 (16b & 16d). Aβ42 structure here is 2NAO. Shaded parallelograms in Figures 16a and 16b are the peptide planes for the residues whose atoms are participating in the hydrogen bonding. Figures 16c and 16d correspond to a view down the peptide bonds showing the peptide plane profile orientation in magenta. Eye icons indicate view perspective.

Supplementary Table 9a: Mapping results for Aβ42's (PDB ID: 5KK3 by Colvin et al.) short range (1:2) dominant atom-atom Coulombic interactions across ensemble structures. Columns for each chain correspond to: residue abbreviation, residue number in peptide sequence, atom identity (IUPAC naming convention) and atom number in PDB file. Energy in *kT*, distance in *nm*. Mapping analysis began on the 11th residue for both isoforms because original structure data for Aβ42 begins with the 11th residue.

Chain 1				Chain 2				Average Distance	Average Coulombic Values	Lower 95% Confidence Interval Bound	Upper 95% Confidence Interval Bound	Margin of Error
HIS	13	C	34	HIS	13	N	509	6.93E-01	-5.42E-01	-6.46E-01	-4.38E-01	1.04E-01
HIS	13	C	34	HIS	13	O	512	8.04E-01	-3.93E-01	-4.39E-01	-3.48E-01	4.54E-02
HIS	13	C	34	HIS	14	N	526	6.44E-01	-6.08E-01	-7.01E-01	-5.14E-01	9.35E-02
HIS	13	C	34	GLN	15	N	543	7.22E-01	-3.44E-01	-3.66E-01	-3.21E-01	2.22E-02
HIS	13	C	34	GLN	15	O	546	7.36E-01	-4.88E-01	-5.30E-01	-4.45E-01	4.22E-02
HIS	13	O	35	HIS	13	C	511	6.47E-01	-6.49E-01	-8.17E-01	-4.80E-01	1.69E-01
HIS	13	O	35	HIS	14	C	528	7.84E-01	-4.12E-01	-4.62E-01	-3.62E-01	5.00E-02
HIS	14	N	49	HIS	13	C	511	6.47E-01	-5.86E-01	-6.39E-01	-5.32E-01	5.35E-02
HIS	14	N	49	HIS	14	C	528	7.00E-01	-4.94E-01	-5.12E-01	-4.76E-01	1.78E-02
HIS	14	N	49	HIS	14	H	536	5.01E-01	-4.48E-01	-5.13E-01	-3.82E-01	6.55E-02
HIS	14	N	49	GLN	15	H	552	5.32E-01	-4.04E-01	-4.30E-01	-3.78E-01	2.58E-02
HIS	14	C	51	VAL	12	O	496	6.56E-01	-4.37E-01	-4.76E-01	-3.97E-01	3.98E-02
HIS	14	C	51	HIS	13	N	509	5.77E-01	-7.59E-01	-8.56E-01	-6.62E-01	9.71E-02
HIS	14	C	51	HIS	13	O	512	6.03E-01	-6.85E-01	-7.55E-01	-6.15E-01	6.98E-02
HIS	14	C	51	HIS	14	N	526	4.28E-01	-1.54E+00	-1.74E+00	-1.34E+00	2.03E-01
HIS	14	C	51	HIS	14	O	529	6.15E-01	-6.43E-01	-6.59E-01	-6.26E-01	1.67E-02
HIS	14	C	51	HIS	14	ND1	532	6.09E-01	-5.44E-01	-6.21E-01	-4.67E-01	7.70E-02
HIS	14	C	51	GLN	15	N	543	4.07E-01	-1.25E+00	-1.30E+00	-1.20E+00	4.88E-02
HIS	14	C	51	GLN	15	O	546	4.31E-01	-1.65E+00	-1.92E+00	-1.38E+00	2.70E-01
HIS	14	C	51	GLN	15	OE1	550	5.42E-01	-1.55E+00	-2.45E+00	-6.50E-01	9.01E-01
HIS	14	C	51	GLN	15	NE2	551	5.04E-01	-2.33E+00	-3.26E+00	-1.40E+00	9.29E-01
HIS	14	C	51	LYS	16	N	560	6.19E-01	-5.26E-01	-5.60E-01	-4.92E-01	3.39E-02
HIS	14	O	52	VAL	12	C	495	6.17E-01	-4.46E-01	-5.14E-01	-3.79E-01	6.74E-02
HIS	14	O	52	HIS	13	C	511	4.24E-01	-1.61E+00	-1.88E+00	-1.34E+00	2.72E-01
HIS	14	O	52	HIS	14	C	528	3.81E-01	-2.03E+00	-2.16E+00	-1.91E+00	1.24E-01
HIS	14	O	52	HIS	14	CG	531	4.82E-01	-4.90E-01	-5.68E-01	-4.12E-01	7.81E-02
HIS	14	O	52	HIS	14	H	536	2.89E-01	-2.25E+00	-3.03E+00	-1.46E+00	7.84E-01
HIS	14	O	52	GLN	15	C	545	3.99E-01	-1.19E+00	-1.41E+00	-9.82E-01	2.12E-01
HIS	14	O	52	GLN	15	CD	549	4.41E-01	-1.86E+00	-2.55E+00	-1.17E+00	6.87E-01
HIS	14	O	52	GLN	15	H	552	1.97E-01	-6.19E+00	-6.49E+00	-5.89E+00	3.02E-01
HIS	14	O	52	GLN	15	HE21	558	4.47E-01	-1.34E+00	-2.05E+00	-6.23E-01	7.14E-01
HIS	14	O	52	LYS	16	C	562	7.24E-01	-5.15E-01	-5.58E-01	-4.72E-01	4.29E-02
HIS	14	ND1	55	HIS	13	C	511	6.09E-01	-5.44E-01	-6.21E-01	-4.68E-01	7.67E-02
HIS	14	ND1	55	HIS	14	C	528	6.88E-01	-4.24E-01	-4.80E-01	-3.67E-01	5.63E-02
GLN	15	N	66	HIS	13	C	511	6.09E-01	-4.93E-01	-5.53E-01	-4.33E-01	6.01E-02
GLN	15	N	66	HIS	14	C	528	5.92E-01	-5.12E-01	-5.34E-01	-4.90E-01	2.23E-02
GLN	15	N	66	GLN	15	C	545	5.18E-01	-4.37E-01	-4.61E-01	-4.13E-01	2.43E-02
GLN	15	N	66	GLN	15	CD	549	5.25E-01	-7.57E-01	-9.30E-01	-5.83E-01	1.73E-01

GLN	15	N	66	GLN	15	H	552	3.98E-01	-6.04E-01	-6.43E-01	-5.66E-01	3.84E-02
GLN	15	C	68	GLN	15	N	543	5.58E-01	-3.71E-01	-3.97E-01	-3.45E-01	2.63E-02
GLN	15	C	68	GLN	15	O	546	3.63E-01	-1.57E+00	-1.64E+00	-1.49E+00	7.45E-02
GLN	15	C	68	GLN	15	OE1	550	6.41E-01	-4.82E-01	-5.99E-01	-3.65E-01	1.17E-01
GLN	15	C	68	GLN	15	NE2	551	6.12E-01	-7.73E-01	-9.75E-01	-5.71E-01	2.02E-01
GLN	15	C	68	LYS	16	N	560	5.74E-01	-3.89E-01	-4.02E-01	-3.75E-01	1.36E-02
GLN	15	O	69	HIS	13	C	511	7.78E-01	-4.46E-01	-4.99E-01	-3.93E-01	5.30E-02
GLN	15	O	69	HIS	14	C	528	7.86E-01	-4.28E-01	-4.47E-01	-4.08E-01	1.93E-02
GLN	15	O	69	GLN	15	C	545	5.95E-01	-4.66E-01	-4.75E-01	-4.56E-01	9.36E-03
GLN	15	O	69	GLN	15	CD	549	7.02E-01	-5.44E-01	-6.03E-01	-4.84E-01	5.93E-02
GLN	15	O	69	LYS	16	C	562	7.60E-01	-4.97E-01	-5.15E-01	-4.79E-01	1.82E-02
GLN	15	CD	72	GLN	15	O	546	6.44E-01	-6.37E-01	-6.85E-01	-5.88E-01	4.88E-02
GLN	15	CD	72	GLN	15	OE1	550	6.39E-01	-8.07E-01	-1.03E+00	-5.86E-01	2.21E-01
GLN	15	CD	72	GLN	15	NE2	551	5.87E-01	-1.48E+00	-1.95E+00	-1.01E+00	4.66E-01
GLN	15	OE1	73	HIS	14	C	528	9.22E-01	-3.66E-01	-3.94E-01	-3.38E-01	2.85E-02
GLN	15	OE1	73	GLN	15	CD	549	6.66E-01	-7.14E-01	-8.69E-01	-5.59E-01	1.55E-01
GLN	15	NE2	74	HIS	14	C	528	8.91E-01	-5.51E-01	-6.05E-01	-4.97E-01	5.39E-02
GLN	15	NE2	74	GLN	15	C	545	7.68E-01	-4.51E-01	-5.02E-01	-4.00E-01	5.11E-02
GLN	15	NE2	74	GLN	15	CD	549	6.49E-01	-1.04E+00	-1.26E+00	-8.30E-01	2.13E-01
GLN	15	NE2	74	GLN	15	HE21	558	6.13E-01	-9.28E-01	-1.33E+00	-5.29E-01	3.99E-01
GLN	15	NE2	74	GLN	15	HE22	559	6.18E-01	-1.11E+00	-1.72E+00	-5.00E-01	6.10E-01
GLN	15	H	75	GLN	15	O	546	5.22E-01	-4.54E-01	-4.92E-01	-4.17E-01	3.75E-02
GLN	15	HA	76	GLN	15	O	546	2.63E-01	-1.49E+00	-1.74E+00	-1.23E+00	2.57E-01
GLN	15	HE21	81	GLN	15	NE2	551	5.92E-01	-1.18E+00	-1.82E+00	-5.53E-01	6.32E-01
GLN	15	HE22	82	GLN	15	NE2	551	6.84E-01	-6.49E-01	-8.61E-01	-4.37E-01	2.12E-01
LYS	16	N	83	HIS	13	C	511	6.86E-01	-4.35E-01	-4.86E-01	-3.85E-01	5.05E-02
LYS	16	N	83	HIS	14	C	528	6.58E-01	-4.63E-01	-4.88E-01	-4.38E-01	2.53E-02
LYS	16	N	83	GLN	15	C	545	4.06E-01	-8.99E-01	-9.37E-01	-8.60E-01	3.86E-02
LYS	16	N	83	GLN	15	CD	549	6.16E-01	-5.55E-01	-6.45E-01	-4.65E-01	9.01E-02
LYS	16	N	83	GLN	15	H	552	4.98E-01	-3.97E-01	-4.45E-01	-3.48E-01	4.87E-02
LYS	16	N	83	LYS	16	C	562	5.39E-01	-7.81E-01	-8.32E-01	-7.30E-01	5.09E-02
LYS	16	N	83	LEU	17	C	584	6.67E-01	-3.93E-01	-4.24E-01	-3.62E-01	3.10E-02
LYS	16	N	83	LEU	17	H	590	4.24E-01	-5.17E-01	-5.95E-01	-4.40E-01	7.74E-02
LYS	16	C	85	HIS	14	N	526	8.62E-01	-3.73E-01	-3.90E-01	-3.56E-01	1.71E-02
LYS	16	C	85	HIS	14	O	529	8.82E-01	-3.60E-01	-3.76E-01	-3.44E-01	1.58E-02
LYS	16	C	85	GLN	15	N	543	7.19E-01	-3.79E-01	-4.05E-01	-3.53E-01	2.64E-02
LYS	16	C	85	GLN	15	O	546	4.24E-01	-1.84E+00	-2.11E+00	-1.57E+00	2.68E-01
LYS	16	C	85	GLN	15	OE1	550	8.09E-01	-5.45E-01	-6.97E-01	-3.93E-01	1.52E-01
LYS	16	C	85	GLN	15	NE2	551	8.03E-01	-7.82E-01	-1.01E+00	-5.59E-01	2.24E-01
LYS	16	C	85	LYS	16	N	560	5.47E-01	-7.53E-01	-8.02E-01	-7.03E-01	4.96E-02
LYS	16	C	85	LYS	16	O	563	6.00E-01	-7.91E-01	-8.17E-01	-7.64E-01	2.63E-02
LYS	16	C	85	LEU	17	N	582	3.96E-01	-1.35E+00	-1.42E+00	-1.28E+00	7.06E-02
LYS	16	C	85	LEU	17	O	585	4.21E-01	-1.91E+00	-2.25E+00	-1.57E+00	3.39E-01
LYS	16	C	85	VAL	18	N	601	5.94E-01	-6.52E-01	-7.02E-01	-6.01E-01	5.09E-02
LYS	16	O	86	HIS	14	C	528	7.89E-01	-4.25E-01	-4.55E-01	-3.95E-01	2.98E-02
LYS	16	O	86	GLN	15	C	545	4.63E-01	-8.69E-01	-1.03E+00	-7.08E-01	1.61E-01

LYS	16	O	86	GLN	15	CD	549	7.52E-01	-5.22E-01	-6.79E-01	-3.65E-01	1.57E-01
LYS	16	O	86	LYS	16	C	562	3.74E-01	-2.51E+00	-2.70E+00	-2.31E+00	1.95E-01
LYS	16	O	86	LYS	16	HA	570	3.05E-01	-8.60E-01	-1.08E+00	-6.44E-01	2.16E-01
LYS	16	O	86	LEU	17	C	584	3.78E-01	-2.06E+00	-2.49E+00	-1.63E+00	4.26E-01
LYS	16	O	86	LEU	17	H	590	1.98E-01	-5.64E+00	-5.88E+00	-5.39E+00	2.44E-01
LYS	16	O	86	VAL	18	CB	605	6.30E-01	-3.99E-01	-4.46E-01	-3.52E-01	4.72E-02
LYS	16	O	86	VAL	18	H	608	5.41E-01	-6.12E-01	-6.76E-01	-5.48E-01	6.40E-02
LYS	16	H	92	GLN	15	O	546	2.00E-01	-5.21E+00	-5.22E+00	-5.19E+00	1.78E-02
LYS	16	H	92	GLN	15	NE2	551	5.85E-01	-5.18E-01	-6.81E-01	-3.54E-01	1.63E-01
LYS	16	H	92	LYS	16	N	560	3.93E-01	-5.86E-01	-6.27E-01	-5.45E-01	4.05E-02
LEU	17	N	105	LYS	16	C	562	5.86E-01	-5.25E-01	-5.48E-01	-5.03E-01	2.27E-02
LEU	17	N	105	LEU	17	C	584	5.11E-01	-5.66E-01	-5.98E-01	-5.35E-01	3.15E-02
LEU	17	N	105	LEU	17	H	590	3.98E-01	-4.82E-01	-5.16E-01	-4.48E-01	3.40E-02
LEU	17	C	107	GLN	15	O	546	7.54E-01	-3.99E-01	-4.19E-01	-3.78E-01	2.05E-02
LEU	17	C	107	LYS	16	O	563	7.84E-01	-3.70E-01	-3.83E-01	-3.56E-01	1.37E-02
LEU	17	C	107	LEU	17	N	582	5.65E-01	-4.51E-01	-4.82E-01	-4.20E-01	3.14E-02
LEU	17	C	107	LEU	17	O	585	3.65E-01	-2.09E+00	-2.21E+00	-1.97E+00	1.19E-01
LEU	17	C	107	VAL	18	N	601	5.79E-01	-5.39E-01	-5.54E-01	-5.24E-01	1.46E-02
LEU	17	O	108	LYS	16	C	562	8.03E-01	-4.45E-01	-4.65E-01	-4.25E-01	2.03E-02
LEU	17	O	108	LEU	17	C	584	6.01E-01	-6.17E-01	-6.30E-01	-6.04E-01	1.32E-02
LEU	17	HA	114	LEU	17	O	585	2.65E-01	-1.27E+00	-1.47E+00	-1.08E+00	1.95E-01
VAL	18	N	124	LYS	16	C	562	6.68E-01	-5.08E-01	-5.40E-01	-4.76E-01	3.18E-02
VAL	18	N	124	LEU	17	C	584	4.06E-01	-1.27E+00	-1.32E+00	-1.21E+00	5.61E-02
VAL	18	N	124	LEU	17	H	590	4.89E-01	-3.69E-01	-4.09E-01	-3.28E-01	4.06E-02
VAL	18	N	124	VAL	18	C	603	5.67E-01	-4.42E-01	-4.59E-01	-4.24E-01	1.73E-02
VAL	18	N	124	VAL	18	CB	605	5.53E-01	-4.16E-01	-4.50E-01	-3.81E-01	3.40E-02
VAL	18	N	124	VAL	18	H	608	5.79E-01	-4.13E-01	-4.21E-01	-4.05E-01	8.27E-03
VAL	18	C	126	LEU	17	O	585	4.00E-01	-1.30E+00	-1.47E+00	-1.13E+00	1.67E-01
VAL	18	C	126	VAL	18	N	601	5.28E-01	-5.19E-01	-5.46E-01	-4.91E-01	2.78E-02
VAL	18	C	126	VAL	18	O	604	6.10E-01	-3.37E-01	-3.41E-01	-3.32E-01	4.34E-03
VAL	18	C	126	PHE	19	N	617	4.28E-01	-7.11E-01	-7.53E-01	-6.68E-01	4.23E-02
VAL	18	O	127	LYS	16	C	562	7.67E-01	-3.51E-01	-3.72E-01	-3.31E-01	2.05E-02
VAL	18	O	127	LEU	17	C	584	4.25E-01	-1.03E+00	-1.16E+00	-8.97E-01	1.32E-01
VAL	18	O	127	VAL	18	C	603	3.89E-01	-9.94E-01	-1.04E+00	-9.48E-01	4.55E-02
VAL	18	O	127	VAL	18	CB	605	4.64E-01	-5.83E-01	-6.74E-01	-4.92E-01	9.11E-02
VAL	18	O	127	VAL	18	H	608	5.31E-01	-4.55E-01	-4.83E-01	-4.27E-01	2.81E-02
VAL	18	O	127	PHE	19	C	619	5.08E-01	-6.36E-01	-6.99E-01	-5.73E-01	6.28E-02
VAL	18	O	127	PHE	19	H	628	2.49E-01	-2.01E+00	-2.55E+00	-1.46E+00	5.50E-01
VAL	18	CB	128	LEU	17	O	585	4.26E-01	-9.76E-01	-1.10E+00	-8.55E-01	1.21E-01
VAL	18	CB	128	VAL	18	N	601	5.54E-01	-4.17E-01	-4.62E-01	-3.71E-01	4.51E-02
VAL	18	H	131	LYS	16	O	563	6.65E-01	-3.90E-01	-4.13E-01	-3.66E-01	2.32E-02
VAL	18	H	131	LEU	17	N	582	4.60E-01	-5.68E-01	-6.30E-01	-5.05E-01	6.21E-02
VAL	18	H	131	LEU	17	O	585	1.94E-01	-1.00E+01	-1.09E+01	-9.16E+00	8.87E-01
VAL	18	H	131	VAL	18	N	601	3.93E-01	-1.06E+00	-1.09E+00	-1.02E+00	3.34E-02
VAL	18	H	131	VAL	18	O	604	5.91E-01	-3.57E-01	-3.68E-01	-3.45E-01	1.19E-02

VAL	18	H	131	PHE	19	N	617	4.95E-01	-4.94E-01	-5.36E-01	-4.52E-01	4.24E-02
PHE	19	N	140	LEU	17	C	584	6.21E-01	-3.85E-01	-4.16E-01	-3.55E-01	3.04E-02
PHE	19	N	140	VAL	18	C	603	5.74E-01	-3.54E-01	-3.73E-01	-3.35E-01	1.92E-02
PHE	19	N	140	PHE	19	C	619	6.03E-01	-3.88E-01	-4.01E-01	-3.76E-01	1.23E-02
PHE	19	N	140	PHE	19	H	628	3.97E-01	-4.55E-01	-4.87E-01	-4.23E-01	3.19E-02
PHE	19	C	142	LEU	17	O	585	6.87E-01	-4.52E-01	-4.82E-01	-4.21E-01	3.07E-02
PHE	19	C	142	PHE	19	N	617	4.73E-01	-6.91E-01	-7.63E-01	-6.19E-01	7.17E-02
PHE	19	C	142	PHE	19	O	620	5.58E-01	-7.15E-01	-9.76E-01	-4.54E-01	2.61E-01
PHE	19	C	142	PHE	20	N	637	4.31E-01	-9.14E-01	-1.06E+00	-7.69E-01	1.44E-01
PHE	19	C	142	PHE	20	O	640	4.10E-01	-1.47E+00	-1.85E+00	-1.10E+00	3.74E-01
PHE	19	C	142	ALA	21	N	657	5.70E-01	-5.00E-01	-5.74E-01	-4.27E-01	7.36E-02
PHE	19	O	143	LEU	17	C	584	7.67E-01	-3.48E-01	-3.65E-01	-3.30E-01	1.70E-02
PHE	19	O	143	VAL	18	C	603	5.61E-01	-5.18E-01	-5.76E-01	-4.60E-01	5.79E-02
PHE	19	O	143	PHE	19	C	619	4.09E-01	-1.42E+00	-1.64E+00	-1.19E+00	2.24E-01
PHE	19	O	143	PHE	19	H	628	3.74E-01	-7.72E-01	-9.27E-01	-6.16E-01	1.56E-01
PHE	19	O	143	PHE	20	C	639	4.09E-01	-1.57E+00	-2.02E+00	-1.12E+00	4.49E-01
PHE	19	O	143	ALA	21	C	659	6.56E-01	-4.84E-01	-5.54E-01	-4.15E-01	6.94E-02
PHE	20	N	160	PHE	19	C	619	5.60E-01	-5.01E-01	-6.39E-01	-3.63E-01	1.38E-01
PHE	20	N	160	PHE	20	C	639	4.82E-01	-6.68E-01	-7.54E-01	-5.81E-01	8.66E-02
PHE	20	C	162	PHE	20	N	637	6.10E-01	-3.82E-01	-4.10E-01	-3.55E-01	2.76E-02
PHE	20	C	162	PHE	20	O	640	4.07E-01	-1.36E+00	-1.45E+00	-1.27E+00	9.42E-02
PHE	20	C	162	ALA	21	N	657	5.50E-01	-5.27E-01	-5.72E-01	-4.81E-01	4.50E-02
PHE	20	C	162	ALA	21	O	660	5.40E-01	-9.29E-01	-1.25E+00	-6.04E-01	3.25E-01
PHE	20	C	162	GLU	22	O	670	7.35E-01	-4.41E-01	-5.12E-01	-3.71E-01	7.06E-02
PHE	20	O	163	PHE	20	C	639	6.01E-01	-5.37E-01	-5.60E-01	-5.14E-01	2.29E-02
PHE	20	O	163	ALA	21	C	659	6.69E-01	-4.63E-01	-5.26E-01	-4.00E-01	6.32E-02
PHE	20	HA	172	PHE	20	O	640	2.70E-01	-8.55E-01	-1.02E+00	-6.92E-01	1.63E-01
ALA	21	N	180	PHE	20	C	639	4.71E-01	-7.62E-01	-8.38E-01	-6.86E-01	7.64E-02
ALA	21	N	180	ALA	21	C	659	4.63E-01	-8.71E-01	-1.05E+00	-6.94E-01	1.77E-01
ALA	21	C	182	PHE	20	O	640	5.83E-01	-6.28E-01	-7.29E-01	-5.28E-01	1.00E-01
ALA	21	C	182	ALA	21	N	657	6.34E-01	-4.04E-01	-4.43E-01	-3.66E-01	3.84E-02
ALA	21	C	182	ALA	21	O	660	4.38E-01	-1.44E+00	-1.73E+00	-1.14E+00	2.99E-01
ALA	21	C	182	GLU	22	N	667	5.76E-01	-5.20E-01	-5.82E-01	-4.59E-01	6.18E-02
ALA	21	C	182	GLU	22	O	670	6.53E-01	-6.02E-01	-7.24E-01	-4.80E-01	1.22E-01
ALA	21	C	182	GLU	22	OE1	674	8.03E-01	-5.46E-01	-6.47E-01	-4.45E-01	1.01E-01
ALA	21	C	182	GLU	22	OE2	675	8.33E-01	-5.23E-01	-6.29E-01	-4.17E-01	1.06E-01
ALA	21	C	182	ASP	23	N	682	7.05E-01	-4.55E-01	-5.04E-01	-4.05E-01	4.96E-02
ALA	21	O	183	ALA	21	C	659	5.77E-01	-7.09E-01	-8.50E-01	-5.68E-01	1.41E-01
ALA	21	O	183	GLU	22	CD	673	8.37E-01	-4.43E-01	-4.86E-01	-4.00E-01	4.31E-02
ALA	21	H	185	PHE	20	O	640	3.40E-01	-1.48E+00	-2.04E+00	-9.25E-01	5.57E-01
ALA	21	H	185	ALA	21	N	657	4.20E-01	-5.43E-01	-5.92E-01	-4.94E-01	4.88E-02
ALA	21	H	185	ALA	21	O	660	3.57E-01	-2.22E+00	-3.72E+00	-7.28E-01	1.49E+00
GLU	22	N	190	PHE	20	C	639	6.34E-01	-4.14E-01	-4.70E-01	-3.58E-01	5.61E-02
GLU	22	N	190	ALA	21	C	659	4.59E-01	-9.01E-01	-1.03E+00	-7.76E-01	1.25E-01
GLU	22	N	190	GLU	22	C	669	5.42E-01	-5.10E-01	-5.98E-01	-4.22E-01	8.78E-02
GLU	22	N	190	GLU	22	CD	673	7.18E-01	-4.75E-01	-5.77E-01	-3.72E-01	1.03E-01

GLU	22	C	192	ALA	21	O	660	5.30E-01	-7.40E-01	-8.85E-01	-5.94E-01	1.46E-01
GLU	22	C	192	GLU	22	N	667	5.76E-01	-4.36E-01	-4.96E-01	-3.76E-01	6.04E-02
GLU	22	C	192	GLU	22	O	670	5.21E-01	-7.91E-01	-9.26E-01	-6.57E-01	1.35E-01
GLU	22	C	192	GLU	22	OE1	674	7.54E-01	-5.44E-01	-7.08E-01	-3.81E-01	1.63E-01
GLU	22	C	192	GLU	22	OE2	675	7.62E-01	-5.58E-01	-7.57E-01	-3.60E-01	1.99E-01
GLU	22	C	192	ASP	23	N	682	5.08E-01	-7.55E-01	-8.32E-01	-6.77E-01	7.76E-02
GLU	22	C	192	ASP	23	OD1	688	7.01E-01	-5.65E-01	-7.98E-01	-3.32E-01	2.33E-01
GLU	22	C	192	ASP	23	OD2	689	6.94E-01	-5.09E-01	-5.70E-01	-4.47E-01	6.16E-02
GLU	22	O	193	PHE	20	C	639	7.34E-01	-4.26E-01	-4.61E-01	-3.90E-01	3.51E-02
GLU	22	O	193	ALA	21	C	659	5.73E-01	-8.36E-01	-1.08E+00	-5.92E-01	2.44E-01
GLU	22	O	193	GLU	22	C	669	5.02E-01	-9.13E-01	-1.17E+00	-6.57E-01	2.56E-01
GLU	22	O	193	GLU	22	CD	673	7.74E-01	-6.94E-01	-9.71E-01	-4.17E-01	2.77E-01
GLU	22	O	193	ASP	23	C	684	6.29E-01	-4.77E-01	-5.36E-01	-4.18E-01	5.94E-02
GLU	22	O	193	ASP	23	CG	687	6.87E-01	-6.64E-01	-7.37E-01	-5.90E-01	7.40E-02
GLU	22	O	193	VAL	24	H	701	5.43E-01	-6.74E-01	-7.94E-01	-5.54E-01	1.20E-01
GLU	22	CD	196	ALA	21	O	660	5.03E-01	-2.14E+00	-3.21E+00	-1.06E+00	1.07E+00
GLU	22	CD	196	GLU	22	OE1	674	6.04E-01	-1.64E+00	-2.23E+00	-1.05E+00	5.92E-01
GLU	22	CD	196	GLU	22	OE2	675	6.23E-01	-1.35E+00	-1.72E+00	-9.77E-01	3.69E-01
GLU	22	CD	196	ASP	23	N	682	6.16E-01	-1.12E+00	-1.50E+00	-7.46E-01	3.75E-01
GLU	22	OE1	197	ALA	21	C	659	6.20E-01	-1.42E+00	-2.15E+00	-7.00E-01	7.23E-01
GLU	22	OE1	197	GLU	22	CD	673	6.05E-01	-1.60E+00	-2.21E+00	-9.88E-01	6.13E-01
GLU	22	OE2	198	ALA	21	C	659	6.08E-01	-1.27E+00	-1.78E+00	-7.64E-01	5.09E-01
GLU	22	OE2	198	GLU	22	CD	673	6.41E-01	-1.52E+00	-2.17E+00	-8.70E-01	6.48E-01
GLU	22	H	199	GLU	22	O	670	5.01E-01	-6.38E-01	-8.38E-01	-4.38E-01	2.00E-01
ASP	23	N	205	ALA	21	C	659	6.62E-01	-5.11E-01	-5.65E-01	-4.57E-01	5.39E-02
ASP	23	N	205	GLU	22	C	669	5.34E-01	-6.76E-01	-7.67E-01	-5.85E-01	9.11E-02
ASP	23	N	205	GLU	22	CD	673	7.48E-01	-5.70E-01	-6.72E-01	-4.68E-01	1.02E-01
ASP	23	N	205	ASP	23	C	684	6.30E-01	-4.40E-01	-4.81E-01	-3.98E-01	4.19E-02
ASP	23	N	205	ASP	23	CG	687	6.09E-01	-8.69E-01	-1.13E+00	-6.07E-01	2.62E-01
ASP	23	N	205	ASP	23	H	690	5.07E-01	-5.45E-01	-6.66E-01	-4.24E-01	1.21E-01
ASP	23	N	205	VAL	24	H	701	5.51E-01	-5.81E-01	-6.29E-01	-5.32E-01	4.86E-02
ASP	23	C	207	GLU	22	O	670	5.27E-01	-7.83E-01	-1.05E+00	-5.18E-01	2.66E-01
ASP	23	C	207	ASP	23	N	682	4.75E-01	-8.94E-01	-1.11E+00	-6.78E-01	2.16E-01
ASP	23	C	207	ASP	23	O	685	6.21E-01	-3.99E-01	-4.06E-01	-3.92E-01	6.94E-03
ASP	23	C	207	ASP	23	OD1	688	5.84E-01	-8.12E-01	-1.15E+00	-4.70E-01	3.42E-01
ASP	23	C	207	ASP	23	OD2	689	5.77E-01	-7.70E-01	-1.00E+00	-5.38E-01	2.32E-01
ASP	23	C	207	VAL	24	N	694	4.58E-01	-7.24E-01	-7.62E-01	-6.85E-01	3.88E-02
ASP	23	C	207	VAL	24	O	697	5.21E-01	-4.86E-01	-5.37E-01	-4.35E-01	5.12E-02
ASP	23	O	208	GLU	22	C	669	4.26E-01	-1.16E+00	-1.47E+00	-8.42E-01	3.13E-01
ASP	23	O	208	GLU	22	CD	673	7.57E-01	-4.94E-01	-5.70E-01	-4.18E-01	7.59E-02
ASP	23	O	208	ASP	23	C	684	3.87E-01	-1.23E+00	-1.28E+00	-1.18E+00	4.92E-02
ASP	23	O	208	ASP	23	CG	687	4.57E-01	-1.54E+00	-2.07E+00	-1.01E+00	5.30E-01
ASP	23	O	208	ASP	23	H	690	4.03E-01	-1.52E+00	-2.53E+00	-5.17E-01	1.00E+00
ASP	23	O	208	ASP	23	HA	691	2.59E-01	-7.42E-01	-8.51E-01	-6.33E-01	1.09E-01
ASP	23	O	208	VAL	24	C	696	5.14E-01	-6.23E-01	-6.83E-01	-5.64E-01	5.92E-02
ASP	23	O	208	VAL	24	CB	698	5.16E-01	-5.50E-01	-6.17E-01	-4.82E-01	6.73E-02

ASP	23	O	208	VAL	24	H	701	2.76E-01	-3.32E+00	-3.84E+00	-2.80E+00	5.22E-01
ASP	23	CG	210	GLU	22	O	670	7.38E-01	-5.93E-01	-6.80E-01	-5.05E-01	8.74E-02
ASP	23	CG	210	ASP	23	N	682	6.29E-01	-9.18E-01	-1.28E+00	-5.58E-01	3.60E-01
ASP	23	CG	210	ASP	23	O	685	8.26E-01	-4.09E-01	-4.73E-01	-3.44E-01	6.47E-02
ASP	23	CG	210	ASP	23	OD1	688	6.05E-01	-1.32E+00	-1.79E+00	-8.52E-01	4.71E-01
ASP	23	CG	210	ASP	23	OD2	689	5.92E-01	-1.52E+00	-2.08E+00	-9.60E-01	5.60E-01
ASP	23	CG	210	VAL	24	N	694	7.17E-01	-4.82E-01	-5.71E-01	-3.92E-01	8.95E-02
ASP	23	OD1	211	GLU	22	C	669	7.39E-01	-4.68E-01	-5.53E-01	-3.84E-01	8.44E-02
ASP	23	OD1	211	ASP	23	CG	687	6.30E-01	-1.09E+00	-1.35E+00	-8.19E-01	2.68E-01
ASP	23	OD1	211	VAL	24	H	701	6.93E-01	-5.43E-01	-7.27E-01	-3.60E-01	1.84E-01
ASP	23	OD2	212	ASP	23	CG	687	6.10E-01	-1.57E+00	-2.53E+00	-6.08E-01	9.59E-01
ASP	23	OD2	212	VAL	24	H	701	6.95E-01	-4.95E-01	-5.95E-01	-3.95E-01	1.00E-01
ASP	23	H	213	ASP	23	N	682	5.22E-01	-4.94E-01	-5.67E-01	-4.21E-01	7.27E-02
VAL	24	N	217	ASP	23	C	684	5.68E-01	-4.35E-01	-4.52E-01	-4.18E-01	1.69E-02
VAL	24	N	217	ASP	23	CG	687	6.41E-01	-5.95E-01	-6.97E-01	-4.94E-01	1.01E-01
VAL	24	N	217	VAL	24	C	696	5.85E-01	-4.12E-01	-4.36E-01	-3.89E-01	2.36E-02
VAL	24	N	217	VAL	24	H	701	4.03E-01	-9.98E-01	-1.07E+00	-9.22E-01	7.50E-02
VAL	24	C	219	ASP	23	OD2	689	7.61E-01	-4.12E-01	-4.76E-01	-3.48E-01	6.37E-02
VAL	24	C	219	VAL	24	N	694	5.13E-01	-5.58E-01	-6.07E-01	-5.10E-01	4.86E-02
VAL	24	C	219	VAL	24	O	697	3.85E-01	-1.03E+00	-1.11E+00	-9.49E-01	8.31E-02
VAL	24	C	219	GLY	25	O	713	5.30E-01	-5.85E-01	-6.29E-01	-5.41E-01	4.36E-02
VAL	24	O	220	VAL	24	C	696	6.02E-01	-3.48E-01	-3.60E-01	-3.36E-01	1.20E-02
VAL	24	O	220	VAL	24	H	701	5.40E-01	-4.41E-01	-4.83E-01	-3.99E-01	4.20E-02
VAL	24	O	220	GLY	25	C	712	6.93E-01	-3.40E-01	-3.54E-01	-3.25E-01	1.45E-02
VAL	24	CB	221	VAL	24	N	694	5.06E-01	-5.17E-01	-5.74E-01	-4.60E-01	5.72E-02
VAL	24	CB	221	VAL	24	O	697	4.68E-01	-6.02E-01	-7.36E-01	-4.67E-01	1.35E-01
VAL	24	H	224	ASP	23	N	682	6.61E-01	-4.04E-01	-4.61E-01	-3.46E-01	5.75E-02
VAL	24	H	224	ASP	23	OD2	689	7.47E-01	-4.26E-01	-5.08E-01	-3.45E-01	8.16E-02
VAL	24	H	224	VAL	24	N	694	5.91E-01	-3.97E-01	-4.11E-01	-3.82E-01	1.48E-02
GLY	25	N	233	VAL	24	C	696	4.07E-01	-8.31E-01	-9.21E-01	-7.41E-01	8.96E-02
GLY	25	N	233	VAL	24	H	701	4.19E-01	-7.72E-01	-8.85E-01	-6.60E-01	1.13E-01
GLY	25	N	233	GLY	25	C	712	4.86E-01	-6.85E-01	-7.40E-01	-6.31E-01	5.45E-02
GLY	25	C	235	VAL	24	N	694	7.01E-01	-3.73E-01	-4.02E-01	-3.44E-01	2.91E-02
GLY	25	C	235	VAL	24	O	697	4.63E-01	-8.45E-01	-9.44E-01	-7.46E-01	9.88E-02
GLY	25	C	235	GLY	25	N	710	5.97E-01	-4.26E-01	-4.47E-01	-4.05E-01	2.07E-02
GLY	25	C	235	GLY	25	O	713	3.69E-01	-1.88E+00	-2.00E+00	-1.75E+00	1.26E-01
GLY	25	C	235	SER	26	N	717	5.85E-01	-6.43E-01	-6.68E-01	-6.17E-01	2.56E-02
GLY	25	C	235	SER	26	O	720	7.95E-01	-3.77E-01	-3.92E-01	-3.62E-01	1.51E-02
GLY	25	C	235	SER	26	OG	722	7.08E-01	-5.24E-01	-5.75E-01	-4.72E-01	5.15E-02
GLY	25	C	235	ASN	27	N	728	6.33E-01	-4.35E-01	-4.66E-01	-4.03E-01	3.14E-02
GLY	25	C	235	ASN	27	O	731	7.46E-01	-3.83E-01	-4.02E-01	-3.63E-01	1.94E-02
GLY	25	C	235	ASN	27	ND2	735	8.78E-01	-4.35E-01	-4.70E-01	-3.99E-01	3.51E-02
GLY	25	O	236	GLY	25	C	712	5.95E-01	-5.82E-01	-6.01E-01	-5.62E-01	1.96E-02
GLY	25	H	237	VAL	24	N	694	4.08E-01	-6.02E-01	-7.37E-01	-4.67E-01	1.35E-01
GLY	25	H	237	VAL	24	O	697	2.22E-01	-3.32E+00	-4.13E+00	-2.50E+00	8.10E-01
GLY	25	H	237	GLY	25	N	710	4.10E-01	-4.62E-01	-5.06E-01	-4.18E-01	4.39E-02

GLY	25	H	237	GLY	25	O	713	3.70E-01	-8.55E-01	-1.02E+00	-6.85E-01	1.70E-01
SER	26	N	240	VAL	24	C	696	5.21E-01	-6.45E-01	-6.89E-01	-6.01E-01	4.42E-02
SER	26	N	240	VAL	24	H	701	6.49E-01	-3.93E-01	-4.13E-01	-3.73E-01	2.02E-02
SER	26	N	240	GLY	25	C	712	3.97E-01	-1.64E+00	-1.71E+00	-1.57E+00	7.08E-02
SER	26	N	240	SER	26	C	719	5.51E-01	-6.13E-01	-6.53E-01	-5.73E-01	3.98E-02
SER	26	N	240	SER	26	H	723	5.76E-01	-3.95E-01	-4.10E-01	-3.81E-01	1.46E-02
SER	26	N	240	SER	26	HA	724	3.90E-01	-4.22E-01	-4.59E-01	-3.85E-01	3.67E-02
SER	26	N	240	SER	26	HG	727	6.17E-01	-4.59E-01	-5.28E-01	-3.91E-01	6.84E-02
SER	26	N	240	ASN	27	C	730	6.66E-01	-5.26E-01	-5.66E-01	-4.86E-01	3.99E-02
SER	26	N	240	ASN	27	CG	733	7.16E-01	-4.33E-01	-4.70E-01	-3.96E-01	3.70E-02
SER	26	N	240	ASN	27	H	736	4.14E-01	-6.62E-01	-7.60E-01	-5.65E-01	9.74E-02
SER	26	CA	241	GLY	25	O	713	3.53E-01	-4.32E-01	-4.68E-01	-3.96E-01	3.59E-02
SER	26	C	242	GLY	25	O	713	3.98E-01	-1.29E+00	-1.40E+00	-1.18E+00	1.13E-01
SER	26	C	242	SER	26	N	717	5.29E-01	-6.75E-01	-7.29E-01	-6.20E-01	5.42E-02
SER	26	C	242	SER	26	O	720	5.97E-01	-5.48E-01	-5.59E-01	-5.38E-01	1.05E-02
SER	26	C	242	SER	26	OG	722	5.98E-01	-6.11E-01	-6.66E-01	-5.56E-01	5.50E-02
SER	26	C	242	ASN	27	N	728	3.91E-01	-1.13E+00	-1.16E+00	-1.09E+00	3.88E-02
SER	26	C	242	ASN	27	O	731	4.05E-01	-1.27E+00	-1.41E+00	-1.14E+00	1.35E-01
SER	26	C	242	ASN	27	OD1	734	6.19E-01	-5.24E-01	-6.99E-01	-3.50E-01	1.75E-01
SER	26	C	242	ASN	27	ND2	735	6.24E-01	-6.99E-01	-7.96E-01	-6.03E-01	9.66E-02
SER	26	C	242	LYS	28	N	742	5.95E-01	-4.19E-01	-4.48E-01	-3.89E-01	2.96E-02
SER	26	O	243	VAL	24	C	696	6.34E-01	-4.52E-01	-4.89E-01	-4.16E-01	3.62E-02
SER	26	O	243	GLY	25	C	712	4.59E-01	-1.24E+00	-1.37E+00	-1.10E+00	1.36E-01
SER	26	O	243	SER	26	C	719	3.74E-01	-1.72E+00	-1.85E+00	-1.58E+00	1.31E-01
SER	26	O	243	SER	26	H	723	5.52E-01	-4.76E-01	-5.29E-01	-4.23E-01	5.29E-02
SER	26	O	243	SER	26	HA	724	3.03E-01	-1.01E+00	-1.29E+00	-7.44E-01	2.71E-01
SER	26	O	243	SER	26	HG	727	5.38E-01	-7.07E-01	-8.64E-01	-5.49E-01	1.57E-01
SER	26	O	243	ASN	27	C	730	3.63E-01	-2.46E+00	-2.84E+00	-2.07E+00	3.86E-01
SER	26	O	243	ASN	27	CG	733	4.88E-01	-1.10E+00	-1.28E+00	-9.16E-01	1.83E-01
SER	26	O	243	ASN	27	H	736	1.95E-01	-5.94E+00	-6.40E+00	-5.49E+00	4.55E-01
SER	26	O	243	ASN	27	HD21	740	5.36E-01	-5.49E-01	-6.53E-01	-4.45E-01	1.04E-01
SER	26	O	243	LYS	28	C	744	6.91E-01	-6.15E-01	-6.54E-01	-5.77E-01	3.86E-02
SER	26	CB	244	GLY	25	O	713	3.44E-01	-5.91E-01	-6.74E-01	-5.08E-01	8.28E-02
SER	26	OG	245	GLY	25	C	712	5.27E-01	-1.08E+00	-1.40E+00	-7.66E-01	3.16E-01
SER	26	OG	245	SER	26	C	719	5.94E-01	-6.21E-01	-6.81E-01	-5.62E-01	5.96E-02
SER	26	OG	245	SER	26	HA	724	4.07E-01	-4.66E-01	-5.54E-01	-3.79E-01	8.74E-02
SER	26	OG	245	SER	26	HG	727	4.94E-01	-1.00E+00	-1.34E+00	-6.58E-01	3.43E-01
SER	26	OG	245	ASN	27	C	730	6.93E-01	-5.99E-01	-7.03E-01	-4.95E-01	1.04E-01
SER	26	OG	245	ASN	27	CG	733	8.57E-01	-3.72E-01	-4.04E-01	-3.41E-01	3.14E-02
SER	26	OG	245	ASN	27	H	736	4.97E-01	-5.13E-01	-6.10E-01	-4.15E-01	9.76E-02
SER	26	H	246	VAL	24	O	697	3.68E-01	-9.19E-01	-1.05E+00	-7.84E-01	1.34E-01
SER	26	H	246	GLY	25	N	710	4.48E-01	-4.95E-01	-5.31E-01	-4.59E-01	3.59E-02
SER	26	H	246	GLY	25	O	713	1.99E-01	-6.59E+00	-6.78E+00	-6.39E+00	1.92E-01
SER	26	H	246	SER	26	N	717	4.03E-01	-9.40E-01	-1.01E+00	-8.74E-01	6.55E-02
SER	26	H	246	SER	26	O	720	6.01E-01	-3.89E-01	-4.16E-01	-3.61E-01	2.75E-02
SER	26	H	246	SER	26	OG	722	5.59E-01	-5.16E-01	-5.87E-01	-4.44E-01	7.19E-02

SER	26	H	246	ASN	27	N	728	4.47E-01	-5.87E-01	-6.71E-01	-5.02E-01	8.47E-02
SER	26	HG	250	GLY	25	O	713	4.65E-01	-9.28E-01	-1.24E+00	-6.14E-01	3.14E-01
SER	26	HG	250	SER	26	N	717	5.90E-01	-5.31E-01	-6.62E-01	-4.00E-01	1.31E-01
SER	26	HG	250	SER	26	OG	722	5.11E-01	-9.42E-01	-1.24E+00	-6.46E-01	2.97E-01
ASN	27	N	251	GLY	25	C	712	6.02E-01	-4.83E-01	-5.12E-01	-4.54E-01	2.88E-02
ASN	27	N	251	SER	26	C	719	5.82E-01	-4.30E-01	-4.51E-01	-4.09E-01	2.09E-02
ASN	27	N	251	ASN	27	C	730	5.17E-01	-7.22E-01	-7.65E-01	-6.78E-01	4.32E-02
ASN	27	N	251	ASN	27	CG	733	5.70E-01	-5.61E-01	-6.40E-01	-4.82E-01	7.88E-02
ASN	27	N	251	ASN	27	H	736	3.89E-01	-6.06E-01	-6.52E-01	-5.59E-01	4.65E-02
ASN	27	C	253	GLY	25	O	713	7.17E-01	-4.25E-01	-4.43E-01	-4.07E-01	1.78E-02
ASN	27	C	253	SER	26	N	717	8.13E-01	-3.59E-01	-3.75E-01	-3.43E-01	1.57E-02
ASN	27	C	253	SER	26	O	720	7.77E-01	-4.17E-01	-4.29E-01	-4.05E-01	1.19E-02
ASN	27	C	253	SER	26	OG	722	8.58E-01	-3.90E-01	-4.15E-01	-3.65E-01	2.51E-02
ASN	27	C	253	ASN	27	N	728	5.63E-01	-5.92E-01	-6.22E-01	-5.61E-01	3.05E-02
ASN	27	C	253	ASN	27	O	731	3.72E-01	-2.01E+00	-2.16E+00	-1.86E+00	1.48E-01
ASN	27	C	253	ASN	27	OD1	734	5.99E-01	-7.57E-01	-1.02E+00	-4.93E-01	2.64E-01
ASN	27	C	253	ASN	27	ND2	735	5.83E-01	-1.14E+00	-1.45E+00	-8.31E-01	3.12E-01
ASN	27	C	253	LYS	28	N	742	5.90E-01	-5.41E-01	-5.61E-01	-5.21E-01	2.00E-02
ASN	27	C	253	LYS	28	O	745	8.07E-01	-3.78E-01	-3.89E-01	-3.67E-01	1.11E-02
ASN	27	C	253	GLY	29	N	764	6.70E-01	-3.57E-01	-3.78E-01	-3.37E-01	2.04E-02
ASN	27	O	254	ASN	27	C	730	6.05E-01	-6.14E-01	-6.32E-01	-5.95E-01	1.82E-02
ASN	27	O	254	ASN	27	CG	733	6.70E-01	-4.84E-01	-5.42E-01	-4.26E-01	5.80E-02
ASN	27	O	254	LYS	28	C	744	7.92E-01	-4.27E-01	-4.38E-01	-4.15E-01	1.16E-02
ASN	27	CG	256	GLY	25	O	713	7.50E-01	-3.71E-01	-3.92E-01	-3.50E-01	2.12E-02
ASN	27	CG	256	ASN	27	N	728	6.61E-01	-4.17E-01	-4.81E-01	-3.52E-01	6.49E-02
ASN	27	CG	256	ASN	27	O	731	5.52E-01	-7.96E-01	-1.01E+00	-5.87E-01	2.09E-01
ASN	27	CG	256	ASN	27	OD1	734	6.19E-01	-7.73E-01	-1.17E+00	-3.72E-01	4.01E-01
ASN	27	CG	256	ASN	27	ND2	735	5.75E-01	-1.33E+00	-1.92E+00	-7.39E-01	5.92E-01
ASN	27	OD1	257	ASN	27	C	730	6.49E-01	-6.65E-01	-9.26E-01	-4.03E-01	2.61E-01
ASN	27	OD1	257	ASN	27	CG	733	6.00E-01	-1.01E+00	-1.60E+00	-4.22E-01	5.89E-01
ASN	27	ND2	258	GLY	25	C	712	8.89E-01	-4.29E-01	-4.73E-01	-3.84E-01	4.46E-02
ASN	27	ND2	258	SER	26	C	719	8.57E-01	-3.86E-01	-4.46E-01	-3.26E-01	5.97E-02
ASN	27	ND2	258	ASN	27	C	730	7.11E-01	-7.40E-01	-9.60E-01	-5.20E-01	2.20E-01
ASN	27	ND2	258	ASN	27	CG	733	6.27E-01	-9.74E-01	-1.31E+00	-6.37E-01	3.37E-01
LYS	28	N	265	SER	26	C	719	6.53E-01	-3.43E-01	-3.62E-01	-3.24E-01	1.88E-02
LYS	28	N	265	ASN	27	C	730	4.09E-01	-1.29E+00	-1.34E+00	-1.24E+00	4.70E-02
LYS	28	N	265	ASN	27	CG	733	5.29E-01	-6.90E-01	-8.14E-01	-5.66E-01	1.24E-01
LYS	28	N	265	LYS	28	C	744	5.78E-01	-6.65E-01	-6.92E-01	-6.37E-01	2.80E-02
LYS	28	N	265	GLY	29	C	766	7.14E-01	-3.46E-01	-3.64E-01	-3.29E-01	1.73E-02
LYS	28	C	267	SER	26	O	720	8.54E-01	-4.17E-01	-4.39E-01	-3.96E-01	2.12E-02
LYS	28	C	267	ASN	27	N	728	6.88E-01	-4.58E-01	-4.87E-01	-4.29E-01	2.90E-02
LYS	28	C	267	ASN	27	O	731	3.93E-01	-2.09E+00	-2.40E+00	-1.79E+00	3.04E-01
LYS	28	C	267	ASN	27	OD1	734	6.44E-01	-7.44E-01	-9.70E-01	-5.19E-01	2.25E-01
LYS	28	C	267	ASN	27	ND2	735	6.27E-01	-1.11E+00	-1.37E+00	-8.49E-01	2.62E-01
LYS	28	C	267	LYS	28	N	742	5.20E-01	-8.46E-01	-8.91E-01	-8.01E-01	4.47E-02
LYS	28	C	267	LYS	28	O	745	6.07E-01	-7.69E-01	-7.81E-01	-7.56E-01	1.24E-02

LYS	28	C	267	GLY	29	N	764	4.21E-01	-1.22E+00	-1.28E+00	-1.15E+00	6.66E-02
LYS	28	C	267	GLY	29	O	767	6.83E-01	-5.49E-01	-5.72E-01	-5.25E-01	2.38E-02
LYS	28	C	267	ALA	30	N	771	5.54E-01	-6.81E-01	-7.21E-01	-6.41E-01	4.03E-02
LYS	28	C	267	ALA	30	O	774	6.91E-01	-5.87E-01	-6.24E-01	-5.51E-01	3.68E-02
LYS	28	C	267	ILE	31	N	781	8.40E-01	-3.32E-01	-3.45E-01	-3.19E-01	1.28E-02
LYS	28	C	267	ALA	42	O	946	8.36E-01	-4.19E-01	-4.57E-01	-3.81E-01	3.80E-02
LYS	28	O	268	SER	26	C	719	7.44E-01	-3.43E-01	-3.59E-01	-3.27E-01	1.61E-02
LYS	28	O	268	ASN	27	C	730	4.18E-01	-1.61E+00	-1.77E+00	-1.44E+00	1.67E-01
LYS	28	O	268	ASN	27	CG	733	5.70E-01	-7.52E-01	-8.89E-01	-6.15E-01	1.37E-01
LYS	28	O	268	LYS	28	C	744	3.73E-01	-2.50E+00	-2.61E+00	-2.38E+00	1.18E-01
LYS	28	O	268	LYS	28	H	751	5.15E-01	-3.87E-01	-4.06E-01	-3.67E-01	2.00E-02
LYS	28	O	268	LYS	28	HA	752	2.56E-01	-1.30E+00	-1.42E+00	-1.18E+00	1.20E-01
LYS	28	O	268	GLY	29	C	766	4.87E-01	-1.03E+00	-1.12E+00	-9.40E-01	9.12E-02
LYS	28	O	268	GLY	29	H	768	2.23E-01	-3.99E+00	-4.67E+00	-3.30E+00	6.83E-01
LYS	28	O	268	ALA	30	C	773	6.76E-01	-4.91E-01	-5.32E-01	-4.51E-01	4.05E-02
LYS	28	O	268	ALA	30	H	776	4.23E-01	-7.73E-01	-9.24E-01	-6.22E-01	1.51E-01
LYS	28	H	274	ASN	27	N	728	4.50E-01	-4.16E-01	-4.65E-01	-3.67E-01	4.90E-02
LYS	28	H	274	ASN	27	O	731	1.99E-01	-4.89E+00	-4.97E+00	-4.81E+00	7.63E-02
LYS	28	H	274	ASN	27	ND2	735	4.81E-01	-8.12E-01	-1.14E+00	-4.85E-01	3.27E-01
LYS	28	H	274	LYS	28	N	742	4.05E-01	-5.40E-01	-5.64E-01	-5.16E-01	2.40E-02
GLY	29	N	287	ASN	27	C	730	6.05E-01	-4.43E-01	-4.80E-01	-4.06E-01	3.69E-02
GLY	29	N	287	LYS	28	C	744	5.70E-01	-5.86E-01	-6.00E-01	-5.73E-01	1.36E-02
GLY	29	N	287	GLY	29	C	766	6.00E-01	-4.21E-01	-4.36E-01	-4.05E-01	1.59E-02
GLY	29	N	287	GLY	29	H	768	3.92E-01	-5.15E-01	-5.46E-01	-4.83E-01	3.16E-02
GLY	29	N	287	ALA	30	H	776	4.71E-01	-3.72E-01	-3.98E-01	-3.47E-01	2.56E-02
GLY	29	C	289	ASN	27	O	731	6.07E-01	-5.84E-01	-6.38E-01	-5.30E-01	5.41E-02
GLY	29	C	289	ASN	27	ND2	735	6.54E-01	-7.98E-01	-9.55E-01	-6.41E-01	1.57E-01
GLY	29	C	289	LYS	28	N	742	6.78E-01	-3.84E-01	-4.09E-01	-3.59E-01	2.50E-02
GLY	29	C	289	LYS	28	O	745	6.67E-01	-5.10E-01	-5.34E-01	-4.85E-01	2.44E-02
GLY	29	C	289	GLY	29	N	764	4.57E-01	-7.95E-01	-8.54E-01	-7.37E-01	5.89E-02
GLY	29	C	289	GLY	29	O	767	5.82E-01	-6.10E-01	-6.21E-01	-6.00E-01	1.08E-02
GLY	29	C	289	ALA	30	N	771	3.80E-01	-1.37E+00	-1.43E+00	-1.31E+00	5.73E-02
GLY	29	C	289	ALA	30	O	774	3.99E-01	-1.72E+00	-1.98E+00	-1.46E+00	2.56E-01
GLY	29	C	289	ALA	30	CB	775	4.55E-01	-4.98E-01	-5.39E-01	-4.56E-01	4.20E-02
GLY	29	C	289	ILE	31	N	781	5.75E-01	-5.59E-01	-5.95E-01	-5.23E-01	3.61E-02
GLY	29	C	289	ILE	31	O	784	8.55E-01	-3.56E-01	-3.68E-01	-3.43E-01	1.26E-02
GLY	29	O	290	ASN	27	C	730	6.40E-01	-5.58E-01	-6.47E-01	-4.68E-01	8.97E-02
GLY	29	O	290	LYS	28	C	744	5.18E-01	-1.03E+00	-1.17E+00	-8.88E-01	1.41E-01
GLY	29	O	290	GLY	29	C	766	3.68E-01	-1.88E+00	-2.01E+00	-1.76E+00	1.26E-01
GLY	29	O	290	GLY	29	H	768	3.55E-01	-1.01E+00	-1.28E+00	-7.45E-01	2.65E-01
GLY	29	O	290	ALA	30	C	773	3.62E-01	-2.05E+00	-2.43E+00	-1.66E+00	3.83E-01
GLY	29	O	290	ALA	30	H	776	1.93E-01	-6.21E+00	-6.69E+00	-5.72E+00	4.85E-01
GLY	29	O	290	ILE	31	C	783	6.89E-01	-4.29E-01	-4.68E-01	-3.90E-01	3.92E-02
GLY	29	O	290	ILE	31	H	789	5.30E-01	-4.35E-01	-4.79E-01	-3.91E-01	4.39E-02
ALA	30	N	294	LYS	28	C	744	6.86E-01	-4.32E-01	-4.47E-01	-4.16E-01	1.55E-02
ALA	30	N	294	GLY	29	C	766	5.67E-01	-5.15E-01	-5.32E-01	-4.98E-01	1.70E-02

ALA	30	N	294	ALA	30	C	773	4.93E-01	-6.99E-01	-7.23E-01	-6.75E-01	2.41E-02
ALA	30	N	294	ALA	30	H	776	3.83E-01	-6.87E-01	-7.41E-01	-6.33E-01	5.37E-02
ALA	30	C	296	ASN	27	ND2	735	8.17E-01	-4.93E-01	-5.55E-01	-4.31E-01	6.22E-02
ALA	30	C	296	GLY	29	O	767	7.81E-01	-3.34E-01	-3.43E-01	-3.25E-01	8.70E-03
ALA	30	C	296	ALA	30	N	771	5.65E-01	-5.11E-01	-5.36E-01	-4.87E-01	2.46E-02
ALA	30	C	296	ALA	30	O	774	3.56E-01	-2.20E+00	-2.25E+00	-2.15E+00	5.11E-02
ALA	30	C	296	ILE	31	N	781	5.68E-01	-5.59E-01	-5.68E-01	-5.49E-01	9.42E-03
ALA	30	C	296	ILE	31	O	784	7.79E-01	-4.09E-01	-4.16E-01	-4.02E-01	6.73E-03
ALA	30	C	296	ILE	32	N	800	6.57E-01	-4.11E-01	-4.17E-01	-4.04E-01	6.28E-03
ALA	30	C	296	ILE	32	O	803	7.83E-01	-4.05E-01	-4.14E-01	-3.96E-01	8.98E-03
ALA	30	O	297	GLY	29	C	766	7.97E-01	-3.59E-01	-3.70E-01	-3.47E-01	1.12E-02
ALA	30	O	297	ALA	30	C	773	5.96E-01	-6.21E-01	-6.30E-01	-6.12E-01	9.00E-03
ALA	30	O	297	ILE	31	C	783	7.75E-01	-3.68E-01	-3.73E-01	-3.64E-01	4.58E-03
ALA	30	CB	298	ALA	30	C	773	5.23E-01	-3.45E-01	-3.64E-01	-3.26E-01	1.91E-02
ALA	30	H	299	ALA	30	O	774	4.96E-01	-4.85E-01	-5.04E-01	-4.66E-01	1.90E-02
ALA	30	HA	300	ALA	30	O	774	2.43E-01	-1.38E+00	-1.42E+00	-1.34E+00	3.92E-02
ILE	31	N	304	GLY	29	C	766	6.73E-01	-4.00E-01	-4.16E-01	-3.84E-01	1.61E-02
ILE	31	N	304	ALA	30	C	773	4.07E-01	-1.25E+00	-1.29E+00	-1.20E+00	4.21E-02
ILE	31	N	304	ALA	30	H	776	4.96E-01	-3.99E-01	-4.30E-01	-3.68E-01	3.07E-02
ILE	31	N	304	ILE	31	C	783	5.52E-01	-5.94E-01	-6.06E-01	-5.83E-01	1.14E-02
ILE	31	N	304	ILE	31	H	789	5.75E-01	-3.14E-01	-3.19E-01	-3.08E-01	5.34E-03
ILE	31	N	304	ILE	31	HA	790	3.56E-01	-5.44E-01	-5.66E-01	-5.22E-01	2.20E-02
ILE	31	N	304	ILE	32	C	802	7.09E-01	-3.54E-01	-3.59E-01	-3.49E-01	5.03E-03
ILE	31	N	304	ILE	32	H	808	4.56E-01	-5.42E-01	-5.50E-01	-5.33E-01	8.55E-03
ILE	31	C	306	ALA	30	O	774	4.15E-01	-1.47E+00	-1.58E+00	-1.36E+00	1.08E-01
ILE	31	C	306	ILE	31	N	781	5.30E-01	-6.52E-01	-6.69E-01	-6.35E-01	1.69E-02
ILE	31	C	306	ILE	31	O	784	6.07E-01	-6.65E-01	-6.75E-01	-6.55E-01	1.02E-02
ILE	31	C	306	ILE	32	N	800	4.14E-01	-1.19E+00	-1.21E+00	-1.17E+00	2.08E-02
ILE	31	C	306	ILE	32	O	803	4.43E-01	-1.38E+00	-1.45E+00	-1.32E+00	6.43E-02
ILE	31	C	306	GLY	33	N	819	6.42E-01	-3.57E-01	-3.63E-01	-3.51E-01	6.17E-03
ILE	31	O	307	GLY	29	C	766	7.75E-01	-4.21E-01	-4.36E-01	-4.06E-01	1.48E-02
ILE	31	O	307	ALA	30	C	773	4.32E-01	-1.48E+00	-1.56E+00	-1.39E+00	8.45E-02
ILE	31	O	307	ILE	31	C	783	3.65E-01	-2.30E+00	-2.36E+00	-2.23E+00	6.40E-02
ILE	31	O	307	ILE	31	H	789	5.22E-01	-5.38E-01	-5.52E-01	-5.24E-01	1.38E-02
ILE	31	O	307	ILE	31	HA	790	2.47E-01	-2.12E+00	-2.21E+00	-2.03E+00	9.39E-02
ILE	31	O	307	ILE	32	C	802	4.27E-01	-1.52E+00	-1.59E+00	-1.45E+00	6.51E-02
ILE	31	O	307	ILE	32	H	808	2.00E-01	-7.47E+00	-7.58E+00	-7.36E+00	1.13E-01
ILE	31	O	307	ILE	32	HA	809	4.89E-01	-3.31E-01	-3.38E-01	-3.24E-01	6.52E-03
ILE	31	O	307	GLY	33	C	821	7.74E-01	-4.21E-01	-4.31E-01	-4.11E-01	9.57E-03
ILE	31	H	312	ALA	30	N	771	4.70E-01	-4.54E-01	-4.85E-01	-4.23E-01	3.13E-02
ILE	31	H	312	ALA	30	O	774	1.94E-01	-7.42E+00	-8.06E+00	-6.78E+00	6.40E-01
ILE	31	H	312	ILE	31	N	781	3.86E-01	-8.32E-01	-8.55E-01	-8.09E-01	2.26E-02
ILE	31	H	312	ILE	31	O	784	5.82E-01	-4.21E-01	-4.31E-01	-4.11E-01	1.02E-02
ILE	31	H	312	ILE	32	N	800	4.69E-01	-5.06E-01	-5.16E-01	-4.95E-01	1.01E-02
ILE	31	H	312	ILE	32	O	803	6.31E-01	-3.55E-01	-3.62E-01	-3.47E-01	7.74E-03
ILE	32	N	323	ALA	30	C	773	6.38E-01	-4.38E-01	-4.51E-01	-4.24E-01	1.38E-02

ILE	32	N	323	ILE	31	C	783	5.77E-01	-5.39E-01	-5.46E-01	-5.31E-01	7.33E-03
ILE	32	N	323	ILE	32	C	802	5.29E-01	-6.56E-01	-6.66E-01	-6.45E-01	1.10E-02
ILE	32	N	323	ILE	32	H	808	3.90E-01	-8.07E-01	-8.24E-01	-7.90E-01	1.66E-02
ILE	32	C	325	ALA	30	O	774	7.51E-01	-3.91E-01	-4.04E-01	-3.78E-01	1.28E-02
ILE	32	C	325	ILE	31	O	784	7.76E-01	-4.11E-01	-4.17E-01	-4.05E-01	5.98E-03
ILE	32	C	325	ILE	32	N	800	5.55E-01	-5.88E-01	-6.00E-01	-5.77E-01	1.16E-02
ILE	32	C	325	ILE	32	O	803	3.61E-01	-2.36E+00	-2.42E+00	-2.30E+00	6.08E-02
ILE	32	C	325	GLY	33	N	819	5.74E-01	-4.52E-01	-4.59E-01	-4.45E-01	6.87E-03
ILE	32	C	325	GLY	33	O	822	7.98E-01	-3.21E-01	-3.25E-01	-3.16E-01	4.11E-03
ILE	32	O	326	ILE	31	C	783	7.86E-01	-4.01E-01	-4.07E-01	-3.95E-01	5.64E-03
ILE	32	O	326	ILE	32	C	802	6.05E-01	-6.70E-01	-6.79E-01	-6.61E-01	8.55E-03
ILE	32	O	326	ILE	32	H	808	5.91E-01	-4.07E-01	-4.14E-01	-4.00E-01	7.27E-03
ILE	32	O	326	GLY	33	C	821	8.14E-01	-3.85E-01	-3.90E-01	-3.80E-01	4.95E-03
ILE	32	H	331	ALA	30	O	774	6.00E-01	-3.54E-01	-3.69E-01	-3.38E-01	1.55E-02
ILE	32	H	331	ILE	32	O	803	5.19E-01	-5.46E-01	-5.58E-01	-5.33E-01	1.21E-02
ILE	32	HA	332	ILE	32	N	800	3.56E-01	-5.44E-01	-5.63E-01	-5.25E-01	1.88E-02
ILE	32	HA	332	ILE	32	O	803	2.45E-01	-2.17E+00	-2.19E+00	-2.16E+00	1.57E-02
GLY	33	N	342	ILE	31	C	783	6.52E-01	-3.46E-01	-3.55E-01	-3.37E-01	9.21E-03
GLY	33	N	342	ILE	32	C	802	4.13E-01	-9.95E-01	-1.02E+00	-9.71E-01	2.42E-02
GLY	33	N	342	ILE	32	H	808	4.71E-01	-4.14E-01	-4.26E-01	-4.02E-01	1.20E-02
GLY	33	N	342	GLY	33	C	821	5.92E-01	-4.33E-01	-4.41E-01	-4.24E-01	8.50E-03
GLY	33	C	344	ILE	31	O	784	8.59E-01	-3.52E-01	-3.62E-01	-3.43E-01	9.53E-03
GLY	33	C	344	ILE	32	N	800	6.77E-01	-3.95E-01	-4.04E-01	-3.86E-01	8.86E-03
GLY	33	C	344	ILE	32	O	803	4.00E-01	-1.84E+00	-1.92E+00	-1.76E+00	8.07E-02
GLY	33	C	344	GLY	33	N	819	4.91E-01	-6.61E-01	-6.77E-01	-6.44E-01	1.67E-02
GLY	33	C	344	GLY	33	CA	820	3.92E-01	-4.02E-01	-4.16E-01	-3.89E-01	1.34E-02
GLY	33	C	344	GLY	33	O	822	5.91E-01	-5.92E-01	-6.11E-01	-5.73E-01	1.89E-02
GLY	33	C	344	LEU	34	N	826	4.87E-01	-6.40E-01	-6.63E-01	-6.18E-01	2.26E-02
GLY	33	C	344	LEU	34	O	829	7.35E-01	-4.30E-01	-4.95E-01	-3.65E-01	6.50E-02
GLY	33	C	344	MET	35	O	848	5.93E-01	-6.81E-01	-7.73E-01	-5.90E-01	9.15E-02
GLY	33	C	344	VAL	36	N	862	6.76E-01	-4.02E-01	-4.41E-01	-3.64E-01	3.82E-02
GLY	33	O	345	ILE	31	C	783	7.26E-01	-3.82E-01	-3.95E-01	-3.69E-01	1.32E-02
GLY	33	O	345	ILE	32	C	802	4.21E-01	-1.30E+00	-1.36E+00	-1.24E+00	6.46E-02
GLY	33	O	345	ILE	32	H	808	5.96E-01	-3.30E-01	-3.42E-01	-3.17E-01	1.29E-02
GLY	33	O	345	GLY	33	C	821	3.94E-01	-1.57E+00	-1.63E+00	-1.51E+00	6.23E-02
GLY	33	O	345	GLY	33	H	823	4.95E-01	-3.86E-01	-3.98E-01	-3.73E-01	1.24E-02
GLY	33	O	345	GLY	33	HA2	824	2.67E-01	-7.53E-01	-8.84E-01	-6.21E-01	1.31E-01
GLY	33	O	345	LEU	34	C	828	5.70E-01	-6.32E-01	-6.53E-01	-6.11E-01	2.08E-02
GLY	33	O	345	LEU	34	H	834	3.59E-01	-9.68E-01	-1.15E+00	-7.84E-01	1.84E-01
GLY	33	O	345	MET	35	C	847	5.67E-01	-7.11E-01	-8.32E-01	-5.90E-01	1.21E-01
GLY	33	H	346	ILE	32	N	800	4.50E-01	-4.31E-01	-4.47E-01	-4.15E-01	1.61E-02
GLY	33	H	346	ILE	32	O	803	1.98E-01	-5.98E+00	-6.37E+00	-5.59E+00	3.88E-01
GLY	33	H	346	GLY	33	N	819	3.87E-01	-5.27E-01	-5.41E-01	-5.13E-01	1.40E-02
LEU	34	N	349	ILE	32	C	802	5.83E-01	-4.17E-01	-4.40E-01	-3.95E-01	2.27E-02
LEU	34	N	349	GLY	33	C	821	5.27E-01	-5.34E-01	-5.54E-01	-5.13E-01	2.07E-02
LEU	34	N	349	LEU	34	C	828	6.38E-01	-3.45E-01	-3.54E-01	-3.36E-01	9.10E-03

LEU	34	N	349	LEU	34	H	834	4.23E-01	-4.10E-01	-4.30E-01	-3.90E-01	2.01E-02
LEU	34	C	351	ILE	32	O	803	6.53E-01	-5.80E-01	-6.12E-01	-5.47E-01	3.24E-02
LEU	34	C	351	GLY	33	N	819	6.47E-01	-3.55E-01	-3.73E-01	-3.37E-01	1.80E-02
LEU	34	C	351	GLY	33	O	822	5.99E-01	-5.69E-01	-6.03E-01	-5.35E-01	3.43E-02
LEU	34	C	351	LEU	34	N	826	4.52E-01	-7.64E-01	-8.20E-01	-7.09E-01	5.56E-02
LEU	34	C	351	LEU	34	O	829	5.83E-01	-7.23E-01	-9.49E-01	-4.97E-01	2.26E-01
LEU	34	C	351	MET	35	N	845	4.17E-01	-1.11E+00	-1.27E+00	-9.57E-01	1.56E-01
LEU	34	C	351	MET	35	O	848	3.92E-01	-1.96E+00	-2.37E+00	-1.56E+00	4.01E-01
LEU	34	C	351	VAL	36	N	862	5.41E-01	-6.47E-01	-7.26E-01	-5.68E-01	7.93E-02
LEU	34	O	352	ILE	32	C	802	7.21E-01	-4.27E-01	-4.54E-01	-4.00E-01	2.67E-02
LEU	34	O	352	GLY	33	C	821	4.80E-01	-1.10E+00	-1.26E+00	-9.41E-01	1.59E-01
LEU	34	O	352	LEU	34	C	828	4.14E-01	-1.61E+00	-1.83E+00	-1.38E+00	2.25E-01
LEU	34	O	352	LEU	34	H	834	3.83E-01	-9.80E-01	-1.30E+00	-6.64E-01	3.16E-01
LEU	34	O	352	MET	35	C	847	3.66E-01	-2.75E+00	-3.41E+00	-2.08E+00	6.65E-01
LEU	34	H	357	ILE	32	O	803	5.59E-01	-3.70E-01	-4.00E-01	-3.41E-01	2.93E-02
LEU	34	HA	358	LEU	34	N	826	3.17E-01	-4.74E-01	-5.17E-01	-4.30E-01	4.37E-02
MET	35	N	368	GLY	33	C	821	6.07E-01	-4.48E-01	-5.16E-01	-3.79E-01	6.86E-02
MET	35	N	368	LEU	34	C	828	5.89E-01	-5.01E-01	-6.57E-01	-3.44E-01	1.57E-01
MET	35	N	368	MET	35	C	847	4.79E-01	-7.70E-01	-8.09E-01	-7.31E-01	3.89E-02
MET	35	C	370	ILE	32	O	803	8.30E-01	-3.87E-01	-4.04E-01	-3.69E-01	1.77E-02
MET	35	C	370	LEU	34	O	829	7.80E-01	-4.58E-01	-6.61E-01	-2.55E-01	2.03E-01
MET	35	C	370	MET	35	N	845	6.19E-01	-4.29E-01	-4.39E-01	-4.19E-01	1.00E-02
MET	35	C	370	MET	35	O	848	3.82E-01	-1.98E+00	-2.15E+00	-1.81E+00	1.69E-01
MET	35	C	370	VAL	36	N	862	5.71E-01	-5.81E-01	-5.97E-01	-5.65E-01	1.57E-02
MET	35	O	371	LEU	34	C	828	8.37E-01	-3.37E-01	-3.74E-01	-3.01E-01	3.67E-02
MET	35	O	371	MET	35	C	847	6.02E-01	-6.53E-01	-6.78E-01	-6.29E-01	2.44E-02
MET	35	H	376	MET	35	O	848	4.76E-01	-5.33E-01	-5.89E-01	-4.77E-01	5.62E-02
MET	35	HA	377	MET	35	O	848	2.52E-01	-1.30E+00	-1.42E+00	-1.18E+00	1.19E-01
VAL	36	N	385	GLY	33	C	821	6.91E-01	-3.84E-01	-4.20E-01	-3.49E-01	3.57E-02
VAL	36	N	385	LEU	34	C	828	6.95E-01	-3.79E-01	-4.34E-01	-3.25E-01	5.46E-02
VAL	36	N	385	MET	35	C	847	4.54E-01	-9.92E-01	-1.02E+00	-9.67E-01	2.53E-02
VAL	36	N	385	VAL	36	C	864	5.87E-01	-4.08E-01	-4.18E-01	-3.97E-01	1.05E-02
VAL	36	N	385	VAL	36	CB	866	4.83E-01	-5.64E-01	-5.91E-01	-5.37E-01	2.74E-02
VAL	36	N	385	VAL	36	H	869	5.98E-01	-3.86E-01	-3.99E-01	-3.73E-01	1.32E-02
VAL	36	C	387	MET	35	O	848	4.42E-01	-9.99E-01	-1.05E+00	-9.47E-01	5.24E-02
VAL	36	C	387	VAL	36	N	862	5.26E-01	-5.21E-01	-5.34E-01	-5.07E-01	1.37E-02
VAL	36	C	387	VAL	36	O	865	6.12E-01	-3.36E-01	-3.45E-01	-3.27E-01	9.18E-03
VAL	36	C	387	GLY	37	N	878	4.57E-01	-6.08E-01	-6.46E-01	-5.71E-01	3.77E-02
VAL	36	C	387	GLY	37	O	881	5.03E-01	-6.58E-01	-7.06E-01	-6.11E-01	4.75E-02
VAL	36	C	387	GLY	38	O	888	6.69E-01	-3.54E-01	-3.76E-01	-3.32E-01	2.19E-02
VAL	36	O	388	MET	35	C	847	4.52E-01	-9.07E-01	-9.51E-01	-8.63E-01	4.35E-02
VAL	36	O	388	VAL	36	C	864	3.87E-01	-1.01E+00	-1.05E+00	-9.66E-01	4.14E-02
VAL	36	O	388	VAL	36	CB	866	3.24E-01	-1.49E+00	-1.70E+00	-1.28E+00	2.06E-01
VAL	36	O	388	VAL	36	H	869	5.16E-01	-4.83E-01	-4.99E-01	-4.67E-01	1.57E-02
VAL	36	O	388	GLY	37	C	880	5.08E-01	-6.66E-01	-7.14E-01	-6.18E-01	4.82E-02
VAL	36	O	388	GLY	37	H	882	2.87E-01	-1.44E+00	-1.77E+00	-1.11E+00	3.33E-01

VAL	36	O	388	GLY	38	C	887	6.76E-01	-3.57E-01	-3.76E-01	-3.38E-01	1.94E-02
VAL	36	CB	389	MET	35	O	848	5.55E-01	-5.18E-01	-5.54E-01	-4.82E-01	3.61E-02
VAL	36	H	392	ILE	32	O	803	6.82E-01	-4.08E-01	-4.28E-01	-3.87E-01	2.10E-02
VAL	36	H	392	LEU	34	O	829	6.66E-01	-4.73E-01	-7.19E-01	-2.27E-01	2.46E-01
VAL	36	H	392	MET	35	N	845	5.20E-01	-4.62E-01	-4.78E-01	-4.46E-01	1.64E-02
VAL	36	H	392	MET	35	O	848	2.90E-01	-3.34E+00	-4.11E+00	-2.58E+00	7.68E-01
VAL	36	H	392	VAL	36	N	862	4.29E-01	-8.48E-01	-9.03E-01	-7.93E-01	5.49E-02
VAL	36	H	392	GLY	37	N	878	5.57E-01	-3.77E-01	-4.01E-01	-3.53E-01	2.38E-02
GLY	37	N	401	MET	35	C	847	6.23E-01	-4.03E-01	-4.30E-01	-3.77E-01	2.67E-02
GLY	37	N	401	VAL	36	C	864	5.52E-01	-3.89E-01	-4.06E-01	-3.72E-01	1.70E-02
GLY	37	N	401	GLY	37	C	880	5.66E-01	-4.79E-01	-5.03E-01	-4.55E-01	2.38E-02
GLY	37	N	401	GLY	37	H	882	3.98E-01	-4.95E-01	-5.32E-01	-4.58E-01	3.71E-02
GLY	37	C	403	MET	35	O	848	7.32E-01	-4.28E-01	-4.50E-01	-4.06E-01	2.21E-02
GLY	37	C	403	GLY	37	N	878	5.15E-01	-5.95E-01	-6.28E-01	-5.61E-01	3.37E-02
GLY	37	C	403	GLY	37	O	881	3.66E-01	-1.92E+00	-2.05E+00	-1.79E+00	1.31E-01
GLY	37	C	403	GLY	38	N	885	5.74E-01	-4.64E-01	-4.83E-01	-4.45E-01	1.89E-02
GLY	37	C	403	GLY	38	O	888	4.63E-01	-1.05E+00	-1.12E+00	-9.70E-01	7.71E-02
GLY	37	C	403	VAL	39	N	892	6.72E-01	-4.00E-01	-4.14E-01	-3.86E-01	1.41E-02
GLY	37	O	404	GLY	37	C	880	5.96E-01	-5.82E-01	-6.06E-01	-5.58E-01	2.43E-02
GLY	37	O	404	GLY	38	C	887	6.50E-01	-4.84E-01	-5.01E-01	-4.67E-01	1.72E-02
GLY	37	HA2	406	GLY	37	O	881	2.75E-01	-6.70E-01	-7.52E-01	-5.88E-01	8.23E-02
GLY	38	N	408	GLY	37	C	880	4.06E-01	-1.07E+00	-1.14E+00	-1.00E+00	7.05E-02
GLY	38	N	408	GLY	37	H	882	4.39E-01	-3.90E-01	-4.35E-01	-3.44E-01	4.52E-02
GLY	38	N	408	GLY	38	C	887	4.41E-01	-8.69E-01	-9.36E-01	-8.02E-01	6.68E-02
GLY	38	C	410	GLY	37	O	881	4.65E-01	-1.04E+00	-1.12E+00	-9.59E-01	8.06E-02
GLY	38	C	410	GLY	38	N	885	6.25E-01	-3.86E-01	-3.97E-01	-3.75E-01	1.06E-02
GLY	38	C	410	GLY	38	O	888	3.60E-01	-2.00E+00	-2.14E+00	-1.86E+00	1.43E-01
GLY	38	C	410	VAL	39	N	892	5.72E-01	-5.63E-01	-5.85E-01	-5.40E-01	2.24E-02
GLY	38	C	410	VAL	40	N	908	6.54E-01	-4.24E-01	-4.38E-01	-4.09E-01	1.47E-02
GLY	38	O	411	GLY	37	C	880	6.90E-01	-4.30E-01	-4.47E-01	-4.14E-01	1.66E-02
GLY	38	O	411	GLY	38	C	887	6.00E-01	-5.72E-01	-5.92E-01	-5.52E-01	1.98E-02
GLY	38	O	411	VAL	40	H	915	6.51E-01	-3.67E-01	-3.89E-01	-3.45E-01	2.22E-02
GLY	38	H	412	GLY	37	N	878	4.24E-01	-4.24E-01	-4.71E-01	-3.78E-01	4.68E-02
GLY	38	H	412	GLY	37	O	881	2.04E-01	-4.68E+00	-5.36E+00	-3.99E+00	6.85E-01
GLY	38	H	412	GLY	38	N	885	3.95E-01	-5.02E-01	-5.32E-01	-4.71E-01	3.10E-02
GLY	38	H	412	GLY	38	O	888	3.00E-01	-1.53E+00	-1.82E+00	-1.25E+00	2.85E-01
VAL	39	N	415	GLY	37	C	880	5.52E-01	-6.09E-01	-6.36E-01	-5.81E-01	2.75E-02
VAL	39	N	415	GLY	38	C	887	4.09E-01	-1.26E+00	-1.30E+00	-1.21E+00	4.46E-02
VAL	39	N	415	VAL	39	C	894	5.53E-01	-4.65E-01	-4.83E-01	-4.48E-01	1.77E-02
VAL	39	N	415	VAL	39	CB	896	5.21E-01	-4.76E-01	-5.12E-01	-4.39E-01	3.67E-02
VAL	39	N	415	VAL	39	H	899	5.75E-01	-4.20E-01	-4.30E-01	-4.09E-01	1.04E-02
VAL	39	N	415	VAL	40	H	915	4.56E-01	-7.21E-01	-7.42E-01	-7.00E-01	2.07E-02
VAL	39	C	417	GLY	37	O	881	6.45E-01	-3.79E-01	-3.96E-01	-3.62E-01	1.72E-02
VAL	39	C	417	GLY	38	O	888	4.20E-01	-1.03E+00	-1.11E+00	-9.50E-01	7.82E-02
VAL	39	C	417	VAL	39	N	892	5.22E-01	-5.31E-01	-5.56E-01	-5.06E-01	2.52E-02
VAL	39	C	417	VAL	39	O	895	6.01E-01	-3.49E-01	-3.57E-01	-3.41E-01	8.04E-03

VAL	39	C	417	VAL	40	N	908	4.09E-01	-9.66E-01	-9.98E-01	-9.34E-01	3.19E-02
VAL	39	C	417	VAL	40	O	911	4.41E-01	-7.20E-01	-7.44E-01	-6.95E-01	2.40E-02
VAL	39	C	417	ILE	41	N	924	6.39E-01	-3.41E-01	-3.48E-01	-3.35E-01	6.82E-03
VAL	39	O	418	GLY	37	C	880	6.85E-01	-3.47E-01	-3.57E-01	-3.37E-01	9.78E-03
VAL	39	O	418	GLY	38	C	887	4.34E-01	-9.82E-01	-1.06E+00	-9.05E-01	7.69E-02
VAL	39	O	418	VAL	39	C	894	3.63E-01	-1.19E+00	-1.22E+00	-1.16E+00	3.28E-02
VAL	39	O	418	VAL	39	CB	896	4.28E-01	-6.89E-01	-7.34E-01	-6.43E-01	4.52E-02
VAL	39	O	418	VAL	39	H	899	5.13E-01	-4.91E-01	-5.17E-01	-4.64E-01	2.69E-02
VAL	39	O	418	VAL	40	C	910	4.29E-01	-7.72E-01	-8.07E-01	-7.36E-01	3.60E-02
VAL	39	O	418	VAL	40	CB	912	4.29E-01	-6.81E-01	-7.13E-01	-6.49E-01	3.16E-02
VAL	39	O	418	VAL	40	H	915	2.00E-01	-6.57E+00	-6.65E+00	-6.50E+00	7.33E-02
VAL	39	CB	419	GLY	37	O	881	5.67E-01	-4.42E-01	-4.67E-01	-4.17E-01	2.52E-02
VAL	39	CB	419	GLY	38	O	888	4.25E-01	-9.14E-01	-1.08E+00	-7.43E-01	1.70E-01
VAL	39	CB	419	VAL	39	N	892	5.42E-01	-4.37E-01	-4.81E-01	-3.93E-01	4.41E-02
VAL	39	CB	419	VAL	40	N	908	5.33E-01	-4.49E-01	-4.77E-01	-4.21E-01	2.83E-02
VAL	39	H	422	GLY	37	O	881	3.79E-01	-1.34E+00	-1.50E+00	-1.19E+00	1.58E-01
VAL	39	H	422	GLY	38	N	885	4.99E-01	-4.84E-01	-5.13E-01	-4.55E-01	2.92E-02
VAL	39	H	422	GLY	38	O	888	1.98E-01	-8.53E+00	-9.01E+00	-8.05E+00	4.80E-01
VAL	39	H	422	VAL	39	N	892	3.88E-01	-1.09E+00	-1.15E+00	-1.04E+00	5.55E-02
VAL	39	H	422	VAL	39	O	895	5.94E-01	-3.53E-01	-3.73E-01	-3.34E-01	1.95E-02
VAL	39	H	422	VAL	40	N	908	4.85E-01	-6.27E-01	-6.67E-01	-5.86E-01	4.04E-02
VAL	40	N	431	GLY	38	C	887	6.35E-01	-4.50E-01	-4.69E-01	-4.31E-01	1.91E-02
VAL	40	N	431	VAL	39	C	894	5.71E-01	-4.34E-01	-4.47E-01	-4.21E-01	1.30E-02
VAL	40	N	431	VAL	40	C	910	5.27E-01	-5.18E-01	-5.33E-01	-5.04E-01	1.43E-02
VAL	40	N	431	VAL	40	CB	912	5.55E-01	-4.08E-01	-4.15E-01	-4.01E-01	7.35E-03
VAL	40	N	431	VAL	40	H	915	3.84E-01	-1.13E+00	-1.18E+00	-1.07E+00	5.45E-02
VAL	40	C	433	VAL	40	N	908	5.55E-01	-4.61E-01	-4.69E-01	-4.53E-01	7.81E-03
VAL	40	C	433	VAL	40	O	911	3.65E-01	-1.17E+00	-1.22E+00	-1.13E+00	4.58E-02
VAL	40	C	433	ILE	41	N	924	5.81E-01	-4.18E-01	-4.29E-01	-4.07E-01	1.15E-02
VAL	40	O	434	VAL	40	C	910	6.08E-01	-3.40E-01	-3.46E-01	-3.34E-01	5.67E-03
VAL	40	O	434	VAL	40	H	915	5.89E-01	-3.59E-01	-3.64E-01	-3.53E-01	5.90E-03
VAL	40	CB	435	VAL	40	N	908	5.29E-01	-4.54E-01	-4.66E-01	-4.42E-01	1.19E-02
VAL	40	CB	435	VAL	40	O	911	4.31E-01	-6.75E-01	-7.09E-01	-6.40E-01	3.47E-02
VAL	40	H	438	GLY	38	O	888	6.02E-01	-4.31E-01	-4.53E-01	-4.08E-01	2.26E-02
VAL	40	H	438	VAL	40	N	908	5.73E-01	-4.22E-01	-4.35E-01	-4.10E-01	1.28E-02
VAL	40	H	438	VAL	40	O	911	5.14E-01	-4.88E-01	-5.09E-01	-4.66E-01	2.18E-02
ILE	41	N	447	VAL	39	C	894	6.59E-01	-3.20E-01	-3.27E-01	-3.14E-01	6.31E-03
ILE	41	N	447	VAL	40	C	910	4.15E-01	-9.34E-01	-9.57E-01	-9.11E-01	2.29E-02
ILE	41	N	447	VAL	40	CB	912	5.41E-01	-4.32E-01	-4.41E-01	-4.22E-01	9.41E-03
ILE	41	N	447	VAL	40	H	915	4.78E-01	-6.44E-01	-6.59E-01	-6.29E-01	1.53E-02
ILE	41	N	447	ILE	41	C	926	5.61E-01	-5.77E-01	-6.12E-01	-5.42E-01	3.53E-02
ILE	41	N	447	ILE	41	HA	933	3.66E-01	-5.13E-01	-5.63E-01	-4.64E-01	4.95E-02
ILE	41	N	447	ALA	42	H	949	4.82E-01	-4.33E-01	-4.85E-01	-3.81E-01	5.19E-02
ILE	41	C	449	VAL	40	N	908	7.07E-01	-3.55E-01	-3.65E-01	-3.44E-01	1.01E-02
ILE	41	C	449	VAL	40	O	911	4.21E-01	-1.04E+00	-1.11E+00	-9.66E-01	7.01E-02
ILE	41	C	449	ILE	41	N	924	5.31E-01	-6.52E-01	-6.79E-01	-6.24E-01	2.73E-02

ILE	41	C	449	ILE	41	O	927	6.06E-01	-6.69E-01	-6.84E-01	-6.53E-01	1.54E-02
ILE	41	C	449	ALA	42	N	943	4.24E-01	-1.02E+00	-1.09E+00	-9.43E-01	7.56E-02
ILE	41	C	449	ALA	42	O	946	6.19E-01	-5.76E-01	-6.18E-01	-5.34E-01	4.20E-02
ILE	41	C	449	ALA	42	CB	947	4.76E-01	-4.46E-01	-5.17E-01	-3.76E-01	7.07E-02
ILE	41	C	449	ALA	42	OXT	948	7.23E-01	-4.22E-01	-4.46E-01	-3.97E-01	2.49E-02
ILE	41	O	450	LYS	28	C	744	7.79E-01	-5.24E-01	-5.57E-01	-4.91E-01	3.28E-02
ILE	41	O	450	GLY	29	C	766	7.96E-01	-4.03E-01	-4.26E-01	-3.80E-01	2.33E-02
ILE	41	O	450	VAL	39	C	894	7.67E-01	-3.31E-01	-3.44E-01	-3.17E-01	1.35E-02
ILE	41	O	450	VAL	40	C	910	4.31E-01	-1.18E+00	-1.29E+00	-1.07E+00	1.13E-01
ILE	41	O	450	VAL	40	CB	912	5.79E-01	-5.13E-01	-5.41E-01	-4.86E-01	2.78E-02
ILE	41	O	450	VAL	40	H	915	6.23E-01	-4.90E-01	-5.17E-01	-4.63E-01	2.70E-02
ILE	41	O	450	ILE	41	C	926	3.83E-01	-2.01E+00	-2.10E+00	-1.93E+00	8.30E-02
ILE	41	O	450	ILE	41	H	932	5.24E-01	-5.37E-01	-5.69E-01	-5.04E-01	3.23E-02
ILE	41	O	450	ILE	41	HA	933	2.67E-01	-1.78E+00	-2.09E+00	-1.47E+00	3.10E-01
ILE	41	O	450	ALA	42	C	945	5.36E-01	-8.91E-01	-9.74E-01	-8.09E-01	8.28E-02
ILE	41	O	450	ALA	42	H	949	2.49E-01	-3.67E+00	-4.27E+00	-3.07E+00	6.03E-01
ILE	41	H	455	VAL	40	N	908	4.58E-01	-5.35E-01	-5.49E-01	-5.20E-01	1.50E-02
ILE	41	H	455	VAL	40	O	911	1.99E-01	-5.00E+00	-5.26E+00	-4.74E+00	2.58E-01
ILE	41	H	455	ILE	41	N	924	3.93E-01	-7.92E-01	-8.29E-01	-7.54E-01	3.74E-02
ILE	41	H	455	ILE	41	O	927	5.85E-01	-4.18E-01	-4.39E-01	-3.97E-01	2.09E-02
ILE	41	H	455	ALA	42	N	943	4.91E-01	-4.13E-01	-4.55E-01	-3.71E-01	4.20E-02
ALA	42	N	466	ILE	41	C	926	5.69E-01	-5.01E-01	-5.21E-01	-4.80E-01	2.01E-02
ALA	42	N	466	ALA	42	C	945	6.34E-01	-3.98E-01	-4.14E-01	-3.82E-01	1.60E-02
ALA	42	N	466	ALA	42	H	949	3.89E-01	-6.52E-01	-6.88E-01	-6.16E-01	3.57E-02
ALA	42	C	468	LYS	28	O	745	6.93E-01	-4.79E-01	-5.45E-01	-4.12E-01	6.70E-02
ALA	42	C	468	LYS	28	NZ	750	4.15E-01	-7.75E-01	-1.01E+00	-5.35E-01	2.39E-01
ALA	42	C	468	GLY	29	N	764	6.12E-01	-4.04E-01	-4.47E-01	-3.61E-01	4.30E-02
ALA	42	C	468	ILE	41	O	927	6.41E-01	-5.99E-01	-6.33E-01	-5.65E-01	3.42E-02
ALA	42	C	468	ALA	42	N	943	4.18E-01	-1.07E+00	-1.18E+00	-9.55E-01	1.15E-01
ALA	42	C	468	ALA	42	O	946	4.55E-01	-1.18E+00	-1.29E+00	-1.06E+00	1.17E-01
ALA	42	C	468	ALA	42	CB	947	3.43E-01	-1.03E+00	-1.16E+00	-9.09E-01	1.24E-01
ALA	42	C	468	ALA	42	OXT	948	5.77E-01	-6.69E-01	-7.11E-01	-6.28E-01	4.15E-02
ALA	42	O	469	LYS	28	C	744	6.05E-01	-8.08E-01	-9.41E-01	-6.74E-01	1.34E-01
ALA	42	O	469	LYS	28	HZ1	761	4.71E-01	-6.22E-01	-7.91E-01	-4.53E-01	1.69E-01
ALA	42	O	469	LYS	28	HZ2	762	4.10E-01	-1.10E+00	-1.66E+00	-5.36E-01	5.62E-01
ALA	42	O	469	LYS	28	HZ3	763	4.11E-01	-1.17E+00	-1.63E+00	-7.05E-01	4.64E-01
ALA	42	O	469	ILE	41	C	926	6.08E-01	-6.22E-01	-7.24E-01	-5.20E-01	1.02E-01
ALA	42	O	469	ALA	42	C	945	5.33E-01	-8.05E-01	-8.72E-01	-7.39E-01	6.66E-02
ALA	42	O	469	ALA	42	H	949	4.18E-01	-8.53E-01	-1.12E+00	-5.87E-01	2.66E-01
ALA	42	OXT	471	LYS	28	C	744	6.09E-01	-7.84E-01	-9.01E-01	-6.68E-01	1.16E-01
ALA	42	OXT	471	LYS	28	HZ1	761	3.97E-01	-1.20E+00	-1.75E+00	-6.59E-01	5.44E-01
ALA	42	OXT	471	LYS	28	HZ2	762	3.58E-01	-1.76E+00	-2.90E+00	-6.19E-01	1.14E+00
ALA	42	OXT	471	LYS	28	HZ3	763	3.42E-01	-2.88E+00	-4.75E+00	-1.01E+00	1.87E+00
ALA	42	OXT	471	ILE	41	C	926	4.80E-01	-1.06E+00	-1.22E+00	-8.98E-01	1.61E-01
ALA	42	OXT	471	ALA	42	C	945	3.78E-01	-1.95E+00	-2.26E+00	-1.64E+00	3.13E-01
ALA	42	OXT	471	ALA	42	H	949	2.96E-01	-2.23E+00	-2.86E+00	-1.59E+00	6.37E-01

ALA	42	OXT	471	ALA	42	HB2	952	2.74E-01	-6.61E-01	-7.78E-01	-5.43E-01	1.17E-01

Supplementary Table 9b: Mapping results for Aβ42's (PDB ID: 5KK3) short range (1:2) dominant atom-atom Lennard-Jones interactions across ensemble structures. Columns for each chain correspond to: residue abbreviation, residue number in peptide sequence, atom identity (IUPAC naming convention) and atom number in PDB file. Energy in *kT*, distance in *nm*. Mapping analysis began on the 11th residue for both isoforms because original structure data for Aβ42 begins with the 11th residue.

Chain 1				Chain 2				Average Distance	Average L-J Values	Lower 95% Confidence Interval Bound	Upper 95% Confidence Interval Bound	Margin of Error
HIS	14	C	51	GLN	15	N	543	4.07E-01	-1.64E-01	-1.71E-01	-1.57E-01	6.86E-03
HIS	14	O	52	HIS	14	CA	527	3.83E-01	-1.97E-01	-2.19E-01	-1.75E-01	2.21E-02
HIS	14	O	52	HIS	14	C	528	3.81E-01	-1.93E-01	-2.04E-01	-1.82E-01	1.11E-02
HIS	14	O	52	GLN	15	CA	544	3.67E-01	-2.16E-01	-2.30E-01	-2.02E-01	1.40E-02
HIS	14	O	52	GLN	15	CB	547	3.72E-01	-2.04E-01	-2.32E-01	-1.76E-01	2.78E-02
GLN	15	CA	67	GLN	15	CB	547	4.14E-01	-1.47E-01	-1.63E-01	-1.32E-01	1.53E-02
GLN	15	C	68	GLN	15	O	546	3.63E-01	-2.12E-01	-2.17E-01	-2.08E-01	4.46E-03
LYS	16	N	83	GLN	15	C	545	4.06E-01	-1.65E-01	-1.72E-01	-1.58E-01	7.01E-03
LYS	16	CA	84	GLN	15	O	546	3.85E-01	-1.92E-01	-2.12E-01	-1.73E-01	1.97E-02
LYS	16	C	85	LEU	17	N	582	3.96E-01	-1.76E-01	-1.84E-01	-1.68E-01	7.90E-03
LYS	16	O	86	LYS	16	CA	561	3.84E-01	-1.90E-01	-2.20E-01	-1.61E-01	2.96E-02
LYS	16	O	86	LYS	16	C	562	3.74E-01	-1.98E-01	-2.07E-01	-1.89E-01	9.39E-03
LEU	17	CA	106	LEU	17	CB	586	4.18E-01	-1.45E-01	-1.58E-01	-1.32E-01	1.31E-02
LEU	17	C	107	LEU	17	O	585	3.65E-01	-2.10E-01	-2.17E-01	-2.03E-01	6.95E-03
VAL	18	N	124	LEU	17	C	584	4.06E-01	-1.65E-01	-1.73E-01	-1.57E-01	7.93E-03
VAL	18	CA	125	LEU	17	O	585	3.84E-01	-2.10E-01	-2.24E-01	-1.95E-01	1.43E-02
VAL	18	C	126	LEU	17	O	585	4.00E-01	-1.61E-01	-1.84E-01	-1.38E-01	2.27E-02
VAL	18	O	127	VAL	18	CA	602	3.65E-01	-2.11E-01	-2.29E-01	-1.92E-01	1.86E-02
VAL	18	O	127	VAL	18	C	603	3.89E-01	-1.82E-01	-1.91E-01	-1.73E-01	8.70E-03
ASP	23	O	208	ASP	23	C	684	3.87E-01	-1.85E-01	-1.94E-01	-1.76E-01	9.26E-03
ASP	23	O	208	VAL	24	N	694	3.55E-01	-2.83E-01	-3.06E-01	-2.60E-01	2.31E-02
VAL	24	C	219	VAL	24	O	697	3.85E-01	-1.87E-01	-2.03E-01	-1.71E-01	1.63E-02
GLY	25	N	233	VAL	24	C	696	4.07E-01	-1.60E-01	-1.78E-01	-1.42E-01	1.77E-02
GLY	25	N	233	GLY	25	O	713	4.10E-01	-1.93E-01	-2.24E-01	-1.61E-01	3.14E-02
GLY	25	CA	234	GLY	25	O	713	3.79E-01	-2.01E-01	-2.24E-01	-1.78E-01	2.33E-02
GLY	25	C	235	GLY	25	O	713	3.69E-01	-2.03E-01	-2.10E-01	-1.96E-01	7.12E-03
SER	26	N	240	GLY	25	C	712	3.97E-01	-1.77E-01	-1.84E-01	-1.69E-01	7.75E-03
SER	26	CA	241	GLY	25	O	713	3.53E-01	-2.19E-01	-2.37E-01	-2.02E-01	1.76E-02
SER	26	C	242	ASN	27	N	728	3.91E-01	-1.84E-01	-1.89E-01	-1.78E-01	5.54E-03
SER	26	O	243	SER	26	CA	718	3.83E-01	-1.83E-01	-2.10E-01	-1.56E-01	2.72E-02
SER	26	O	243	SER	26	C	719	3.74E-01	-1.99E-01	-2.10E-01	-1.88E-01	1.10E-02
SER	26	CB	244	SER	26	CA	718	4.15E-01	-1.49E-01	-1.62E-01	-1.36E-01	1.30E-02
ASN	27	CA	252	ASN	27	O	731	3.63E-01	-1.74E-01	-2.04E-01	-1.44E-01	3.01E-02
ASN	27	C	253	ASN	27	O	731	3.72E-01	-2.01E-01	-2.10E-01	-1.93E-01	8.40E-03
LYS	28	N	265	ASN	27	C	730	4.09E-01	-1.61E-01	-1.68E-01	-1.53E-01	7.38E-03
LYS	28	CA	266	ASN	27	O	731	3.77E-01	-1.97E-01	-2.18E-01	-1.76E-01	2.09E-02
LYS	28	C	267	ASN	27	O	731	3.93E-01	-1.64E-01	-1.81E-01	-1.48E-01	1.66E-02
LYS	28	C	267	GLY	29	N	764	4.21E-01	-1.45E-01	-1.57E-01	-1.33E-01	1.20E-02
LYS	28	O	268	LYS	28	N	742	4.25E-01	-1.61E-01	-1.81E-01	-1.41E-01	2.02E-02

LYS	28	O	268	LYS	28	CA	743	3.46E-01	-2.14E-01	-2.35E-01	-1.93E-01	2.11E-02
LYS	28	O	268	LYS	28	C	744	3.73E-01	-2.04E-01	-2.11E-01	-1.96E-01	7.33E-03
LYS	28	O	268	GLY	29	CA	765	4.11E-01	-1.68E-01	-1.94E-01	-1.43E-01	2.54E-02
GLY	29	CA	288	GLY	29	N	764	4.41E-01	-1.34E-01	-1.47E-01	-1.20E-01	1.32E-02
GLY	29	C	289	ALA	30	N	771	3.80E-01	-1.91E-01	-1.96E-01	-1.87E-01	4.46E-03
GLY	29	O	290	GLY	29	N	764	4.00E-01	-2.11E-01	-2.58E-01	-1.63E-01	4.79E-02
GLY	29	O	290	GLY	29	CA	765	3.81E-01	-1.95E-01	-2.17E-01	-1.72E-01	2.22E-02
GLY	29	O	290	GLY	29	C	766	3.68E-01	-2.05E-01	-2.12E-01	-1.98E-01	6.90E-03
ALA	30	N	294	ALA	30	O	774	4.06E-01	-1.98E-01	-2.20E-01	-1.75E-01	2.23E-02
ALA	30	CA	295	ALA	30	C	773	4.30E-01	-1.16E-01	-1.20E-01	-1.11E-01	4.42E-03
ALA	30	CA	295	ALA	30	CB	775	4.29E-01	-1.32E-01	-1.44E-01	-1.21E-01	1.16E-02
ALA	30	C	296	ALA	30	O	774	3.56E-01	-2.17E-01	-2.18E-01	-2.15E-01	1.65E-03
ALA	30	CB	298	ALA	30	O	774	4.29E-01	-1.38E-01	-1.56E-01	-1.20E-01	1.84E-02
ILE	31	N	304	ALA	30	C	773	4.07E-01	-1.64E-01	-1.70E-01	-1.57E-01	6.62E-03
ILE	31	N	304	ILE	31	CA	782	4.57E-01	-1.12E-01	-1.18E-01	-1.07E-01	5.47E-03
ILE	31	CA	305	ALA	30	O	774	3.94E-01	-1.97E-01	-2.09E-01	-1.84E-01	1.28E-02
ILE	31	C	306	ALA	30	O	774	4.15E-01	-1.41E-01	-1.57E-01	-1.26E-01	1.56E-02
ILE	31	C	306	ILE	32	N	800	4.14E-01	-1.54E-01	-1.58E-01	-1.50E-01	3.82E-03
ILE	31	O	307	ALA	30	O	774	3.56E-01	-2.88E-01	-3.09E-01	-2.66E-01	2.11E-02
ILE	31	O	307	ILE	31	N	781	4.32E-01	-1.47E-01	-1.58E-01	-1.37E-01	1.03E-02
ILE	31	O	307	ILE	31	CA	782	3.40E-01	-2.07E-01	-2.24E-01	-1.90E-01	1.68E-02
ILE	31	O	307	ILE	31	C	783	3.65E-01	-2.14E-01	-2.16E-01	-2.11E-01	2.43E-03
ILE	31	O	307	ILE	31	CB	785	4.28E-01	-1.38E-01	-1.47E-01	-1.29E-01	8.99E-03
ILE	31	O	307	ILE	32	CA	801	3.97E-01	-1.91E-01	-1.99E-01	-1.83E-01	8.17E-03
ILE	31	O	307	ILE	32	C	802	4.27E-01	-1.24E-01	-1.34E-01	-1.15E-01	9.30E-03
ILE	31	O	307	ILE	32	O	803	3.75E-01	-2.51E-01	-2.74E-01	-2.27E-01	2.39E-02
ILE	31	O	307	ILE	32	CB	804	4.29E-01	-1.36E-01	-1.47E-01	-1.26E-01	1.05E-02
ILE	32	N	323	ILE	32	O	803	4.32E-01	-1.47E-01	-1.55E-01	-1.38E-01	8.72E-03
ILE	32	C	325	ILE	32	O	803	3.61E-01	-2.16E-01	-2.17E-01	-2.15E-01	1.29E-03
ILE	32	CB	327	ILE	32	O	803	4.09E-01	-1.69E-01	-1.81E-01	-1.58E-01	1.16E-02
GLY	33	N	342	ILE	32	C	802	4.13E-01	-1.55E-01	-1.60E-01	-1.51E-01	4.76E-03
GLY	33	N	342	GLY	33	CA	820	4.53E-01	-1.18E-01	-1.24E-01	-1.12E-01	6.00E-03
GLY	33	CA	343	ILE	32	O	803	4.00E-01	-1.85E-01	-1.93E-01	-1.77E-01	8.21E-03
GLY	33	C	344	ILE	32	O	803	4.00E-01	-1.65E-01	-1.75E-01	-1.55E-01	9.72E-03
GLY	33	C	344	GLY	33	CA	820	3.92E-01	-1.53E-01	-1.54E-01	-1.51E-01	1.84E-03
GLY	33	O	345	ILE	32	C	802	4.21E-01	-1.34E-01	-1.45E-01	-1.22E-01	1.12E-02
GLY	33	O	345	ILE	32	O	803	3.53E-01	-3.04E-01	-3.23E-01	-2.84E-01	1.95E-02
GLY	33	O	345	GLY	33	N	819	4.07E-01	-1.96E-01	-2.10E-01	-1.82E-01	1.39E-02
GLY	33	O	345	GLY	33	C	821	3.94E-01	-1.75E-01	-1.84E-01	-1.67E-01	8.63E-03
GLY	33	O	345	LEU	34	N	826	4.08E-01	-1.96E-01	-2.23E-01	-1.69E-01	2.69E-02
LEU	34	CA	350	LEU	34	N	826	4.17E-01	-1.68E-01	-1.82E-01	-1.55E-01	1.37E-02
MET	35	C	370	MET	35	O	848	3.82E-01	-1.91E-01	-2.08E-01	-1.74E-01	1.72E-02
VAL	36	N	385	MET	35	O	848	3.51E-01	-2.67E-01	-2.92E-01	-2.41E-01	2.54E-02
VAL	36	C	387	VAL	36	CA	863	4.25E-01	-1.22E-01	-1.28E-01	-1.16E-01	6.23E-03
VAL	36	O	388	MET	35	O	848	3.88E-01	-2.18E-01	-2.43E-01	-1.92E-01	2.56E-02
VAL	36	O	388	VAL	36	N	862	4.30E-01	-1.51E-01	-1.62E-01	-1.39E-01	1.19E-02

VAL	36	O	388	VAL	36	C	864	3.87E-01	-1.85E-01	-1.94E-01	-1.77E-01	8.24E-03
GLY	37	CA	402	GLY	37	N	878	4.17E-01	-1.69E-01	-1.84E-01	-1.55E-01	1.44E-02
GLY	37	CA	402	GLY	37	O	881	3.53E-01	-2.09E-01	-2.44E-01	-1.75E-01	3.46E-02
GLY	37	C	403	GLY	37	O	881	3.66E-01	-2.06E-01	-2.12E-01	-1.99E-01	6.62E-03
GLY	38	N	408	GLY	37	C	880	4.06E-01	-1.65E-01	-1.78E-01	-1.51E-01	1.38E-02
GLY	38	CA	409	GLY	37	O	881	3.85E-01	-2.06E-01	-2.27E-01	-1.85E-01	2.11E-02
GLY	38	C	410	GLY	38	O	888	3.60E-01	-2.06E-01	-2.12E-01	-2.00E-01	5.93E-03
VAL	39	N	415	GLY	38	C	887	4.09E-01	-1.61E-01	-1.68E-01	-1.54E-01	6.93E-03
VAL	39	CA	416	GLY	38	O	888	3.91E-01	-1.95E-01	-2.07E-01	-1.83E-01	1.16E-02
VAL	39	C	417	GLY	38	O	888	4.20E-01	-1.35E-01	-1.51E-01	-1.18E-01	1.65E-02
VAL	39	C	417	VAL	40	N	908	4.09E-01	-1.61E-01	-1.68E-01	-1.54E-01	6.78E-03
VAL	39	O	418	GLY	38	O	888	3.66E-01	-2.71E-01	-2.99E-01	-2.43E-01	2.76E-02
VAL	39	O	418	VAL	39	N	892	4.27E-01	-1.56E-01	-1.73E-01	-1.38E-01	1.74E-02
VAL	39	O	418	VAL	39	CA	893	3.38E-01	-1.91E-01	-2.21E-01	-1.60E-01	3.02E-02
VAL	39	O	418	VAL	39	C	894	3.63E-01	-2.15E-01	-2.17E-01	-2.12E-01	2.27E-03
VAL	39	O	418	VAL	39	CB	896	4.28E-01	-1.40E-01	-1.56E-01	-1.24E-01	1.61E-02
VAL	39	O	418	VAL	40	CA	909	3.97E-01	-1.91E-01	-1.95E-01	-1.86E-01	4.69E-03
VAL	39	O	418	VAL	40	O	911	3.77E-01	-2.45E-01	-2.69E-01	-2.21E-01	2.41E-02
VAL	39	O	418	VAL	40	CB	912	4.29E-01	-1.37E-01	-1.48E-01	-1.26E-01	1.11E-02
VAL	40	N	431	VAL	40	O	911	4.31E-01	-1.50E-01	-1.62E-01	-1.37E-01	1.22E-02
VAL	40	CA	432	VAL	40	O	911	3.40E-01	-1.97E-01	-2.27E-01	-1.67E-01	3.01E-02
VAL	40	C	433	VAL	40	O	911	3.65E-01	-2.12E-01	-2.15E-01	-2.09E-01	3.45E-03
VAL	40	CG2	437	VAL	40	O	911	4.19E-01	-1.53E-01	-1.71E-01	-1.36E-01	1.72E-02
ILE	41	N	447	VAL	40	C	910	4.15E-01	-1.53E-01	-1.58E-01	-1.48E-01	5.11E-03
ILE	41	CA	448	VAL	40	O	911	3.97E-01	-1.92E-01	-1.98E-01	-1.86E-01	5.94E-03
ILE	41	C	449	ALA	42	N	943	4.24E-01	-1.41E-01	-1.56E-01	-1.26E-01	1.49E-02
ILE	41	O	450	VAL	40	O	911	3.57E-01	-2.73E-01	-3.02E-01	-2.43E-01	2.98E-02
ILE	41	O	450	ILE	41	CA	925	3.58E-01	-2.10E-01	-2.26E-01	-1.94E-01	1.57E-02
ILE	41	O	450	ILE	41	C	926	3.83E-01	-1.92E-01	-2.00E-01	-1.83E-01	8.67E-03

Supplementary Table 10: Mapping results for Aβ42's (PDB ID: 5KK3) long range (1:3) dominant atom-atom Coulombic interactions across ensemble structures. Columns for each chain correspond to: residue abbreviation, residue number in peptide sequence, atom identity (IUPAC naming convention) and atom number in PDB file. Energy in *kT*, distance in *nm*. Mapping analysis began on the 11th residue for both isoforms because original structure data for Aβ42 begins with the 11th residue.

Chain 1				Chain 2				Average Distance	Average Coulombic Values	Lower 95% Confidence Interval Bound	Upper 95% Confidence Interval Bound	Margin of Error
HIS	14	C	51	GLN	15	O	1023	8.04E-01	-4.09E-01	-4.21E-01	-3.98E-01	1.16E-02
HIS	14	O	52	HIS	14	C	1005	8.54E-01	-3.46E-01	-3.56E-01	-3.36E-01	1.01E-02
LYS	16	C	85	GLN	15	O	1023	8.32E-01	-4.23E-01	-4.36E-01	-4.09E-01	1.33E-02
LYS	16	C	85	LEU	17	O	1062	7.80E-01	-4.69E-01	-4.89E-01	-4.49E-01	1.95E-02
LYS	16	O	86	LYS	16	C	1039	8.59E-01	-3.98E-01	-4.08E-01	-3.89E-01	9.31E-03
LYS	16	O	86	LEU	17	C	1061	7.64E-01	-3.88E-01	-4.02E-01	-3.73E-01	1.43E-02
LEU	17	C	107	LEU	17	O	1062	8.42E-01	-3.23E-01	-3.28E-01	-3.18E-01	5.25E-03
VAL	18	H	131	LEU	17	O	1062	6.70E-01	-3.78E-01	-3.83E-01	-3.72E-01	5.50E-03
SER	26	O	243	ASN	27	C	1207	7.71E-01	-4.23E-01	-4.35E-01	-4.11E-01	1.21E-02
LYS	28	C	267	ASN	27	O	1208	7.99E-01	-4.21E-01	-4.40E-01	-4.02E-01	1.89E-02
LYS	28	C	267	ALA	30	O	1251	9.49E-01	-3.36E-01	-3.48E-01	-3.24E-01	1.21E-02
LYS	28	O	268	ASN	27	C	1207	8.13E-01	-3.74E-01	-3.90E-01	-3.57E-01	1.66E-02
LYS	28	O	268	LYS	28	C	1221	8.47E-01	-4.09E-01	-4.19E-01	-3.98E-01	1.06E-02
GLY	29	C	289	ALA	30	O	1251	7.56E-01	-3.95E-01	-4.14E-01	-3.76E-01	1.89E-02
GLY	29	O	290	LYS	28	C	1221	9.16E-01	-3.25E-01	-3.31E-01	-3.19E-01	5.87E-03
GLY	29	O	290	ALA	30	C	1250	7.48E-01	-3.63E-01	-3.79E-01	-3.46E-01	1.65E-02
ALA	30	C	296	ALA	30	O	1251	8.29E-01	-3.27E-01	-3.30E-01	-3.24E-01	2.83E-03
ILE	31	C	306	ALA	30	O	1251	8.11E-01	-3.40E-01	-3.46E-01	-3.34E-01	5.92E-03
ILE	31	C	306	ILE	32	O	1280	8.34E-01	-3.62E-01	-3.68E-01	-3.56E-01	5.70E-03
ILE	31	O	307	ALA	30	C	1250	8.18E-01	-3.75E-01	-3.81E-01	-3.69E-01	6.06E-03
ILE	31	O	307	ILE	31	C	1260	8.45E-01	-3.54E-01	-3.59E-01	-3.48E-01	5.42E-03
ILE	31	O	307	ILE	32	C	1279	8.24E-01	-3.69E-01	-3.76E-01	-3.62E-01	6.99E-03
ILE	31	O	307	ILE	32	H	1285	6.71E-01	-3.12E-01	-3.18E-01	-3.06E-01	5.90E-03
ILE	32	C	325	ILE	32	O	1280	8.49E-01	-3.51E-01	-3.54E-01	-3.48E-01	3.25E-03
GLY	33	C	344	ILE	32	O	1280	7.77E-01	-4.19E-01	-4.27E-01	-4.10E-01	8.08E-03
GLY	33	O	345	ILE	32	C	1279	7.97E-01	-3.21E-01	-3.27E-01	-3.15E-01	6.02E-03
LEU	34	C	351	ILE	32	O	1280	8.82E-01	-3.33E-01	-3.45E-01	-3.21E-01	1.20E-02
MET	35	C	370	MET	35	O	1325	8.68E-01	-3.26E-01	-3.31E-01	-3.20E-01	5.76E-03
VAL	39	H	422	GLY	38	O	1365	6.61E-01	-3.54E-01	-3.59E-01	-3.48E-01	5.80E-03
ILE	41	O	450	ILE	41	C	1403	8.56E-01	-3.47E-01	-3.54E-01	-3.39E-01	7.44E-03
ALA	42	OXT	471	LYS	28	C	1221	8.73E-01	-3.87E-01	-4.14E-01	-3.59E-01	2.73E-02
ALA	42	OXT	471	ALA	42	C	1422	8.28E-01	-3.30E-01	-3.44E-01	-3.16E-01	1.36E-02

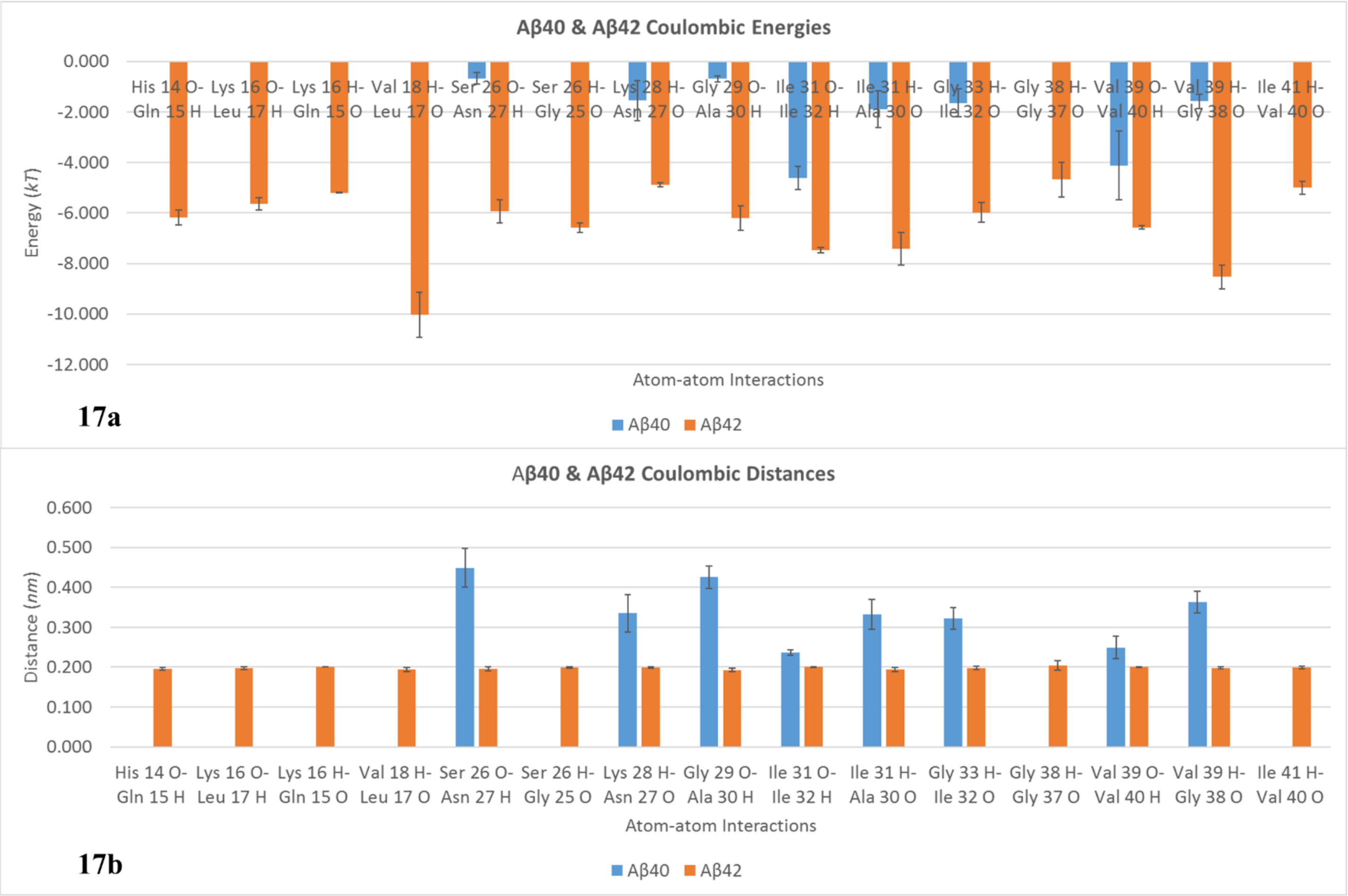

Supplementary Figure 17a & 17b: Atom-atom interactions imparting exceptionally strong hydrogen bonding energies in the 1:2 configuration of Aβ42 (PDB ID: 5KK3 by Colvin et al.) compared to Aβ40 (Aβ40 PDB ID: 2M4J by Lu et al.; Figure 17a) and their respective atom-atom interaction distances (Figure 17b). 95% confidence interval error bars included for analysis across all ensemble members. The first four, sixth, twelfth and fifteenth interactions were not observed in Aβ40. Interaction partners are presented as the residue, residue number in the sequence and the residue's atom of one chain (chain A for both strains) interacting with its partner atom in the 1:2 configuration (on the D-chain in Aβ40 or on the B-chain in Aβ42).

Supplementary Table 11: Ramachandran angle data for Aβ40 and Aβ42 for chains A-D (Aβ40, PDB ID: 2M4J) and chains A-B (Aβ42, PDB ID: 5KK3) for the 1:2 interaction configuration across ensemble structures. Data shown starts with residue 11 since structure data for previous Aβ42 structure, 2MXU, begins at residue 11.

	Aβ40				Aβ42			
	Chain A		Chain B		Chain A		Chain B	
	φ	ψ	φ	ψ	φ	ψ	φ	ψ
GLU 11	-	152.910 ± 0.711	-	151.350 ± 0.487	-	113.950 ± 24.475	-	118.180 ± 26.974
VAL 12	-153.140 ± 0.201	128.500 ± 0.357	-149.530 ± 0.042	130.000 ± 0.072	-117.590 ± 15.591	105.140 ± 39.660	-74.540 ± 45.725	119.550 ± 16.091
HIS 13	-135.180 ± 0.296	146.43 ± 0.743	-129.880 ± 0.120	149.470 ± 0.774	-78.850 ± 34.257	123.440 ± 19.662	-129.740 ± 12.850	59.500 ± 80.411
HIS 14	-165.320 ± 0.569	97.570 ± 0.373	-165.060 ± 0.128	104.570 ± 0.113	-112.800 ± 6.861	8.490 ± 26.078	-82.530 ± 21.870	-54.480 ± 5.829
GLN 15	-107.590 ± 2.008	-173.870 ± 0.751	-111.070 ± 2.030	-176.020 ± 0.175	-118.940 ± 9.519	130.480 ± 7.559	-108.380 ± 7.944	111.720 ± 8.560
LYS 16	-166.350 ± 1.700	97.950 ± 0.938	-162.970 ± 1.638	105.720 ± 0.414	-127.990 ± 7.067	113.950 ± 11.660	-114.180 ± 9.841	123.630 ± 10.138
LEU 17	-117.790 ± 5.123	158.580 ± 2.486	-124.310 ± 5.075	156.280 ± 2.852	-116.130 ± 10.380	118.390 ± 10.395	-127.340 ± 9.539	99.810 ± 9.019
VAL 18	-120.610 ± 9.229	124.710 ± 2.477	-112.980 ± 11.974	129.400 ± 2.469	-134.660 ± 8.727	142.220 ± 15.893	-119.870 ± 10.688	136.880 ± 6.200
PHE 19	-109.630 ± 16.980	133.770 ± 10.920	-110.030 ± 20.201	133.850 ± 7.521	-104.300 ± 12.827	-77.600 ± 32.583	-106.340 ± 10.302	-7.690 ± 50.073
PHE 20	-13.800 ± 67.407	34.610 ± 39.682	-12.140 ± 67.546	30.070 ± 41.404	-128.200 ± 27.892	140.560 ± 11.197	-121.350 ± 45.654	126.910 ± 23.841
ALA 21	-67.680 ± 12.282	-27.670 ± 50.357	-68.800 ± 13.231	-22.500 ± 50.612	-128.210 ± 16.614	-16.030 ± 46.343	-116.740 ± 14.870	-20.670 ± 44.007
GLU 22	-99.030 ± 13.950	117.940 ± 17.139	-102.000 ± 14.163	120.760 ± 16.175	-64.540 ± 83.952	-35.430 ± 110.939	-133.080 ± 27.921	0.990 ± 116.055
ASP 23	-153.20 ± 5.623	137.940 ± 1.318	-148.460 ± 3.594	139.130 ± 1.409	-32.560 ± 37.678	117.750 ± 30.390	-33.530 ± 38.003	120.640 ± 27.232
VAL 24	-67.650 ± 8.347	79.540 ± 83.862	-70.530 ± 8.460	80.410 ± 85.689	-122.630 ± 14.464	106.980 ± 63.638	-127.010 ± 14.332	148.350 ± 7.323
GLY 25	-114.140 ± 3.094	50.210 ± 2.290	-114.730 ± 2.429	54.310 ± 3.261	-85.780 ± 16.278	-66.280 ± 8.731	-89.550 ± 7.702	-81.750 ± 13.291
SER 26	-67.480 ± 6.196	-79.970 ± 87.939	-61.550 ± 4.009	-83.240 ± 87.483	-128.270 ± 4.435	86.420 ± 7.203	-114.350 ± 10.789	101.550 ± 6.123
ASN 27	-69.580 ± 1.699	86.480 ± 5.961	-69.260 ± 3.501	91.230 ± 4.565	-101.220 ± 5.073	109.860 ± 13.988	-109.390 ± 7.732	111.960 ± 7.995
LYS 28	-82.690 ± 5.998	135.220 ± 7.876	-87.910 ± 5.247	137.660 ± 8.087	-130.940 ± 13.604	120.640 ± 13.235	-124.450 ± 3.264	111.820 ± 13.668
GLY 29	-62.690 ± 0.809	-153.170 ± 10.428	-60.700 ± 2.630	-151.460 ± 9.486	92.140 ± 12.896	63.310 ± 17.138	88.190 ± 13.707	87.240 ± 10.903
ALA 30	-104.030 ± 6.560	122.180 ± 4.709	-105.320 ± 5.670	123.850 ± 5.584	-117.120 ± 13.986	118.410 ± 7.073	-130.790 ± 9.981	104.290 ± 7.173
ILE 31	-100.480 ± 8.486	130.830 ± 4.794	-103.190 ± 8.379	137.610 ± 3.972	-137.680 ± 6.799	135.100 ± 5.109	-121.360 ± 4.968	129.090 ± 4.516
ILE 32	-147.380 ± 4.687	-78.070 ± 95.222	-152.980 ± 5.812	-77.810 ± 96.409	-131.210 ± 1.822	122.630 ± 2.354	-130.630 ± 5.027	125.050 ± 2.517
GLY 33	63.670 ± 0.773	21.120 ± 16.942	63.990 ± 0.473	20.500 ± 16.506	-124.010 ± 66.107	35.360 ± 88.241	-31.520 ± 109.187	102.960 ± 59.067
LEU 34	-83.930 ± 8.423	*126.710 ± 3.472*	-84.830 ± 7.589	134.400 ± 2.025	-86.200 ± 18.673	-23.550 ± 34.074	-70.750 ± 13.752	-36.400 ± 40.085
MET 35	-159.790 ± 2.975	*150.550 ± 3.927*	-157.400 ± 0.722	160.480 ± 2.626	-110.360 ± 37.379	66.660 ± 10.096	-93.770 ± 36.336	83.650 ± 13.953
VAL 36	-154.990 ± 2.092	*160.240 ± 4.458*	-163.110 ± 1.703	161.350 ± 2.732	-123.330 ± 11.305	132.270 ± 9.093	-129.770 ± 12.213	110.210 ± 64.154
GLY 37	55.960 ± 0.472	*69.450 ± 2.115*	60.710 ± 0.370	64.440 ± 0.783	-122.380 ± 7.188	148.270 ± 13.632	-137.310 ± 14.763	152.790 ± 9.917
GLY 38	152.790 ± 7.822	7.410 ± 109.716	150.820 ± 7.315	8.980 ± 110.119	74.770 ± 12.909	77.900 ± 10.721	58.260 ± 6.782	87.890 ± 3.666
VAL 39	-122.680 ± 5.114	*153.420 ± 4.657*	-123.45 ± 4.172	151.310 ± 3.842	-120.650 ± 11.464	131.010 ± 7.715	-131.910 ± 4.738	125.820 ± 6.784
VAL 40	65.280 ± 0.705	-	73.420 ± 1.158	-	-135.520 ± 7.355	126.520 ± 3.370	-126.600 ± 5.845	126.520 ± 2.514
ILE 41	-	-	-	-	***-134.050 ± 6.343***	***153.130 ± 7.317***	***-129.660 ± 5.408***	***141.560 ± 4.078***
ALA 42	-	-	-	-	***-91.080 ± 5.953***	-	***-84.130 ± 2.845***	-

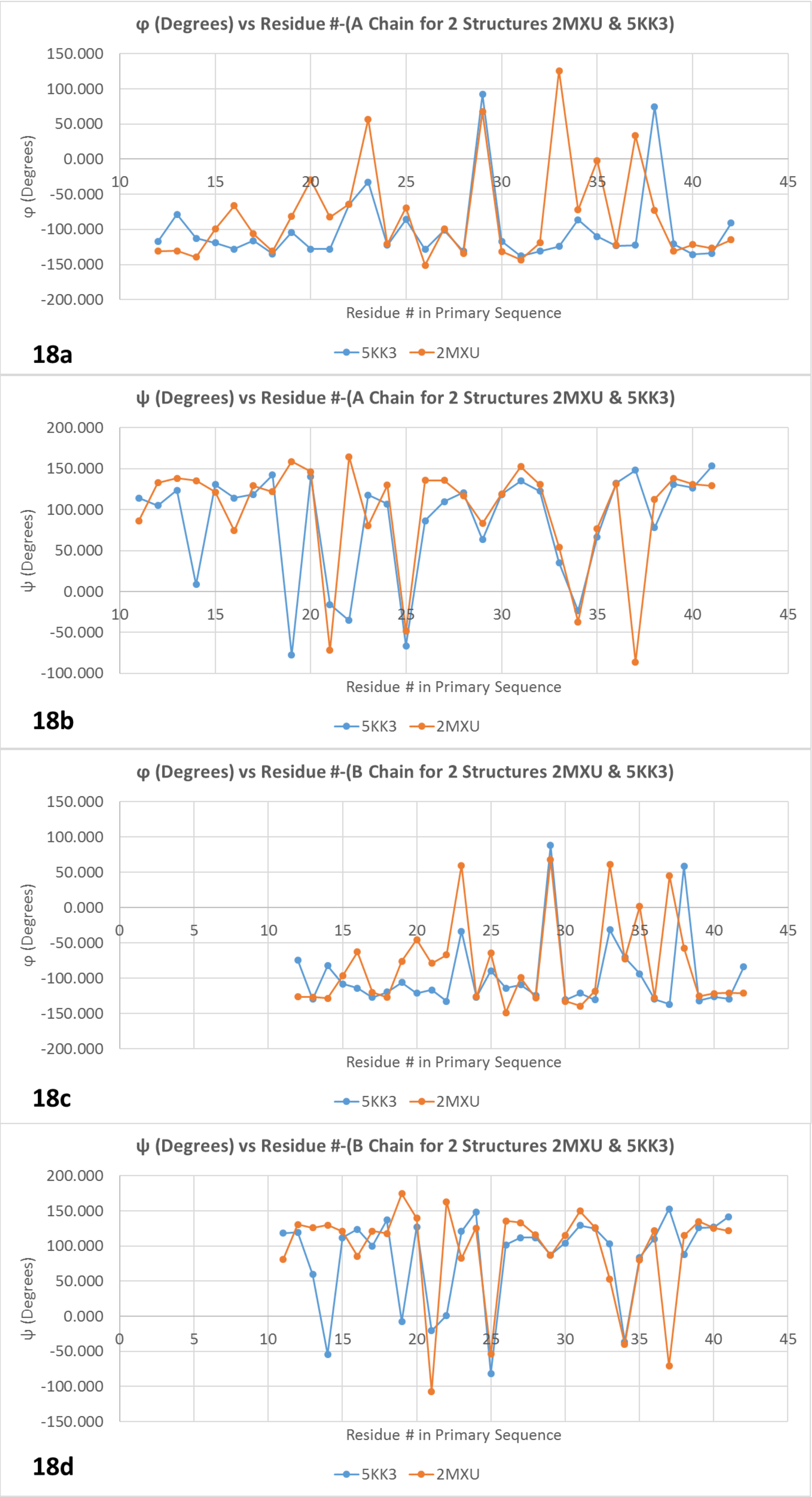

Supplementary Figure 18a-18d: Ramachandran angle profiles for Aβ42 structures 2MXU and 5KK3 featured as stacked curves for φ and ψ angle value distribution comparison.

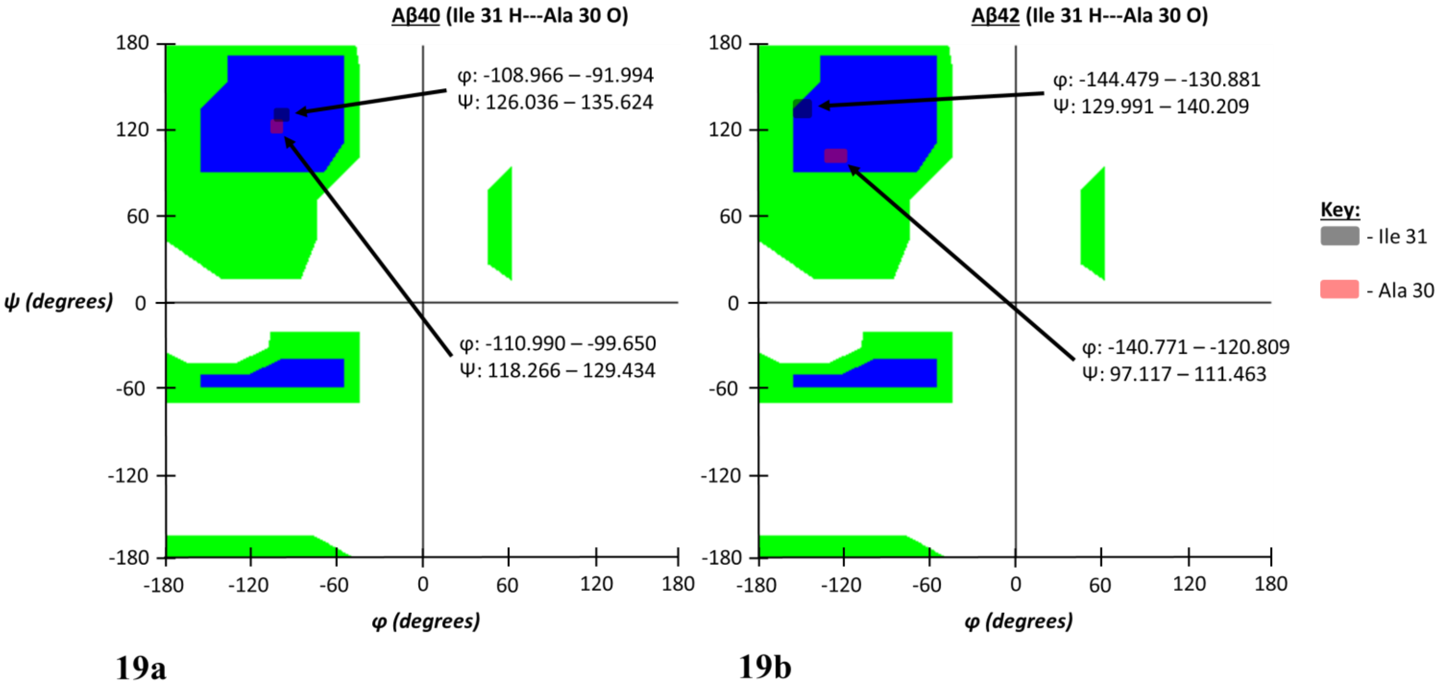

Supplementary Figure 19a-19b: Ramachandran angle profiles for an exceptionally strong atom-atom interaction (Ile 31 H interacting with Ala 30 O) for Aβ40 (PDB ID: 2M4J, 19a) and Aβ42 (PDB ID: 5KK3, 19b). Ranges for φ and ψ correspond to data spread according to 95% confidence interval analysis for all ensemble members as previously described. As stated before, the first atom is from the A chain of both isoforms and the second corresponds to the partner atom on the appropriate 1:2 interaction chain configuration. Note the retention of β-sheet secondary structure for both residues.

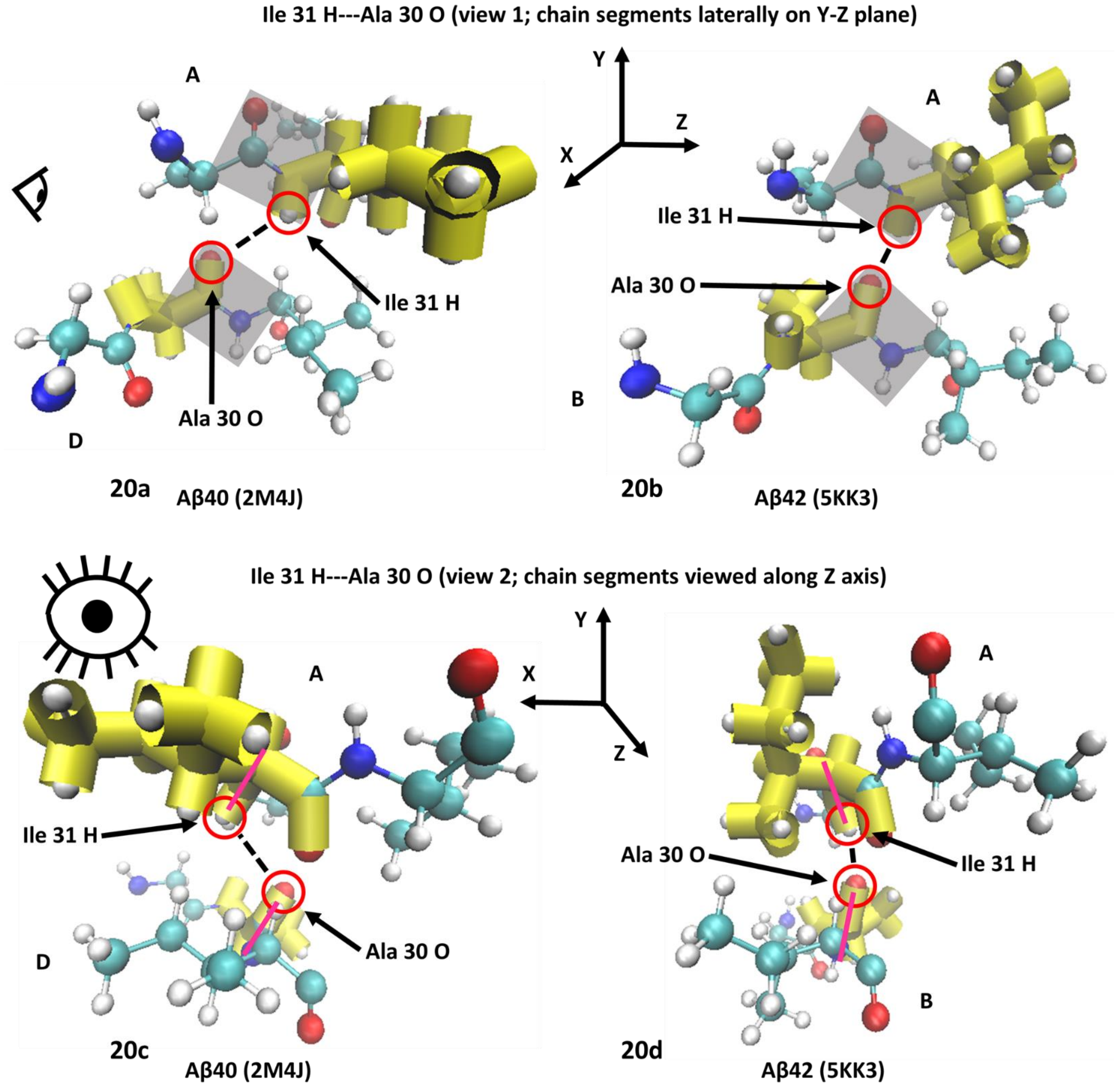

Supplementary Figure 20a-20d: Molecule representations of peptide plane alignment for Aβ40 (20a & 20c) and Aβ42 (20b & 20d). Aβ42 structure here is 5KK3. Shaded parallelograms in Figures 20a and 20b are the peptide planes for the residues whose atoms are participating in the hydrogen bonding. Figures 20c and 20d correspond to a view down the peptide bonds showing the peptide plane profile orientation in magenta. Eye icons indicate view perspective.

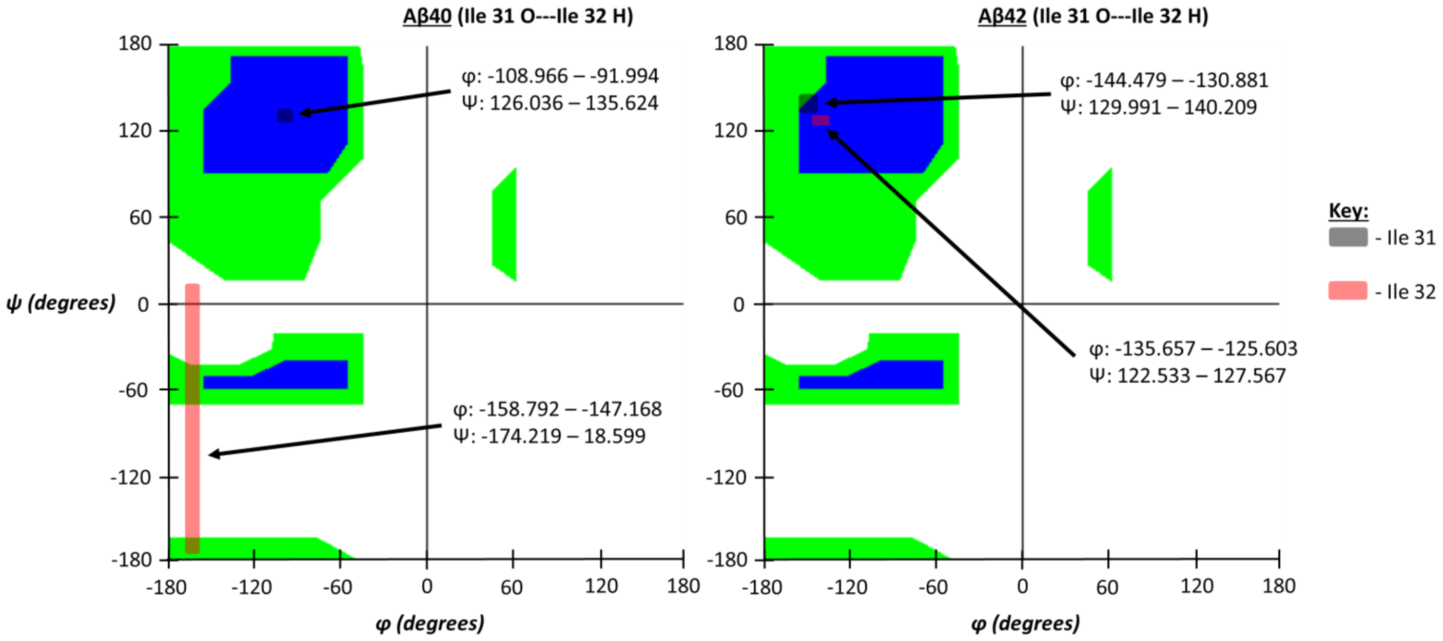

21a **21b**

Supplementary Figure 21a-21b: Ramachandran angle profiles for an exceptionally strong atom-atom interaction (Ile 31 O interacting with Ile 32 H) for Aβ40 (PDB ID: 2M4J, 21a) and Aβ42 (PDB ID: 5KK3, 21b). Ranges for φ and ψ correspond to data spread according to 95% confidence interval analysis for all ensemble members as previously described. As stated before, the first atom is from the A chain of both isoforms and the second corresponds to the partner atom on the appropriate 1:2 interaction chain configuration. Note the retention of β-sheet secondary structure for Ile 31 and the acquisition of β-sheet structure for Ile 32 in Aβ42 compared to Aβ40.

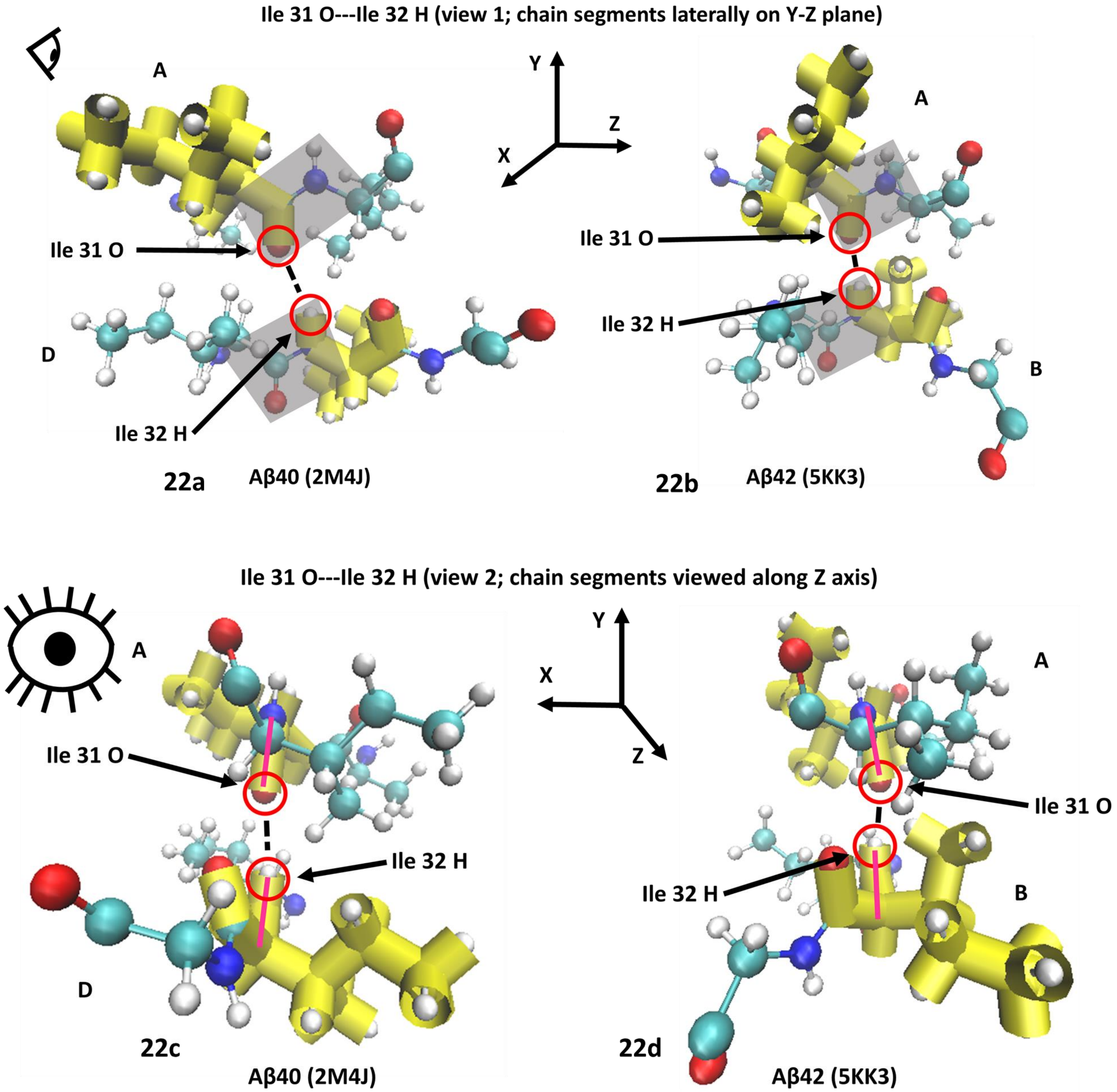

Supplementary Figure 22a-22d: Molecule representations of peptide plane alignment for Aβ40 (22a & 22c) and Aβ42 (22b & 22d). Aβ42 structure here is 5KK3. Shaded parallelograms in Figures 22a and 22b are the peptide planes for the residues whose atoms are participating in the hydrogen bonding. Figures 22c and 22d correspond to a view down the peptide bonds showing the peptide plane profile orientation in magenta. Eye icons indicate view perspective.

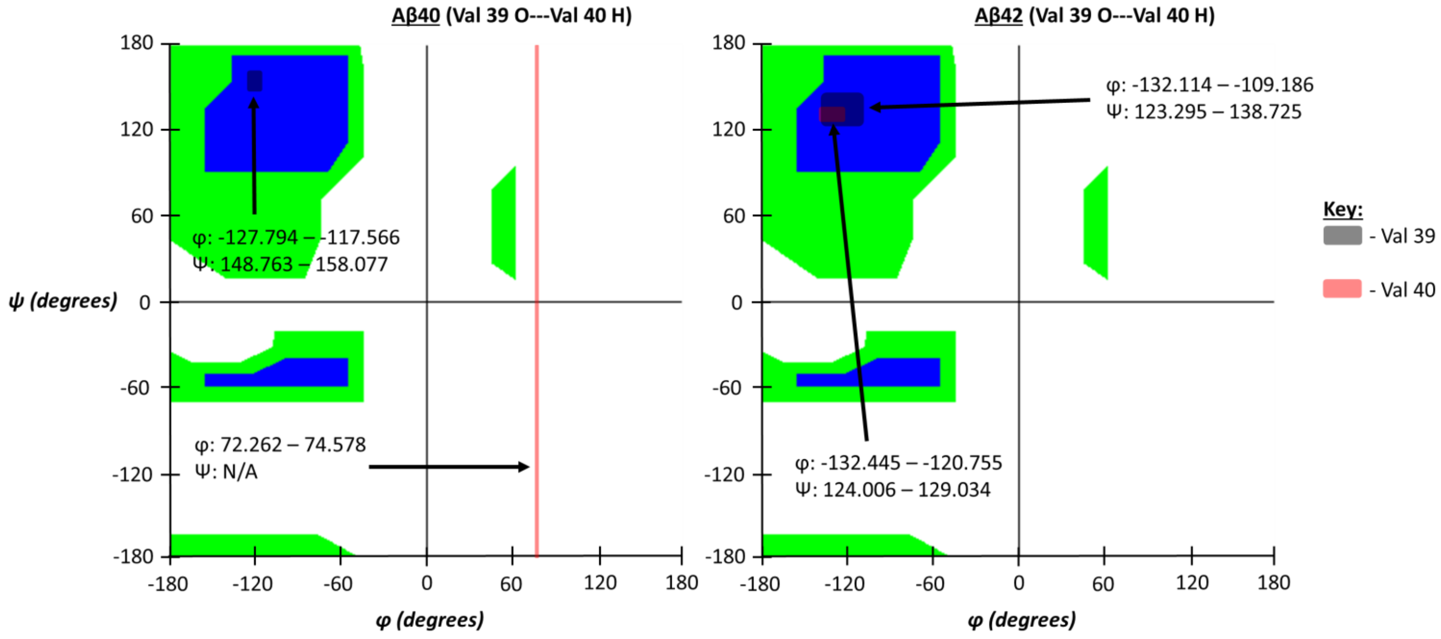

23a **23b**

Supplementary Figure 23a-23b: Ramachandran angle profiles for an exceptionally strong atom-atom interaction (Val 39 O interacting with Val 40 H) for Aβ40 (PDB ID: 2M4J, 23a) and Aβ42 (PDB ID: 5KK3, 23b). Ranges for φ and ψ correspond to data spread according to 95% confidence interval analysis for all ensemble members as previously described. As stated before, the first atom is from the A chain of both isoforms and the second corresponds to the partner atom on the appropriate 1:2 interaction chain configuration. Note the retention of β-sheet secondary structure for Val 39 and the acquisition of β-sheet structure for Val 40.

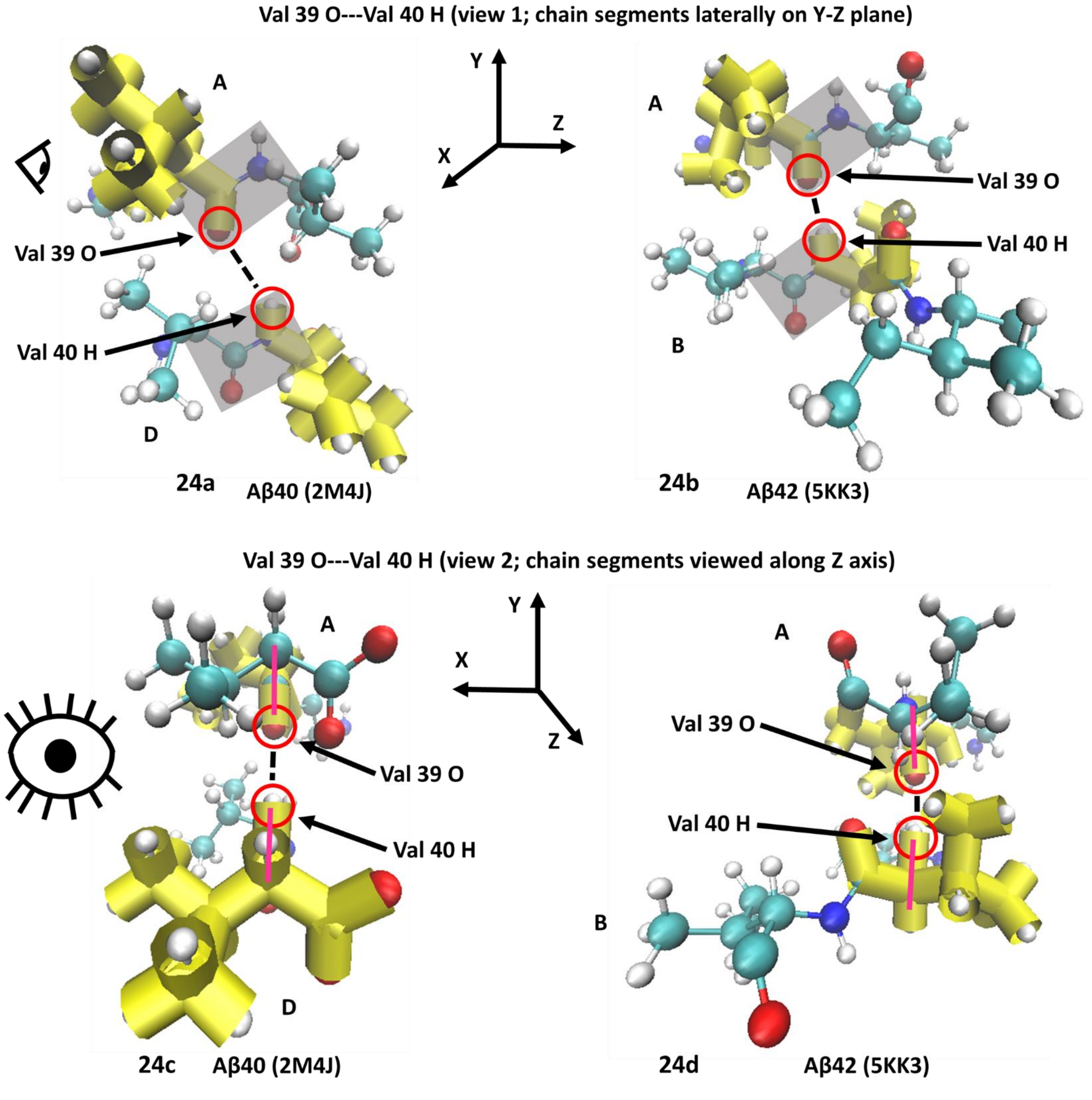

Supplementary Figure 24a-24d: Molecule representations of peptide plane alignment for Aβ40 (24a & 24c) and Aβ42 (24b & 24d). Aβ42 structure here is 5KK3. Shaded parallelograms in Figures 24a and 24b are the peptide planes for the residues whose atoms are participating in the hydrogen bonding. Figures 24c and 24d correspond to a view down the peptide bonds showing the peptide plane profile orientation in magenta. Eye icons indicate view perspective.
